# Weight regain after behavioural weight management programmes and its impact on quality of life and cost effectiveness: Evidence synthesis and health economic analyses

**DOI:** 10.1111/dom.14895

**Published:** 2022-11-02

**Authors:** Jamie Hartmann‐Boyce, Linda J Cobiac, Annika Theodoulou, Jason L. Oke, Ailsa R Butler, Peter Scarborough, Anastasios Bastounis, Anna Dunnigan, Rimu Byadya, F. D. Richard Hobbs, Falko F. Sniehotta, Ben Amies‐Cull, Paul Aveyard, Susan A. Jebb

**Affiliations:** ^1^ Radcliffe Primary Care Building, Nuffield Department of Primary Care Health Sciences University of Oxford Oxford UK; ^2^ School of Medicine and Dentistry Griffith University Brisbane Queensland Australia; ^3^ Nuffield Department of Primary Care Health Sciences University of Oxford Oxford UK; ^4^ NIHR Oxford Biomedical Research Centre Oxford University Hospitals NHS Foundation Trust Oxford UK; ^5^ NIHR Oxford Biomedical Research Centre, Nuffield Department of Population Health University of Oxford Oxford UK; ^6^ Division of Epidemiology and Public Health, School of Medicine University of Nottingham Nottingham UK; ^7^ Oxford University Hospitals NHS Foundation Trust Oxford UK; ^8^ Royal Free London NHS Foundation Trust London UK; ^9^ United Nations World Food Programme Cox's Bazar Bangladesh; ^10^ Faculty of Medical Sciences, Population Health Sciences Institute Newcastle University Newcastle upon Tyne UK; ^11^ Nuffield Department of Population Health University of Oxford Oxford UK

**Keywords:** cost‐effectiveness, diet, exercise, meta‐analysis, obesity, quality of life, weight management

## Abstract

**Aims:**

We used data from a recent systematic review to investigate weight regain after behavioural weight management programmes (BWMPs, sometimes referred to as lifestyle modification programmes) and its impact on quality‐of‐life and cost‐effectiveness.

**Materials and Methods:**

Trial registries, databases and forward‐citation searching (latest search December 2019) were used to identify randomized trials of BWMPs in adults with overweight/obesity reporting outcomes at ≥12 months, and after programme end. Two independent reviewers screened records. One reviewer extracted data and a second checked them. The differences between intervention and control groups were synthesized using mixed‐effect, meta‐regression and time‐to‐event models. We examined associations between weight difference and difference in quality‐of‐life. Cost‐effectiveness was estimated from a health sector perspective.

**Results:**

In total, 155 trials (n > 150 000) contributed to analyses. The longest follow‐up was 23 years post‐programme. At programme end, intervention groups achieved –2.8 kg (95%CI –3.2 to –2.4) greater weight loss than controls. Weight regain after programme end was 0.12‐0.32 kg/year greater in intervention relative to control groups, with a between‐group difference evident for at least 5 years. Quality‐of‐life increased in intervention groups relative to control at programme end and thereafter returned to control as the difference in weight between groups diminished. BWMPs with this initial weight loss and subsequent regain would be cost‐effective if delivered for under £560 (£8.80‐£3900) per person.

**Conclusions:**

Modest rates of weight regain, with persistent benefits for several years, should encourage health care practitioners and policymakers to offer obesity treatments that cost less than our suggested thresholds as a cost‐effective intervention to improve long‐term weight management.

**Registration:**

The review is registered on PROSPERO, CRD42018105744.

## 1 INTRODUCTION

Worldwide, almost 2 billion adults are estimated to live with overweight or obesity.^1^ Excess weight is a major risk factor for premature death from non‐communicable diseases^2^ and a recognized risk factor for musculoskeletal disorders.^3^, ^4^ Health care costs for people with obesity are estimated to be 36% higher than for people with a healthy weight.^5^

Behavioural weight management programmes (BWMPs; or lifestyle modification programmes) that aim to achieve weight loss through changes to diet, exercise, or both, are a mainstay of adult obesity treatment. Compared with no support, BWMPs can increase weight loss during the programme and up to 12 months from baseline, but uncertainty remains about their longer‐term effects.^6^

Weight loss followed by weight regain may undermine quality of life (QoL). A recent individual patient data meta‐analysis showed that weight loss was associated with improved QoL, even in the face of some weight regain, but included only five trials.^7^ However, qualitative data suggest that weight loss followed by weight regain is demoralizing, and a cohort study suggested it is associated with symptoms of depression.^8^, ^9^ Concerns about weight regain and the effects on QoL are barriers to prescription, uptake and engagement with BWMPs.^10^

The trajectory of weight regain is crucial to health economic modelling. English national guidelines suggest that BWMPs are cost‐effective if they lead to at least a sustained 1 kg weight loss in perpetuity.^11^ However, few studies show such a trajectory. In a 2007 review, Dansinger et al. mapped weight regain trajectories in intervention arms from 24 trials testing dietary counselling, and found regain of between 0.01 and 0.04 body mass index (BMI) units/month, with longest follow‐up at 60 months.^11^ Since then, many more studies have provided data, at substantially longer follow‐up points. Dansinger et al. have two further important limitations, namely its focus on dietary counselling as opposed to a wider range of interventions, and its reliance on rates of weight regain only within intervention arms. Comparator groups often lose weight in trials of BWMPs, and it is critical that when estimating programme effectiveness and cost‐effectiveness, data from trials are considered in relation to a control, not least, as people recruited to trials have a high motivation to lose weight and often succeed, at least in the short‐term.^12^

Here we aim to synthesize all available data to quantify weight change following BWMPs and its impact on QoL, modelling cost‐effectiveness implications compared with minimal or no intervention controls.

## 2 METHODS

The analyses presented here follow on from a systematic review investigating impact of programme characteristics on weight regain.^13^ Full methodological details can be found in the pre‐registered protocol.^14^

### 2.1 Search

We searched for randomized controlled trials in registries and 11 electronic databases in September 2018 using terms relevant to BWMPs (see Hartmann‐Boyce et al.^14^ and Table S1). Searches were run since database conception, restricted to full papers published in English. Authors were contacted where necessary for additional information. In December 2019 (before analyses), we ran forward citation searches for included or ongoing studies where the most recent publication was from January 2007 onwards to identify additional longer‐term follow‐up data.

### 2.2 Eligibility criteria

Studies had to be randomized controlled trials of adults (≥18 years) with overweight or obesity at study start (BMI of ≥25 kg/m^2^ or ≥23 kg/m^2^ in Asian populations). We excluded studies in pregnancy. In our analysis of weight regain trajectories, we restricted the dataset to studies that included a minimal intervention comparator arm and studies where the intervention group had on average lost weight compared with control (>0 kg) at end of the intervention (the difference did not have to be statistically significant).

We excluded interventions targeting multiple risk factors (e.g. including smoking cessation) and involving medications and/or surgery. Studies had to follow participants for ≥12 months, and measure weight change both at and after programme end. Where interventions varied in levels of support offered, we defined programme end as the point at which contact intensity markedly reduced (see Hartmann‐Boyce et al.^14^ for details).

### 2.3 Outcomes

Summary estimates of weight change after programme end (kg) and changes in QoL.

### 2.4 Screening, data extraction and risk of bias assessments

Two reviewers independently screened studies. Data extraction and risk of bias assessments (using the Cochrane risk of bias tool v1^15^) were done by one reviewer and checked by a second. Discrepancies were resolved by discussion or by referral to a third reviewer.

### 2.5 Data synthesis

We calculated the difference between weight and QoL in intervention groups from those in the control groups at programme end, and at each available time point after programme end. We converted all QoL measures such that higher scores indicated better QoL. Standardized mean differences were used for QoL because of use of multiple scales; they were then back converted to a common unit (Short‐Form 36^16^) for illustrative purposes. We extracted results as reported by authors; in nearly all studies, this meant that we used complete cases or multiple imputation data.

### 2.6 Statistical synthesis

We report pooled weighted averages at programme end for descriptive purposes. We synthesized changes in weight and QoL following programme end using three different approaches, using R 4.0.2.

- Model 1: mixed model with a random intercept for each study, regressing differences between intervention and control on time since programme end.
- Model 2: random effects meta‐regression (with time since programme end as predictor variable) based on baseline and final follow‐up.
- Model 3: time‐to‐event, evaluating the time at which half of the studies had an estimate for the difference between BWMP and control that reached zero.

We also used model 1 and model 2 to quantify associations between weight change and QoL. These analyses included all studies with available data.

Pre‐registered sensitivity analyses excluded studies at high risk of bias in any domain. To investigate possible publication bias, we assessed whether the length of follow‐up was related to the amount of weight lost at programme end, based on concerns that if a study had not found a significant difference in weight at programme end, it would be less likely to follow‐up participants because investigators may not want to use/obtain additional resource to collect these measures.

### 2.7 Modelling

We examined potential cost‐effectiveness of BMWPs using an existing health state model, PRIMEtime, to simulate lifetime impact on population health and expenditure on health and social care, under different weight regain trajectories. PRIMEtime has been previously used to evaluate obesity interventions successfully.^17^, ^18^ BMI is calculated from age‐ and sex‐specific height and weight data, both with and without the application of the body weight change related to an intervention. This estimated reduction in BMI reduces risk of developing a range of diseases including heart disease, stroke and cancer.^21^ PRIMEtime uses a proportional multi‐state lifetable model^19^, ^20^ to simulate two populations ageing and developing disease: one with the incidence of disease and risk factor distribution reflecting the current UK population and one after changing the body weight of participants, both calculated using population impact fractions. This translates the effects of weight reduction on incidence, prevalence and mortality from these diseases in the population. Utility weights quantify health impacts in quality‐adjusted life years (QALYs), and unit costs associated with health and social care enable cost‐effectiveness analysis of interventions.^21^, ^22^

We evaluated the cost at which intervention would be considered cost‐effective against a threshold of £20 000 per QALY, as recommended by the National Institute for Health and Care Excellence (NICE),^23^ and the cost at which intervention would be cost‐saving. We also quantified potential return on investment.^24^ To examine the effect of weight regain on cost‐effectiveness, we used PRIMEtime to evaluate, using input from the mixed model with random intercepts (model 1), the cost‐effectiveness of weight loss intervention with a linear regain of weight until all lost weight had been regained. Our intervention scenario was compared with a reference scenario that assumes a continuation of baseline obesity prevalence in the population. We assumed the weight loss intervention would be targeted at adults (18+ years) with a BMI of at least 30. Change in BMI was estimated from change in weight using energy balance equations from Hall et al.^25^

For the PRIMEtime analyses, we populated the model with data for a 2017 baseline year (Section S2). The model was then run over the lifetime of the 2017 population, and future cost offsets and QALYs were discounted back to the baseline year at a rate of 3.5% per annum, as recommended by NICE.^23^ We produced model outputs from a health sector perspective and a health and social care perspective, which additionally included cost offsets associated with social care in old age.

From the QALYs and cost offsets, we calculated the maximum per person cost for weight loss intervention to be considered cost‐effective against thresholds between £0 and £50 000 per QALY assuming an intervention achieved the observed mean weight loss at programme end and had the observed weight regain trajectory. Return on investment was estimated by monetizing the QALY at £60 000, the rate recommended in UK health impact assessment of government policies.^24^ We estimated 95% uncertainty intervals for all outcome measures using Monte Carlo analysis (5000 iterations). We assumed lognormal distributions of uncertainty around relative risks and unit costs, and normal distributions around utilities and weight regain. To model uncertainty in the weight regain trajectories, we prioritized the uncertainty around the start point of the trajectories (where most data were available). The Monte Carlo iterations were drawn from a lognormal distribution of weight loss at programme end, which was observed from the included studies. The rate of weight regain was modelled without uncertainty. To explore the main sources of uncertainty in our results we produced tornado plots for the primary outcome. To explore structural uncertainty in our model outcomes we conducted a sensitivity analysis where the weight regain trajectories were based on the random effects meta‐regression (model 2).

## 3 RESULTS

### 3.1 Search results

Details of the search can be found in Figure S1. In total, 155 studies provided sufficient data to be included in this paper (Table S3).

### 3.2 Characteristics of included studies

Table 1 shows summary data for included studies. Further detail is in Table S3 (primary references), Table S4 (full risk of bias assessments), Table S5 (key characteristics), Table S6 (baseline demographics) and Table S7 (intervention characteristics).

**TABLE 1:** Summary information on characteristics of studies contributing to statistical analyses

Characteristic	Number of studies (N = 155)		
Geographical region	North America: 79		
	South America: 1		
	Europe: 52		
	Asia: 6		
	Australia and New Zealand: 15		
	Africa: 1		
	Mixed (Australia and Europe): 1		
Recruitment method	Self‐initiated: 52		
	Prompted: 71		
	Required: 0		
	Not reported: 32		
Inclusion criteria restricted to those with a pre‐existing medical condition (e.g. only those with type 2 diabetes)	74		
Intervention content (selected characteristics), by study arm (not mutually exclusive)	By study arm: 395; diet and exercise: 220		
	Diet only: 64		
	Exercise only: 16		
	Partial meal replacements: 13		
	Total meal replacements: 10		
	Intermittent fasting: 2		
	Financial incentives (contingent on weight loss): 13		
Intervention delivery mode	By study arm, note some arms may include more than one mode		
	In person: 298		
	Telephone: 94		
	Internet: 47		
	App: 4		
	Print: 157		
	Video: 5		
	Text message: 7		
	Other (none of the above, see Table S7 for more detail): 24		
	Unclear: 4		
Intervention setting	By study arm, some arms may include >1 setting		
	Inpatient: 9		
	Residential: 1		
	Healthcare: 141		
	Community: 166		
	Workplace: 9		
	Home: 73		
	Median (IQR)		
Age in years	48.2 (11)		
Baseline BMI	33.7 kg/m^2^ (4.7)		
	Mean (min‐max) (n = 155 studies) in months		
Length of follow‐up (months)	25.4 (11.5‐360)		
Programme length (months) (most intensive intervention arm)	6.5 (0.7‐72)		
Programme length (months) (longest study arm)	7.1 (0.7‐72)		
Risk of bias domains	Low risk	Unclear risk	High risk
Overall risk of bias	36	82	37
Selection bias (random sequence generation and allocation concealment)	52	102	1
Detection bias	134	17	4
Attrition bias	130	4	21
Other risk of bias^a^	‐	‐	15

^a^ Only assessed where suspected, as per Cochrane guidance. Abbreviations: BMI, body mass index; IQR, interquartile range.

Most studies (n = 79) were conducted in North America, and 52 in Europe. The median BMI of participants at baseline was 33.7 kg/m^2^ and median age was 48.2 years. Mean programme length was 6.5 months. Mean follow‐up was just over 2 years.

Fifty‐six per cent of studies were at unclear risk of bias, 23% at low risk and 24% at high risk (Tables 1 and S4).

To be included, interventions had to involve changes to diet, physical activity, or both. Most interventions were delivered in‐person, supporting participants to change diet and physical activity (Table S7). The relationship of intervention characteristics to weight regain is examined elsewhere.^13^

### 3.3 Effects of interventions

### 3.4 Weight

Weight regain trajectories were calculated with data from 145 studies (n = 43 151). The longest follow‐up following the programme end was 23 years. At programme end, the average weight change in control arms was –2.1 kg (SD 3.3) and in intervention arms was –4.9 kg (SD 3.8), a mean (95% CI) weight difference in favour of intervention of –2.8 kg (95% CI –3.2 to –2.4).

One year after programme end (47 studies), the mean weight change from baseline was –4.0 kg (SD 3.8) in intervention arms and –1.6 (2.4) in control arms. At 5 years after programme end (three studies), the mean difference was –2.6 kg (1.5) in intervention arms and –0.6 (0.7) in control arms. Length of follow‐up was not statistically significantly associated with weight difference at programme end (Figure S2).

**FIGURE 1:**
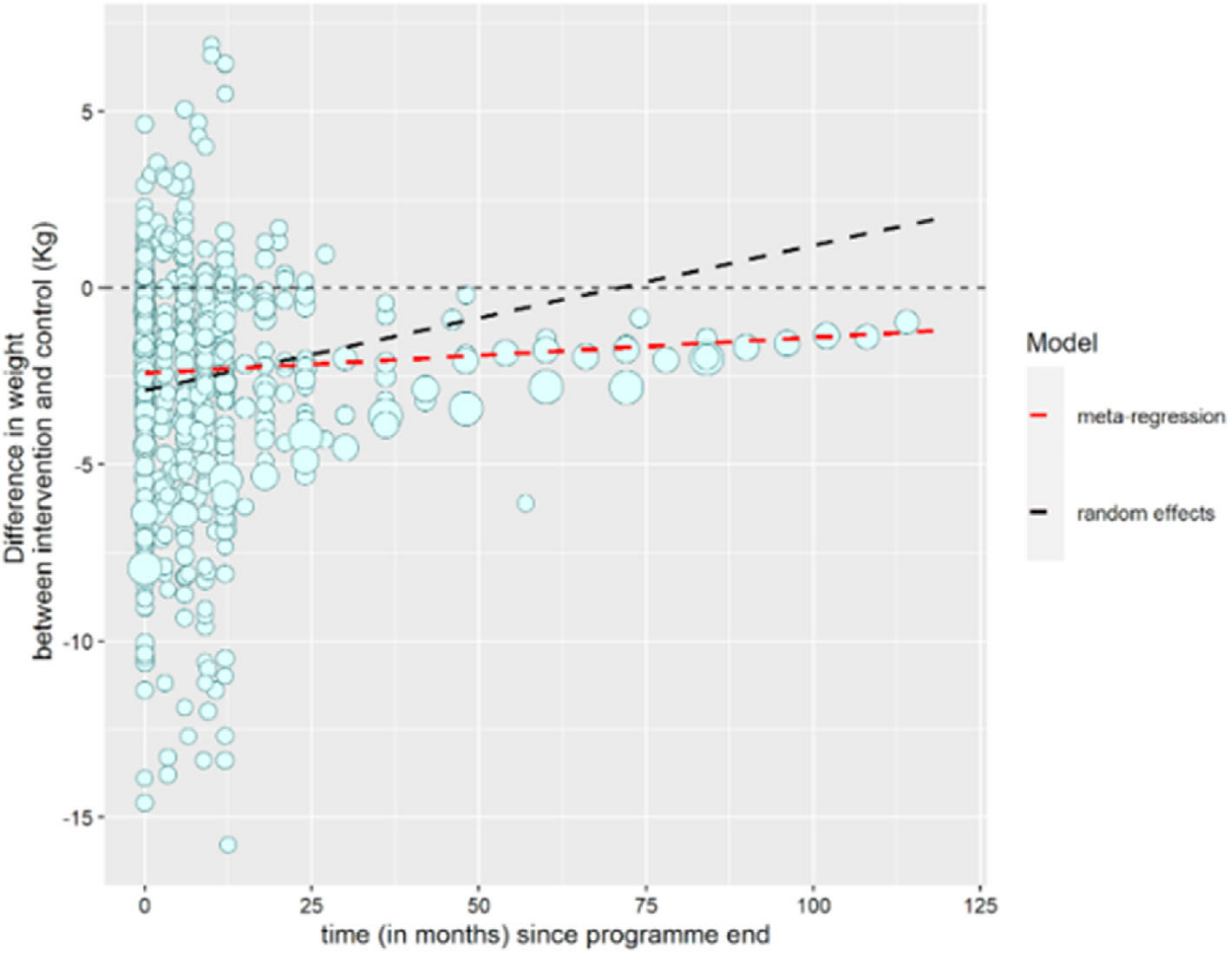
Difference in weight change between intervention and comparator arms by time since programme end. Dot size is proportional to number of participants in each study. Dashed lines represent estimates of average trend in weight change difference from random effects and meta‐regression

The mixed model (model 1) estimated an average weight regain relative to control after programme end of 0.027 kg (95% confidence interval [CI] 0.018‐0.036) per month (Figure 1). In meta‐regression (model 2), average weight regain (relative to control) after programme end was lower, at 0.010 kg (95% CI 0.002‐0.018)/month (Figure 1; more detail displayed in Figure S3). The time‐to‐event model (Figure S4) showed that the median time to reach no difference in weight between intervention and control was 168 months (14 years) after programme end; this sits between estimates from models 1 and 2. Removing studies at high risk of bias slightly decreased the estimate of average trend for the random effects (from 0.027 to 0.026 kg; 95% CI 0.015‐0.038) and meta‐regression (from 0.010 to 0.0064 kg; 95% CI –0.0065 to 0.019) models. After removing studies at high risk of bias from the time‐to‐event model, the median time could not be estimated, as weight did not return to control weight in at least half these studies.

### 3.5 Quality of life

Thirty‐five studies (n = 15 315) reported data on QoL. The longest follow‐up was 9 years after programme end. Mean difference between intervention and comparator in weight at programme end among those studies reporting QoL data were –3.7 kg (3.2). At programme end, mean (95% CI) standardized QoL was higher in intervention than control arms by 0.21 (0.18‐0.24). This equates to a difference of (median, IQR) 7.5 (0.6, 10) in the SF36. After programme end, the average trend in standardized QoL difference was estimated by the random effects model as –0.002 (95% CI –0.007 to 0.004)/month and in the meta‐regression model as –0.0014 (–0.0031 to 0.00023)/month (Figure S5). Both predicted a time to return to no difference from comparator of at least 9 years. The median time for difference in QoL in the intervention group to return to the control group in Kaplan‐Meier analysis was shorter, at 18 months (Figure S6).

For every kg of weight regained relative to control, standardized QoL was estimated to decline by 0.02 (95% CI 0.04 to 0.012) relative to control. Removing studies at high risk of bias did not significantly affect findings (Table S8).

### 3.6 Modelling of cost‐effectiveness

**FIGURE 2:**
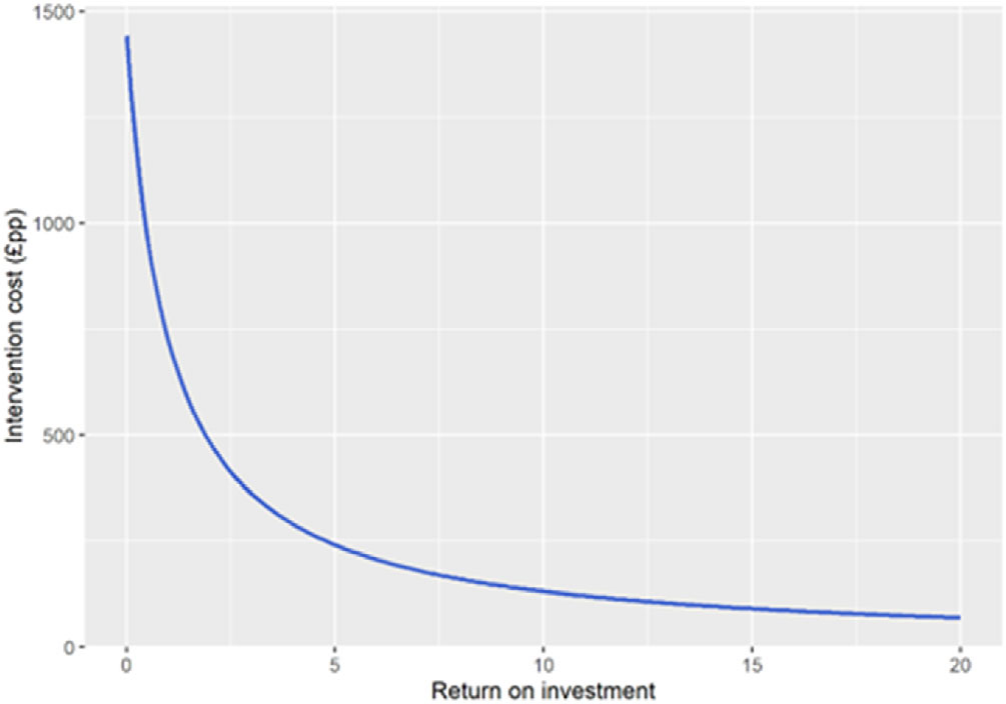
Return on investment with intervention to reduce weight

From a health sector perspective, assuming a weight difference at programme end of –2.8 kg and incorporating regain estimates from meta‐regression, BWMPs would be cost‐effective if delivered for under £560 (£8.80‐£3900). This amount reflects the total cost per person who receives the intervention, including intervention materials, provider costs and participant recruitment. To be cost‐saving, an intervention would need to cost less than £61 (£0.97‐£430) per person. Cost margins are slightly more favourable when the perspective is broadened to include costs of providing social care in old age, where the maximum cost of the intervention would be £630 for a willingness to pay threshold of £20 000/QALY (Table 2). Assuming that the benefits remained stable, lower cost estimates resulted in higher return on investment (Figure 2). The tornado plot (Figure S7) shows that the majority of uncertainty in the cost/QALY estimate is because of variance in the total amount of weight lost at programme end. There is a very clear right (positive) skew to this uncertainty because of the cost variable being lognormally distributed. Table S9 provides the same outcomes under our sensitivity analysis using weight regain trajectories from model 2. Because the weight regain trajectory predicted by the second model was slower than the primary analysis using model 1, the benefits of BMI reductions accrued over longer periods, so slightly higher intervention costs can be tolerated at each willingness to pay threshold. For example, with a health sector perspective and using a threshold of £20 000 per QALY, our sensitivity analysis suggests that the maximum intervention cost is £890 (£8.90‐£5600).

**TABLE 2:** Total per person cost of intervention at the threshold of intervention cost‐savings and at the threshold range for cost‐effectiveness defined by NICE for a weight difference of 2.8 kg at programme end

Threshold	Perspective	
	Health care	Health and social care
Linear weight regain after weight loss		
Cost‐saving (£0 per QALY)	£61 (£0.97‐£430)	£120 (£1.8‐£870)
NICE lower (£20 000 per QALY)	£560 (£8.8‐£3900)	£630 (£9.8‐£4300)
NICE upper (£30 000 per QALY)	£810 (£13‐£5500)	£880 (£14‐£6000)
No weight regain after weight loss		
Cost‐saving (£0 per QALY)	£210 (£23‐£790)	£450 (£49‐£1700)
NICE lower (£20 000 per QALY)	£2000 (£230‐£7100)	£2200 (£260‐£8100)

Abbreviations: NICE, National Institute for Health and Care Excellence; QALY, quality‐adjusted life years.

**FIGURE 3:**
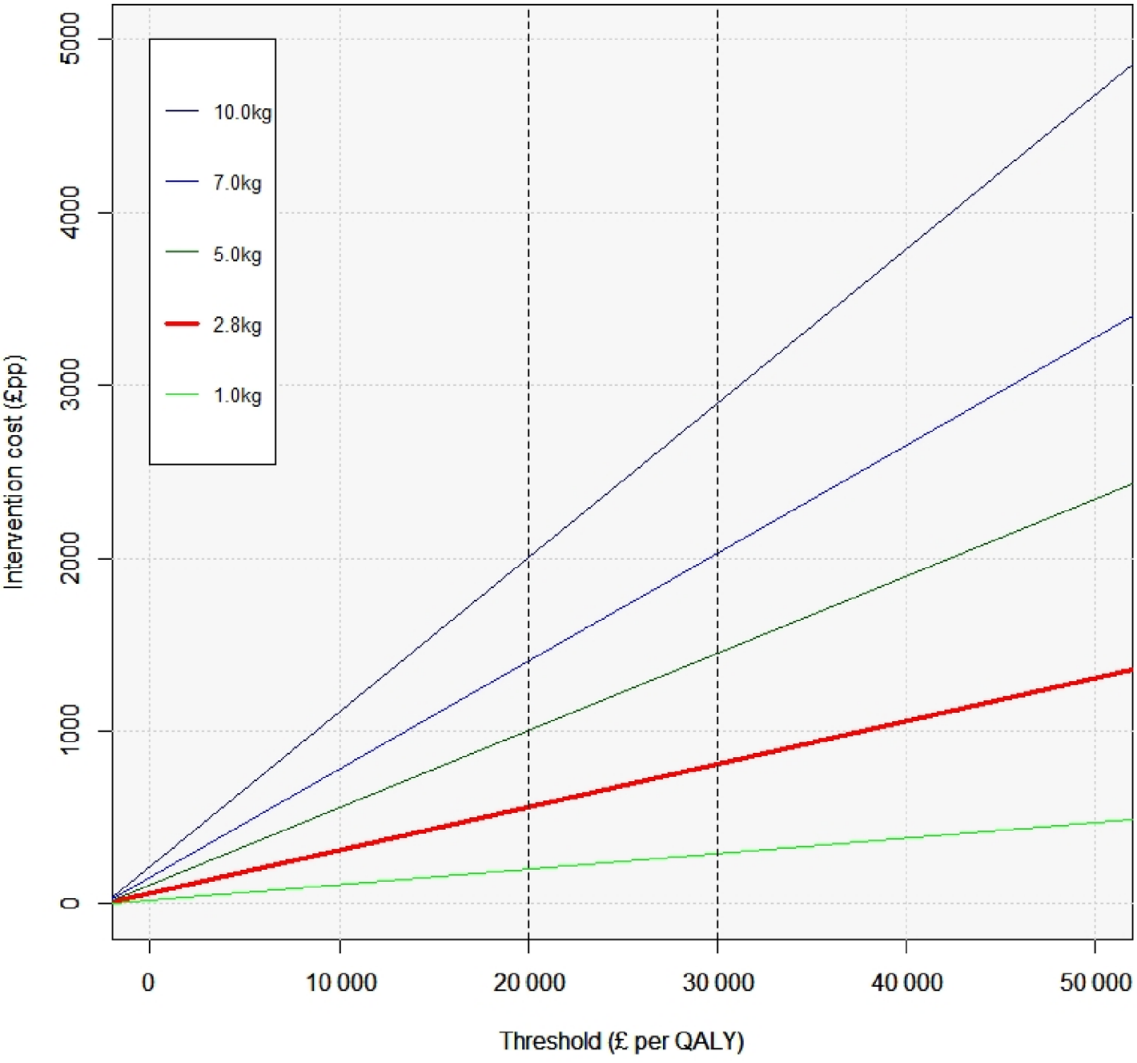
Intervention unit cost needed to achieve cost‐effectiveness at different thresholds for different levels of initial weight loss (assumes health benefits for different weight losses scales linearly). Abbreviation: QALY, quality‐adjusted life year

Our primary estimates are based on the average 2.8 kg weight loss observed across the trials at the intervention end. Interventions that are more effective could cost more and be cost‐effective/saving, while interventions less effective would need to be less costly. Figure 3 shows the unit costs that would be needed to achieve cost‐effectiveness at different thresholds for various differences at programme end in weight loss between intervention and control groups (assuming that health benefits scale linearly). For example, Figure 3 shows that a weight management programme reducing weight by 7 kg more than control would be cost‐effective (at a threshold of £20 k per QALY) if it costs £1400 or less.

## 4 DISCUSSION

To our knowledge, this is the largest ever study quantifying weight change after BWMPs cease, and the impact on QoL, and cost‐effectiveness given the weight regain. We estimated that weight regain occurs at a rate of approximately 0.01‐0.03 kg/month (0.12‐0.32 kg/year) compared with comparator groups, meaning weight takes at least 5 years after the programme to reach no difference between intervention and a lower intensity intervention or no intervention. QoL was improved compared with control at programme end; the difference diminished slowly over 9 years, the rate depending on the differences between groups in weight regain. Allowing for weight regain, we estimate that BWMPs would be cost‐effective, from a health service perspective, if delivered for under £560 (£8.80‐£3900) per person. Assuming no regain—as some models currently do—changes this figure to £2000 (£230‐£7100).

Weight regain following BWMPs is an area rich in speculation but with little formal investigation. Dansinger et al. estimated regain of between 0.01 and 0.04 BMI units per month from 24 studies, which was somewhat higher than our estimate, with the longest follow‐up at 60 months.^26^ Our estimates of weight regain are based on data from 145 studies with maximum follow‐up of 23 years. Moreover, Dansinger et al. reported rates of weight regain within intervention arms. In our data, both intervention and comparator groups lost weight relative to baseline and both groups weighed less than baseline up to 5 years.

Our review also examined changes in QoL. There was no evidence that QoL was worse than baseline following weight regain. Our data provide reassurance that population effects on wellbeing would probably be positive for several years after programme end. However, the short‐term data were highly heterogeneous and bear further examination to explore the potential differences; we cannot exclude that QoL for some individuals was harmed by engaging in BWMPs. Our data imply that the improvements in QoL observed from an individual patient data meta‐analysis of BWMPs are not solely because of enhanced wellbeing from provider contact/programme involvement, but are linked to weight change.^7^

Cost‐effectiveness is related to the initial weight loss achieved compared with the counterfactual, and variability in both weight regain and initial weight loss determine the health economic benefits of BWMPs. Our results are based on the mean weight loss observed in our meta‐analysis. For programmes that typically result in greater initial weight loss, such as meal replacement interventions, costs could be higher and still be cost‐effective. The health benefits associated with intervention are largely proportional to weight loss but with diminishing returns because of additional costs of health care associated with added years of healthy life. It is worth noting that our model took no account of the direct benefits of weight loss on QoL that our review reports, hence the current estimates of cost/QALY may be underestimates. Further, while the cost‐effectiveness model was based on the general population, we would expect the higher disease rates in overweight and obese populations to translate into a greater impact on incidence and better cost‐effectiveness for a given level of weight loss.

Our review has some important limitations. Study quality and reporting was often an issue. Because of the lack of reporting standards for weight loss trials, we made several assumptions. We used a predefined, although essentially arbitrary definition of programme end, defined as a marked step‐down in intensity. Variations in how missing data were imputed—or not—by study authors may have introduced spurious differences between studies and biased estimates in favour of BWMPs. Our focus on differences over time within studies (as opposed to across studies) means we are less influenced by this issue than standard meta‐analyses of effectiveness at specific time points, but never‐the‐less, this remains a limitation, in particular, regarding estimates of absolute changes in weight and quality of life at specific time points. The proportional multistate lifetable model structure that PRIMEtime uses has been developed for non‐communicable disease modelling with the advantage of limiting the numbers of simulated states, despite multiple disease endpoints.^27^ This comes with a trade‐off that disease endpoints are assumed to be because of independent probabilities (with the exceptions of type 2 diabetes with ischaemic heart disease or stroke). This should not bias the modelled outcomes, as QALYs are calculated additively from utility weights and the increased costs of multimorbidity are accounted for through modelling cost uncertainty on a lognormal distribution. The impact of weight regain on obesity complications and risk factors will be examined in a companion publication.

We cannot rule out publication bias, although reassuringly, our test for publication bias was not significant. Limiting our searches to English will have omitted some relevant trials, but there is no reason to think that the rate of weight gain or the relationship between weight change and QoL would be different from those published in English. As the focus of this review was on weight regain, when we estimated regain trajectories, we restricted our study set to those in which weight had been lost relative to a control condition at programme end; this was necessary given that the focus of our review is on weight regain following weight loss. Our latest search date was December 2019, hence we will have missed studies published subsequently; there is no specific reason to think studies published before 2020 would find notably different outcomes from those published after. Despite being the largest review of its type to date, data are still limited; in particular, only three studies followed up beyond 5 years. The mean follow‐up of just over 2 years from programme end compares unfavourably with current levels of reporting for other chronic diseases, and surgical and medical obesity treatments. Finally, the considerable variation between weight trajectories after programme end across the studies meant our planned model did not fit the data neatly. We therefore presented results using three different models. Although the models produced broadly consonant results, in a separate publication, we found no evidence that most differences in the dietary and physical activity recommendations and behavioural components explained variation in weight regain trajectories between studies.^13^ This remains an area for further research.

These estimates derive from a meta‐analysis that compared more intensive BWMPs with either less intensive or no intervention comparators. This allowed us to estimate the rate of weight regain after initial loss, the focus of this analysis, but not the effect of BWMPs versus no intervention. It is plausible that these regain estimates apply to other treatments for obesity, such as pharmacotherapy, but this requires further investigation, particularly noting that modalities that provide greater weight loss or less regain may be more cost‐effective than BWMPs. The review reassures people living with obesity that the positive effects of BWMPs are not immediately negated by rapid weight regain after programme end, nor that QoL will be negatively impacted. It should encourage health care practitioners and policymakers that BWMPs that cost less than our suggested thresholds can be a cost‐effective intervention to improve long‐term weight management of obesity, despite weight regain after programme end.

## AUTHOR CONTRIBUTIONS

JHB, PA, SJ, FS, FDRH, PS and JO conceived and designed the review. JHB, RB, AT, ARB, AB and AD conducted screening. JHB, AT, ARB, AB and AD conducted data extraction and assessed studies for risk of bias. JO conducted the main statistical analyses. LC, PS and BAC designed and conducted cost‐effectiveness analyses. JHB prepared the first draft of the review, with further input from AT, PA and SJ and all authors contributed to the interpretation and final write up.

## FUNDING INFORMATION

This research was funded by the British Heart Foundation, PG/17/68/33247 and National Institute for Health Research (NIHR) Oxford Biomedical Research Centre (BRC) Obesity, Diet and Lifestyle Theme. JHB, PA, SAJ and FDRH are part‐funded by NIHR Oxford BRC. JHB is also part funded by an NIHR Cochrane Programme Grant. PA and SAJ are NIHR senior investigators and are funded by NIHR Oxford and Thames Valley Applied Research Collaboration. FDRH also acknowledges part‐funding from the NIHR Applied Research Collaboration Oxford & Thames Valley, and the NIHR Oxford Medtech and In‐Vitro Diagnostics Co‐operative. PS is funded by a BHF fellowship (FS/15/34/31656). The views expressed are those of the authors and not necessarily those of the BHF, the NHS, the NIHR or the Department of Health and Social Care. Study sponsors had no role in study design, collection, analysis, interpretation, writing, or the decision to submit the manuscript for publication.

## CONFLICT OF INTEREST

PA and SAJ were investigators on a trial of a low energy total diet replacement programme funded by Cambridge Weight Plan. PA spoke at a seminar at the Royal College of General Practitioners conference that was funded by Novo Nordisk. Neither of these led to personal payments. All other authors declare that they have no competing interests.

### PEER REVIEW

The peer review history for this article is available at https://publons.com/publon/10.1111/dom.14895.

## Supporting information


**Table S1.** MEDLINE search strategy
**Table S2.** Data inputs for PRIMEtime analysis
**Table S3.** Included studies reference list
**Table S4.** Risk of bias of included studies
**Table S5.** Characteristics of included studies
**Table S6.** Baseline demographics
**Table S7.** Intervention characteristics
**Table S8.** Sensitivity analyses
**Table S9.** Cost‐effectiveness sensitivity analysis using outputs from meta‐regression (model 2): total per person cost of intervention at the threshold of intervention cost‐savings and at the threshold range for cost‐effectiveness defined by the National Institute for Health and Care Excellence (NICE) for a weight difference of 2.5 kg at programme end.
**Figure S1.** PRISMA diagram of study flow
**Figure S2.** Mean difference in weight change at programme end mapped against follow‐up time
**Figure S3.** Weight regain trajectory as per the linear model overlaid on the observed difference in weight change between intervention and comparator arms by time since programme end. Dot size is proportional to number of participants in each study. The red dashed line represents estimates of average trend in weight change difference from the random effects model. The green dotted line represents the meta‐regression model.
**Figure S4.** Kaplan Meier plot showing time for intervention group mean weight to reach that of control group
**Figure S5.** Difference in standardised quality of life change between intervention and comparator arms by time since programme end (higher = better). Dots size is proportional to number of participants in the study. Dashed lines represent estimates of average trend from model 1 and model 2
**Figure S6.** Kaplan Meier plot showing probability that study arm that had quality of life difference at programme end is zero on follow‐up
**Figure S7.** Tornado plot for the primary outcome, centred on median outcome from 1500 runs of the PRIMEtime model.

## Data Availability

As this is an evidence synthesis, data is available in the public domain. Details of our analyses and the files used for these are available from the authors on request.
