# Supplementary material for: Weight regain after behavioural weight management programmes and its impact on quality of life and cost effectiveness: Evidence synthesis and health economic analyses
Source: Diabetes Obes Metab. 2022 Nov 2;25(2):526–35. doi: 10.1111/dom.14895 (PMC10092406; doi:10.1111/dom.14895)
Supplement: Supplementary file 1 — Table S1. MEDLINE search strategy Table S2. Data inputs for PRIMEtime analysis Table S3. Included studies reference list Table S4. Risk of bias of included studies Table S5. Characteristics of included studies Table S6. Baseline demographics Table S7. Intervention characteristics Table S8. Sensitivity analyses Table S9. Cost‐effectiveness sensitivity analysis using outputs from meta‐regression (model 2): total per person cost of intervention at the threshold of intervention cost‐savings and at the threshold range for cost‐effectiveness defined by the National Institute for Health and Care Excellence (NICE) for a weight difference of 2.5 kg at programme end. Figure S1. PRISMA diagram of study flow Figure S2. Mean difference in weight change at programme end mapped against follow‐up time Figure S3. Weight regain trajectory as per the linear model overlaid on the observed difference in weight change between intervention and comparator arms by time since programme end. Dot size is proportional to number of participants in each study. The red dashed line represents estimates of average trend in weight change difference from the random effects model. The green dotted line represents the meta‐regression model. Figure S4. Kaplan Meier plot showing time for intervention group mean weight to reach that of control group Figure S5. Difference in standardised quality of life change between intervention and comparator arms by time since programme end (higher = better). Dots size is proportional to number of participants in the study. Dashed lines represent estimates of average trend from model 1 and model 2 Figure S6. Kaplan Meier plot showing probability that study arm that had quality of life difference at programme end is zero on follow‐up Figure S7. Tornado plot for the primary outcome, centred on median outcome from 1500 runs of the PRIMEtime model. [file DOM-25-526-s001.docx]

CONTENTS

[SUPPLEMENTAL TABLES 2](#_Toc114582138)

[Table 1. MEDLINE search strategy 2](#_Toc114582139)

[Table 2. Data inputs for PRIMEtime analysis 5](#_Toc114582140)

[Table 3. Included studies reference list 6](#_Toc114582141)

[Table 4. Risk of bias of included studies 14](#_Toc114582142)

[Table 5. Characteristics of included studies 70](#_Toc114582143)

[Table 6. Baseline demographics 85](#_Toc114582144)

[Table 7. Intervention characteristics 96](#_Toc114582145)

[Table 8. Sensitivity analyses 151](#_Toc114582146)

[Table 9. Cost-effectiveness sensitivity analysis using outputs from meta-regression (model 2): total per person cost of intervention at the threshold of intervention cost-savings and at the threshold range for cost-effectiveness defined by the National Institute for Health and Care Excellence (NICE) for a weight difference of 2.5kg at programme end. 151](#_Toc114582147)

[SUPPLEMENTAL FIGURES 152](#_Toc114582148)

[Figure 1. PRISMA diagram of study flow 152](#_Toc114582149)

[Figure 2. Mean difference in weight change at programme end mapped against follow-up time 153](#_Toc114582150)

[Figure 3. Weight regain trajectory as per the linear model overlaid on the observed difference in weight change between intervention and comparator arms by time since programme end. Dot size is proportional to number of participants in each study. The red dashed line represents estimates of average trend in weight change difference from the random effects model. The green dotted line represents the meta-regression model. 154](#_Toc114582151)

[Figure 4. Kaplan Meier plot showing time for intervention group mean weight to reach that of control group 155](#_Toc114582152)

[Figure 5. Difference in standardised quality of life change between intervention and comparator arms by time since programme end (higher = better). Dots size is proportional to number of participants in the study. Dashed lines represent estimates of average trend from model 1 and model 2 156](#_Toc114582153)

[Figure 6. Kaplan Meier plot showing probability that study arm that had quality of life difference at programme end is zero on follow-up. 157](#_Toc114582154)

[Figure 7. Tornado plot for the primary outcome, centred on median outcome from 1500 runs of the PRIMEtime model. 158](#_Toc114582155)

# SUPPLEMENTAL TABLES

### Table 1. MEDLINE search strategy

| # ▲ | **Searches** |
| --- | --- |
| 1 | overweight/ or obesity/ or obesity, morbid/ or obesity, abdominal/ |
| 2 | (overweight or over weight or overeat* or over eat* or overfeed* or over feed*).ti,ab. |
| 3 | obes*.ti,ab. |
| 4 | 1 or 2 or 3 |
| 5 | Weight Reduction Programs/ |
| 6 | Weight Loss/ and (modific* or therap* or intervention* or strateg* or program* or management or scheme* or group* or club? or pathway* or service?).ti,ab. |
| 7 | ((weight adj1 (los* or reduc* or manag*)) and (modific* or therap* or intervention* or strateg* or program* or management or scheme* or group* or club? or pathway* or service?)).ti,ab. |
| 8 | obesity/dh, pc, th or obesity, abdominal/dh, pc, th or obesity, morbid/dh, pc, th |
| 9 | diet therapy/ or diet, fat-restricted/ or diet, reducing/ or caloric restriction/ or diet, carbohydrate-restricted/ |
| 10 | Dietetics/ed, mt |
| 11 | (diets or dieting).ti,ab. |
| 12 | ((low calorie* or low energy or hypocaloric or calorie control* or low fat* or fat control* or low carbohydrate* or carbohydrate control*) adj2 diet*).ti,ab. |
| 13 | (health* adj1 eating).ti,ab. |
| 14 | (diet* adj2 (modific* or therap* or intervention* or strateg* or program* or management or scheme* or group* or club?)).ti,ab. |
| 15 | (nutrition* adj2 (modific* or therap* or intervention* or strateg* or program* or management or scheme*)).ti,ab. |
| 16 | (Weight Watchers or slimming world or lighterlife or lighter life or jenny craig or diet chef or nutrisystem or slimfast or slim fast or medifast or optifast or modifast or nutrilett or hmr or cambridge diet).ti,ab. |
| 17 | (commercial* adj2 (program* or intervention? or weight loss or diet?)).ti,ab. |
| 18 | (meal? adj2 replace*).ti,ab. |
| 19 | ((preprepared or pre-prepared or prepared) adj2 (meal? or food? or snack? or portion?)).ti,ab. |
| 20 | ((prepack* or pre-pack*) adj2 (meal? or food? or snack? or portion?)).ti,ab. |
| 21 | (eating plan? or meal plan* or planned meal? or planned menu?).ti,ab. |
| 22 | (portion control* adj2 (diet? or meal? or food? or weight loss)).ti,ab. |
| 23 | (liquid adj2 (diet? or meal? or food?)).ti,ab. |
| 24 | (replace* adj (snack? or drink? or food? or liquid?)).ti,ab. |
| 25 | shake?.ti,ab. |
| 26 | (isoenergetic adj (diet? or food? or meal?)).ti,ab. |
| 27 | or/5-26 |
| 28 | exp *exercise/ and (modific* or therap* or intervention* or strateg* or program* or management or scheme* or group* or club?).ti,ab. |
| 29 | exercise therapy/ |
| 30 | ((exercis* or physical activity) adj5 (therap* or intervention* or program* or scheme* or group* or club? or class* or session*)).ti,ab. |
| 31 | ((exercis* or physical activity) adj5 (chang* or modif* or increas*)).ti,ab. |
| 32 | ((walk* or step* or jog* or run* or bicycl* or cycle* or cycling) adj5 (therap* or intervention* or program* or scheme* or group* or club? or class* or session*)).ti,ab. |
| 33 | ((walk* or step* or jog* or run* or bicycl* or cycle* or cycling) adj5 (chang* or modif* or increas*)).ti,ab. |
| 34 | (active travel* or active transport* or active commut*).ti,ab. |
| 35 | (aerobics or ((fitness or aerobic*) adj5 (therap* or intervention* or program* or scheme* or group* or club? or class* or session*))).ti,ab. |
| 36 | ((reduc* or chang* or modif* or decreas*) adj2 sedentary behavio?r).ti,ab. |
| 37 | (dance adj5 (therap* or intervention* or program* or scheme* or group* or club? or class* or session*)).ti,ab. |
| 38 | personal trainer*.ti,ab. |
| 39 | gym.mp. or gyms.ti,ab. |
| 40 | or/28-39 |
| 41 | cognitive therapy/ or behavior therapy/ or counseling/ |
| 42 | (((behavio* or cognitive) adj2 therap*) or cbt).ti,ab. |
| 43 | ((behavio* or cognitive or therap*) adj2 intervention*).ti,ab. |
| 44 | ((chang* or modif*) adj2 (lifestyle* or behavio?r)).ti,ab. |
| 45 | (counseling or counselling).ti,ab. |
| 46 | motivational interview*.ti,ab. |
| 47 | or/41-46 |
| 48 | Internet/ or social media/ or webcasts as topic/ or podcasts as topic/ |
| 49 | electronic mail/ |
| 50 | therapy, computer-assisted/ |
| 51 | exp cellular phone/ |
| 52 | ((internet or www* or web or computerized or web-based or net or online or podcast* or video*) adj5 (therap* or intervention* or strateg* or program* or management or scheme* or group* or club?)).ti,ab. |
| 53 | (("mobile phone" or "mobile phones" or "cell phone" or "cell phones" or "cellular phones" or "cellular phone" or txt or pxt or sms or mms or app or iphone* or "android phone") adj5 (therap* or intervention* or strateg* or program* or management or scheme* or group* or club?)).ti,ab. |
| 54 | or/48-53 |
| 55 | self care/ |
| 56 | patient education as topic/ |
| 57 | health education/ |
| 58 | ((self help or manual* or information aid* or leaflet* or booklet* or pamphlet* or workbook* or self manag* or self change or self directed* or bibliotherapy) adj5 (therapy or intervention* or strateg* or program* or management or scheme* or group* or club?)).ti,ab. |
| 59 | or/55-58 |
| 60 | 27 or 40 or 47 or 54 or 59 |
| 61 | Weight Loss/ |
| 62 | Weight Gain/ |
| 63 | *Body Mass Index/ |
| 64 | (weight adj1 (los* or reduc* or manag* or gain or regain or increas* or chang*)).ti,ab. |
| 65 | ((bmi or body mass) adj2 (reduc* or decreas* or increas* or chang*)).ti,ab. |
| 66 | or/61-65 |
| 67 | 4 and 60 and 66 |
| 68 | limit 67 to "reviews (maximizes specificity)" |
| 69 | randomized controlled trial.pt. |
| 70 | controlled clinical trial.pt. |
| 71 | randomized.ab. |
| 72 | placebo.ab. |
| 73 | clinical trials as topic.sh. |
| 74 | randomly.ab. |
| 75 | trial.ti. |
| 76 | 69 or 70 or 71 or 72 or 73 or 74 or 75 |
| 77 | exp animals/ not humans.sh. |
| 78 | 76 not 77 |
| 79 | 67 and 78 |
| 80 | (editorial or comment or letter or review).pt. |
| 81 | 79 not 80 |
| 82 | 68 or 81 |
| 83 | limit 82 to ("all infant (birth to 23 months)" or "newborn infant (birth to 1 month)" or "infant (1 to 23 months)" or "preschool child (2 to 5 years)" or "child (6 to 12 years)") |
| 84 | 82 not 83 |

### Table 2. Data inputs for PRIMEtime analysis

| Parameter | Data source |
| --- | --- |
| Population numbers | 2017 values for England and Wales from the Human Mortality Database(26) |
| Population mortality rates | 2017 values for England and Wales from the Human Mortality Database(26) |
| Body mass index | Distribution mean and standard deviation derived by age and sex from the Health Survey for England 2017(27) |
| Height | Mean height derived by age, sex and BMI category from the Health Survey for England 2017(27) |
| Disease incidence, baseline prevalence, and case fatality | Derived by age and sex using the *disbayes* R package (github.com/chjackson/disbayes) and Global Burden of Disease data(28) for England in 2017. |
| Background trends in disease incidence and case fatality | Average annual change estimated by age (0-34, 35-64, 65+) and sex from *disbayes* analyses of incidence and case fatality in 2007 and 2017. In PRIMEtime, trends were applied for 10 years, with disease rates assumed to remain constant thereafter. |
| Relative risks of disease associated with BMI | Relative risks of ischaemic heart disease, ischaemic stroke, type 2 diabetes, colorectal cancer, liver cancer, kidney cancer and cirrhosis from the Prospective Studies Collaboration.(29) Relative risk of post-menopausal breast cancer from Renehan et al.(30) |
| EQ-5D utility weights | Background and disease-specific weights from Sullivan et al.(31) |
| Health and social care costs | Background and disease-specific health and formal care costs derived by Briggs et al.(22) Inflation-adjusted to 2017 using the Health Services Index.(32) |

### Table 3. Included studies reference list

| **Study ID** | **Primary reference** |
| --- | --- |
| Abed 2013 | Abed HS, Wittert GA, Leong DP, et al. Effect of weight reduction and cardiometabolic risk factor management on symptom burden and severity in patients with atrial fibrillation: a randomized clinical trial. *JAMA* 2013; **310**(19): 2050-60. |
| Ackermann 2011 | Ackermann RT, Finch EA, Caffrey HM, Lipscomb ER, Hays LM, Saha C. Long-term effects of a community-based lifestyle intervention to prevent type 2 diabetes: the DEPLOY extension pilot study. *Chronic Illn* 2011; **7**(4): 279-90. |
| Agras 1990 | Agras WS, Taylor CB, Feldman DE, Losch M, Burnett KF. Developing computer-assisted therapy for the treatment of obesity. *Behavior Therapy* 1990; **21**(1): 99-109 |
| Ahern 2017 | Ahern AL, Wheeler GM, Aveyard P, et al. Extended and standard duration weight-loss programme referrals for adults in primary care (WRAP): a randomised controlled trial. *Lancet* 2017; **389**(10085): 2214-25 |
| Almanza -Aguilera 2018 | Almanza-Aguilera E, Brunius C, Bernal-Lopez MR, et al. Impact in Plasma Metabolome as Effect of Lifestyle Intervention for Weight-Loss Reveals Metabolic Benefits in Metabolically Healthy Obese Women. *J Proteome Res* 2018; **17**(8): 2600-10. |
| Anderson 2014 | Anderson AS, Craigie AM, Caswell S, et al. The impact of a bodyweight and physical activity intervention (BeWEL) initiated through a national colorectal cancer screening programme: randomised controlled trial. *BMJ* 2014; **348**: g1823. |
| Appel 2011 | Appel LJ, Clark JM, Yeh HC, et al. Comparative effectiveness of weight-loss interventions in clinical practice. *N Engl J Med* 2011; **365**(21): 1959-68. |
| Ard 2004 | Ard JD, Grambow SC, Liu D, Slentz CA, Kraus WE, Svetkey LP. The effect of the PREMIER interventions on insulin sensitivity. *Diabetes Care* 2004; **27**(2): 340-7. |
| Ard 2018 | Ard JD, Gower B, Hunter G, et al. Effects of Calorie Restriction in Obese Older Adults: The CROSSROADS Randomized Controlled Trial. *J Gerontol A Biol Sci Med Sci* 2017; **73**(1): 73-80 |
| Ash 2006 | Ash S, Reeves M, Bauer J, et al. A randomised control trial comparing lifestyle groups, individual counselling and written information in the management of weight and health outcomes over 12 months.  *Int J Obes (Lond)* 2006; **30**(10): 1557-64. |
| Aveyard 2016 | Aveyard P, Lewis A, Tearne S, et al. Screening and brief intervention for obesity in primary care: a parallel, two-arm, randomised trial. *Lancet* 2016; **388**(10059): 2492-500. |
| Azar 2013 | Azar KM, Xiao L, Ma J. Baseline obesity status modifies effectiveness of adapted diabetes prevention program lifestyle interventions for weight management in primary care. *Biomed Res Int* 2013; **2013**: 191209. |
| Bacon 2002 | Bacon L, Keim NL, Van Loan MD, et al. Evaluating a 'non-diet' wellness intervention for improvement of metabolic fitness, psychological well-being and eating and activity behaviors. *Int J Obes Relat Metab Disord* 2002; **26**(6): 854-65. |
| Barnes 2017 | arnes RD, Ivezaj V, Martino S, Pittman BP, Grilo CM. Back to Basics? No Weight Loss from Motivational Interviewing Compared to Nutrition Psychoeducation at One-Year Follow-Up. *Obesity (Silver Spring)* 2017; **25**(12): 2074-8. |
| Bartels 2015 | Bartels SJ, Pratt SI, Aschbrenner KA, et al. Pragmatic replication trial of health promotion coaching for obesity in serious mental illness and maintenance of outcomes. *Am J Psychiatry* 2015; **172**(4): 344-52. |
| Beavers 2017 | Beavers KM, Ambrosius WT, Rejeski WJ, et al. Effect of Exercise Type During Intentional Weight Loss on Body Composition in Older Adults with Obesity. *Obesity (Silver Spring)* 2017; **25**(11): 1823-9. |
| Bennett 2012 | Bennett GG, Warner ET, Glasgow RE, et al. Obesity treatment for socioeconomically disadvantaged patients in primary care practice. *Arch Intern Med* 2012; **172**(7): 565-74. |
| Bennett 2013 | Bennett GG, Foley P, Levine E, et al. Behavioral treatment for weight gain prevention among black women in primary care practice: a randomized clinical trial. *JAMA Intern Med* 2013; **173**(19): 1770-7. |
| Berry 2014 | Berry DC, Schwartz TA, McMurray RG, et al. The family partners for health study: a cluster randomized controlled trial for child and parent weight management. *Nutr Diabetes* 2014; **4**(1): e101. |
| Bertram 1990 | Bertram SR, Venter I, Stewart RI. Weight loss in obese women--exercise v. dietary education. *S Afr Med J* 1990; **78**(1): 15-8. |
| Bertz 2012 | Bertz F, Brekke HK, Ellegård L, Rasmussen KM, Wennergren M, Winkvist A. Diet and exercise weight-loss trial in lactating overweight and obese women. *Am J Clin Nutr 2012;* **96**(4): 698-705. |
| Bo 2007 | Bo S, Ciccone G, Baldi C, et al. Effectiveness of a lifestyle intervention on metabolic syndrome. A randomized controlled trial. *J Gen Intern Med* 2007; **22**(12): 1695-703. |
| Brown 2014 | Brown C, Goetz J, Hamera E, Gajewski B. Treatment response to the RENEW weight loss intervention in schizophrenia: impact of intervention setting. *Schizophr Res* 2014; **159**(2-3): 421-5. |
| Burke 2005 | Burke V, Beilin LJ, Cutt HE, Mansour J, Wilson A, Mori TA. Effects of a lifestyle programme on ambulatory blood pressure and drug dosage in treated hypertensive patients: a randomized controlled trial. *J Hypertens* 2005; **23**(6): 1241-9. |
| Burke 2015 | Burke LE, Ewing LJ, Ye L, et al. The SELF trial: A self-efficacy-based behavioral intervention trial for weight loss maintenance. *Obesity (Silver Spring)* 2015; **23**(11): 2175-82. |
| Cheyette 2007 | Cheyette C. Weight No More: a randomised controlled trial for people with type 2 diabetes on insulin therapy. *Pract Diab Int* 2007; **24**(9): 450-6. |
| Christensen 2012 | Christensen JR, Overgaard K, Carneiro IG, Holtermann A, Søgaard K. Weight loss among female health care workers--a 1-year workplace based randomized controlled trial in the FINALE-health study. *BMC Public Health* 2012; **12**: 625. |
| Cleo 2018 | Cleo G, Glasziou P, Beller E, Isenring E, Thomas R. Habit-based interventions for weight loss maintenance in adults with overweight and obesity: a randomized controlled trial. *Int J Obes (Lond)* 2019; **43**(2): 374-83. |
| Conroy 2015 | Conroy MB, Sward KL, Spadaro KC, et al. Effectiveness of a physical activity and weight loss intervention for middle-aged women: healthy bodies, healthy hearts randomized trial. *J Gen Intern Med* 2015; **30**(2): 207-13. |
| Cooper 2010 | Cooper Z, Doll HA, Hawker DM, et al. Testing a new cognitive behavioural treatment for obesity: A randomized controlled trial with three-year follow-up. *Behav Res Ther* 2010; **48**(8): 706-13. |
| Cousins 1992 | Cousins JH, Rubovits DS, Dunn JK, Reeves RS, Ramirez AG, Foreyt JP. Family versus individually oriented intervention for weight loss in Mexican American women. *Public Health Rep* 1992; **107**(5): 549-55. |
| Craighead 1989 | Craighead LW, Blum MD. Supervised exercise in behavioral treatment for moderate obesity. *Behavior Therapy* 1989; **20**(1): 49-59. |
| Dale 2009 | Dale KS, Mann JI, McAuley KA, Williams SM, Farmer VL. Sustainability of lifestyle changes following an intensive lifestyle intervention in insulin resistant adults: Follow-up at 2-years. *Asia Pac J Clin Nutr* 2009; **18**(1): 114-20. |
| Dalziel 2006 | Dalziel K, Segal L, de Lorgeril M. A mediterranean diet is cost-effective in patients with previous myocardial infarction. *J Nutr* 2006; **136**(7): 1879-85. |
| Damschroder 2014 | Damschroder LJ, Lutes LD, Kirsh S, et al. Small-changes obesity treatment among veterans: 12-month outcomes. *Am J Prev Med* 2014; **47**(5): 541-53. |
| Daumit 2013 | Daumit GL, Dickerson FB, Wang NY, et al. A behavioral weight-loss intervention in persons with serious mental illness. *N Engl J Med* 2013; **368**(17): 1594-602. |
| deVos 2016 | de Vos BC, Runhaar J, van Middelkoop M, Krul M, Bierma-Zeinstra SM. Long-term effects of a randomized, controlled, tailor-made weight-loss intervention in primary care on the health and lifestyle of overweight and obese women. *Am J Clin Nutr* 2016; **104**(1): 33-40. |
| Diabetes Prevention Program R G 2009 | Knowler WC, Fowler SE, Hamman RF, et al. 10-year follow-up of diabetes incidence and weight loss in the Diabetes Prevention Program Outcomes Study. *Lancet* 2009; **374**(9702): 1677-86. |
| Djuric 2002 | Djuric Z, DiLaura NM, Jenkins I, et al. Combining weight-loss counseling with the weight watchers plan for obese breast cancer survivors. *Obes Res* 2002; **10**(7): 657-65. |
| Duncan 2016 | Duncan S, Goodyear-Smith F, McPhee J, Zinn C, Grøntved A, Schofield G. Family-centered brief intervention for reducing obesity and cardiovascular disease risk: A randomized controlled trial. *Obesity (Silver Spring)* 2016; **24**(11): 2311-8. |
| Eakin 2014 | Eakin EG, Winkler EA, Dunstan DW, et al. Living well with diabetes: 24-month outcomes from a randomized trial of telephone-delivered weight loss and physical activity intervention to improve glycemic control. *Diabetes care* 2014; **37**(8): 2177-85. |
| Eaton 2016 | Eaton CB, Hartman SJ, Perzanowski E, et al. A Randomized Clinical Trial of a Tailored Lifestyle Intervention for Obese, Sedentary, Primary Care Patients. *Ann Fam Med* 2016; **14**(4): 311-9. |
| Fernandez-Ruiz 2018 | Fernández-Ruiz VE, Armero-Barranco D, Paniagua-Urbano JA, Sole-Agusti M, Ruiz-Sánchez A, Gómez-Marín J. Short-medium-long-term efficacy of interdisciplinary intervention against overweight and obesity: Randomized controlled clinical trial. *Int J Nurs Prac* 2018; **24**(6): e12690. |
| Foley 2016 | Foley P, Steinberg D, Levine E, et al. Track: A randomized controlled trial of a digital health obesity treatment intervention for medically vulnerable primary care patients. *Contemp Clin Trials* 2016; **48**: 12-20. |
| Foster-Schubert 2012 | Foster-Schubert KE, Alfano CM, Duggan CR, et al. Effect of diet and exercise, alone or combined, on weight and body composition in overweight-to-obese postmenopausal women. *Obesity (Silver Spring)* 2012; **20**(8): 1628-38. |
| Fuller 2012 | Fuller NR, Lau NS, Denyer G, Caterson ID. A 12-month, randomised, controlled trial to examine the efficacy of the Korean diet in an Australian overweight and obese population - A follow up analysis. *Obes Res Clin Pract* 2012; **6**(4): e263-346. |
| Goodwin 2014 | Goodwin PJ, Segal RJ, Vallis M, et al. Randomized trial of a telephone-based weight loss intervention in postmenopausal women with breast cancer receiving letrozole: the LISA trial. *J Clin Oncol* 2014; **32**(21): 2231-9. |
| Green 2015 | Green CA, Yarborough BJ, Leo MC, et al. Weight maintenance following the STRIDE lifestyle intervention for individuals taking antipsychotic medications. *Obesity (Silver Spring)* 2015; **23**(10): 1995-2001. |
| Grilo 2011 | Grilo CM, Masheb RM, Wilson GT, Gueorguieva R, White MA. Cognitive-behavioral therapy, behavioral weight loss, and sequential treatment for obese patients with binge-eating disorder: a randomized controlled trial. *J Consult Clin Psychol* 2011; **79**(5): 675-85. |
| Grilo 2014 | Grilo CM, Masheb RM, White MA, et al. Treatment of binge eating disorder in racially and ethnically diverse obese patients in primary care: randomized placebo-controlled clinical trial of self-help and medication. *Behav Res Ther* 2014; **58**: 1-9. |
| Hanson 1976 | Hanson RW, Borden BL, Hall SM, Hall RG. Use of programmed instruction in teaching self-management skills to overweight adults. *Behavior Therapy* 1976; **7**(3): 366-73. |
| Hardcastle 2013 | Hardcastle SJ, Taylor AH, Bailey MP, Harley RA, Hagger MS. Effectiveness of a motivational interviewing intervention on weight loss, physical activity and cardiovascular disease risk factors: a randomised controlled trial with a 12-month post-intervention follow-up*. Int J Behav Nutr Phys Act* 2013; **10**: 40. |
| Harrigan 2016 | Harrigan M, Cartmel B, Loftfield E, et al. Randomized Trial Comparing Telephone Versus In-Person Weight Loss Counseling on Body Composition and Circulating Biomarkers in Women Treated for Breast Cancer: The Lifestyle, Exercise, and Nutrition (LEAN) Study. *J Clin Oncol* 2016; **34**(7): 669-76. |
| Harris 2017 | Harris L, Hankey C, Jones N, et al. A cluster randomised control trial of a multi-component weight management programme for adults with intellectual disabilities and obesity. *Br J Nutr* 2017; **118**(3): 229-40. |
| Hunt 2014 | Hunt K, Wyke S, Gray CM, et al. A gender-sensitised weight loss and healthy living programme for overweight and obese men delivered by Scottish Premier League football clubs (FFIT): a pragmatic randomised controlled trial. *Lancet* 2014; **383**(9924): 1211-21. |
| Huseinovic 2016 | Huseinovic E, Bertz F, Leu Agelii M, Hellebö Johansson E, Winkvist A, Brekke HK. Effectiveness of a weight loss intervention in postpartum women: results from a randomized controlled trial in primary health care. *Am J Clin Nutr* 2016; **104**(2): 362-70. |
| Irwin 2003 | Irwin ML, Yasui Y, Ulrich CM, et al. Effect of exercise on total and intra-abdominal body fat in postmenopausal women: a randomized controlled trial. *JAMA* 2003; **289**(3): 323-30. |
| Jackson 1982 | Jackson HJ, Thorbecke PJ. Treating obesity of mentally retarded adolescents and adults: an exploratory program. *Am Jj Ment Defic* 1982; **87**(3): 302-8. |
| Jakicic 2011 | Jakicic JM, Otto AD, Lang W, et al. The effect of physical activity on 18-month weight change in overweight adults. *Obesity (Silver Spring)* 2011; **19**(1): 100-9. |
| Jebb 2011 | Jebb SA, Ahern AL, Olson AD, et al. Primary care referral to a commercial provider for weight loss treatment versus standard care: a randomised controlled trial. *Lancet* 2011; **378**(9801): 1485-92. |
| Jebb 2017 | Jebb SA, Astbury NM, Tearne S, Nickless A, Aveyard P. Doctor Referral of Overweight People to a Low-Energy Treatment (DROPLET) in primary care using total diet replacement products: a protocol for a randomised controlled trial. *BMJ open* 2017; **7**(8): e016709. |
| Jeffery 1995 | Jeffery RW, Wing RR. Long-term effects of interventions for weight loss using food provision and monetary incentives. *J Consult Clin Psychol* 1995; **63**(5): 793-6. |
| Jenkins 2017 | Jenkins DJA, Boucher BA, Ashbury FD, et al. Effect of Current Dietary Recommendations on Weight Loss and Cardiovascular Risk Factors. *J Am Coll Cardiol* 2017; **69**(9): 1103-12. |
| John 2011 | John LK, Loewenstein G, Troxel AB, Norton L, Fassbender JE, Volpp KG. Financial incentives for extended weight loss: a randomized, controlled trial. *J Gen Intern Med* 2011; **26**(6): 621-6. |
| Jolly 2011 | Jolly K, Lewis A, Beach J, et al. Comparison of range of commercial or primary care led weight reduction programmes with minimal intervention control for weight loss in obesity: lighten Up randomised controlled trial. *BMJ* 2011; **343**: d6500. |
| Jones 1986 | Jones SE, Owens HM, Bennett GA. Does behaviour therapy work for dietitians? An experimental evaluation of the effects of three procedures in a weight reduction clinic. *Hum Nutr Appl Nutr* 1986; **40**(4): 272-81. |
| Jones 1999 | Jones DW, Miller ME, Wofford MR, et al. The effect of weight loss intervention on antihypertensive medication requirements in the hypertension Optimal Treatment (HOT) study. *Am J Hypertens* 1999; **12**(12 Pt 1-2): 1175-80. |
| Katzer 2008 | Katzer L, Bradshaw AJ, Horwath CC, Gray AR, O'Brien S, Joyce J. Evaluation of a "nondieting" stress reduction program for overweight women: a randomized trial. *Am J Health Promot* 2008; **22**(4): 264-74. |
| Kuller 2012 | Kuller LH, Pettee Gabriel KK, Kinzel LS, et al. The Women on the Move Through Activity and Nutrition (WOMAN) study: final 48-month results. *Obesity (Silver Spring)* 2012; **20**(3): 636-43. |
| Leahey 2014 | Leahey TM, Thomas G, Fava JL, et al. Adding evidence-based behavioral weight loss strategies to a statewide wellness campaign: a randomized clinical trial. *Am J Public Health* 2014; **104**(7): 1300-6. |
| Ley 2004 | Ley SJ, Metcalf PA, Scragg RK, Swinburn BA. Long-term effects of a reduced fat diet intervention on cardiovascular disease risk factors in individuals with glucose intolerance. *Diabetes Res Clin Pract* 2004; **63**(2): 103-12. |
| Li 2016 | Li X, Cai X, Ma X, et al. Short- and Long-Term Effects of Wholegrain Oat Intake on Weight Management and Glucolipid Metabolism in Overweight Type-2 Diabetics: A Randomized Control Trial. *Nutrients* 2016; **8**(9). |
| Lindstrom 2003 | Lindström J, Louheranta A, Mannelin M, et al. The Finnish Diabetes Prevention Study (DPS): Lifestyle intervention and 3-year results on diet and physical activity. *Diabetes care* 2003; **26**(12): 3230-6. |
| Liss 2016 | Liss DT, Finch EA, Gregory DL, Cooper A, Ackermann RT. Design and participant characteristics for a randomized effectiveness trial of an intensive lifestyle intervention to reduce cardiovascular risk in adults with type 2 diabetes: The I-D-HEALTH study. *Contemp Clin Trials* 2016; **46**: 114-21. |
| Little 2016 | Little P, Stuart B, Hobbs FR, et al. An internet-based intervention with brief nurse support to manage obesity in primary care (POWeR+): a pragmatic, parallel-group, randomised controlled trial. *Lancet Diabetes Endocrinol* 2016; **4**(10): 821-8. |
| Ma 2015 | Ma J, Strub P, Xiao L, et al. Behavioral weight loss and physical activity intervention in obese adults with asthma. A randomized trial. *Ann Am Thorac Soc* 2015; **12**(1): 1-11. |
| Manzoni 2016 | Manzoni GM, Cesa GL, Bacchetta M, et al. Virtual Reality-Enhanced Cognitive-Behavioral Therapy for Morbid Obesity: A Randomized Controlled Study with 1 Year Follow-Up. *Cyberpsychol Behav Soc Netw* 2016; **19**(2): 134-40. |
| Marniemi 1990 | Marniemi J, Seppänen A, Hakala P. Long-term effects on lipid metabolism of weight reduction on lactovegetarian and mixed diet. *Inter J Obes* 1990; **14**(2): 113-25. |
| Martin 2008 | Martin PD, Dutton GR, Rhode PC, Horswell RL, Ryan DH, Brantley PJ. Weight loss maintenance following a primary care intervention for low-income minority women. *Obesity (Silver Spring)* 2008; **16**(11): 2462-7. |
| Mefferd 2007 | Mefferd K, Nichols JF, Pakiz B, Rock CL. A cognitive behavioral therapy intervention to promote weight loss improves body composition and blood lipid profiles among overweight breast cancer survivors. *Breast Cancer Res Treat* 2007; **104**(2): 145-52. |
| Melchart 2017 | Melchart D, Löw P, Wühr E, Kehl V, Weidenhammer W. Effects of a tailored lifestyle self-management intervention (TALENT) study on weight reduction: a randomized controlled trial. *Diabetes Metab Syndr Obes* 2017; **10**: 235-45. |
| Melin 2003 | Melin I, Karlström B, Lappalainen R, Berglund L, Mohsen R, Vessby B. A programme of behaviour modification and nutrition counselling in the treatment of obesity: a randomised 2-y clinical trial. *Int J Obes Relat Metab Disord*  2003; **27**(9): 1127-35. |
| Menard 2005 | Ménard J, Payette H, Baillargeon JP, et al. Efficacy of intensive multitherapy for patients with type 2 diabetes mellitus: a randomized controlled trial. *CMAJ* 2005; **173**(12): 1457-66. |
| Mensinger 2016 | Mensinger JL, Calogero RM, Stranges S, Tylka TL. A weight-neutral versus weight-loss approach for health promotion in women with high BMI: A randomized-controlled trial. *Appetite* 2016; **105**: 364-74. |
| Messier 2013 | Messier SP, Mihalko SL, Legault C, et al. Effects of intensive diet and exercise on knee joint loads, inflammation, and clinical outcomes among overweight and obese adults with knee osteoarthritis: the IDEA randomized clinical trial. *JAMA* 2013; **310**(12): 1263-73. |
| Miller 2002 | Miller ER, 3rd, Erlinger TP, Young DR, et al. Results of the Diet, Exercise, and Weight Loss Intervention Trial (DEW-IT). *Hypertension* 2002; **40**(5): 612-8. |
| Mitsui 2008 | Mitsui T, Shimaoka K, Tsuzuku S, Kajioka T, Sakakibara H. Gentle exercise of 40 minutes with dietary counseling is effective in treating metabolic syndrome. *Tohoku J Exp Med* 2008; **215**(4): 355-61 |
| Morgan 2010 | Morgan PJ, Lubans DR, Collins CE, Warren JM, Callister R. 12-month outcomes and process evaluation of the SHED-IT RCT: an internet-based weight loss program targeting men. *Obesity (Silver Spring)* 2011; **19**(1): 142-51. |
| Muggia 2014 | Muggia C, Falchi AG, Michelini I, et al. Brief group cognitive behavioral treatment in addition to prescriptive diet versus standard care in obese and overweight patients. A randomized controlled trial. *e-SPEN* 2014; **9**(1): e26-e33. |
| Munsch 2003 | Munsch S, Biedert E, Keller U. Evaluation of a lifestyle change programme for the treatment of obesity in general practice. *Swiss Med Wkly* 2003; **133**(9-10): 148-54. |
| Nakata 2014 | Nakata Y, Okada M, Hashimoto K, Harada Y, Sone H, Tanaka K. Weight loss maintenance for 2 years after a 6-month randomised controlled trial comparing education-only and group-based support in Japanese adults. *Obes Facts* 2014; **7**(6): 376-87 |
| Nanchahal 2012 | Nanchahal K, Power T, Holdsworth E, et al. A pragmatic randomised controlled trial in primary care of the Camden Weight Loss (CAMWEL) programme. *BMJ open* 2012; **2**(3). |
| Ng 2015 | Ng SSS, Chan RSM, Woo J, et al. A Randomized Controlled Study to Examine the Effect of a Lifestyle Modification Program in OSA. *Chest* 2015; **148**(5): 1193-203. |
| Nicklas 2004 | Nicklas BJ, Ambrosius W, Messier SP, et al. Diet-induced weight loss, exercise, and chronic inflammation in older, obese adults: a randomized controlled clinical trial. *Am J Clin Nutr* 2004; **79**(4): 544-51. |
| Nilsen 2011 | Nilsen V, Bakke PS, Gallefoss F. Effects of lifestyle intervention in persons at risk for type 2 diabetes mellitus - results from a randomised, controlled trial. *BMC public health* 2011; **11**: 893. |
| Nordby 2012 | Nordby P, Auerbach PL, Rosenkilde M, et al. Endurance training per se increases metabolic health in young, moderately overweight men. *Obesity (Silver Spring)* 2012; **20**(11): 2202-12. |
| Nurkkala 2015 | Nurkkala M, Kaikkonen K, Vanhala ML, Karhunen L, Keränen AM, Korpelainen R. Lifestyle intervention has a beneficial effect on eating behavior and long-term weight loss in obese adults. *Eat Behav* 2015; **18**: 179-85. |
| Oldroyd 2006 | Oldroyd JC, Unwin NC, White M, Mathers JC, Alberti KG. Randomised controlled trial evaluating lifestyle interventions in people with impaired glucose tolerance. *Diabetes Res Clin Pract* 2006; **72**(2): 117-27. |
| Pan 1997 | Pan XR, Li GW, Hu YH, et al. Effects of diet and exercise in preventing NIDDM in people with impaired glucose tolerance. The Da Qing IGT and Diabetes Study. *Diabetes Care* 1997; **20**(4): 537-44. |
| Parikh 2010 | Parikh P, Simon EP, Fei K, Looker H, Goytia C, Horowitz CR. Results of a pilot diabetes prevention intervention in East Harlem, New York City: Project HEED. *Am J Public Health* 2010; **100 Suppl 1**(Suppl 1): S232-9. |
| Patel 2016 | Patel MS, Asch DA, Troxel AB, et al. Premium-Based Financial Incentives Did Not Promote Workplace Weight Loss In A 2013-15 Study. *Health Aff (Millwood)* 2016; **35**(1): 71-9. |
| Pekkarinen 2015 | Pekkarinen T, Kaukua J, Mustajoki P. Long-term weight maintenance after a 17-week weight loss intervention with or without a one-year maintenance program: a randomized controlled trial. *J Obes* 2015; **2015**: 651460. |
| Perri 1984 | Perri MG, Shapiro RM, Ludwig WW, Twentyman CT, McAdoo WG. Maintenance strategies for the treatment of obesity: an evaluation of relapse prevention training and posttreatment contact by mail and telephone. *J Consult Clin Psychol* 1984; **52**(3): 404-13. |
| Perri 2001 | Perri MG, Nezu AM, McKelvey WF, Shermer RL, Renjilian DA, Viegener BJ. Relapse prevention training and problem-solving therapy in the long-term management of obesity. *J Consult Clin Psychol* 2001; **69**(4): 722-6. |
| Perri 2014 | Perri MG, Limacher MC, von Castel-Roberts K, et al. Comparative effectiveness of three doses of weight-loss counseling: two-year findings from the rural LITE trial. *Obesity (Silver Spring)* 2014; **22**(11): 2293-300. |
| Pettman 2009 | Pettman TL, Buckley JD, Misan GM, Coates AM, Howe PR. Health benefits of a 4-month group-based diet and lifestyle modification program for individuals with metabolic syndrome. *Obes Res Clin Pract* 2009; **3**(4): 221-35. |
| Poelman 2015 | Poelman MP, de Vet E, Velema E, de Boer MR, Seidell JC, Steenhuis IH. PortionControl@HOME: results of a randomized controlled trial evaluating the effect of a multi-component portion size intervention on portion control behavior and body mass index Ann Behav Med 2015; **49**(1): 18-28. |
| Promrat 2010 | Promrat K, Kleiner DE, Niemeier HM, et al. Randomized controlled trial testing the effects of weight loss on nonalcoholic steatohepatitis. *Hepatology* 2010; **51**(1): 121-9. |
| Provencher 2009 | Provencher V, Bégin C, Tremblay A, et al. Health-At-Every-Size and eating behaviors: 1-year follow-up results of a size acceptance intervention. *J Am Diet Assoc* 2009; **109**(11): 1854-61. |
| Rejeski 2011 | Rejeski WJ, Brubaker PH, Goff DC, Jr., et al. Translating weight loss and physical activity programs into the community to preserve mobility in older, obese adults in poor cardiovascular health. *Arch Internal Med* 2011; **171**(10): 880-6. |
| Ridgeway 1999 | Ridgeway NA, Harvill DR, Harvill LM, Falin TM, Forester GM, Gose OD. Improved control of type 2 diabetes mellitus: a practical education/behavior modification program in a primary care clinic. *South Med J* 1999; **92**(7): 667-72. |
| Rolls 2005 | Rolls BJ, Roe LS, Beach AM, Kris-Etherton PM. Provision of foods differing in energy density affects long-term weight loss. *Obes Res* 2005; **13**(6): 1052-60. |
| Rosas 2015 | Rosas LG, Thiyagarajan S, Goldstein BA, et al. The effectiveness of two community-based weight loss strategies among obese, low-income US Latinos. *J Acad Nutr Diet* 2015; **115**(4): 537-50.e2. |
| Ross 2012 | Ross R, Lam M, Blair SN, et al. Trial of prevention and reduction of obesity through active living in clinical settings: a randomized controlled trial. *Arch Intern Med* 2012; **172**(5): 414-24. |
| Samaras 1997 | Samaras K, Ashwell S, Mackintosh AM, Fleury AC, Campbell LV, Chisholm DJ. Will older sedentary people with non-insulin-dependent diabetes mellitus start exercising? A health promotion model. *Diabetes Res Clin Pract* 1997; **37**(2): 121-8. |
| Santanasto 2011 | Santanasto AJ, Glynn NW, Newman MA, et al. Impact of weight loss on physical function with changes in strength, muscle mass, and muscle fat infiltration in overweight to moderately obese older adults: a randomized clinical trial. *J Obes* 2011; **2011**. |
| Sattin 2016 | Sattin RW, Williams LB, Dias J, et al. Community Trial of a Faith-Based Lifestyle Intervention to Prevent Diabetes Among African-Americans. *J Community Health* 2016; **41**(1): 87-96. |
| Schubel 2016 | Schübel R, Graf ME, Nattenmüller J, et al. The effects of intermittent calorie restriction on metabolic health: Rationale and study design of the HELENA Trial. *Contemp Clin Trials* 2016; **51**: 28-33. |
| Seligman 2011 | Seligman BG, Polanczyk CA, Santos AS, et al. Intensive practical lifestyle intervention improves endothelial function in metabolic syndrome independent of weight loss: a randomized controlled trial. *Metabolism* 2011; **60**(12): 1736-40. |
| Snel 2012 | Snel M, Sleddering MA, Vd Peijl ID, et al. Quality of life in type 2 diabetes mellitus after a very low calorie diet and exercise. *Eur J Intern Med* 2012; **23**(2): 143-9. |
| Solbrig 2019 | Solbrig L, Whalley B, Kavanagh DJ, et al. Functional imagery training versus motivational interviewing for weight loss: a randomised controlled trial of brief individual interventions for overweight and obesity. *Int J Obes (Lond)* 2019; **43**(4): 883-94. |
| Somers 2012 | Somers TJ, Blumenthal JA, Guilak F, et al. Pain coping skills training and lifestyle behavioral weight management in patients with knee osteoarthritis: a randomized controlled study. *Pain* 2012; **153**(6): 1199-209. |
| Spring 2017 | Spring B, Pellegrini CA, Pfammatter A, et al. Effects of an abbreviated obesity intervention supported by mobile technology: The ENGAGED randomized clinical trial. *Obesity (Silver Spring)* 2017; **25**(7): 1191-8. |
| Stahre 2005 | Stahre L, Hällström T. A short-term cognitive group treatment program gives substantial weight reduction up to 18 months from the end of treatment. A randomized controlled trial. *Eat Weight Disord* 2005; **10**(1): 51-8. |
| Stahre 2007 | Stahre L, Tärnell B, Håkanson CE, Hällström T. A randomized controlled trial of two weight-reducing short-term group treatment programs for obesity with an 18-month follow-up. *Int J Behav Med* 2007; **14**(1): 48-55 |
| Stenius-Aarniala 2000 | Stenius-Aarniala B, Poussa T, Kvarnström J, Grönlund EL, Ylikahri M, Mustajoki P. Immediate and long term effects of weight reduction in obese people with asthma: randomised controlled study. *BMJ* 2000; **320**(7238): 827-32. |
| Stevens 1993 | Stevens VJ, Corrigan SA, Obarzanek E, et al. Weight loss intervention in phase 1 of the Trials of Hypertension Prevention. The TOHP Collaborative Research Group. *Arch Intern Med* 1993; **153**(7): 849-58. |
| Stevens 2001 | Stevens VJ, Obarzanek E, Cook NR, et al. Long-term weight loss and changes in blood pressure: results of the Trials of Hypertension Prevention, phase II. *Ann Intern Med* 2001; **134**(1): 1-11. |
| Strobl 2013 | Ströbl V, Knisel W, Landgraf U, Faller H. A combined planning and telephone aftercare intervention for obese patients: effects on physical activity and body weight after one year. *J Rehabil Med* 2013; **45**(2): 198-205. |
| Tapsell 2017 | Tapsell LC, Lonergan M, Batterham MJ, et al. Effect of interdisciplinary care on weight loss: a randomised controlled trial. *BMJ open* 2017; **7**(7): e014533. |
| TarragaMarcos 2017 | Tárraga Marcos ML, Panisello Royo JM, Carbayo Herencia JA, Rosich Domenech N, Alins Presas J, Tárraga López PJ. Effect on the lipid parameters of an intervention to reduce weight in overweight and obese patients. *Clin Investig Arterioscler* 2017; **29**(3): 103-10. |
| Teeriniemi 2018 | Teeriniemi AM, Salonurmi T, Jokelainen T, et al. A randomized clinical trial of the effectiveness of a Web-based health behaviour change support system and group lifestyle counselling on body weight loss in overweight and obese subjects: 2-year outcomes. *J Intern Med* 2018; **284**(5): 534-45. |
| ter Bogt 2009 | ter Bogt NC, Bemelmans WJ, Beltman FW, Broer J, Smit AJ, van der Meer K. Preventing weight gain: one-year results of a randomized lifestyle intervention. *Am J Prev Med* 2009; **37**(4): 270-7. |
| The Look AHEAD Research Group 2010 | Wing RR. Long-term effects of a lifestyle intervention on weight and cardiovascular risk factors in individuals with type 2 diabetes mellitus: four-year results of the Look AHEAD trial. *Arch Intern Med* 2010; **170**(17): 1566-75. |
| Trepanowski 2017 | Trepanowski JF, Kroeger CM, Barnosky A, et al. Effect of Alternate-Day Fasting on Weight Loss, Weight Maintenance, and Cardioprotection Among Metabolically Healthy Obese Adults: A Randomized Clinical Trial. *JAMA Intern Med* 2017; **177**(7): 930-8. |
| Tsai 2010 | Tsai AG, Wadden TA, Rogers MA, Day SC, Moore RH, Islam BJ. A primary care intervention for weight loss: results of a randomized controlled pilot study. *Obesity (Silver Spring)* 2010; **18**(8): 1614-8. |
| Tuomilehto 2009 | Tuomilehto HP, Seppä JM, Partinen MM, et al. Lifestyle intervention with weight reduction: first-line treatment in mild obstructive sleep apnea. *Am J Respir Crit Care Med* 2009; **179**(4): 320-7. |
| van de Glind 2017 | van de Glind I, Bunn C, Gray CM, et al. The intervention process in the European Fans in Training (EuroFIT) trial: a mixed method protocol for evaluation. *Trials* 2017; **18**(1): 356. |
| vanWier 2011 | van Wier MF, Dekkers JC, Hendriksen IJ, et al. Effectiveness of phone and e-mail lifestyle counseling for long term weight control among overweight employees. *J Occup Environ Med* 2011; **53**(6): 680-6. |
| Vissers 2010 | Vissers D, Verrijken A, Mertens I, et al. Effect of long-term whole body vibration training on visceral adipose tissue: a preliminary report. *Obes Facts* 2010; **3**(2): 93-100. |
| von Gruenigen 2012 | von Gruenigen V, Frasure H, Kavanagh MB, et al. Survivors of uterine cancer empowered by exercise and healthy diet (SUCCEED): a randomized controlled trial. *Gynecologic oncology* 2012; **125**(3): 699-704. |
| vonGruenigen 2008 | von Gruenigen VE, Courneya KS, Gibbons HE, Kavanagh MB, Waggoner SE, Lerner E. Feasibility and effectiveness of a lifestyle intervention program in obese endometrial cancer patients: a randomized trial. *Gynecol Oncol* 2008; **109**(1): 19-26. |
| West 2007 | West DS, DiLillo V, Bursac Z, Gore SA, Greene PG. Motivational interviewing improves weight loss in women with type 2 diabetes. *Diabetes Care* 2007; **30**(5): 1081-7. |
| West 2011 | West DS, Bursac Z, Cornell CE, et al. Lay health educators translate a weight-loss intervention in senior centers: a randomized controlled trial. *Am J Prev Med* 2011; **41**(4): 385-91. |
| Whelton 1998 | Whelton PK, Appel LJ, Espeland MA, et al. Sodium reduction and weight loss in the treatment of hypertension in older persons: a randomized controlled trial of nonpharmacologic interventions in the elderly (TONE). TONE Collaborative Research Group. *JAMA* 1998; **279**(11): 839-46. |
| Wilson 2016 | Wilson MG, DeJoy DM, Vandenberg RJ, Corso P, Padilla H, Zuercher H. Effect of Intensity and Program Delivery on the Translation of Diabetes Prevention Program to Worksites: A Randomized Controlled Trial of Fuel Your Life. *J Occup Environ Med* 2016; **58**(11): 1113-20. |
| Wilson 2016b | Wilson MG, DeJoy DM, Vandenberg R, Padilla H, Davis M. FUEL Your Life: A Translation of the Diabetes Prevention Program to Worksites. *Am J Health Promot* 2016; **30**(3): 188-97. |
| Wing 1998 | Wing RR, Venditti E, Jakicic JM, Polley BA, Lang W. Lifestyle intervention in overweight individuals with a family history of diabetes. *Diabetes Care* 1998; **21**(3): 350-9. |
| Yannakoulia 2008 | Yannakoulia M, Poulia KA, Mylona E, Kontogianni MD. Effectiveness of an intensive nutritional intervention in patients with type 2 diabetes mellitus: results from a pilot study. *Rev Diabet Stud* 2007; **4**(4): 226-30. |
| Yardley 2014 | Yardley L, Ware LJ, Smith ER, et al. Randomised controlled feasibility trial of a web-based weight management intervention with nurse support for obese patients in primary care. *Int J Behav Nutr Phys Act* 2014; **11**: 67. |
| Yates 2009 | Yates T, Davies M, Gorely T, Bull F, Khunti K. Effectiveness of a pragmatic education program designed to promote walking activity in individuals with impaired glucose tolerance: a randomized controlled trial. *Diabetes Care* 2009; **32**(8): 1404-10. |
| Yates 2018 | Yates MS, Coletta AM, Zhang Q, et al. Prospective Randomized Biomarker Study of Metformin and Lifestyle Intervention for Prevention in Obese Women at Increased Risk for Endometrial Cancer. *Cancer Prev Res (Phila)* 2018; **11**(8): 477-90 |
| Yeh 2016 | Yeh MC, Heo M, Suchday S, et al. Translation of the Diabetes Prevention Program for diabetes risk reduction in Chinese immigrants in New York City. *Diabet Med* 2016; **33**(4): 547-51. |
| Yin 2018 | Yin Z, Perry J, Duan X, et al. Cultural adaptation of an evidence-based lifestyle intervention for diabetes prevention in Chinese women at risk for diabetes: results of a randomized trial. *Int Health* 2018; **10**(5): 391-400. |
| Zhang 2016 | Zhang HJ, He J, Pan LL, et al. Effects of Moderate and Vigorous Exercise on Nonalcoholic Fatty Liver Disease: A Randomized Clinical Trial. *JAMA Internal Med* 2016; **176**(8): 1074-82. |

#

### Table 4. Risk of bias of included studies

| **Study ID** | | **Random sequence generation  (Selection bias)** | **Allocation concealment**  **(Selection bias)** | **Blinding of outcome assessment**  **(Detection bias)** | **Incomplete outcome data**  **(Attrition bias)** | **Other bias** |
| --- | --- | --- | --- | --- | --- | --- |
| **1** | **Abed 2013** | UNCLEAR | UNCLEAR | LOW | LOW | HIGH |
|  | Assessment justification: | Single-centre, partially blinded RCT. | No explicit information regarding allocation concealment provided:  Study coordinators, treating physicians, and other personnel, with the exception of weight loss counselors, were blinded to randomization. Patients were instructed not to disclose their status. Patient records contained generic statements without indicating group allocation. | Weight objectively measured. | At 12 months, 109 (73%) had completed the study (57 in the intervention group and 52 in the control group). By 15 months, 81 (54%) remained (42 in the intervention group and 39 in the control group). | Weight data at 3, 6, 9 and 12 months from a sub-study (Abed 2015). 87 participants agreed to CMR (cardiac magnetic resonance) imaging (43 in control group, 44 in intervention group at baseline). 69 participants had baseline and 12-month follow-up (33 in control group, 36 in intervention group at 12 months). |
| **2** | **Ackermann 2011** | UNCLEAR | UNCLEAR | LOW | LOW |  |
|  | Assessment justification: | "matched-pair, group-randomized pilot intervention trial involving two YMCA facilities in greater Indianapolis."  No further information provided regarding matching. | NS | Body weight was measured using a calibrated, beam-balanced scale with participants wearing light clothing and no shoes. | Less than 50% attrition at 12-month follow-up. |  |
| **3** | **Agras 1990** | LOW | UNCLEAR | LOW | LOW |  |
|  | Assessment justification: | "computer generated table of random numbers" | NS | Weight objectively measured. | 12-months follow-up:  29/30 computer alone; 29/30 computer + group;  30/30 behaviour therapy |  |
| **4** | **Ahern 2017** | LOW | LOW | LOW | LOW |  |
|  | Assessment justification: | "The randomisation sequence was generated by the trial statistician and allocates participants in a 2:5:5 allocation stratified by centre and gender, with a block size of 12." | " The sequence is unknown to research staff and participants.” | Weight objectively measured.  "Weight and fat mass will be measured in kg using a Tanita segmental body composition analyser." | 3-months retention rate:  Brief intervention: 68%;  12-week programme: 76.4%;  52-week programme: 86.2%;  12-month retention rate:  Brief intervention: 58.7%;  12-week programme: 63.9%;  52-week programme: 68.2%;  24-month retention rate:  Brief intervention: 63%;  12-week programme: 67%;  52-week programme: 69.7%; |  |
| **5** | **Almanza -Aguilera 2018** | UNCLEAR | UNCLEAR | LOW | HIGH |  |
|  | Assessment justification: | Participants were randomly allocated to either the control or the treatment group.  No further information given. | NS | "Anthropometric measurements, including weight, height, waist circumference (WC), and BMI, were taken by trained nurses..." | "Of the 115 participants recruited, 58 were excluded due to dropout or failure to show at all visits (n = 43), illness (n = 6), unavailable sample at some time point (at baseline, 3 or 12 months, n = 7), or change of residence (n = 2). Therefore, 57 participants were included in the present data analyses." Control n = 27 analysed out of 48 randomised; Treatment n = 30 analysed out of 67 (44.7% retained). |  |
| 6 | **Anderson 2014** | LOW | LOW | LOW | LOW |  |
|  | Assessment justification: | “A statistician, independent of the analysis of study outcomes, had previously generated a randomisation list (site specific identification numbers and group allocation) by using a permuted block technique, with block sizes of four and eight, stratified by trial site.” | “This list was emailed to the study administrator and trial manager. Research nurses allocated participants a site specific identification number sequentially and notified the study administrator on completion of baseline measures for each participant. The study administrator then identified the participant’s group allocation from the randomisation list and notified the lifestyle counsellor of participants allocated to the intervention group or sent the weight loss booklet to participants allocated to usual care.” | Weight objectively measured.  “The study team, including the research nurses, were blinded to the participant’s group allocation until completion of the primary outcome analysis. Exceptions were the trial manager, study administrator, lifestyle counsellors, and participants who could not be blinded owing to the nature of the intervention. None of these unblinded staff had a role in data analysis.” | “The remaining 329 were randomised (163 to intervention, 166 to control). At three months 314 (94% intervention, 97% control) participants had completed the primary outcome measures, and 305 (91% intervention, 95% control) completed the trial at 12 months (93%).” |  |
| **7** | **Appel 2011** | LOW | LOW | LOW | LOW |  |
|  | Assessment justification: | "Randomization was stratified according to sex and was generated in blocks of 3 and 6 with the use of a Web-based program." | Web-based program. | "Participants were asked to make in-person follow-up visits 6, 12, and 24 months after randomization. At each of these visits, weight was measured on a high-quality, calibrated digital scale, with the participant wearing light, indoor clothes and no shoes." | 6-month follow-up: Control: 113/138*100= 81.9%;  Remote: 129/139*100 = 92.8%;  In-person: 124/138*100 = 89.9%  12-month follow-up: Control: 108/138*100= 78.3%;  Remote: 124/139*100 = 89.2%;  In-person: 123/138*100 = 89.1%  24-month follow-up : Control: 129/138*100= 93.5%;  Remote: 132/139*100 = 95%;  In-person: 133/138*100 = 96.4% |  |
| **8** | **Ard 2004** | LOW | LOW | LOW | LOW |  |
|  | Assessment justification: | Randomization assignments were made centrally by a computer program. Assignments were stratified by clinic and hypertension status; the randomization block size was 24. | Randomization assignments were made centrally by a computer program. | Weight was measured using a calibrated scale. | Less than 25% attrition at 6-month and 18-months follow-up. |  |
| **9** | **Ard 2018** | LOW | LOW | LOW | LOW |  |
|  | Assessment justification: | The statistician generated blocked random assignments using a computer-based algorithm, stratified by age category (65–74, 75+), sex, and race. | Allocations were concealed in sealed envelopes that were opened by a research assistant at the time of randomization. | Body weight was measured in light clothing on calibrated electronic scales to the nearest 0.1 pound and converted to kilograms. | Less than 50% attrition. |  |
| **10** | **Ash 2006** | LOW | UNCLEAR | LOW | HIGH |  |
|  | Assessment justification: | Randomised by the project manager, using a random number table, into one of three intervention groups at one of two hospital sites. The allocation ratio for the two hospital sites (public and private) was 2:1 due to available resources for implementing the intervention. | NS | Weight objectively measured. | BO = 20/54 complete data 37% IDT = 44/65 complete data 66.7% FBI = 26/57 complete data 45.6% Significant between group difference in drop out and people who dropped out had significantly higher baseline BMI. At 12 months 24 BO, 49 IDT and 29 FBI had weight measurements. |  |
| **11** | **Aveyard 2016** | LOW | LOW | LOW | LOW |  |
|  | Assessment justification: | “An independent statistician used Stata Software version 12 to produce a randomisation list that was stratified by physician, with random permuted blocks of four.” | “Randomisation was done via preprepared randomisation cards labelled with a code representing the allocation, which were placed in opaque sealed envelopes and given to physicians to open at the time of treatment assignment.” | Weight objectively measured. | “We weighed 1419 (75%) of participants at the 12-month follow-up.” |  |
| **12** | **Azar 2013** | LOW | LOW | LOW | LOW |  |
|  | Assessment justification: | Participants are randomized on a 1:1:1 basis to one of three arms: UC, SM, or CM. Pocock's "minimization" procedure is used to assure better than chance group balance with respect to participant age, gender, race, BMI, fasting blood glucose, waist circumference, and use of PAMFOnline, which is PAMF's online patient portal to access his or her own health record (user vs. non-user). For each participant about to be randomized, a computerized randomization algorithm automatically calculates an imbalance score for each of the balancing factors, as the excess or deficit of previously randomized participants in each arm matching the current patient on that factor. These scores are summed over factors to form a total imbalance score, S, for each treatment arm. The randomization probability of assigning the patient to the treatment associated with the smallest S is set to 2/3, and the other two treatments are each assigned a probability of 1/6 based on Efron's biased coin method. | A designated research staff member who is not involved in follow-up data collection or data analysis assigns each study arm a non-revealing label, e.g., A, B, or C, and performs actual randomization of the participants. | Weight objectively measured. | 171/241 participants at 24-months. |  |
| **13** | **Bacon 2002** | UNCLEAR | UNCLEAR | LOW | LOW |  |
|  | Assessment justification: | “To ensure balance in the treatment groups, the enrolled subjects (n = 78) were divided into BMI quartiles, and high/ low sets for dietary restraint, 34 degrees of flexible and rigid control of eating, 35 age, and self-reported activity level. The subjects in these subgroups were then randomly assigned to one of two treatment groups.” | NS | Weight objectively measured.  Blood pressure was assessed in duplicate using the oscillometric technique. Fasting blood samples were analyzed for blood lipids (total cholesterol, low-density lipoprotein [LDL] cholesterol, and high-density lipoprotein [HDL] cholesterol). | 52-weeks:  Diet group: 23/39 completed testing; HAES group: 34 attended (29 completed testing)/36 |  |
| **14** | **Barnes 2017** | UNCLEAR | UNCLEAR | LOW | LOW |  |
|  | Assessment justification: | Participants were randomly assigned, stratified by BED diagnosis, to one of three conditions.  No further information given. | NS | Weight objectively measured. | Less than 50% attrition at 12-month follow-up. |  |
| **15** | **Bartels 2015** | LOW | UNCLEAR | LOW | LOW |  |
|  | Assessment justification: | “Randomization between In SHAPE and the comparison condition was stratified by age (21 to 44 years versus 45 years and older) and psychiatric diagnosis (mood disorders versus schizophrenia spectrum disorders). Each combination of stratification categories had its own randomization schedule that was blocked on every fourth assignment to ensure balance between treatment arms. Randomization was conducted sequentially across all sites (not within sites).” | NS | Weight objectively measured.  'Blood pressure was measured before (resting heart rate) and after completing the 6-MWT'  'Lipids were measured using the CardioChek PA Analyzer, a portable testing system that produces reliable values for total cholesterol, LDL, HDL, and triglycerides using a multi-panel test strip and a single drop of blood acquired with a finger prick.' | 18-months:  Control: 83/106 Intervention: 80/104 |  |
| **16** | **Beavers 2017** | UNCLEAR | UNCLEAR | LOW | LOW | UNCLEAR |
|  | Assessment justification: | "Eligible participants will be randomized to one of the three treatment arms at the end of baseline testing using a stratified (by wave) block randomization scheme." | NS | Weight objectively measured. | "At the 6-month assessment, 90.3% were retained, whereas at 18 months, this value was 77.1%. There was no differential loss to follow-up as a function of treatment group at either time point: p=.07 at 6 months and p=.268 at 18 months." | Cholesterol, glucose and BP measured but not reported. Authors contacted however do not have statistical support to provide further analyse. Judged as unclear for selective reporting. |
| **17** | **Bennett 2012** | LOW | UNCLEAR | LOW | LOW |  |
|  | Assessment justification: | Participants were randomized to treatment arm using computer-generated allocations, blocked by clinic and sex. | NS | Weight objectively measured. | 24-months:  Usual care: 166/185 Intervention: 148/180 |  |
| **18** | **Bennett 2013** | LOW | UNCLEAR | LOW | LOW |  |
|  | Assessment justification: | A computer-generated randomization algorithm to allocate participants equally (1:1) across the 2 treatment arms (intervention and usual care); those in the intervention arm were further randomized to 1 of 2 interventionists. | NS | Weight objectively measured.  Secondary measures included waist circumference, blood pressure, and fasting glucose, triglyceride, and cholesterol level. | Usual care: 90/97 Intervention: 86/97 |  |
| **19** | **Berry 2014** | UNCLEAR | LOW | LOW | LOW | HIGH |
|  | Assessment justification: | Cluster randomization. “The sequence of each school was randomized before the start of the study and was stratiﬁed by county. A total of 18 months had passed and the ﬁrst group had completed their time in the study prior to the second enrollment in each school. This design preserved a balance of treatment groups within each site to avoid confounding site effects with intervention effects.” | “Participants and staff were blinded to group assignment from enrollment until implementation.” | Weight objectively measured. | 59% of control group and 57% of intervention group at last follow up.  "To assess the extent of selection bias owing to attrition, the mean values for BMI percentiles were compared between those participants who did not contribute data beyond the Phase I intervention and those who did. There were no signiﬁcant differences between these groups, either overall or by experimental group (P.0.35). " | Wait-list control |
| **20** | **Bertram 1990** | HIGH | UNCLEAR | UNCLEAR | HIGH |  |
|  | Assessment justification: | 'Randomly selected' 200 informed women with a body mass index (BMI) greater than 30 volunteered to participate in a 16-week study. In order to optimise compliance, only subjects who declared their willingness to be assigned to any one of the three interventions were selected. Fifteen subjects were then randomly selected for each of the above three groups, so that age and BMI were similar. | NS | NS | At 16 weeks "Of the 45 subjects who started the project, 36 completed the 16-week course: 2 subjects became pregnant and were withdrawn from the exercise group, while 7 subjects 'absconded' from the control group and were lost to the study. There were no withdrawals from the lecture group. "At follow up "Unfortunately we were only able to re-test 12 of the 36 subjects who completed the original study; 18 of the remaining 24 were unable to participate either because they had changed residence or because they were employed and unable to attend re-evaluation sessions. The remaining 6 subjects admitted to having gained weight, and refused to participate in the follow up study. All 6 of these subjects were from the control group. Of the 12 subjects re-tested, 3 were from the exercise group, 7 from the lecture group, and 2 from the control group." |  |
| **21** | **Bertz 2012** | LOW | UNCLEAR | LOW | LOW |  |
|  | Assessment justification: | Random number table | Allocation method not reported but described as ‘concealed’. | Weight objectively measured.  Body composition was measured by using dual-energy X-ray absorptiometry (DXA) (Lunar Prodigy; GE Lunar Corp). Muscle mass was calculated from DXA. | 92% followed up at 12-months, intervention 100%, D 76%, E 83%, control 76%. 4 missing (6%); 2 medical reasons (3%). |  |
| **22** | **Bo 2007** | LOW | LOW | LOW | LOW |  |
|  | Assessment justification: | “The randomization procedure was automatically performed by a statistician using an SAS program developed to minimize the differences between the two groups for all stratifying variables. The patients were randomly allocated to receive either standard lifestyle recommendations from their physicians (control group, n=188) or a structured lifestyle intervention program for 1 year carried out by health professionals (intervention group, n=187).” | “Random allocation with a minimization algorithm was centrally performed in a single step. The researchers then received the two lists of nominative data. The possibility for researchers to predict or influence the allocation of participants was thus completely prevented.” | Weight, waist circumference, and blood pressure were measured. Fasting glucose, insulin, triglycerides, high-density lipoprotein (HDL) cholesterol, uric acid, and hs-CRP values were measured before and after the study in both groups. | 12-months:  Control: 166/188 Intervention: 169/187 |  |
| **23** | **Brown 2014** | LOW | UNCLEAR | LOW | LOW |  |
|  | Assessment justification: | Randomised block design. Computer-generated random assignment was used to assign an equal number of individuals from each risk group to the weight loss program (RENEW) or to a control group. | NS | Weight objectively measured. | 136 at baseline. 92 at 12 months (47 intervention; 45 control) |  |
| **24** | **Burke 2005** | LOW | UNCLEAR | LOW | LOW |  |
|  | Assessment justification: | Random allocation - computer-generated random numbers. Random allocation to groups was stratiﬁed by age and BMI and used a block size of 4. | NS | Weight objectively measured. | Control:  98/118 at 4 months; 90/118 at 1 year follow up (16 months);  64/118 at 3 year follow up (40 months). Intervention:  106/123 at 4 months; 102/123 at 16 months; 76/123 at 40 months. |  |
| **25** | **Burke 2015** | LOW | UNCLEAR | LOW | LOW |  |
|  | Assessment justification: | Randomization used the minimization method. Treatment assignments were determined considering gender and ethnicity (White vs. non-White) to ensure balance across the treatment groups. | NS | "Data were collected at the research center by trained staff using standardized procedures and questionnaires. Equipment was standardized and routinely calibrated" | 79.2% in SBT arm and 81% in SBT+SE arm completed 18-month assessment. |  |
| **26** | **Cheyette 2007** | UNCLEAR | UNCLEAR | LOW | LOW |  |
|  | Assessment justification: | "Non probability volunteer sampling was used to assign people to either the intervention or the control group." | NS | Weight objectively measured. | " At six and 12 months follow up a total of eight people dropped out from the intervention group and two from the control group." |  |
| **27** | **Christensen 2012** | LOW | LOW | LOW | LOW | LOW |
|  | Assessment justification: | Cluster-randomization procedure. A cluster formation of the groups was performed to assure equal allocation in the intervention and reference groups balanced on sex, age, job seniority or job type with cluster size varying from 3 to 15. | The randomization was done by an external research group, which had no knowledge of the work place or the participants. Clusters were randomly allocated to intervention and control by the drawing of sealed envelopes from a bag. | The test manager was blinded regarding the participants intervention status, and whenever possible the same test manager tested the subject at all three rounds of tests | 98 participants --> 83 participants. | Clusters were created based on information from the screening questionnaire and the management of working teams, day and evening/night shifts and close working relations. This approach was chosen to avoid contamination, and to benefit from the social support in work teams, thereby in- creasing compliance. |
| **28** | **Cleo 2018** | LOW | UNCLEAR | LOW | LOW |  |
|  | Assessment justification: | “Computer-generated randomization occurred after baseline assessment to allocate participants to either: TTT, DSD, or WL control (allocation ratio 1:1:1). We used minimization stratiﬁed on BMI categories (overweight, obese class I, II, III); age (18–32, 33–47, 48–62, 63–75 years); and gender.” | NS | Weight objectively measured. | At 12-months:  21/25 (84%) 22/25 (88%) |  |
| **29** | **Conroy 2015** | UNCLEAR | LOW | UNCLEAR | LOW |  |
|  | Assessment justification: | “…randomization occurred in a 1:1 allocation. Each woman was allowed to draw a sealed envelope that contained a designation assignment, either interventionist-led (IL) or self-guided (SG).” | Sealed envelopes. | Weight was measured by a trained staff member in clinic using a standard balance beam scale (SECA Medichoice) and following a written protocol.  " For the 12-month followup, 62 (74 %) of 84 participating women had an in-person assessment (with study-measured weight), with the remainder of the outcomes assessed by phone." Breakdown by group not clear | “Follow-up was better in the IL group (90 % at 3 months and 96 % at 12 months) than in the SG group (63 % at 3 months and 76 % at 12 months), but otherwise did not differ by other participant characteristics.” |  |
| **30** | **Cooper 2010** | LOW | LOW | LOW | LOW |  |
|  | Assessment justification: | “Participants were allocated to the three treatments by HAD (who had no involvement in participant recruitment) using a stratified computer-generated randomization scheme with random permuted blocks of varying size within two strata. Participants were assigned to the two strata on the basis of their binge eating frequency with those reporting 12 or more episodes over the previous 12 weeks being classed as belonging to a binge eating subgroup.” | “The allocation sequence was concealed in numbered sealed opaque envelopes. At the point of randomization, the next envelope in the sequence was opened by one of the two senior clinicians.” | Weight objectively measured. | At 36-months the following completed assessment:  44/51 GSH  44/50 BT  46/49 CBT |  |
| **31** | **Cousins 1992** | UNCLEAR | UNCLEAR | LOW | LOW |  |
|  | Assessment justification: | “Subjects were stratified according to weight and randomly assigned to one of the three treatment groups.” No further information given | NS | Weight and height measured "using a standard physician's scale." | Total at start 168.  82 excluded because of missing data. 86 completed >50% LOW The remaining 82 subjects were excluded because of missing data at any of the 3-, 6-, and 12-month measurement sessions. Preliminary ANOVA revealed no significant differences on any of the baseline measures, including initial BMI, initial weight, age, acculturation, years of education, or income between the 86 subjects included in these analyses and the 82 who were excluded. |  |
| **32** | **Craighead 1989** | UNCLEAR | UNCLEAR | LOW | LOW |  |
|  | Assessment justification: | “The subjects who met the qualifications for the study were rank-ordered according to pounds overweight and randomly assigned within blocks of three to one of the experimental conditions. Two groups were formed within each condition depending on subject's availability for meeting times.” | NS | 'subjects were weighed' at baseline, at each session, at end of 12 weeks and at 1 year. | 62 recruited, 42 at 12 weeks, 38 at 1 year 14 dropped out. 'The dropout rate was not significantly different among the groups' The treatment analyses were conducted on the 42 subjects who fully participated in the treatment condition to which they had been assigned. |  |
| **33** | **Dale 2009** | UNCLEAR | UNCLEAR | LOW | LOW |  |
|  | Assessment justification: | NS | NS | Weight objectively measured.  “At the time of the euglycemic insulin clamp study, fasting blood samples were taken for lipid measurements, and anthropometry and blood pressure measurements were repeated.” | 87% followed up at 12 months (87% MI, 92% II, 87% control). Reasons for attrition not reported. Reviewers assumed equal loss to follow-up between intervention arms. |  |
| **34** | **Dalziel 2006** | UNCLEAR | UNCLEAR | UNCLEAR | LOW | HIGH |
|  | Assessment justification: | NS | NS | NS | "Shortly after randomisation, 21 (8 in the controls and 13 in the experimental group refused follow-up) (table 1)." The mean rate of withdrawal from follow-up was similar in the experimental (8%) and control (7%) groups. | Did not explicitly aim for weight loss so may introduce clinical heterogeneity into the review. Included after discussion as dietary intervention versus control. |
| **35** | **Damschroder 2014** | LOW | UNCLEAR | LOW | LOW |  |
|  | Assessment justification: | “…a biostatistician provided block randomized assignments (by medical center and two BMI categories [o35 or Z35] to ensure balance between groups) using random permutated blocks constructed by Stata’s *ralloc* command; block sizes ranged from 3 to 9…” | “Investigators were blind to assignments until baseline assessments were complete.” | Anthropometric measures (height, weight, and waist circumference); blood pressure; and self-reported measures including a Food Frequency Questionnaire; EuroQoL-5D utility assessment (with level of painsubscale); Satisfaction with Life Scale; demographic characteristics; laboratory testing for cholesterol and glucose metabolism; and a 6-minute walk test were collected in baseline, 3-month, and 12-month assessments. | Move:  3mth: 115/159;  12mth: 119/159  Aspire phone:  3mth: 131/162;  12mth: 120/162  Aspire group:  3mth: 127/160;  12mth: 122/160;  Follow up 332/481 consented to long term follow up. Move:  18mth: 92/112;  24mth: 90/112  Aspire phone:  18mth: 95/105;  24mth: 92/105  Aspire group:  18mth: 102/115;  24mth: 104/115 |  |
| **36** | **Daumit 2013** | UNCLEAR | UNCLEAR | LOW | LOW |  |
|  | Assessment justification: | "Randomization was stratified according to sex and study site; assignments were generated in blocks of two and four." No further detail given | NS | Weight objectively measured. Measurements of blood pressure, waist circumference, and fasting blood chemical levels were obtained at baseline and at 6 and 18 months. | Control: 142/147 Intervention: 137/144 |  |
| **37** | **deVos 2016** | UNCLEAR | LOW | LOW | LOW | LOW |
|  | Assessment justification: | "...subjects were randomized using consecutive case numbers. For the diet-and exercise program, subjects were randomized 1:1 using block randomization with block size 20." | "A research assistant not involved in the trial provided a sealed envelope that was opened by the subject in the presence of the researcher." | "For the first 2.5 y, all participants were home-visited every 6 mo by a research assistant..." "Body weight was also measured during these visits." "After 6.6 y, participants were visited once more for measurements and a questionnaire." | After 2.5 y, 10.1% of the participants were lost to follow-up. After 6.6 y, 247 participants (60.7%) agreed to additional measurements and questions. "No significant difference in attrition rate was found between the randomly assigned groups." | Original study design included 4 groups ((1) Lifestyle intervention plus placebo; (2) Lifestyle intervention plus Glucosamine; (3) Control plus placebo; (4) Control plus Glucosamine) which were combined into two groups. "The preventive effects of a weight-loss program and of oral glucosamine sulfate compared with placebo on the incidence of knee osteoarthritis were investigated in a 2x2 factorial design with a follow-up time of 6.6 y." No effects of glucosamine on these outcomes were expected or detected. Therefore, the glucosamine intervention will be disregarded in the present manuscript." |
| **38** | **Diabetes Prevention Program R G 2009** | LOW | LOW | LOW | LOW | HIGH |
|  | Assessment justification: | “The randomization was done centrally by computer…”  Random treatment assignments were stratified according to clinical center and were generated by the coordinating center through computer linkup to the field center at time of randomization. Therefore, assignment was unknown until randomization. Assignments to metformin and placebo were double-blinded. | “…assignments to the lifestyle group were blinded until randomization, while assignments to the medication groups were blinded until the end of the study.” | Lifestyle intervention participants were weighed privately at the start of every individual session and were encouraged to weigh themselves at home daily or a minimum of once per week. | Placebo yr. 1 - 1027/1082;  yr. 2 - 1015/1082;  yr. 3 - 975/1082.  Bridge period DPPOS - 1085 eligible, 935 enrolled. DPPOS  yr. 1 882/935;  yr. 2 874/935;  yr. 3 844/935;  yr. 4 - 827/935;  yr. 5 - 846/935;  yr. 6 808/935;  yr. 7 - 789/935;  yr. 8 766/935;  yr 9. 760/935;  yr. 10 - 763/935;  yr. 11- 769/935.  Lifestyle  yr. 1 - 1026/1079;  yr. 2 - 1001/1079;  yr. 3 - 972/1079.  Bridge period DPPOS - 1068 eligible, 914 enrolled. DPPOS  yr. 1 855/914;  yr. 2 827/914;  yr. 3 816/914; yr. 4 - 810/914;  yr. 5 - 824/914;  yr. 6 783/914;  yr. 7 - 763/914;  yr. 8 757/914;  yr. 9 738/914;  yr. 10 - 725/914; yr. 11.- 738/914. | 0-3 years LOW  From year 4 HIGH DPP was a 3-year randomized clinical trial followed by open-label modified intervention follow-up. |
| **39** | **Djuric 2002** | UNCLEAR | UNCLEAR | HIGH | LOW | HIGH |
|  | Assessment justification: | 48 patients were randomly assigned into four research groups by random block design. At baseline: “There were no differences among the four groups in body weight and BMI. Nevertheless, there were significant differences in percentage body fat, total cholesterol, and LDL-C at baseline, indicating that the block randomization process did not equalize all parameters among groups. However, the highest values of these parameters were not consistently found in any one group.” | NS | 'Weighed in clothing but without shoes using a professional beam scale (model 402KLS; Health-o-Meter, Bridgeview, IL), and percentage of body fat was measured using tetrapolar bioelectrical impedance (model BIA101S; RJL Systems, Clinton Township, MI). Height was measured at baseline only.' | 18.75% dropped out by end of study.  At 12-months:  Control: 12/13  WW: 8/11  Individualised: 9/13  Comprehensive: 10/11 | Missing outcome data - study states intention to follow up to 30 months but 30-month data not available. Data for 3 and 6 months extracted from graphs but some inconsistency between graphs and what is reported in text. |
| **40** | **Duncan 2016** | LOW | LOW | LOW | HIGH |  |
|  | Assessment justification: | “…participants were randomized into one of two groups using a simple randomization procedure stratified by clinic with a 1:1 allocation ratio.”  “…the order of control and intervention envelopes was distributed at each practice using a computer-generated randomization list.” “Participants were randomized within practices such that some within a practice were assigned to treatment and some to control conditions. Participating practices were not used as the unit of randomization to avoid between-practice effects confounding between-group differences.” | “Practice nurses and physicians were blinded to the designation of the envelopes…” (notifying participants of allocation to either control or intervention) | Weight objectively measured. | While participant drop-out from baseline to 4 months was 49%, the lack of between-group differences in baseline demographic and health indicators in individuals that dropped out of the study indicates that systematic bias was not introduced.  "Of the 320 participants randomly assigned to control and intervention groups, 156 (48.8%) were followed-up at 4 months, with 157 (49.1%) at 12 months." |  |
| **41** | **Eakin 2014** | LOW | LOW | LOW | LOW | LOW |
|  | Assessment justification: | "Randomization was by the minimization method (18) using the MINIM program (www.sghms.ac.uk/depts/phs/guide/randser.htm)." | "Allocation is performed using the free Minim computer software [48] and conducted by a research assistant with minor involvement in participant recruitment." | "Data are collected via objective measurements conducted in participants’ homes, telephone interviews, and selfadministered questionnaires at baseline, 6-, 18-, and 24- months by research staff and registered nurses blind to participants’ study group." | "Attrition at 24 months was nondifferential and modest in both groups, yet ;40% of telephone counseling participants chose to discontinue receiving the intervention by withdrawal from either the intervention or study participation altogether." | "... even among telephone counseling group participants who did not withdraw, intervention delivery was difficult, with just over half of participants completing at least 75% of scheduled intervention calls." |
| **42** | **Eaton 2016** | LOW | LOW | LOW | LOW |  |
|  | Assessment justification: | “After the baseline visit was completed, participants were block randomized within practice in pairs using a random number generator created by the data manager with SPSS for Windows, version 11.0 (IBM).” | “After completion of the initial lifestyle counseling session, the research assistant gave each participant an envelope that revealed the study arm to which the participant was assigned.” | Height, weight, waist circumference, resting heart rate, and resting blood pressure were measured at each visit. | Control:  78/106 at 6mths; 75/106 at 12mths; 77/106 at 18mths; 75/106 at 24mths Enhanced intervention: 88/105 at 6mths; 84/105 at 12mths; 75/105 at 18mths; 73/105 at 24mths |  |
| **43** | **Fernandez-Ruiz 2018** | UNCLEAR | UNCLEAR | LOW | LOW |  |
|  | Assessment justification: | Randomisation was performed using a simple table of numbers: 37 patients in the control group and 37 in the experimental group.  “A random allocation sequence was generated by a member of the scientific staff through extraction of successive numbered balls from an opaque container, alternating between the experimental and the control group' | Refer to ‘Random sequence generation (selection bias)’. | “The efficacy of the intervention was evaluated through anthropometric (body mass index, weight, different parameters, and skinfolds, as stated in Section 2) and cardiovascular measures taken before, during, and after intervention.' 'Anthropometric and cardiovascular measures were taken at the pretest stage, every 6 months during the programme, and 1 year after it finished.” | No loss to follow up reported. |  |
| **44** | **Foley 2016** | LOW | UNCLEAR | LOW | LOW |  |
|  | Assessment justification: | “Randomization occurred at the baseline visit, using a computer-based algorithm. The randomization algorithm allocated participants equally (1:1) across treatment arms, after accounting for CHC, gender and ethnicity (Hispanic vs. non-Hispanic) in order to ensure the equal representation of these characteristics across arms.” | NS | Weight measured objectively. | Less than 50% attrition at 12-month follow-up. |  |
| **45** | **Foster-Schubert 2012** | LOW | LOW | LOW | LOW | UNCLEAR |
|  | Assessment justification: | “The random assignment was generated by a computerized program, stratified according to BMI (<30 kg/m2 or ≥30 kg/m2) and participants’ self-reported race/ethnicity (non-Hispanic white, black, or other). In addition, to achieve a proportionally smaller number of women assigned to the control group, a permuted blocks randomization with blocks of four was used, wherein the control assignment was randomly eliminated from each block with a probability of ~1 in 4.” Blocked-randomisation. (Permuted-block randomization (ratio 0.75 : 1 : 1 : 1) to assign a proportionally smaller number of women to the control group.) | Central computerised allocation. | Weight measured objectively. | 91% followed up at 12m overall: 92% D+E, 89% D only, 91% E only, 92% usual care. 2 unavoidable losses (<1%); 8% missing; 1% medical reason. | Control group received intervention at 12m, unclear if they knew in advance. |
| **46** | **Fuller 2012** | UNCLEAR | UNCLEAR | LOW | LOW |  |
|  | Assessment justification: | The randomisation process was completed by the study database (Filemaker Pro), upon entry of the participant’s initials and fulfilment of trial requirements. | NS | Weight objectively measured. | 22/35 Korean group and 28/35 Western group completed the study. |  |
| **47** | **Goodwin 2014** | LOW | UNCLEAR | LOW | LOW |  |
|  | Assessment justification: | "Random assignment was performed centrally by the Ontario Clinical Oncology Group, and a computer-generated block randomization scheme with blocks of various size was used." | NS | Weight objectively measured. | "Six patients in the mail-based intervention and seven in the LI arm did not complete the 24-month intervention period because of a primary outcome event (new disease, metastases, death); of the remaining patients, 14 (8.7%) and 16 (9.9%), respectively, withdrew (including patients who transferred care, those who were lost to follow-up, or those with noncompliance). Month-24 weight measurements were available from 264 (90.1%) of 293 participants still on the study." |  |
| **48** | **Green** **2015** | LOW | UNCLEAR | LOW | LOW |  |
|  | Assessment justification: | Minimisation. Participants were assigned to intervention or usual-care using a stratified blocked (on gender and BMI [27–34.9 and ≥35]) randomization procedure, within sites. We used computer and paper-based randomization systems; sequence generated by author NAP. | Staff not involved in data collection informed participants about randomization. Others were blinded to assignment, and participants were routinely reminded not to discuss assignment during assessments. Usual care participants were free to pursue alternative weight-loss efforts. | Blinded staff collected data at all study periods, including scale-measured weights. | Follow-up rates were 90.5% of participants at 6 months (n = 181), 85% at 1 year (n = 170), and 81.5% (n = 164) at 2 years (83.2% if 3 deaths are removed). We found no significant differences in attrition between study arms at any assessment point. |  |
| **49** | **Grilo 2011** | LOW | UNCLEAR | LOW | LOW |  |
|  | Assessment justification: | "Randomization to treatment was performed without any restriction or stratification, using a computer-generated sequence." | "Randomization was determined after formal acceptance into the study and completion of all assessments. Randomization assignment was kept from participants until the start of treatment." | "Weight and height were measured at baseline and again immediately prior to beginning treatment using a trade-legal medical balance-beam scale. Weight was measured bi-weekly throughout treatment, at post-treatment, and at 6- and 12-month follow-ups." | Retention rate:  Post-intervention:  CBT: 75.6%;  BWL: 68.9%;  CBT+BWL: 60.0%  6-Months: CBT: 82.2%;  BWL: 86.7%;  CBT+BWL: 85.7%  12-Months:  CBT: 82.2%;  BWL: 82.2%;  CBT+BWL: 71.4%  "Completion rates, which did not differ statistically, were: 76% (N=34) for CBT, 69% (N=31) for BWL, and 60% (N=21) for CBT+BWL. Follow-up (6-and 12-month) assessments were obtained for over 80% of patients (Figure 1)." |  |
| **50** | **Grilo 2014** | LOW | LOW | LOW | LOW |  |
|  | Assessment justification: | "Randomization to treatment assignment occurred in the exact order following completion of all assessments and medical approval and was performed independently from the investigators by a research-pharmacist at a separate Yale facility using a computer-generated schedule generated by a biostatistician. Participants were randomly assigned with stratification by BED status." | "Randomization to treatment assignment occurred in the exact order following completion of all assessments and medical approval and was performed independently from the investigators by a research-pharmacist at a separate Yale facility using a computer-generated schedule generated by a biostatistician. Participants were randomly assigned with stratification by BED status." | "The assessments were performed independently by doctoral research evaluators at our research clinic who were blinded to both the medication status and to whether participants received the shCBT." | "Post-treatment assessments were obtained for 84% of patients and follow-up assessments were obtained for 83% of patients at the 6-month follow-up and for 86% of patients at the 12-month follow-up."  Retention rates: Post-treatment: Placebo: 20/27*100=74.1%; Placebo/CBTsh: 22/25*100 = 88%  6-Months: Placebo: 22/27*100=81.4%; Placebo/CBTsh: 23/25*100 = 92%  12-Months: Placebo: 19/27*100=70.4%; Placebo/CBTsh: 21/25*100 = 84% |  |
| **51** | **Hanson 1976** | UNCLEAR | UNCLEAR | UNCLEAR | HIGH |  |
|  | Assessment justification: | NS | NS | NS | Attrition greater than 25% at 5-month follow-up and approximately 50% at 1-year follow-up. |  |
| **52** | **Hardcastle 2013** | LOW | UNCLEAR | LOW | LOW |  |
|  | Assessment justification: | "A statistician, who had no contact with the participants, was asked to develop a randomisation protocol such that participants were allocated to the MI intervention and minimal intervention groups by a ratio of 7:5. The randomisation protocol was stratified by gender and age based on patient records. The patients within each stratum were divided into blocks of 12 and then randomly allocated to the MI intervention and minimal intervention groups using computer generated random numbers by the predetermined ratio." | NS | "The practice nurse was blind to the treatment allocated to each patient at baseline and subsequent assessments." | At 18 months, 41% from the intervention group and 31% of the control group were lost to follow-up. |  |
| **53** | **Harrigan 2016** | LOW | LOW | HIGH | HIGH |  |
|  | Assessment justification: | "Permuted-block randomization with random block size was performed by the study biostatistician" | "...blinded study staff using unmarked envelopes." | "Height (using a stadiometer) and weight were measured at baseline and 6 months." "... self-reported weight from baseline to 12 month" | Because there were 15 (15%) individuals who were missing body weight measurements at 6 months, multiple imputation with data augmentation under the multivariate normal model was conducted using SAS PROC MI, as described by Allison.15 The final results were consistent with the results without multiple imputations.  Completed 12-Months: Usual care: N = 19 (58%);  Telephone: N = 15 (44%);  In-Person: N = 22 (67%) |  |
| **54** | **Harris 2017** | LOW | LOW | LOW | LOW |  |
|  | Assessment justification: | "The researcher will telephone an interactive voice response system (IVRS), hosted by the Robertson Centre for Biostatistics, University of Glasgow. The researcher will register each participant in the study, by giving the participants’ cluster number, the number of individuals within the cluster, level of intellectual disabilities and presence of Down Syndrome. After registering each participant, the system will notify the principal investigator of the allocation (TAKE 5 intervention or WWToo intervention)." | "After registering each participant, the system will notify the principal investigator of the allocation (TAKE 5 intervention or WWToo intervention)." | "A researcher (L. H.) who was blind to study group allocation was responsible for collecting all outcome measures, completed at baseline, at 6 and 12 months." "Weight in kg was measured to the nearest 100 g, using SECA877 scales (SE approval class III; SECA)." | WWToo had no lost to follow-up at 12 months; TAKE 5 intervention group had 2 participants lost to follow-up at 12 months. |  |
| **55** | **Hunt 2014** | LOW | LOW | LOW | LOW | HIGH |
|  | Assessment justification: | “After baseline measurement, the randomisation sequence was generated by the Tayside Clinical Trials Unit (TCTU) statistician (with no day to day role in the study at this point) with SAS (version 9.2), blocked (block size between two and nine dependent on how many participants were recruited at a club), and stratified by club.” | “The allocation sequence was sent in a password protected file to a database manager (not part of the research team) who assigned individuals to each group.” | Weight measured objectively. | Comparison (control): 347/374 at 12-weeks; 355/374 at 12-months. Intervention:  330/374 at 12-weeks; 333/374 at 12-months | 12 month wait-list control. |
| **56** | **Huseinovic 2016** | LOW | LOW | LOW | LOW |  |
|  | Assessment justification: | "...simple randomization procedure that used numbered and sealed envelopes generated through a random number table prepared by the project coordinator." | Refer to ‘Random sequence generation (selection bias)’. | "All study measures and administration of intervention were completed by 2 dietitians at the primary health care clinics. Blinding of the study dietitians was not possible..." | D Group:  Baseline n=54;  12-wk: n=47;  1-year: n=44 C Group:  Baseline n=56;  12-wk n=53;  1-year: n=45 |  |
| **57** | **Irwin 2003** | LOW | LOW | LOW | LOW |  |
|  | Assessment justification: | “Randomization was performed by random number generation…”  “Randomization was stratified by BMI (<27.5 vs >27.5) to ensure equal numbers of heavier and lighter women in each study group.” | “…group assignment was placed in a sealed envelope…” | Weight measured objectively. | Less than 50% attrition at 12-months follow-up. |  |
| **58** | **Jackson 1982** | UNCLEAR | UNCLEAR | LOW | UNCLEAR |  |
|  | Assessment justification: | NS | NS | "Both groups were weighed weekly by a teacher and a teacher's assistant to ensure reliability of measurements." | Authors do not report that any participants were lost to follow-up. |  |
| **59** | **Jakicic 2011** | LOW | UNCLEAR | LOW | LOW |  |
|  | Assessment justification: | "Randomization was performed by the study statistician using a computer program with randomization blocked by gender." | NS | "Body weight was assessed using a calibrated balance-beam scale to the nearest 0.1kg (0.25 pounds) with the subject clothed in a cloth hospital gown." | "There was no significant difference in attrition rates between groups based on χ2 analysis."  At the 18-month assessment 82.1% from Self-help group, 72.4% from the Moderate PA group and 81.8% from the High PA group contributed to assessment data. |  |
| **60** | **Jebb 2011** | LOW | LOW | LOW | LOW |  |
|  |  | "The randomisation sequence was computer generated with Stata (version 9.0) by APM and built into the database by the data manager, who was independent from the study team, and was stratified by country, sex, and diabetes status, with an upper limit of 50% of participants with diabetes." | "Treatment allocation was concealed by use of an online database (Filemaker Pro 9, version 3)." | "In the UK and Australia, bodyweight (in light clothes without shoes) and fat mass were measured with a Tanita BC-418 segmental body composition analyser (Tanita Corporation of America, Arlington Heights, IL, USA). In Germany, weight was measured in GP practices with standard scales, and fat mass was measured at the research centre with the Tanita BC-418."  "All participants who did not complete the 24-month visit but had not formally withdrawn from the study were asked to provide self-reported weights in a telephone follow-up survey (Australia and the UK) or a postal survey (Germany)." Majority was objectively reported. | 12-month retention rate: Standard care: 214/395*100 = 54.2% Commercial programme: 230/377*100 = 61%  18-month retention rate:  Standard care: 115/395*100 = 29.1% Commercial programme: 121/377*100 =32.1%  24-month retention rate: Standard care: 98/395*100 = 24.8% Commercial programme: 105/377*100 = 27.9% |  |
| **61** | **Jebb 2017** | LOW | LOW | LOW | LOW |  |
|  | Assessment justification: | "An independent statistician produced a computer generated randomisation list with 1:1 allocation using stratified block randomisation" | "After the nurse had confirmed eligibility, participants were enrolled in the study and the allocation was revealed using an online randomisation programme to ensure full allocation concealment. " | Weight was objectively measured. | 95/140 UC and 104/138 TDR followed up at 12 months. |  |
| **62** | **Jeffery 1995** | UNCLEAR | UNCLEAR | LOW | LOW | UNCLEAR |
|  | Assessment justification: | “Study participants were randomized within centre and sex to one of five treatment groups.”  No further information given. | NS | Weight measured objectively. | Retention rates for total sample: 6-month follow-up: 89% of total sample  12-month follow-up: 87% of total sample  18-month follow-up: 85% of total sample  "There was no differences among treatment groups at any individual follow-up point in the percentage of participants completing assessments. However, the percentage of participants who completed all three follow-ups differed by treatment group: control group (70%), SBT (65%), SBT + FP (90%), SBT + I (85%), and SBT + FP +I (83%)." 30 month follow-up: 88% of total sample "There were no differences among treatment groups, centers or sex in the percent of participants lost to follow-up." | Weight data reported in text does not seem to match that presented in the figure for the 30-month data for the SBT group. |
| **63** | **Jenkins 2017** | LOW | LOW | LOW | HIGH |  |
|  | Assessment justification: | "A statistician not involved in the day-to-day operation of the interventions created blocks of random assignments (n=39)." | "Assignments were sealed in ordered, numbered, opaque envelopes. Upon consent and eligibility confirmation for the individual or household, the coordinator opened each envelope in sequence and assigned the participant to the treatment group it contained." | "Completed questionnaires, fasting blood, anthropometric, and blood pressure measurements were obtained at baseline and at subsequent clinic attendances at 6 and 18 months at St. Michael’s Hospital." | 6-month retention of 91% in the 2 food delivery arms versus 67% when no food was provided. When no food was provided, groups which had received a prior provision of food resulted at 18 months in an 81% retention versus 57% where no food had been provided. |  |
| **64** | **John 2011** | LOW | UNCLEAR | LOW | LOW | HIGH |
|  | Assessment justification: | "Participants were randomized evenly to participate in a weight-monitoring program (control condition) or the same program with one of two financial incentive plans (deposit contract condition, hereafter referred to as DC) using a block size of six, with stratification based on sex and age (30–49 vs. 50–70)." | NS | "At the end of each month, participants received $20 for returning to the clinic to be weighed." | 32 weeks follow-up: "At the primary outcome point, ten percent of participants were lost to follow-up (C = 1/22, DC1 = 1/22, DC2 = 4/22)" 68 week follow-up: Sixty-five percent of participants [43/66; Control = 14/22*100 = 63.6%; DC = 29/44* 100 = 65.9%] returned to the clinic for a follow-up weigh-in approximately 36 weeks after the last participant had completed the 32-week intervention. | Study involved randomisation to 3 arms: (1) weight-monitoring program (control condition); (2) weight-monitoring program plus deposit contract condition 1; (3) Weight-monitoring program plus deposit contract condition 2. They differed as follows: "For half of the DC participants, the first 24 weeks of the study were described, in both written and verbal communication to participants, as the ‘weight loss period;’ the final 8 weeks (i.e., weeks 25–32) were framed as the ‘maintenance of weight loss period’ (DC1). The second incentive condition was the same except that there was no explicit distinction between the two periods of the study (DC2), which was also the case in the control condition." The two deposit contract condition groups have been extracted as 1 group as only reported as such Varying follow-up depending on time of recruitment: "To assess longer term maintenance following the 32-week intervention, participants returned for a weigh-in approximately 36 weeks after the last participant had completed the 32-week intervention."; "Recruitment began in June 2008 and ended in September 2008; follow-up ended in January 2010." |
| **64** | **Jolly 2011** | LOW | LOW | HIGH | LOW |  |
|  | Assessment justification: | "The randomisation sequence was prepared by an independent statistician..." | "An independent statistician prepared two separate randomisation sequences, and, to ensure blinding, the allocations were placed in opaque, consecutively numbered envelopes, which the call centre staff used in order. The block sizes were determined to achieve one to one randomisation across groups, except for the two primary care arms, for which spaces were limited and allocation was in a ratio of 1 to 0.7 compared with the other groups." | "When participants attend their first weight-loss session in the six interventions, the leader/counsellor measures participants’ height and weight. Scales are validated by the research team using standardised weights, unless evidence of recent independent validation is provided. The commercial providers often use self-reported height, so this will be re-measured at follow-up by the blinded assessor. People in the comparator control group and people who are randomised but who do not attend their allocated programme are contacted and a researcher makes an appointment to measure height and weight. During the 12-week programmes the service providers record weights on each visit. The comparator group are weighed at baseline only."  "At three months after programme start (programme end) the service providers weigh participants. Participants who are no longer attending their allocated programme are contacted and offered follow-up at home or another convenient location. If participants decline to be followed-up in person, they are asked to provide a self-reported weight, which is recorded as self-report. "  Flow of participants through trial figure highlights that over a third of weight measures were self-reported. | "At programme end, 658 (88.9%) participants were followed up; 522 (70.5%) were followed up at one year (fig 1)." Attrition rate at intervention end (3 months):  Weight watches: 95%; slimming world: 93%; Rosemary Conley: 88%; NHS Size down: 87%; GP: 82.6%;  Pharmacy: 82.3%; Choice: 95%; Comparator: 83% Weight watches: 82%; slimming world: 62%; Rosemary Conley: 74%; NHS Size down: 66%; GP: 65.7%;  Pharmacy: 58.6%; Choice: 79%; Comparator: 72% |  |
| **65** | **Jones 1986** | UNCLEAR | UNCLEAR | UNCLEAR | HIGH |  |
|  | Assessment justification: | NS | NS | "On the first appointment height and weight were measured, using a beam balance scale"; No information on how weight was measured at follow-up time points. | "Of 160 subjects attending an initial interview, only 69 (43%) completed treatment; of these, 58 were seen at the 1-year follow-up." (36.3%) |  |
| **66** | **Jones 1999** | UNCLEAR | UNCLEAR | UNCLEAR | LOW |  |
|  | Assessment justification: | "Patients were randomized in a single blind fashion to either the weight loss intervention group or the control group. Randomization was done in a blocked fashion to ensure that equal numbers of the three HOT treatment groups were in both the weight loss intervention group and the control group." | NS | "Weights for both groups were measured at 6-month intervals during follow-up required by the HOT protocol." | "Four patients in the weight loss group and five patients in the control group did not complete the study and were excluded from the data analysis." |  |
| **67** | **Katzer 2008** | UNCLEAR | UNCLEAR | LOW | LOW |  |
|  | Assessment justification: | "Randomization was stratified by age and BMI and performed independently by a statistician." | NS | Weight objectively measured. | "By the end of the initial 10-week intervention, 53 participants had withdrawn from the study (24%), and an additional 28 participants (overall dropout rate=37%) had withdrawn by the 12-month follow-up. Dropout rates were similar in the three treatments (data not shown)." |  |
| **68** | **Kuller 2012** | LOW | LOW | LOW | LOW |  |
|  | Assessment justification: | Block randomisation. Randomization sequence designed by independent statistician' | “…allocation via sealed, numbered envelopes opened sequentially.” | Weight, height, and waist circumference were measured at clinic visits at baseline, 18 months, and 30 months.  Standard laboratory measurements included total cholesterol, HDL-C, triglycerides, insulin, and glucose after 12-hour fasting samples. | 83% followed up at 18m overall: 82% intervention, 84% control. Reasons for attrition NS. |  |
| **69** | **Leahey 2014** | LOW | UNCLEAR | LOW | LOW |  |
|  | Assessment justification: | With a random number generator, we assigned participants by using a 1:2:2 randomization scheme. | NS | Weight objectively measured. | Less than 50% attrition at 6- and 12-month follow-up. |  |
| **70** | **Ley 2004** | HIGH | UNCLEAR | LOW | LOW |  |
|  | Assessment justification: | “They were then individually randomised to either an intervention group that was asked to consume a reduced-fat (RF), but otherwise ad libitum diet, or a control diet (CD) group that continued with their usual diet. An exception to this individual randomisation was made at one work-site where all six participants were Pacific Island’s women who worked closely together. They were all assigned to the RF group because individual randomisation was impractical. All those found to have diabetes on re-testing were referred to their general practitioners for management, but were still randomised for the study.”  Broken randomisation. | All those found to have diabetes on re-testing were referred to their general practitioners for management but were still randomised for the study. | Weight objectively measured. | less than 50% attrition at 1 - and 5-year follow-up. |  |
| **71** | **Li 2016** | LOW | UNCLEAR | LOW | LOW | UNCLEAR |
|  | Assessment justification: | "After a one‐week run‐in period, participants were randomly allocated to one of the following four groups by computer‐generated random numbers." | NS | "All measurements were conducted with standard procedures by the same clinical staff in the third hospital of Inner Mongolia medical college, who were blinded to the group allocation." | Percentage of participants retained at follow-up at 1 year: Usual care: 98.3%; Diet: 96.2%; 50g oats: 96.3%; 94.9%; "Eleven patients dropped out during the 1-year follow-up due to personal reasons with no difference in drop-out rates among the four groups (p = 0.774)." | Unclear whether groups were stratified by BMI in parent study. "A subgroup of 298 subjects, meeting the Chinese criteria of overweight (body mass index ≥ 24 kg/m2), was selected from 445 adult patients with T2DM, who had participated in the 30-day centralized management of a dietary program and the 1-year free-living follow-up in Baotou, China." |
| **72** | **Lindstrom 2003** | LOW | UNCLEAR | LOW | LOW | HIGH |
|  | Assessment justification: | A randomization list was used | The nurses scheduling visits were blinded to randomisation. Study staff were not blinded. | Weight objectively measured. | At 3 years: 203/ 257 Control 231/ 265 Intervention  At 4 years: 170/257 Control 198/265 Intervention  From 5 years:  166/257 Control  200/265 Intervention | After the decision to end the intervention period, the intervention was continued until each participant’s next scheduled annual clinic visit. The end date thus varied from March 2000 to December 2001. After active intervention (median 4 years, range 1–6 years), participants still free of diabetes and willing to continue their participation (from year 6 - 200 in the intervention group and 166 in the control group) were further followed until diabetes diagnosis, dropout or the end of 2009, with a median total follow-up of 9 years and a time span of 13 years from baseline. |
| **73** | **Liss 2016** | LOW | LOW | LOW | LOW |  |
|  | Assessment justification: | "Prior to the study enrollment phase, randomization lists were generated by a senior statistician using SAS, version 9.2 (Cary, NC). Lists were created using 1-to-1 allocation, with blocks of 4, stratified by Y study site and race (non-Hispanic White; African-American; Other).” | "Randomization blocks were implemented by the study programmer (AC) and pre-loaded into a back-end field of a Microsoft Access (Redmond, WA) database table that was not available to study RAs. After the study RA collected data required for randomization at each participant's screening/enrollment visit, she clicked a button in Access to execute the randomization." | Weight objectively measured. | At 12 months, 78% of participants from the GLI group and 76% from the standard care group returned for outcome assessment. |  |
| **74** | **Little 2016** | LOW | LOW | UNCLEAR | LOW |  |
|  | Assessment justification: | “Upon completion of baseline questionnaires, the website automatically randomly assigned patients (1:1:1) via computer-generated random numbers…” | “Participants and investigators were masked to group allocation at the point of randomisaton…” | “Weight loss was measured with participants lightly clothed without shoes, at the same time every day when possible, with automated digital scales (Tanita Europe BV, Amsterdam, the Netherlands).” “When a blinded weight measurement could not be obtained, we used practice nurses’ recorded weights, and when that was not possible, we used participants’ reported weights.” | Weight loss averaged over 12 months was recorded in 666 (81%) participants.” Control: 136/279 weight at 6mths (HIGH); 227/279 weight at 12mths (LOW). Power + face to face: 148/269 wt at 6mths (LOW); 221/269 wt at 12mths (LOW). Power + remote: 155/270 wt at 6mths (LOW); 218/270 wt at 12mths (LOW). |  |
| **75** | **Ma 2015** | LOW | LOW | LOW | LOW |  |
|  | Assessment justification: | We applied our published dynamic block randomization method to assure better than chance between-treatment balance across six prognostic factors (study site, age, sex, race/ethnicity, BMI, and ACQ score). The method automatically ensures allocation concealment. Participants were randomly assigned to one of two treatment conditions | This randomization procedure not only minimizes imbalance for the chosen baseline covariates between treatment groups and correlated characteristics, but also ensures concealment of treatment allocation, with recruitment staff completely unable to influence allocation. A designated research staff member who is not involved in follow-up data collection or data analysis will carry out randomization using a computerized program. (Ma 2010) The trial design precluded blinding participants or interventionists to treatment assignment; however, the investigators, Data and Safety Monitoring Board members, outcome assessors, and data analyst were masked throughout the trial. Ma 2015 | 'Both groups given a weight scale.' 'Published protocols were used to obtain height (baseline only), weight, waist circumference, and blood pressure measurements.' Indicates weighed. | Control:  157/165 at 6mths; 147/165 at 12mths. Intervention:  154/165 at 6mths; 142/165 at 12mths. |  |
| **76** | **Manzoni 2016** | LOW | UNCLEAR | HIGH | HIGH |  |
|  | Assessment justification: | Authors only mention that the randomization scheme used for selecting the condition was generated by using the Web site www.randomization.com. However, no further information regarding the sequence generation is offered. | NS | Participants’ data were obtained 1 week after the start of the inpatient program, during the last week of hospitalization, and at 1-year follow-up (by postal mail) (LOW). Data at follow-up were self-reported (HIGH). | Control 29/52 at follow up (55.7%) LOW CBT 38/54 at follow up (70%) LOW VR 46/57 at follow up (80.7%) LOW Between group HIGH |  |
| **77** | **Marniemi 1990** | UNCLEAR | UNCLEAR | UNCLEAR | UNCLEAR |  |
|  | Assessment justification: | NS | NS | NS | All participant data used in the analysis but it's unclear how many were lost to follow-up. |  |
| **78** | **Martin 2008** | UNCLEAR | UNCLEAR | LOW | HIGH |  |
|  | Assessment justification: | “Eight physicians practicing at two clinics were randomly assigned to provide one of the two treatment conditions, with four physicians providing personalized weight loss interventions and four providing standard care treatment (two intervention and two control at each clinic). Specifically, the randomization utilized clinic as a stratification variable, and the practices within each stratum were assigned to level of treatment under a balanced randomization. The basis of the randomization was the within-stratum ranks of a uniform (0,1) (pseudo-) random deviate generated for each participating physician. This resulted in a nested design, with participants recruited for the study being identified with the randomization assignment of their primary care physician. The purpose of using a nested design was to reduce the likelihood of treatment contamination in the control subjects, and for this reason, only the intervention physicians were trained in the use of the active treatment components.” | NS | Trained personnel measured participants’ weight and height at each assessment in the physician’s office using a standardized protocol with a calibrated scale and stadiometer. | Completed 6mth programme 106/144 (or 106/137 as 7 become not eligible). At 6mths Standard care: 58/73 (or 58/69) LOW At 6 mths Intervention: 48/71 (or 48/68) LOW 'After accounting for medical exclusions and women lost to follow-up for other reasons, 105 completed the 6-month program, which resulted in an attrition rate of 27%. Another 42 participants discontinued the study by month 9, 51 discontinued by month 12, and 53 discontinued by month 18. Thus, the attrition rates at the 9-month, 12-month, and 18-month follow-up assessments were 29, 35, and 37%, respectively. Comparing between the two treatment groups, attrition was significantly greater (29%) in the intervention group immediately following the active treatment phase (i.e., month 6) as compared with standard care (12%), P < 0.01. Attrition was also greater among intervention participants (44%) at the final follow-up (i.e., month 18) as compared with standard care (23%), P < 0.02.' |  |
| **79** | **Mefferd 2007** | UNCLEAR | UNCLEAR | LOW | LOW | UNCLEAR |
|  | Assessment justification: | NS | NS | "Anthropometric measurements (obtained at baseline and 16 weeks) included height and weight (measured without shoes)..." | A little over ten percent of the participants dropped out of the study during the 16 weeks under analysis in this report, yielding a final sample size of (n = 76) at 16 weeks. All nine dropouts had been assigned to the intervention group.  Retention rate:  16-weeks:  Control: 100%; Intervention: 47/56*100= 83.9% 12-Months:  Control: 25/29*100 = 86%;  Intervention: 44/56*100 = 78.6% | Wait-list control; unclear if control group participants were aware they were wait-listed. |
| **80** | **Melchart 2017** | LOW | LOW | LOW | LOW |  |
|  | Assessment justification: | "Randomization and allocation envelopes were prepared by an independent statistician at the Institute for Medical Statistics and Epidemiology at the Technical University of Munich." | "The trial physicians were instructed to open the sealed envelopes in a strictly sequential order of enrollment and to disclose the allocated treatment arm to the study participant." | "Body weight, height, waist circumference, blood pressure, and heart rates were measured by certified IHM coaches at each of the five examination visits. The teams were trained to perform the examinations in a standardized way (eg, subjects wearing light clothes and no shoes, with use of calibrated scales for measuring weight)." | "... Of 111 subjects who commenced with the IHM group, 17 (15.3%) prematurely discontinued the study, while in the UC group the dropout rate was 18.2% (10 of 55). The majority of dropouts left the study before the control visit at month 3, and this occurred in 10 of 17 cases in the IHM group and in 9 of 10 in the UC group."  Participant dropouts: 3-Months:  IHM: 90.8%; UC: 84%;  6-Months:  IHM: 88.1%; UC: 82%;  9-Months:  IHM: 85.3%; UC: 82% 12-Months:  IHM: 84.4%; UC: 84% |  |
| **81** | **Melin 2003** | UNCLEAR | UNCLEAR | LOW | LOW |  |
|  | Assessment justification: | “The subjects were randomised into two groups according to gender, age and BMI: an intensively treated group (group 1) and a less intensively treated group (group 2.)”  No further information given. | NS | Weight objectively measured.  The laboratory tests were performed according to clinical routine. Blood glucose concentrations were determined by the glucose oxidase method.12 Serum insulin assays were performed by the Phadebas test (Pharmacia, Uppsala, Sweden).12 The blood pressure was measured in the right arm with a sphygmomanometer. The cuff size was 15.45 cm depending on the arm circumference. The recordings were made to the nearest 2 mmHg twice after 10 min supine rest, and the mean of the two measurements was used in the analyses. | Less intensive group: 19/21 at 6mths;  18/21 at 12mths;  15/21 at 24mths.  More Intensive group: 19/22 at 6mths;  17/22 at 12mths;  17/22 at 24mths. |  |
| **82** | **Menard 2005** | LOW | UNCLEAR | LOW | LOW | UNCLEAR |
|  |  | “Using a blocked randomization (n = 4) stratified by hemoglobin A1c value (< 10% and ≥ 10%), patients were assigned by an independent person using a computer program to receive intensive multitherapy or usual care.” | NS | “Fasting plasma glucose levels, hemoglobin A1c concentrations, blood pressure and serum lipoprotein levels were measured after a 12-hour, overnight fast at baseline and at 6, 12 and 18 months. Weight and height were measured, and body mass index (kg/m2) was calculated. Fasting plasma glucose levels were measured using a glucose oxidaze method. Cholesterol, high-density lipoprotein cholesterol, and triglyceride levels were measured using a colorimetric process (Johnson & Johnson Ortho-Clinical Diagnostics, Rochester, NY). Low-density lipoprotein cholesterol levels were calculated with the Friedewald formula. Hemoglobin A1c concentrations were measured by high-performance liquid chromatography (Bio-Rad VARIANT, Hercules, Calif.).” | Control:  35/36 at 12mths;  29/36 at 18mths.  intervention:  34/36 at 12mths; 32/36 at 18mths. | For ethical reasons, patients in the control group had protocol-driven laboratory tests, and they and their physicians received information about diabetes and its management as well as the results of these tests. Thus, control group patients may have received more aggressive treatment and attention than they normally would have. |
| **83** | **Mensinger 2016** | LOW | LOW | LOW | HIGH |  |
|  | Assessment justification: | “This study was a 1:1 parallel-group randomized design comparing the effectiveness of two 6-month group-based “healthy living programs” (weight-neutral or weight-loss). Folded index cards containing program assignments from a computer-generated randomization scheme were placed into sealed and sequentially numbered opaque envelopes.” | “Folded index cards containing program assignments from a computer-generated randomization scheme were placed into sealed and sequentially numbered opaque envelopes. Upon completion of the baseline assessments where informed consent was obtained, participants were given an envelope containing a welcome letter with their assignment and instructions.” | Weight measured objectively.  Waist and hip circumference was measured to the nearest quarter inch with a flexible tape measure on bare skin. Venous blood samples were drawn after an overnight fast in order to obtain glucose levels and lipid panels (total cholesterol, LDL-C, HDL-C, total cholesterol-HDL ratio, and triglycerides). We followed standardized methods established by the National High Blood Pressure Education Program and averaged two blood pressure (BP) readings using a Welch Allyn cuff with an aneroid sphygmomanometer. | Weight neutral -  39/40 at 6mths (LOW); 19/40 at 24mths (HIGH) weight loss -  33/40 at 6mths (LOW); 21/40 at 6mths (LOW).  Attrition rate at 6-month for the weight loss group > 25%, change from low to high. |  |
| **84** | **Messier 2013** | LOW | UNCLEAR | LOW | LOW |  |
|  | Assessment justification: | "A stratified-block randomization method was used to assign all eligible persons to 1 of the 3 intervention groups, stratified by BMI and sex." | NS | Weight, height, and BMI were obtained at baseline, 6months, and 18months using standard techniques. | "Of the 454 participants, 399 (88%) completed the study (returned for 18- month follow-up). Retention did not differ significantly among the groups (E, 89%; D, 85%; D+E, 89%)..." |  |
| **85** | **Miller 2002** | LOW | LOW | LOW | LOW |  |
|  | Assessment justification: | Randomization was stratified by race (African American and other), with an allocation ratio of 1:1 and a block size of 4. The order of randomization was constructed from a published list of random numbers; | assignments were issued by the study coordinator who opened sealed opaque envelopes that contained the group assignment. | Weight was measured biweekly by blinded personnel using a certified balance-beam scale. | Less than 50% attrition at 12-months follow-up. |  |
| **86** | **Mitsui 2008** | UNCLEAR | UNCLEAR | UNCLEAR | LOW |  |
|  | Assessment justification: | "...were randomly assigned to the intervention group..." | NS | NS | "Two participants in the intervention group and 1 in the control group dropped out of the program after week 12 for personal reasons." |  |
| **87** | **Morgan 2010** | LOW | LOW | LOW | LOW |  |
|  | Assessment justification: | "The random allocation sequence was generated by a computer-based random number-producing algorithm in block lengths of six to ensure an equal chance of allocation to each group." | "To ensure concealment, the sequence was generated by a statistician and given to the project manager. Randomization was completed by a research assistant who was not involved in the assessment of participants and the allocation sequence was concealed when enrolling participants." | "Weight was measured without shoes on a digital scale to 0.1kg (model CH-150 kp; A&D Mercury, Adelaide, Australia)"  Systolic and diastolic blood pressure and resting heart rate were measured using a NISSEI/DS-105E digital electronic blood pressure monitor (Nihon Seimitsu Sokki, Gunma, Japan) under standardized procedures. | Retention rate: 3-month follow-up: Control: 27/31*100 = 87.1%;  SHED-IT: 28/34*100 = 82.4%  6-month follow-up: Control: 26/31*100 = 83.9%;  SHED-IT: 28/34*100 = 82.4%  12-month follow-up: Control: 20/31*100 = 64.5%;  SHED-IT: 26/34*100 = 76.5% |  |
| **88** | **Muggia 2014** | LOW | LOW | UNCLEAR | HIGH |  |
|  | Assessment justification: | “Participants were randomly allocated (allocation ratio 1:1) to the standard care or cognitive behavioral therapy group, using a computer-generated randomization application of STATA statistical package.” | “Randomization list was kept at Biometric Unit and clinicians were unaware of the treatment group until the subjects were enrolled. The treatment allocation was communicated by phone to the clinician every time a new patient was enrolled.” | Information on how weight was measured not stated. | A high attrition rate is observed and although multiple imputations are performed to reduce its impact bias, this cannot be excluded as also reported in similar studies.  At the six month, 114 patients (69.9% of the total) attended the follow-up visit, with an attrition rate of 30.1%. The percentage of visits attended was significantly greater in group A (83.3% vs 70.4% in group B, p < 0.001). At the 12 month, 78 patients (47.8% of the total) attended the follow-up visit, with an attrition rate at 1 year of 47.8 per 100 person-year. A total of 44 patients in the treatment arm A (53.0%) and 34 (42.5%) in group B completed the follow-up, with no significant differences between the two groups. |  |
| **89** | **Munsch 2003** | UNCLEAR | UNCLEAR | UNCLEAR | HIGH |  |
|  | Assessment justification: | NS | NS | "During the investigation the BMI (kg/m2) was recorded at the start, end and 12 months after completion of treatment." | “The dropout rate until the end treatment was 23%, 29% and 37% in the GP BASEL, the GP control and in the Clinic BASEL groups, respectively. This resulted in a distorted ratio between BASEL treated and control subjects (ratio: almost 3:1; aimed ratio: 3:2). The dropout rate between the end of treatment and the 1–year follow up was 0%, 33% and 52% in the GP BASEL, the GP control and in the Clinic BASEL groups, respectively." |  |
| **90** | **Nakata 2014** | LOW | LOW | LOW | LOW | UNCLEAR |
|  | Assessment justification: | " After the motivational lecture, the participants were randomly assigned to one of the 3 groups using simple randomisation procedures involving computerised random numbers." | "The allocation data were generated by an investigator (MO) who had no contact with the participants or other staff members, and the data were maintained at a central secure location until completion of the motivational lecture." | "Data were collected at baseline and at months 3, 6, 18 and 30 in the hospital by trained hospital staff members who were blinded to the treatment assignment process. The primary outcome measure was the amount of weight lost from baseline to 30 months." | The attrition rates were 9.6% (12/125) and 20.0% (25/125) at months 18 and 30, respectively (Fig. 1). The numbers of individuals lost to follow-up at 30 months were similar in both groups (p = 0.531). | "Due to ethical concerns, we provided group-based support to the control group after the 6-month intervention period and did not include them in the follow-up measurements." |
| **91** | **Nanchahal 2012** | LOW | UNCLEAR | LOW | LOW |  |
|  | Assessment justification: | "Participants were randomly allocated (allocation ratio 1:1) to the control or intervention group (TP, EH, AS), using a computer-generated randomisation application written in VBA for MS Access (TP). The Taves method of minimisation 48 was used to ensure the groups were balanced for general practice, gender, age group (≤50/>50 years), BMI category (≤30/>30 kg/m2), diagnosis of diabetes (yes/no) and taking antipsychotic medication or not." | NS | The study was single blinded with members of the study team assessing baseline and follow-up measurements blinded to group assignment.  Weight (in light clothing) was measured using the Tanita (BC 420 MA) scales. The scales also reported per cent body fat, basal metabolic rate and metabolic age (age expected for a given value of basal metabolic rate). Waist was measured midway between the iliac crest and the costal margin to the nearest 0.1 cm. Blood pressure and heart rate were measured using a digital automatic monitor (Omron Model M10-IT), with the average of three readings recorded where possible. | Retention rate: 6-months (step change in intervention intensity): Control: 67.9%; CAMWEL: 70.2%  12-months:  Control: 60.0%; CAMWEL: 53.9% |  |
| **92** | **Ng 2015** | LOW | UNCLEAR | LOW | LOW |  |
|  | Assessment justification: | “Recruited patients were randomized in 1:1 ratio to participate in the LMP or usual care through the use of a computer-generated list of random numbers.” | NS | "Anthropometric measurements, ESS, and laboratory tests, which included liver and renal function, fasting glucose, and lipids, were performed at baseline, 4 months, and 12 months." | "Sixteen participants in the intervention group were excluded after randomization, as six had never attended dietician visits, seven attended fewer than four dietician visits, and three maintained their high-energy and -fat food intake. There were six subjects in the control group lost to follow-up."  LMP Group:  45/61*100 = 73.8%;  Control:  37/43*100 = 86% |  |
| **93** | **Nicklas 2004** | LOW | UNCLEAR | LOW | LOW |  |
|  | Assessment justification: | "A variable-block randomization method was used to assign all eligible persons to 1 of the 4 intervention arms, stratified by race (white versus nonwhite). A list of random assignments to the 4 groups was computer-generated within each stratum, with blocks of 4, 8, and 12 chosen with equal probability." | "Once a subject met the eligibility criteria, a computer program displayed the next group assignment and logged it into the database." | "Each subject’s weight (without shoes) was measured at baseline and at the 6-month and 18-month followup visits, using the same calibrated scale. Measurement at each session was scheduled for the same time of day." | "Of the 316 randomized participants, 252 (80%) completed the study (returned for the final data collection visit).""Retention of participants was not significantly different between the 4 groups (for healthy lifestyle, 86%; for diet only, 77%; for exercise only, 80%; for diet plus exercise, 76%)."Retention of participants was not significantly different between the 4 groups (for healthy lifestyle, 86%; for diet only, 77%; for exercise only, 80%; for diet plus exercise, 76%)" Attrition rate:  6 Month: Healthy lifestyle: 89.7%; Exercise only: 87.5%; Diet only: 87.8; Diet plus exercise: 82.9% 18 Month: Healthy lifestyle: 86%; Exercise only: 80%; Diet only: 77; Diet plus exercise: 76% |  |
| **94** | **Nilsen 2011** | LOW | LOW | LOW | LOW |  |
|  | Assessment justification: | Groups were randomly assigned to an “individual physician group” (IG) or an “individual plus interdisciplinary group” (IIG) by use of closed envelope method with unknown block sizes. | Closed envelope method. | “At every visit to the study physician, the following assessments were performed: fasting blood sample, systolic and diastolic blood pressure (SBP and DBP) according to recommended standards [18], waist circumference at a level midway between the lowest rib and the iliac crest to the nearest cm, height without shoes to the nearest cm (only first visit) and weight in indoor clothes to the nearest 100 g. Blood pressures were measured by an Omron M41 and weight with a Seca 771.” | Control IG: 89/104 at follow up Intervention IIG 93/109 |  |
| **95** | **Nordby 2012** | LOW | LOW | UNCLEAR | HIGH |  |
|  | Assessment justification: | "The randomization was performed over the three 6-mo blocks, using a manual lottery in which participants drew their own lot." | "Sixty participants were randomized, each participant drawing his own lot." | "Before, during, and after the intervention, participants underwent a panel of tests." | Dropout rates for each group post intervention were >20% for all groups. "Of the 36 participants who completed T, D or T-iD, 28 participated in follow-up visits: 9 participants in T (6 months: n = 7; 12 months: n = 8), 10 participants in D and 9 participants in T-iD)." Only 41% of participants in group T participated at the 6-month time point. |  |
| **96** | **Nurkkala 2015** | UNCLEAR | UNCLEAR | LOW | LOW |  |
|  | Assessment justification: | NS | NS | "All measurements were performed at baseline and at 9, 24 and 36 months. Body weight was measured to the nearest 0.1 kg using a calibrated scale (SOEHNLE S20, Soehnle waagen, Germany)." | "Fifty-nine participants (66%) in the intervention group and seventeen participants (57%) in the control group completed the study." |  |
| **97** | **Oldroyd 2006** | LOW | UNCLEAR | LOW | LOW |  |
|  | Assessment justification: | Eligible participants who agreed to take part were randomly allocated using a random number table to the intervention or control group at the first baseline appointment. | “Researchers performing the randomisation were blind to the group allocation.” | Weight was measured to the nearest 0.1 kg with the participants lightly clothed on SECA scales (Alpha Model 770 digital, SECA Limited, Birmingham, UK) | 6-month follow-up: Control: 32/39 * 100 = 82%; Intervention: 37/39*100 = 94.9%  12-month follow-up: Control: 30/39 * 100 = 77%; Intervention: 32/39*100 = 82%  24-month follow-up: Control: 24/39 * 100 = 62%; Intervention: 30/39*100 = 77% |  |
| **98** | **Pan 1997** | UNCLEAR | UNCLEAR | LOW | UNCLEAR | HIGH |
|  | Assessment justification: | NS | NS | "Briefly, blood pressure, height, and weight were measured in light clothing without shoes following methods used in the WHO multinational study of vascular disease in diabetes (17)." | NS | "The original 6-year study randomised to 4 groups (1. control, 2. diet, 3. exercise 4. diet and exercise). However, for the 20-, 23- and 30 year follow-ups, the authors combined the intervention groups and only reported data for the 'control' versus 'intervention' arms. Authors state that the reason for this was that: "As diabetes incidence did not differ significantly among the three intervention groups during the active intervention period and because of limited power to detect differences, the intervention groups were combined and comparisons were made between the combined intervention group and the control group." |
| **99** | **Parikh 2010** | UNCLEAR | UNCLEAR | LOW | LOW |  |
|  | Assessment justification: | "Participants were randomized to intervention or delayed intervention (in 1 year) by blocked randomization (block size=4) by recruitment site."  No further information given. | NS | Weight measured objectively. | "The study had some attrition: 83 participants returned at 3 months, 79 at 6 months, and 72 at 12 months (37 control, 35 intervention). Four participants became ineligible because of pregnancy. The 23 participants lost to follow-up at 12 months did not differ from those who returned for the final check-up in age, gender, weight, BMI, or family history of diabetes.” |  |
| **100** | **Patel 2016** | UNCLEAR | UNCLEAR | LOW | LOW |  |
|  | Assessment justification: | "We stratified the randomization, so that people with a BMI of 30–40 were randomly assigned separately from those with a BMI of greater than 40." | NS | "All participants received $25 for enrolling, $25 for the six-month weigh-in..." | Control: 100%; Standard: 96.1%; Immediate: 96%;  Daily lottery: 100% analysed. |  |
| **101** | **Pekkarinen 2015** | LOW | LOW | LOW | LOW |  |
|  | Assessment justification: | “A physician who had no contact with the patients carried out randomization using a computer-generated table of random numbers with block size of four and allocated participants.” | Refer to ‘Random sequence generation (selection bias)’. | “Weight was measured using a study purchased digital scale with an accuracy of 0.1 kg (Soehnle model 7307, Soehnle-Waagen GmbH & Co, Murrhardt, Germany) with light clothing and no shoes at baseline, at each session and at weeks 69 and 121.” | No maintenance group 69/100 at 17-weeks; 89/100 at 69-weeks; 75/100 at 121-weeks.  Maintenance group: 79/101 at 17-weeks; 68/101 at 12mths;  75/ 101 at 69-weeks; 68/101 at 121-weeks. |  |
| **102** | **Perri 1984** | UNCLEAR | UNCLEAR | LOW | LOW |  |
|  | Assessment justification: | Subjects were assigned randomly from stratified blocks of percentage overweight to one of six experimental conditions in a 3 X 2 factorial design. | NS | Non-behaviour therapy - 'participants were weighed in front of their group'; Behaviour therapy - 'therapists weighed participants in private' | 28/129 dropped out, attrition rate 22% |  |
| **103** | **Perri 2001** | UNCLEAR | UNCLEAR | UNCLEAR | UNCLEAR |  |
|  | Assessment justification: | The authors only mention that participants were assigned randomly to one of three conditions, however no information regarding the methods of randomization are provided. | NS | "The primary outcome measure was change in body weight assessed over the course of 17 months." | Total number of participants at baseline: 103; 88 completed the 5-month program, yielding an initial treatment completion rate of 85%. At the conclusion of initial treatment, the small number of male participants was unevenly distributed across conditions, and the data from these participants (n = 8) were excluded from further analysis. Thus, the study sample consisted of 80 women. 17 Month follow-up: BT = 15; RPT = 20; PST = 23 |  |
| **104** | **Perri 2014** | UNCLEAR | UNCLEAR | LOW | LOW |  |
|  | Assessment justification: | NS | NS | Weight was measured with a digital scale (Tanita Model BWB-800S, Arlington Heights, IL) at Months 0, 6, and 24. | Control:  162/169 at 6mths; 142/169 at 24mths.  Low dose:  138/148 at 6mths; 112/148 at 24mths.  Moderate dose: 129/134 at 6mths; 112/134 at 24mths.  High dose:  155/161 at 6mths; 126/161 at 24mths. |  |
| **105** | **Pettman 2009** | LOW | LOW | LOW | LOW |  |
|  | Assessment justification: | "Using a random number generator (MS Excel), participant data were then distributed into three groups of approximately equal numbers. Unidentifiable individuals were block-matched to achieve an even gender balance and distribution of MetS risk factors over the 3 groups by calculating means for waist, DBP and age together with counts of males and females for each group. The groups were checked for significant differences between variables using independent samples t-tests. The three groups were then randomly assigned to ‘A’, ‘B’ or ‘C’ corresponding to INT-A, INT-B or CON respectively.” | "Study personnel generating the sequence were not aware of participant details, due to obscuring of identification numbers. Final group assignment was conducted by an impartial person." | "Body weight was measured to the nearest 0.1 kg (Tanita Ultimate scales™ Model 2000, Tanita Corporation, Tokyo, Japan), except for individuals weighing over 150 kg, who were weighed on a single set of electronic glass scales (Model 3200, Propert Pty Ltd, Castle Hill, NSW, Australia). The same set of scales was used at subsequent measurements for each participant." | Retention rate: 4-months:  Control: 86%;  INT-A: 98%;  INT-B: 92.6%  12-months: Control: 36/43*100 83.7%;  INT-A: 44/48*100 = 91.6%;  INT-B: 35/49*100 = 92.6% |  |
| **106** | **Poelman 2015** | LOW | UNCLEAR | HIGH | LOW |  |
|  | Assessment justification: | “Randomization lists were generated with standard statistical computer software (IBM SPSS Statistics 20.0). Based on the randomization list, the researcher (M.P.P) allocated subjects to one of the groups.” | “Due to the nature of the intervention, it was not possible to blind participants to their allocated condition.” | At baseline (T0), weight was measured using two different scales: a professional one (the Marsden MPMS-250 digital scale, Oxfordshire, UK) and the participant’s scale, in light clothes and with shoes removed. Measurements were highly correlated (regression coefficient= 0.99; intercept=-0.10), indicating that both scales yield largely similar results. At T2, the weight was also objectively measured using the professional scale, during a home visit from the researchers. At T1 and T3, participants were asked again to weigh themselves.  T0 = Baseline T1 = 3-months T2 = 6-months T3 = 12-months  T1 (3-months) time point was intervention end and participants self-weighed. | Less than 50% at 12-month follow-up. Control 73.4% Intervention 64% at 12-month follow-up  At 3-months:  usual care: 111/139; intervention: 85/138  At 6-months: usual care: 118/139; intervention: 105/138  At 12-months: usual care: 102/139; intervention: 89/138 |  |
| **107** | **Promrat 2010** | LOW | LOW | LOW | LOW |  |
|  | Assessment justification: | "Randomization was performed using a random number generator developed by the project statistician, with a target enrollment of 30 participants." | "The randomization process was conducted by a project staff who was blinded to the randomization sequence." | "Data collection was obtained by trained staff who were not aware of the group assignment or sequence of measurement." | "Thirty participants (97%) completed the study. One participant (3%) in the lifestyle intervention group withdrew from the study after 3 months. All other participants adhered to the study protocol follow-up schedule." |  |
| **108** | **Provencher 2009** | UNCLEAR | UNCLEAR | LOW | LOW |  |
|  | Assessment justification: | "Randomization was performed within each phase, and women were then assigned to one of the 3 treatment conditions: HAES group (N = 48), SS group (N = 48), or control group (N = 48). | NS | "Height was measured to the nearest millimeter with a stadiometer, and body weight was measured to the nearest 0.1 kg on a calibrated balance. Participants were asked to dress lightly and to remove their shoes for these measurements." | Baseline (T=0):  Control: 46/48*100 = 95.8%;  SS Group: 46/48*100 = 95.8%;  HAES: 100%  4-months (T=4): Control: 38/48*100 = 79.2%;  SS Group: 39/48*100 = 81.3%;  HAES: 44/48*100 =91.7%  10-months (T=10): Control: 34/48*100 = 70.8%;  SS Group: 38/48*100 = 79.2%;  HAES: 45/48*100 =93.8%  16-months (T=16): Control: 32/48*100 = 66.7%;  SS Group: 33/48*100 = 78.8%;  HAES: 41/48*100 =85.4% |  |
| **109** | **Rejeski 2011** | LOW | UNCLEAR | LOW | LOW |  |
|  | Assessment justification: | “Each participant was randomized to treatment using a permuted block randomization scheme with stratification by wave within each county in an effort to minimize confounding between treatment and location.” | NS | Weight measured objectively. | Less than 50% attrition at 18-month follow-up. |  |
| **110** | **Ridgeway 1999** | UNCLEAR | UNCLEAR | LOW | LOW |  |
|  | Assessment justification: | NS | NS | "The patients were weighed..." | CG: 20/28 = 71%; IG: 18/28 = 64% |  |
| **111** | **Rolls 2005** | UNCLEAR | UNCLEAR | LOW | LOW |  |
|  | Assessment justification: | “…a stratified randomization scheme was used to balance the distribution of subject sex and age across the groups.” | NS | “Body weight was measured at each counseling session, with the subject wearing light clothing without shoes, using a scale that was regularly calibrated.” | Less than 50% attrition at 12-month follow-up. |  |
| **112** | **Rosas 2015** | LOW | UNCLEAR | LOW | LOW |  |
|  | Assessment justification: | Participants are randomized to one of three arms according to the ratio 1 UC: 2 CM: 2 CM+CHW. After all baseline data were collected, a blinded data analyst/biostatistician confirmed study data completion and randomizes the participant to one of the three arms in permuted blocks stratified by sex, BMI (30-34.9, 35-39.9, or ± 40), and diabetes status. | The data analyst/ biostatistician was blinded. | Data collection staff were blinded to treatment assignment.  Weight was measured at each assessment visit in duplicate using a Detecto scale, whereas height was measured in duplicate using a wall-mounted stadiometer at baseline only. Participants’ anthropometric measures were assessed without their shoes and coats. | As in other lifestyle intervention trials, all participants did not attend all planned intervention activities (one-on-one case management, groups sessions, and home visits). This limited our ability to test whether the planned intervention had the intended effect. Nevertheless, the percentage of participants attending each activity was within the expected range. Body weight was collected from 207 participants (100%) at baseline, followed by 190 (91.8%) at 6 months, 171 (82.6%) at 12 months, and 177 (85.5%) at 24 months. |  |
| **113** | **Ross 2012** | LOW | UNCLEAR | LOW | LOW |  |
|  | Assessment justification: | Eligible participants were randomized on the basis of a computer automated randomization sequence after the acquisition of primary outcome data. Randomization was stratified by sex, age, and WC measurement | NS | Weight objectively measured. | Of the 490 participants, 396 (80.8%) returned for follow-up testing at 24 months. |  |
| **114** | **Samaras 1997** | UNCLEAR | UNCLEAR | LOW | LOW | UNCLEAR |
|  | Assessment justification: | 'Subjects were randomised into two groups'  No further information given. | NS | All anthropometric measures were performed by a trained investigator (AMM). Body height was measured to the nearest cm using a stadiometer with the subject barefoot; body weight to the nearest 0.1 kg in light street clothing. B | Control:  13/13 at 12-months Intervention:  13/13 at 12-months  0% dropout by end of study | After the 6-month programme, the exercise sessions remained available to subjects in the intervention group. |
| **115** | **Santanasto 2011** | LOW | UNCLEAR | LOW | LOW |  |
|  | Assessment justification: | "Randomization was done using a Microsoft Access-based random-number generating algorithm with stratification by age and sex to further ensure balance between groups (Microsoft Redmond, Washington)." | NS | "At the baseline (BL) screening visit and followup visits, body height (cm) was measured using a wall-mounted stadiometer and body weight (kg)with a standard certified calibrated scale and were used to calculate BMI (weight (kg)/height (m2))." | "All participants, with the exception of one in the PA+SA group, were followed up to their 6FU visits." 12 Month follow-up: PA +LW = 18/21*100 = 85.7%; PA + SA = 93.3% |  |
| **116** | **Sattin 2016** | UNCLEAR | UNCLEAR | UNCLEAR | LOW |  |
|  | Assessment justification: | “Churches were recruited as pairs in the study based on congregation size. These pairs were included in six cohorts with each cohort including either two or four churches. Each church pair was then randomized to the Fit Body and Soul (FBAS) behavioral lifestyle intervention or Health Education (HE) comparison group.”  No further information given. | Allocation concealment through pastor but no further detail given. | NS | No attrition. |  |
| **117** | **Schubel 2016** | LOW | LOW | LOW | LOW |  |
|  | Assessment justification: | "They sequentially enter the study and are randomly allocated to the three dietary programs (ICR, CCR, or HD) by RANDI2 [9], a web-based software using a block size of six. Randomization is stratified by age (<50 years/ ≥ 50 years) and sex." | Refer to ‘Random sequence generation (selection bias)’. | "All outcome assessments (see Table 2) are performed by trained study personnel following standard operating procedures." | Overall, 144 participants (96.0%) completed the 12-wk intervention phase, 143 (95.3%) the 12-wk maintenance phase, and 136 (90.7%) the 26-wk follow-up phase (Figure 1). Across the entire study period of 50 wk there were 4 dropouts in the ICR (91.8%), 7 in the CCR (85.7%), and 2 (96.2%) in the Control group. |  |
| **118** | **Seligman 2011** | LOW | LOW | LOW | LOW |  |
|  | Assessment justification: | “Randomization was performed using a computer sequence with centrally concealed allocation.” | Refer to ‘Random sequence generation (selection bias)’. | “Body mass index was calculated as weight/height2 (kilograms per square meter). Waist was measured between the last rib and the iliac crest. Body fat mass was assessed with bioelectrical impedance (Omron HBF 306 Bioimpedance Analyzer).” | Less than 25% attrition at 12-months follow-up. |  |
| **119** | **Snel 2012** | UNCLEAR | UNCLEAR | LOW | LOW |  |
|  | Assessment justification: | NS | NS | "...patients visited the research center after an overnight fast. Height, weight and waist circumference were measured." | "All patients completed the whole study period of 18 months, there were no dropouts from the study." |  |
| **120** | **Solbrig 2018** | LOW | UNCLEAR | LOW | LOW | UNCLEAR |
|  | Assessment justification: | “Participants were randomized to MI or FIT by the lead researcher using https://www.randomizer.org/ (random pairs option).” | NS | In the two posttreatment assessment sessions, research assistants (RAs) who were blind to the intervention group, collected and recorded primary outcomes. RAs blind to intervention measured waist and weight, and participants completed process measures online. | Attrition: less than 25% of participants in each arm at 6-month (programmes' end) and 12month. | Both interventions were delivered individually by the lead author. Potential for contamination bias. |
| **121** | **Somers 2012** | LOW | LOW | LOW | LOW |  |
|  | Assessment justification: | "...a data technician unfamiliar with the research protocol used a random allocation computer software program to assign participants in blocks (minimum = 27, maximum = 39) to 1 of 4 treatment conditions." | Refer to ‘Random sequence generation (selection bias)’. | "Weight was measured to the nearest 0.1 kg without shoes in the standing position. " | "Seventy percent (n = 163) of all randomized participants completed the 2-year study..."; "Twenty-four participants (6 from PCST-only, 10 from BWM-only, 4 from PCST + BWM, 4 from standard care) dropped out of the study after randomization but before treatment..."; "Participant dropouts at other study intervals were as follows: 20 participants dropped out before the posttreatment assessment (6 from PCST-only, 3 from BWM-only, 5 from PCST + BWM, 6 from standard care); 15 participants dropped out before the 6-month follow-up assessment (5 from PCST-only, 2 from BWMonly, 4 from PCST + BWM only, 4 from standard care); and 10 participants dropped out before the 12-month posttreatment assessment (4 from PCST-only, 4 from BWM-only, 2 from PCST + BWM only, 0 from standard care)." |  |
| **122** | **Spring 2017** | LOW | LOW | LOW | HIGH |  |
|  | Assessment justification: | "Once all eligible participants of a cohort were assigned to a group, the three groups within each stratum were randomized by a statistician using a randomly permuted block with three cells." | "The statistician notified the project staff, who then revealed the treatment assignment (STND, TECH, or SELF) to participants during the first in-person group session." | "Body weight was measured without shoes on a calibrated balance beam scale at baseline and at 3, 6, and 12 months." | "Attrition at the final 12-month follow-up assessment was greater for SELF (25.0%) than either STND (12.5%) or TECH (3.1%)." 21.9% difference in attrition rate at 12 months follow-up. |  |
| **123** | **Stahre 2005** | UNCLEAR | UNCLEAR | LOW | LOW |  |
|  | Assessment justification: | NS | NS | “Weighing was always done without shoes and with light clothing using a calibrated scale. Those hospital personnel who were not participating in the study checked all the final weight measures.” | Control:  36/43 at 6mths; 33/43 at 12mths; 31/43 at 18mths.  Treatment: 57/57 at 10-weeks; 47/57 at 6mths,  40/57 at 12mths;  34/57 at 18mths. |  |
| **124** | **Stahre 2007** | UNCLEAR | UNCLEAR | LOW | HIGH |  |
|  | Assessment justification: | NS | NS | "The participants in the two programs were weighed and measured regarding height on the same occasions. Weighing of the participants was done using a calibrated scale with light clothing and without shoes. Two persons checked the measurements of the participants’ weight." | Cognitive program group: 16/27 = 59% retained and commenced treatment, 1 woman did not complete program.   Control group: 26/27 = 96% retained and commenced treatment, 6 women did not complete program.  24/54 (44%) participants followed up at 12 months. |  |
| **125** | **Stenius-Aarniala 2000** | LOW | UNCLEAR | UNCLEAR | LOW |  |
|  | Assessment justification: | Randomisation was by "shuffling cards", with the help of someone not involved in the study. | Refer to ‘Random sequence generation (selection bias)’. | NS | Control: 19/19 Treatment: 19/19 |  |
| **126** | **Stevens 1993** | UNCLEAR | LOW | LOW | LOW |  |
|  | Assessment justification: | "At clinics using the weight reduction intervention, randomization was conducted within high- and low-weight strata, with only high-weight participants eligible for the weight reduction group." | Centralized allocation by telephone; if not possible, sealed opaque envelopes. | "In addition, weights and blood pressures were recorded for all participants during official clinic visits 3, 6, 12, and 18 months after they entered the study." | 93% followed up at 12 months overall:  93% intervention; 93% control.  Reasons for attrition not reported. |  |
| **127** | **Stevens 2001** | UNCLEAR | LOW | LOW | LOW |  |
|  | Assessment justification: | NS | Centralized allocation via telephone to central randomizing centre or via sealed opaque envelopes. | "Blood pressure and weight were measured every 6 months after randomization to the end of follow-up at 36, 42, or 48 months, depending on randomization date. Clinic staff who were blinded to study group assignment made these assessments." | 92% followed up at 18 months overall: 92% intervention, 92% control.  Reasons for attrition not reported. |  |
| **128** | **Strobl 2013** | LOW | LOW | LOW | LOW |  |
|  | Assessment justification: | “The random sequence was generated at the University of Würzburg by staff not working at the rehabilitation clinic, using a computer program.” | “After having recruited a participant, clinic staff requested the randomization result from the scientific staff by phone (telephone randomization) thus guaranteeing concealment of randomization up to recruitment.” | “Body weight was assessed by both self-reports and physician measurements (at 12 months). Both assessments were highly concordant (intraclass correlation coefficient 0.99), with patients reporting slightly lower weight than did physicians (mean difference = –0.61, (standard deviation (SD) 1.88)). For the outcome analysis, the physician measurement of body weight was used whenever possible.” | Usual care:  203/239 at 6mths; 164/239 at 12mths  Intervention:  201/228 at 6mths; 177/228 at 12mths. |  |
| **129** | **Tapsell 2017** | LOW | LOW | LOW | HIGH |  |
|  | Assessment justification: | "Randomisation was conducted after the second screen for eligibility and performed remotely by an investigator unrelated to the clinic using a computer generated randomisation sequence (STATA V12, StataCorp LP, College Station, TX). The randomisation was stratified according to sex and BMI (low BMI: ≤30 and high BMI: >30). Randomisation was performed in randomly allocated blocks of 3, 6 or 9. " | "The randomisation list was provided to the study team who added eligible participants sequentially for each of the strata. The randomisation and participant database was only accessible by the HealthTrack study co-ordinator and administrator for security." | "Body weight (kg) was measured in an upright position in minimal clothing and without shoes using scales with a bio-electrical impedance component to also estimate body fat (%) (Tanita TBF-662, Wedderburn Pty Ltd., Ingleburn, NSW, Australia)." | "The intensive phase was completed by 298 participants (withdrawal rate 18%) and the 12 months follow-up by n=178 participants (withdrawal rate 39%)."  Total sample withdrawal rate at 12 months = 178/377*100 = 47% 12-month follow-up rate per group: Control: 61/126*100 = 48%;  Intervention: 45/120*100 = 36%; Intervention plus walnut: 72/126*100 = 57% |  |
| **130** | **TarragaMarcos 2017** | UNCLEAR | UNCLEAR | LOW | LOW |  |
|  | Assessment justification: | NS | NS | Weight objectively measured.   Blood pressure was measured using an automated and calibrated electronic device, according to the recommendations of the Spanish Society of Arterial Hypertension. | There were no dropouts in G1 or G2 during the follow-up period, however 4 patients left G3 for personal reasons, leaving this group with 55 patients. |  |
| **131** | **Teeriniemi 2018** | LOW | UNCLEAR | LOW | LOW |  |
|  | Assessment justification: | "MS Excel was used by an independent researcher to produce a randomization list with random permuted blocks of 24." | NS | Weight was measured by a study nurse. | "A total of 108 participants (20.3%) did not return to the study centre for the 1-year visit (Fig. 2), and 49 participants dropped out between the 1-year and 2-year visits. Thus, 375 study subjects completed the study per protocol, and the attrition rate at 24 months was 29.5% (n = 157). No statistically significant differences amongst the dropouts were found between the study arms..." |  |
| **132** | **ter Bogt 2009** | LOW | UNCLEAR | LOW | LOW |  |
|  | Assessment justification: | "... patients were allocated using computer-generated random numbers...". | NS | "Body weight was measured on an electronic scale with subjects wearing light clothing and no shoes..." | Low dropout rate after 1 year (9%). |  |
| **133** | **The Look AHEAD Research Group 2010** | LOW | LOW | LOW | LOW | HIGH |
|  | Assessment justification: | "Eligible participants are randomly assigned to either diabetes support and education or lifestyle intervention using a web-based data management system that verifies eligibility. Randomization is stratified by clinical center and blocked with random block sizes." (protocol) | "Eligible participants are randomly assigned to either diabetes support and education or lifestyle intervention using a web-based data management system that verifies eligibility. Randomization is stratified by clinical center and blocked with random block sizes." (protocol) | "Weight was measured in duplicate on a digital scale." | Retention rate:  Year 1:  DSE: 95.7%; ILI: 97.1%; Year 2:  DSE: 93.5%; ILI: 94.9%; Year 3:  DSE: 93.8%; ILI: 94.0%; Year 4:  DSE: 93.0%; ILI: 94.1%; Year 5:  DSE: 92.2%; ILI: 93.3%; Year 6:  DSE: 90.6%; ILI: 92.0%; Year 7:  DSE: 89.3%; ILI: 90.6%; Year 8:  DSE: 88.3%; ILI: 89.9%. | Participants in the intervention arm who, during the first 6 months, failed to lose 10% of their initial weight were offered a weight loss medication (orlistat). Those who lost <5% were encouraged by their lifestyle counselor to try pharmacotherapy, whereas those who lost 5.0% to 9.9% were informed of medication and could receive it on request. Medication was not offered to individuals who lost greater than or equal to 10% of initial weight and maintain the loss. 523 out of 2570 participants in the ILI study arm took Orlistat as part of the intervention. |
| **134** | **Trepanowski 2017** | LOW | UNCLEAR | LOW | LOW | HIGH |
|  | Assessment justification: | "Randomization was performed by a stratified random sampling procedure by sex, age (18-42 years and 43-65 years), and body mass index (25.0-32.5 and 32.6- 39.9). Block size ranged from 1 to 11 participants." | NS | "...body weight, which was measured monthly via a digital scale while the participant was in a hospital gown." | 69.0% of participants completed the study. "The dropout rate was highest in the alternate-day fasting group (13 of 34 [38%]), relative to the daily calorie restriction group (10 of 35 [29%]) and control group (8 of 31 [26%])." | "Participants in the control group were instructed to maintain their weight throughout the trial and not to change their eating or physical activity habits... Controls who completed the 12-month trial received 3 months of free weight-loss counseling and a 12-month gym membership at the end of the study." |
| **135** | **Tsai 2010** | UNCLEAR | LOW | LOW | LOW |  |
|  | Assessment justification: | Randomization was blocked in groups of six. | Sealed envelopes. | Weight was assessed by a research assistant (B.J.I.), who was not masked to treatment assignment. | Control: 24/26 at 6mths; 25/26 at 12mths Brief counselling: 21/24 at 6mths; 22/24 at 12mths |  |
| **136** | **Tuomilehto 2009** | LOW | LOW | LOW | LOW |  |
|  | Assessment justification: | “…the subjects were allocated randomly to two study groups by a study nurse according to a previously generated randomization plan. | Randomised by study nurse who did not take part in subsequent intervention. | The weight was measured at every visit. | Control: 10% drop out Intervention: 13% drop out |  |
| **137** | **van de Glind 2017** | LOW | LOW | LOW | LOW | HIGH |
|  | Assessment justification: | "The allocation sequence for each football club was generated by a computer programme written by a statistician not involved in the final analysis. The sequence was generated using randomised permuted blocks, stratified by club, with block lengths of 4 and 6, at random. The sequence was securely stored, with access restricted to those responsible for maintaining the randomisation system." | "Trial coordinators accessed randomisation allocation via a secure online portal." "It was not possible to mask participants or the fieldwork team to allocation, but the primary outcome measurements could not be accessed by either, and allocation was not known by study statisticians until after database lock." | "Body weight was measured using an electronic flat scale (Tanita HD366) with light clothing." | 91% and 92% of participants per group attended the post program follow-up time point; 88% and 92% attended the 12-month follow-up. | Wait-list control, no blinding. |
| **138** | **vanWier 2011** | LOW | LOW | LOW | LOW |  |
|  | Assessment justification: | After baseline measurements, the employee was randomised to one of the three study groups and either to a group receiving basic weight measurements (80% of each study group) or to a group receiving additional measurements (20% of each study group). This two-step randomisation meant that there were six groups an employee could be assigned to. Randomisation to these six groups was done by block randomisation, with each block containing 15 allocations. A computerized random number generator drew up an allocation schedule. | An administrative assistant put the group allocation in opaque sealed envelopes, numbered 1 to 1,500. These envelopes were taken to the locations of the baseline measurements and opened in the given order. The researchers were blinded for the allocation schedule, but were not blinded for allocation after randomisation. The participants were, in consequence of the nature of the intervention, not blinded for allocation after randomisation. Employees were not allowed to change groups after randomisation. | At baseline 'body weight and body height were assessed by the researchers.' 'Body weight and body height are assessed in all participants. Body weight is measured in kg, to the nearest 0.1 kg, with a digital scale (Seca 770; Seca GmbH & Co, Hamburg, Germany). Participants are wearing light clothing and no shoes. Body height is measured in m, to the nearest 0.001 m, with a portable stadiometer (Seca 214, Leicester Height Measure; Seca GmbH & Co, Hamburg, Germany). ' LOW In addition, in a questionnaire self-reported body weight is assessed. Participants are asked to weigh themselves wearing light clothing and no shoes. HIGH | At 24mths: Control 266/460 Internet 263/464 Phone 263/462 all <50% |  |
| **139** | **Vissers 2010** | UNCLEAR | UNCLEAR | LOW | LOW |  |
|  | Assessment justification: | NS | NS | Body weight was measured with a digital scale to the nearest 0.1 kg. | Less than 50% attrition at 12-month follow-up. |  |
| **140** | **von Gruenigen 2012** | UNCLEAR | UNCLEAR | LOW | LOW |  |
|  | Assessment justification: | "Randomization was stratified using block sizes of 6 or 8 by baseline BMI (25.0–39.9 versus >40)." | NS | "The RD weighed participants in private at the beginning of each session and weekly food/activity records were reviewed." | "Attrition in the trial overall was 21.3%. Six (14.6%) patients in the LI group versus 10 (29.4%) in UC did not complete the twelve-month assessments, p=0.159." |  |
| **141** | **vonGruenigen 2008** | LOW | UNCLEAR | LOW | LOW |  |
|  | Assessment justification: | “Participants were randomly assigned to LI or UC. Randomization was stratified according to patient BMI (25- 39.9 versus >40 kg/m2) using a stratified blocked randomization scheme in order to achieve comparability between the study groups based on BMI…” | NS | “Participants were weighed in street clothes without shoes on a Detecto hand rail scale (model #6855) and weight was recorded to the nearest 0.1 kg.” | At 12-months: Control: 18/22 Intervention: 17/23 |  |
| **142** | **West 2007** | UNCLEAR | LOW | LOW | LOW |  |
|  | Assessment justification: | NS | "women were randomized using a sequentially numbered, closed-envelope procedure." | "All assessments were conducted by trained interviewers blind to experimental condition. Body weight was measured without shoes using a calibrated balance beam scale." | Less than 20% and 50% of attrition at mid- and longer follow-ups. |  |
| **143** | **West 2011** | LOW | UNCLEAR | LOW | LOW |  |
|  | Assessment justification: | "Senior centers were randomized by computer-generated random numbers to either a Lifestyle weight-loss program or to a cognitive training program designed to serve as an attention control, matched in contact time, duration, and structure." | NS | "Body weight was measured in street clothes without shoes using a calibrated digital scale (Tanita BWB 800)..." | "Follow-up assessments were conducted with 211 older adults (93%) at 4 months."  4-months:  Intervention: 106/116*100 = 91.4%; Control:  96/112*100 = 85.7%  12-months:  overall retention rate was 86% and there was no difference between arms. |  |
| **144** | **Whelton 1998** | UNCLEAR | UNCLEAR | LOW | HIGH | HIGH |
|  | Assessment justification: | "Overweight participants are randomly assigned, in a 2 x 2 factorial design, to one of the following four groups..." "Using a computer program, each participant’s eligibility was confirmed prior to enrollment in the trial. Randomization was stratified by clinic and weight status to provide an even distribution of participants among the treatment groups at each site, and blocking of variable length (2, 4, and 8) was used to ensure temporal balance." | NS | "Detailed information was collected at baseline, and an interval medical history (including medication information and symptoms) and measurements of body weight and BP were obtained quarterly." | The protocol clearly states consistent follow-up for 36-months, however the primary paper describes an average follow-up for the 585 patients in the weight loss/no weight loss groups.  “Study data were collected at the 4 eligibility and randomization contacts and at quarterly visits during follow-up from August 1992 until December 1995. Follow-up ranged from 15 to 36 months (median, 29 months)." The number of participants followed up for each of the groups of interest at each time point in unclear. | Weight change data is only reported as two of the four groups, combined as follows: the non-weight loss group (Sodium Reduction and Usual Care) and the Weight loss group (Weight loss intervention and combined [sodium intervention and weight loss intervention). |
| **145** | **Wilson 2010** | LOW | UNCLEAR | UNCLEAR | HIGH |  |
|  | Assessment justification: | "Participants were randomly assigned to 1 of the 3 treatment conditions using a computer-generated sequence with stratification across treatments and within site based on high and low negative affect assessed by the Beck Depression Inventory (BDI)25 with a cutoff point of 18." | NS | "Assessors were blinded to treatment condition", not clear if weight was self-reported. | "At posttreatment, dropout rates were 7%, 28%, and 30% for the IPT, BWL, and CBTgsh groups, respectively. Interpersonal psychotherapy had a significantly lower attrition rate than either BWL or CBTgsh (F1,193=8.3; P.001)." At end of treatment 79.7% from BWL group compared to 90.7% from the IPT group completed assessments. |  |
| **146** | **Wilson 2016** | UNCLEAR | UNCLEAR | LOW | LOW | UNCLEAR |
|  | Assessment justification: | Randomization of worksites into conditions.  No further information given. | NS | Weight objectively measured. | Control: 147/234; Phone 165/233;  Group 106/182  “drop out of approximately 40% of enrolled participants” | No indication that results were adjusted for clustering, and one worksite per condition |
| **147** | **Wilson 2016b** | UNCLEAR | UNCLEAR | LOW | HIGH | HIGH |
|  | Assessment justification: | “Six sites were matched based on the number of employees and randomly assigned to treatment or control groups.' 'Randomization of worksites into intervention or control groups” | NS | Weight objectively measured.  Self-reported secondary outcomes (e.g. food intake and physical activity). | 199 participants in the intervention sites and 46 participants at the control sites did not complete additional measures (post-test or follow-up) and were excluded from the ﬁnal analyses. At the post-test, 236 participated in the intervention (39 who joined after baseline) and 359 participated in the control (52 who joined after baseline). At follow-up, 136 participants in the intervention group and 211 in the control group completed the surveys and measures. Removing the one control group from the ﬁnal analysis because of contamination resulted in 227 (49.5%) participants in the ﬁnal intervention and 135 (69.9%) in the ﬁnal control cohort for outcome analyses.  “LGM analyses controlled for group differences by examining change over time and maximized the number of participants in the ﬁnal cohort.”  “LGM analyses that controlled for group differences by examining change over time and maximized the number of participants in the ﬁnal cohort. “ “Limitations of the study included ...dropout of approximately half of the participants from the study; ... LGM analyses, which enable imputation of data based on two data points “ | 'An additional 39 employees at the intervention sites and 76 employees from the control sites joined the study prior to the post-test, resulting in 236 and 359 participants respectively at post-test.'? 'Need to include all interested participants regardless of risk status, which likely diluted the impact.'  '204 participants were excluded from analysis due to site deviation from protocol.' ('One control group experienced what Cook and Campbell referred to as compensatory rivalry. Contrary to study protocols, the site coordinator initiated a variety of intervention strategies (i.e., biggest loser contest, motivational interviewing sessions, group educational sessions) to ‘‘make their site look better,’’ according to an interview that was conducted with the site coordinator. This created a threat to the internal validity of the study, and as a result the site was removed from the ﬁnal analyses. It could not be included as part of the treatment condition, as the strategies used differed from the planned intervention.') |
| **148** | **Wing 1998** | UNCLEAR | UNCLEAR | LOW | LOW |  |
|  | Assessment justification: | NS | NS | Weight objectively measured. | Less than 50% attrition at 12-month follow-up. |  |
| **149** | **Yannakoulia 2008** | UNCLEAR | UNCLEAR | UNCLEAR | HIGH | UNCLEAR |
|  | Assessment justification: | NS | NS | NS | Fifty percent of patients were dropouts. Comparisons between completers and dropouts revealed no statistically significant differences between the two groups (with regards to history of diabetes, sex, HbA1c, BMI or waist circumference), apart from their age, with those not completing the intervention being younger compared to completers (53 ± 9 vs. 60 ± 9 yr, p = 0.05) (Table 2). A trend for an association between group and dropout was observed: 66.7% in the UC and 33.3% in the IC were dropouts (p = 0.07).  To explore the effect of several factors in relation to the likelihood of being a dropout, a logistic regression was performed. Older people (p = 0.03) and those with newly diagnosed T2DM (p = 0.05) were more likely to complete the program, whereas a tendency for a negative association between attendance of the IC group and the likelihood of dropping out was found (p = 0.08). | "They were also informed about smoking risks and encouraged to stop or limit smoking" but no information on how many smoked and if smoking behaviour changed and no other mentions of smoking in the paper. This could be a potential confounder but without this information difficult to assess. |
| **150** | **Yardley 2014** | LOW | LOW | HIGH | LOW | HIGH |
|  | Assessment justification: | “Participants were then automatically randomised to one of the four groups by a computer algorithm that employed stratification by waist (allocating to the lower weight group if waist < 88 cm for women, < 102 cm for men), and a block size of 60 within each practice.” | “The computer system immediately informed participants which group they had been allocated to, and sent an email to inform the practice nurse.” | Intention was to weigh all participants in practice but due to low levels of attendance, self-report measures were completed at 12 months by a little less than half the sample. | Majority of participants followed-up at 12m | Two practices deviated from protocol by providing considerable weight management support to their usual care patients. Having detected substantial deviations from trial protocol in two practices, these analyses were repeated for the three practices that had followed the protocol correctly. We therefore carried out additional analyses of outcomes in the three practices that had followed protocol by not offering additional nurse support to those in the usual care group (see per protocol analyses below). Even in the per protocol practices, the level of nurse contact was somewhat less than intended, especially in the regular nurse support group, and the level of phone and email contact was very low. There was a skewed distribution of nurse support. Using our revised follow-up procedures, the follow-up for the primary outcome was increased slightly to 68.7% at 12 months, but the proportion having blood tests dropped to 36.9%. |
| **151** | **Yates 2009** | LOW | LOW | LOW | LOW |  |
|  | Assessment justification: | “Participants were randomly assigned, using a block design, to receive either usual care, the PREPARE program, or the PREPARE program without pedometer use and were stratified by age and sex.” | “Participant random assignment was conducted using opaque envelopes and a randomly generated number sequence by a member of our research team with no prior knowledge of recruited individuals other than their age and sex.” | Weight measured objectively.  Waist circumference: midpoint between the lower costal margin and iliac crest;  Height: measured to the nearest 0.1 kg and 0.5cm, respectively. | Less than 50% attrition at 12-month follow-up. |  |
| **152** | **Yates 2018** | UNCLEAR | UNCLEAR | LOW | HIGH |  |
|  | Assessment justification: | NS | NS | Weight objectively measured. | 7/8 no lifestyle and 4/7 lifestyle followed up at one year. |  |
| **153** | **Yeh 2016** | UNCLEAR | UNCLEAR | UNCLEAR | LOW |  |
|  | Assessment justification: | NS | NS | "Anthropometric measures and fasting blood specimens were obtained at baseline, 6 months and 12 months to evaluate weight and cardiometabolic changes." | Attrition was less than 50% follow-up and there was a <20% difference in follow-up between groups at 6m. |  |
| **154** | **Yin 2018** | LOW | UNCLEAR | LOW | LOW |  |
|  | Assessment justification: | Participants were randomised in blocks of 10, using a randomization table by the study statistician. Enrollment and randomization were performed by trained research staff. | NS | Trained research staff measured the participant’s weight, height and waist circumference with light clothes twice and the average was used. Participant’s weight was recorded at each meeting. | Less than 50% of attrition (at 12 months, 19 int. and 5 cont. loss-to-follow-up) |  |
| **155** | **Zhang 2016** | LOW | UNCLEAR | LOW | LOW |  |
|  | Assessment justification: | The randomization schedules were generated using SAS PROC PLAN in SAS statistical software (SAS Institute Inc) and concealed until an eligible participant was ready for enrollment. | NS | Weight measured objectively. | Of 220, 211 (95.9%) completed the 6-month follow-up visit, and 208 (94.5%) completed the 12-month follow-up visit. ITT was followed, undertaking MCMC imputation method. |  |
| **BP:** Blood pressure; **HDL-C:** High density lipoprotein cholesterol; **Mths:** Months; **NS:** Not specified; **PR**: Pulse rate; **RCT**: Randomised controlled trial; **Wk/s**: week/s; **Yr.:** Year; **Yrs:** Years. | | | | | | |

### Table 5. Characteristics of included studies

| **Study ID** | **Country:** | **Follow-up time points (months):** | **Population** | **Study groups and number of participants randomised** | **Outcome measures extracted*** | **Overall Risk of Bias** | **Author contacted** | **Additional information obtained from author^#^:** | **Notes:** |
| --- | --- | --- | --- | --- | --- | --- | --- | --- | --- |
| Abed 2013 | Australia | 3, 6, 9, 12, 15 | Patients with symptomatic atrial fibrillation | Control = 75  Weight management = 75 | Weight; TC; HDL; SBP; FBG; Plasma insulin | High | Y | N |  |
| Ackermann 2011 | USA | 6, 14 | DPP population | Standard advice alone (controls) = 46  YMCA DPP intervention = 46 | Weight; TC; HDL; SBP; HBa1C | Unclear | N | N/A |  |
| Agras 1990 | USA | 3, 6, 12 | Overweight women without additional psychological disorders | Computer alone = 30  Computer + group support = 30 Behaviour therapy = 30 | Weight | Unclear | N | N/A |  |
| Ahern 2017 | UK | 3, 12, 24 | Adults with a BMI ≥ 28 | Brief intervention = 211  12-week behavioural weight-loss programme = 530  52-week behavioural weight-loss programme = 528 | Weight; TC; SBP; HBa1C; QoL | Low | N | N/A |  |
| Almanza -Aguilera 2018 | Spain | 3, 12 | Metabolically healthy obese women (definition based on the general criteria proposed by the International Diabetes Federation (IDF)) | Control (general recommendations) = 48  Treatment (lifestyle weight loss intervention) = 67 | Weight; TC; HDL; SBP; FG | High | Y | Y | Information provided. |
| Anderson 2014 | Scotland | 3, 12 | Overweight or obese adults (aged 50 to 74 years) who had undergone colonoscopy after a positive faecal occult blood test result, as part of the national bowel screening programme, and had a diagnosis of adenoma confirmed by histopathology. | Control (weight loss booklet only) = 166  Intervention (BeWEL) = 163 | Weight; TC; HDL; SBP; HBa1C; HOMA-IR; FG; Plasma insulin | Low | N | N/A |  |
| Appel 2011 | USA | 6, 12, 24 | Adults who were at least 21 years of age with obesity and had one or more cardiovascular risk factors (hypertension, hypercholesterolemia, or diabetes). | Control (Self-directed) = 138 Remote Support Only (N/A) = 139 In-Person Support = 138 | Weight; TC; SBP; FG; QoL | Low | N | N/A | Information available from previous reviews. |
| Ard 2004 | USA | 6, 18 | The target population consisted of generally healthy adults with above optimal BP including individuals with stage 1 hypertension who met Joint National Committee on Detection, Evaluation,  and Treatment of High Blood Pressure (JNC-VI) criteria for at least a 6-month trial of nonpharmacological therapy. | “advice only” comparison group = 273 “established” behavioural intervention group = 268  Established + DASH Intervention Group = 269 | Weight; TC; SBP; FBG; Plasma insulin; QoL; Incidence HTN; Remission HTN | Low | N | N/A | Information available from previous reviews. |
| Ard 2018 | USA | 6, 12 | General population of adults aged 65 and older who were at risk for cardiometabolic disease due to obesity and associated risk factors | Exercise Only = 54  Exercise + Diet Quality + Weight Maintenance = 55  Exercise + Diet Quality + Weight Loss = 55 | Weight; TC; HDL; SBP; FG; QoL; Incidence CV morbidity | Low | N | N/A | Information available from previous reviews. |
| Ash 2006 | Australia | 3, 6, 12 | General population and hospital referrals (one public hospital and one private hospital) overweight and obese | Control Group - Booklet only = 63 Individualised Dietetic Treatment = 66  Fat Booters Incorporated = 62 | Weight | High | N | N/A |  |
| Aveyard 2016 | England | 3, 12 | General population | Advice only = 942  Advice plus weight loss programme = 940 | Weight | Low | N | N/A |  |
| Azar 2013 | USA | 3, 6, 15, 24 | Pre-diabetes and/or metabolic syndrome | Control, Usual care = NS Self-directed = NS Coach-led = NS | Weight; TC; SBP; FG | Low | Y | N |  |
| Bacon 2002 | USA | 3, 6, 12, 24 | Women from the general population | Health at Every Size – control = NS  Diet Group – intervention = NS | Weight; TC; HDL; SBP; QoL | Unclear | N | N/A | Information available from previous reviews. |
| Barnes 2017 | USA | 3, 6, 15 | Overweight and obese with or without binge eating | Treatment as usual (N/A) = 30  Nutrition - ATTENTION CONTROL = 29 Motivational interviewing = 30 | Weight; TC; HDL; SBP; HBa1c; FG | Unclear | Y | Y | Information provided. |
| Bartels 2015 | USA | 3, 6, 9, 12, 18 | People with serious mental illness | Control, Fitness club membership = 106 IN SHAPE = 104 | Weight; TC; HDL; SBP | Unclear | N | N/A | Information available from previous reviews. |
| Beavers 2017 | USA | 6, 18 | Community-dwelling men and women 60–79 years of age | Weight loss = 82  Weight loss + Aerobic training = 86 Weight loss + Resistance training = 81 | Weight; QoL | Unclear | Y | Y | Author confirmed that they could not provide additional data. |
| Bennett 2012 | USA | 6, 12, 18, 24 | Obese patients receiving hypertensive treatment | Control, Usual care = 185 Be Fit, Be Well = 180 | Weight; SBP, QoL | Unclear | Y | Y | Data provided; Information available from previous reviews. |
| Bennett 2013 | USA | 6, 12, 18 | General population | Control, usual care = 97  Weight gain prevention intervention = 97 | Weight; TC; HDL; SBP; FG; QoL | Unclear | Y | Y | Data provided. |
| Berry 2014 | USA | 3, 12, 18 | Parent and child dyad with overweight or obesity | Control = 162  Family based. Nutrition, exercise and coping skills intervention = 184 | Weight; QoL | High | Y | Y | Data provided; Information available from previous reviews. |
| Bertram 1990 | South Africa | 4, 16 | General population | Control - diet only = 15  Diet plus lectures = 15  Diet plus exercise = 15 | Weight; Incidence CV mortality | High |  |  |  |
| Bertz 2012 | Sweden | 3, 12 | Women, 8-12-week post-partum | Control = 17  Diet Only = 17 Exercise only = 18 Intervention = 16 | Weight; TC; SBP; FG; QoL | Unclear |  |  | Information available from previous reviews. |
| Bo 2007 | Italy | 12, 24 | General population (70-72% with metabolic syndrome) | Control standard care = 188 Intervention lifestyle by trained professional = 187 | Weight; TC; HDL; SBP; FG | Low | N | N/A |  |
| Brown 2014 | USA | 3, 6, 12 | Clients at one of four community mental health programs, three in the Kansas City area and one in Las Vegas | Control = 66  RENEW = 70 | Weight | Unclear | N | N/A |  |
| Burke 2005 | Australia | 4, 16, 40 | Hypertensive patients | Control usual care = 118  Low sodium + fish diet = 123 | Weight; TC; HDL; SBP; HOMA-IR; FBG; Plasma insulin; QoL; Remission HTN | Unclear | Y | Y | Information provided. |
| Burke 2015 | USA | 6, 12, 18 | General population | Standard behavioural weight loss treatment = 72 Self-efficacy enhancement plus standard behavioural weight loss treatment = 58 | Weight; QoL | Unclear | N | N/A |  |
| Cheyette 2007 | UK | 4, 6, 12 | Patients with type 2 diabetes on insulin treatment | Control = 20 Weight No More intervention group = 29 | Weight; HbA1c; QoL | Unclear | N | N |  |
| Christensen 2012 | Denmark | 3, 12 | Female overweight healthcare workers | Reference group = 44 Intervention group = 54 | Weight; SBP | Low | N | N/A |  |
| Cleo 2018 | Australia | 3, 12 | General population | Wait list control (N/A) = 25  TTT Top Ten Tips habit formation = 25 DSD Do Something Different online software = 25 | Weight | Unclear | N | N/A |  |
| Conroy 2015 | USA | 3, 12 | General population | Self-guided = 50 Interventionist led = 49 | Weight; SBP | Unclear | N | N/A |  |
| Cooper 2010 | UK | 6, 10, 16, 22, 34, 46 | General population | Guided Self-Help Control = 51 Behaviour Therapy = 50 Cognitive Behaviour Therapy = 49 | Weight | Low | N | N/A |  |
| Cousins 1992 | USA | 3, 6, 12 | General population | Control = 56 Individual = 56 Family = 56 | Weight | Unclear | N | N/A |  |
| Craighead 1989 | USA | 3, 12 | General population | Control, minimal contact = 20 Contracted Exercise = 20 Supervised Exercise = 22 | Weight | Unclear | N | N/A |  |
| Dale 2009 | New Zealand | 4, 8, 12, 24 | Insulin resistant adults | Control = 23 Modest = 31 Intensive intervention = 25 | Weight | Unclear | Y | N | Information available from previous reviews. |
| Dalziel 2006 | France | 2, 12, 48 | Patients who had experienced their first myocardial infarction. | Control = 303  Experimental = 302 | Weight; TC; HDL; SBP; Final follow-up only: Incidence CV morbidity; Incidence CV mortality | High | N | N/A |  |
| Damschroder 2014 | USA | 3, 12, 18, 24 | Veterans | Control, MOVE - usual care = 159  ASPIRE group, individual telephone counselling = 162  ASPIRE group, group counselling = 160 | Weight; HDL; SBP; HbA1c; QoL | Unclear | N | N/A | Information available from previous reviews. |
| Daumit 2013 | USA | 6, 12, 18 | Psychiatric patients | Control, Usual care = 147 ACHIEVE = 144 | Weight; TC; HDL; SBP; FG; QoL; One timepoint only: Plasma insulin; Incidence CV morbidity; Incidence T2DM | Unclear | N | N/A | Information available from previous reviews. |
| deVos 2016 | Netherlands | 6, 12, 18, 24, 30, 80 | Females 50 to 60 years | Control = 204 Tailor-made lifestyle intervention = 203 | Weight; TC; HbA1c; QoL | Unclear | Y | Y | Data provided. |
| Diabetes Prevention Program R G 2009 | USA | 6, 12, 18, 24, 30, 36, 42, 48, 54, 60, 66, 72, 78, 84, 90, 96, 102, 108, 114, 120, 126, 132, 138, 144, 150, 156, 162, 168, 174, 180, 186 | People at high risk for type 2 diabetes (impaired glucose tolerance) | Placebo = 1082 Metformin (N/A) = 1073  Lifestyle = 1079 | Weight; HDL; SBP; HbA1c; FG; QoL; Incidence T2DM | High |  |  | Information available from previous reviews. |
| Djuric 2002 | USA | 3, 6, 12 | Women with stage I or II breast cancer diagnosed within the past 4 years and free of any recurrence. | Control = 13 Weight Watchers = 11  Individualized group = 13 Comprehensive group = 11 | Weight; TC:HDL ratio; TC; HDL; FG; Plasma insulin | High | Y | N | Information available from previous reviews. |
| Duncan 2016 | New Zealand | 4, 12 | Primary health care patients with an elevated 5-year cardiovascular disease risk | Control = 162  Intervention = 158 | Weight; TC:HDL ratio; TC; HDL; SBP. | High | N | N/A |  |
| Eakin 2014 | Australia | 6, 18, 24 | Patients 20–75 years with type 2 diabetes | Usual care = 151  Telephone intervention = 151 | Weight; TC:HDL ratio; TC; HDL; SBP; HbA1C | Low | Y | Y | Data provided |
| Eaton 2016 | United States | 6, 12, 18, 24 | General population | Control, Standard Intervention = 106  Enhanced Intervention = 105 | Weight | Low | N | N/A | Information available from previous reviews. |
| Fernandez-Ruiz 2018 | Spain | 6, 12, 24 | General population (Community Care Centre population (health centre patients)) | Control = 37  Intervention (healthy eating, exercise & CBT) = 37 | Weight; TC; SBP; HbA1C; QoL; Incidence CV mortality | Unclear | N | N/A |  |
| Foley 2016 | USA | 6, 12 | Obese (BMI: 30.0-44.9 kg/m2) community health center patients with a diagnosis of hypertension, diabetes and/or hyperlipidemia | Usual care (Control) = 175  Weight loss intervention = 176 | Weight; TC; HDL; SBP; HbA1c; FG; QoL | Unclear | Y | Y | Data provided. |
| Foster-Schubert 2012 | USA | 6, 12 | Post-menopausal women | Control- usual care = 87  Calorie reduced diet = 118  Aerobic exercise (N/A) = 117  Intervention - diet and exercise = 117 | Weight; HOMA-IR; FG; Plasma insulin; QoL | Unclear | N | N/A |  |
| Fuller 2012 | Australia | 1, 2, 3, 6, 9, 12 | Male or female residents of inner western Sydney aged 18—65 years, with a BMI of  25—45 kg/m^2^ | Western diet group = 35  Korean diet group = 35 | Weight; TC; HDL; SBP; FG; One timepoint only: Plasma Insulin | Unclear | Y | N |  |
| Goodwin 2014 | Canada; USA | 6, 12, 18, 24 | Postmenopausal women diagnosed with T1-3N0-3M0 breast cancer | Mailed-based intervention = 167 Individual lifestyle intervention = 171 | Weight; QoL | Unclear | Y | N | Author response received. |
| Green 2015 | USA | 6, 12, 24 | People taking antipsychotic medications | Usual care = 96 STRIDE = 104 | Weight; HDL; SBP; FBG; QoL | Unclear | Y | N | Information available from previous reviews. |
| Grilo 2011 | USA | 5.5, 11.5, 17.5 | Adults up to 60 years of age who meet full DSM–IV research criteria for BED | Cognitive Behavioral Therapy (CBT) = 45 Behavioral weight loss (BWL) = 45 CBT + BWL (N/A) = 35 | Weight | Unclear | Y | Y | Information provided. |
| Grilo 2014 | USA | 4, 10, 16 | Patients who were obese and met DSM-5 criteria for BED | Placebo = 27 Placebo/CBTsh = 25 Sibutramine (N/A) = 26 Sibutramine/CBTsh (N/A) = 26 | Weight | Low | N | N/A |  |
| Hanson 1976 | USA | 2.5, 5, 12 | General overweight and obese population. | No treatment control condition (N/A) = 10  Attention-placebo control condition = 11  Conventional self-management condition = 7 Programmed text with low therapist-group contact = 12 Programmed text with high therapist-group contact = 13 | Weight | High | N | N/A |  |
| Hardcastle 2013 | UK | 6, 18 | Primary care patients | Control = 131 MI counselling intervention = 203 | Weight; TC; HDL; SBP | Unclear | N | N/A |  |
| Harrigan 2016 | USA | 6, 12 | Breast cancer survivors | Usual Care Group = 33 Telephone Weight Loss Counseling = 34 In-Person Weight Loss Counseling = 33 | Weight; FG | High | Y | Y | Data provided. |
| Harris 2017 | UK | 6, 12 | Adults with an intellectual disability | Waist Winners Too = 24  TAKE 5 = 26 | Weight; QoL | Low | N | N/A |  |
| Hunt 2014 | UK | 3, 12 | Male football fans | Control, Wait-list = 373 FFIT = 374 | Weight; SBP; QoL | High | N | N/A | Information available from previous reviews. |
| Huseinovic 2016 | Sweden | 3, 12, 24 | Women 6–15 week postpartum | Control Group = 56  Diet behaviour modification Group = 54 | Weight; QoL | Low | N | N/A |  |
| Irwin 2003 | USA | 3, 12 | General postmenopausal female population | Control Group = 86  Exercise group = 87 | Weight; HOMA-IR; FG | Low | N | N/A | Information available from previous reviews. |
| Jackson 1982 | Australia | 1, 4, 7, 10, 16 | Females with intellectual disabilities ("mentally retarded females"). | Control = 6  Treatment = 6 | Weight | Unclear | N | N/A |  |
| Jakicic 2011 | USA | 6, 12, 18 | Overweight, sedentary adults | Self Help Group = 89 Moderate Physical Activity = 82 High Physical Activity = 98 | Weight | Unclear | N | N/A |  |
| Jebb 2011 | Australia, Germany, UK | 2, 4, 6, 9, 12, 18, 24 | Adults with a BMI 27-35 and at least one additional risk factor for obesity-related disease | Standard care = 395 Commercial programme = 377 | Weight; TC:HDL ratio; TC; SBP; FG; Incidence T2DM; Remission T2DM | Low | Y | Y | Data provided; Information available from previous reviews. |
| Jebb 2017 | UK | 3, 6, 12, 36 | Obese adults seeking support to lose weight | Usual care = 140 Low energy total diet replacement programme = 138 | Weight; TC; HDL; SBP; HbA1c; FG; Plasma insulin; QoL; One timepoint only: HOMA-IR | Low | N | N/A |  |
| Jeffery 1995 | USA | 6, 12, 18, 30 | Adults 25-45 years with overweight or obesity. | Control group = 40 Standard Behavioural Therapy (SBT) = 40 SBT + Incentives (I) = 41 SBT + Food Provision (FP) = 40 SBT + FP + I = 41 | Weight | Unclear | N | N/A | Information available from previous reviews. |
| Jenkins 2017 | Canada | 6, 18 | General population in the city of Toronto | Control = 486 Dietary advice only = 145 Food basket only = 148 Food and advice = 140 | Weight; TC:HDL ratio; SBP; FG | High | N | N/A |  |
| John 2011 | USA | 7.4, 17 | Adults 30 to 70 years of age with obesity | Control = 22 Deposit contracts group = 44 | Weight | High | Y | N |  |
| Jolly 2011 | UK | 3, 12 | Obese or overweight men and women with a comorbid disorder identified from general practice records, with a raised BMI recorded within their primary care notes within the previous 15 months. The BMI threshold for invitation is that which makes them eligible for primary care obesity management services within the NHS and varies according to ethnic group and the presence or absence of comorbidities. | Minimal intervention comparator = 100 Choice (N/A) = 100  Pharmacy =70  General practice = 70 Weight Watchers = 100 NHS Size Down = 100 Rosemary Conley = 100  Slimming world = 100 | Weight | High | N | N/A | Information available from previous reviews. |
| Jones 1986 | UK | 4, 16 | Female adults | Individual = 21 Group = 17 Leaflet Individual = 22  Leaflet Group = 20  Diary Individual = 20 Diary Group = 19 Leaflet Diary Individual = 21  Leaflet Diary Group = 20 | Weight | High | N | N/A |  |
| Jones 1999 | USA | 3, 6, 12, 18, 24, 30 | Patients with hypertension above the age of 50 | Control Group = NS  Weight Loss Group = NS | Weight | Unclear | Y | Y | Information provided. |
| Katzer 2008 | New Zealand | 2.3, 6.3, 14.3, 26.3 | Women with at least one other cardiovascular risk factor. | Mail-delivered 'non-dieting' program (P3) = 101  Group 'non-dieting' program (P2) = 62 Group 'non-dieting' program plus Relaxation (P1) = 62 | Weight; SBP | Unclear | N | N/A |  |
| Kuller 2012 | USA | 6, 18, 30, 48 | Postmenopausal females | Control - health education = 255  Intervention - lifestyle change = 253 | Weight; HDL; SBP; FG | Low | Y | Y | Information provided. Information available from previous reviews. |
| Leahey 2014 | USA | 3, 6, 12 | General population | SURI alone = 46 SURI plus Internet behavioral weight loss program = 90  SURI plus Internet behavioral weight loss program plus optional group sessions = 94 | Weight | Unclear | N | N/A |  |
| Ley 2004 | New Zealand | 6, 12, 24, 36, 60 | Workers with impaired glucose tolerance ((2 h blood glucose 7.8–11.0 mmol/l) and a further 114 (2%) had high normal blood glucose concentrations (7.0–7.8 mol/l)) | Control diet = 70  Reduced-fat = 66 | Weight; TC/HDL ratio; TC; HDL; SBP | High | N | N/A |  |
| Li 2016 | China | 1, 12 | Adults with Type 2 Diabetes Mellitus who are overweight (BMI ≥ 24 kg/m2) | Usual care group = 60  Diet group = 79  50g-oats group = 80  100g-oats group = 79 | Weight; TC; HbA1c | Unclear | Y | N |  |
| Lindstrom 2003 | Finland | 12, 24, 36, 48, 60, 72, 86, 96, 108, 120 | Impaired glucose tolerance (IGT); People at high risk for type 2 diabetes | Control = 257 Intervention = 265 | Weight; TC/HDL ratio; TC; HDL; SBP; HbA1c; FBG; Incidence DM; Incidence CVD morbidity; One timepoint only: Plasma insulin | High | Y | N | Information available from previous reviews. |
| Liss 2016 | USA | 6, 12 | Adults with type 2 diabetes and a BMI ≥ 24 kg/m2 | Standard care arm = 167 Standard care plus group-based lifestyle intervention = 164 | Weight; TC; SBP; HbA1c | Low | Y | Y | Data provided. |
| Little 2016 | UK | 6, 12 | General population | Control, Nurse follow-up = 279 Web-based support with minimal support (Remote) = 270 Web-based + nurse support (face to face) = 269 | Weight; TC; HDL; SBP; HbA1c; FG; QoL | Unclear | N | N/A | Information available from previous reviews. |
| Ma 2015 | USA | 6, 12 | Obese adults with uncontrolled asthma | Control, Enhanced usual care = 165 Diet and counselling = 165 | Weight | Low | N | N/A | Information available from previous reviews. |
| Manzoni 2016 | Italy | 1.4, 12, | Obese patients admitted to the obesity unit of the Istituto Auxologico Italiano, Verbania, Italy for the treatment of obesity and related comorbidities | Control, Standard behavioral inpatient program = 52  Cognitive–behavioral therapy = 54  CBT + Virtual reality = 57 | Weight | High | N | N/A | Information available from previous reviews. |
| Marniemi 1990 | Finland | 2.5, 6, 12 | General obese and overweight population | Control group = 42  Lactovegetarian weight reduction group = 31  Mixed diet weight reduction = 37 | Weight | Unclear | N | N/A |  |
| Martin 2008 | USA | 6, 9, 12, 18 | African American women | Control, Standard Care = 69 Tailored physician/lifestyle counselling = 68 | Weight | High | N | N/A | Information available from previous reviews. |
| Mefferd 2007 |  | 4, 12 | Adult breast cancer survivors with a BMI ≥ 25.0 kg/m^2^ | Control = 29 Intervention = 56 | Weight; TC | Unclear | Y | Y | Data provided; Information available from previous reviews. |
| Melchart 2017 | Germany | 3, 6, 9, 12 | Adults aged 18–67 years who are moderately overweight | Control group = 57  Intervention group = 109 | Weight; TC/HDL ratio; SBP: FG | Low | Y | Y | Data provided. |
| Melin 2003 | Sweden | 3, 6, 12, 24 | Obesity with complication diagnoses (i.e. diabetes type 2, hypertension, dyslipoproteinemia, polycystic ovary disease and apnoea disorder). | Control, less intensively treated = 21 Intensively treated = 22 | Weight; SBP; FG; Plasma insulin | Unclear | N | N/A | Information available from previous reviews. |
| Menard 2005 | Canada | 6, 12, 18 | Patients with type 2 diabetes | Control - usual care = 36 Intervention - intensive multitherapy = 36 | Weight; TC/HDL ratio; SBP; HbA1c; QoL | Unclear | Y | N | Information available from previous reviews. |
| Mensinger 2016 | USA | 6, 24 | General population | Control, Weight Neutral Program = 40 Weight Loss Program = 40 | Weight; TC/HDL ratio; TC; HDL; SBP; FG; QoL | High | N | N/A | Information available from previous reviews. |
| Messier 2013 | USA | 1, 2, 3, 4, 5, 6, 7, 8, 9, 10, 11, 12, 13, 14, 15, 16, 17, 18 | Ambulatory, community-dwelling persons age 55 years or older with mild or moderate knee osteoarthritis | Exercise only = 150  Diet-induced weight loss only = 152  Diet-induced weight loss plus exercise = 152 | Weight; QoL | Unclear | Y | Y | Data provided. |
| Miller 2002 | USA | 2, 12 | NS | Control Group (Monitoring) = 23 Lifestyle Intervention = 22 | Weight; SBP | Low | Y | N | Information available from previous reviews. |
| Mitsui 2008 | Japan | 3, 12 | 50-69-year-old adults | Control = 22  Intervention = 24 | Weight; TC; HDL; SBP; FG | Unclear | Y | N |  |
| Morgan 2010 | Australia | 3, 6, 12 | Males 18-60 years of age who are overweight or obese. | Control (Information and self-help) = 31 SHED-IT (Internet) group = 34 | Weight; SBP | Low | N | N/A | Information available from previous reviews. |
| Muggia 2014 | Italy | 6, 12 | Overweight and obese patients | Standard care group = 83 Brief CBT group = 80 | Weight; TC; HDL; SBP; HOMA-IR; FG; Plasma insulin | High | N | N/A |  |
| Munsch 2003 | Switzerland | 4, 16 | Adults with obesity | GP control = 17 Clinic BASEL = 52 GP BASEL = 53 | Weight | High | N | N/A | Information available from previous reviews. |
| Nakata 2014 | Japan | 3, 6, 18, 30 | Japanese adults | Control (N/A) = 63 Education-only = 62 Group-based support = 63 | Weight; HDL; SBP; FG | Unclear | Y | Y | Data provided. |
| Nanchahal 2012 | UK | 6, 12 | Adults with BMI ≥ 25 kg/m2 | Usual care control = 190 CAMWEL Intervention = 191 | Weight; SBP; QoL | Unclear | Y | Y | Data provided; Information available from previous reviews. |
| Ng 2015 | UK | 4, 12 | Chinese patients with moderate to severe obstructive sleep apnoea (OSA) diagnosed on portable home sleep monitoring. | Control group = 43 Lifestyle modification program = 61 | Weight; TC; FG; QoL | Unclear | Y | Y | Data provided. |
| Nicklas 2004 | USA | 6, 18 | Community-dwelling sedentary adults 60 years of age or above with symptomatic knee osteoarthritis. | Healthy lifestyle control = 78  Exercise only = 80 Diet only = 82  Diet plus exercise = 76 | Weight | Unclear | N | N/A |  |
| Nilsen 2011 | Norway | 6, 12, 18 | Individuals at high risk for type 2 Diabetes | Control, Individual Physician Group = 104  Individual Plus Interdisciplinary Group = 109 | Weight; TC; HDL; SBP; HbA1c; FG | Low | Y | Y | Data provided; Information available from previous reviews. |
| Nordby 2012 | Denmark | 3, 9, 15 | Younger (age: 20–40 years), sedentary, and only moderately overweight (BMI: 25–30 kg/m^2^) men. | Control = 15  Training and increased diet (N/A) = 13 Training = 17 Energy-reduced diet = 15 | Weight; TC; SBP; HbA1c | High | Y | Y | Data provided. |
| Nurkkala 2015 | Finland | 0, 9, 24, 36 | General population between 18-65 years | Control = 30 Intervention group = 90 | Weight | Unclear | N | N/A |  |
| Oldroyd 2006 | UK | 6, 12, 24 | Men and women of European origin | Control group = 39 Intervention group = 39 | Weight; TC; FG | Unclear | N | N/A | Information available from previous reviews. |
| Pan 1997 | China | 24, 48, 72, 96, 120, 144, 168, 192, 216, 240  252, 264, 276, 288, 360 | Chinese participants with impaired glucose tolerance | Control = 138 Intervention group (Exercise: n=155; Diet: n = 148; Diet plus exercise: n = 135) = 438 | Weight; TC; SBP; FG; Incidence CV morbidity; Incidence CV mortality; Incidence T2DM; One timepoint only: HbA1c | High | Y | N |  |
| Parikh 2010 | USA | 3, 6, 12 | Adults with BMI ≥ 25 kg/m^2^ and prediabetes | Control = 49 Intervention = 50 | Weight; SBP; HbA1c | Unclear | Y | N |  |
| Patel 2016 | USA | 6, 12 | Adults 18 to 70 years | Control group = 50 Standard premium discount = 51  Immediate premium discount = 50 Daily lottery incentive = 50 | Weight | Unclear | N | N/A |  |
| Pekkarinen 2015 | Finland | 4, 16, 30 | Obese refereed patients at an outpatient obesity clinic, Peijas Hospital, Helsinki University Central Hospital | Control, Follow up without intervention = 99 One-year maintenance program = 100 | Weight; QoL | Low | N | N/A | Information available from previous reviews. |
| Perri 1984 | USA | 3, 6.5, 9.5, 15.5 | General population | Non-behavioural therapy = 15 Non-behavior therapy plus post-treatment contact = 16  Behavior therapy = 21 Behavior therapy plus relapse prevention training = 15 Behavior therapy plus post-treatment contact = 15  Behavior therapy plus relapse prevention training plus post-treatment contact = 17 | Weight | Unclear | N | N/A |  |
| Perri 2001 | USA | 5, 11, 17 | General population. | Control, Standard Behavioural Therapy (BT) = NS   BT + Relapse prevention training = NS  BT + problem-solving therapy = NS | Weight | Unclear | N | N/A | Information available from previous reviews. |
| Perri 2014 | USA | 6, 24 | General population | Control, Education group = 169  Low dose, (low intensity lifestyle counselling) = 148  Moderate dose, (Moderate intensity lifestyle counselling) = 134 High dose, (High intensity lifestyle counselling) = 161 | Weight | Unclear | N | N/A | Information available from previous reviews. |
| Pettman 2009 | Australia | 4, 12 | Adults with metabolic syndrome | Control = 50 Intervention B - Passive follow-up = 54  Intervention A - Active follow-up = 49 | Weight; TC; SBP; HOMA-IR; One timepoint only: FG | Low | Y | Y | Data provided. |
| Poelman 2015 | Netherlands | 3, 6, 12 | General population | Control Condition = 139 Intervention condition = 139 | Weight | High | Y | Y | Data provided. |
| Promrat 2010 | USA | 3, 6, 9, 12 | Adults who were overweight or obese and diagnosed with Nonalcoholic steatohepatitis | Control = 10 Lifestyle Intervention = 21 | Weight; TC; HbA1c; HOMA-IR | Low | Y | Y | Data provided. |
| Provencher 2009 | Canada | 4, 10, 16 | Premenopausal women | Control group = 48 Social support = 48  Health-At-Every-Size = 48 | Weight; TC; One timepoint only: HDL; | Unclear | Y | Y | Data provided. |
| Rejeski 2011 | USA | 6, 18 | Patients with CVD or cardiometabolic dysfunction | Successful aging control arm, = 93 Physical activity = 97  Weight loss and physical activity = 98 | Weight | Unclear | N | N/A | Information available from previous reviews. |
| Ridgeway 1999 | USA | 6, 12 | Patients with Type 2 Diabetes | Control = 28  Intervention Group = 28 | Weight; TC; HbA1c; FBG | Unclear | N | N/A |  |
| Rolls 2005 | USA | 0.92, 1.8, 2.8, 3.7, 4.6, 5.5, 6.4, 7.4, 8.3, 9.2, 10.1, 11, 12 | Overweight and obese women and men | Comparison-control = 50 Two snacks = 50  One soup = 50  Two soups = 50 | Weight; TC; SBP | Unclear | Y | Y | Data provided. |
| Rosas 2015 | California | 6, 12, 24 | Participants are obese Spanish-speaking adults with at least one cardiovascular risk factor recruited from a community health center in a low-income neighborhood of San Mateo County, California. | Usual care = 41  Case-management intervention = 84 Case-management + Community health worker intervention = 82 | Weight; TC; HDL; SBP; HbA1c; FG | Unclear | N | N/A |  |
| Ross 2012 | Canada | 6, 12, 18, 24 | General population | Control condition = 241 Behavioral intervention group = 249 | Weight; TC; HDL; SBP; FG | Unclear | N | N/A | Information available from previous reviews. |
| Samaras 1997 | Australia | 6, 12 | Mature-aged people, performing less than 1 hour of exercise per week | Control =13  Intervention = 13 | Weight; TC; HDL; HbA1c; FG; Plasma insulin | Unclear | N | N/A | Information available from previous reviews. |
| Santanasto 2011 | USA | 6, 12 | Community dwelling older men and woman age 60 and over, who were overweight to moderately obese and living a sedentary lifestyle (formal exercise less than 3x/week for a total of less than 90 min/week). | Physical Activity plus Successful Ageing = 15 Physical Activity plus Weight Loss = 21 | Weight | Unclear | N | N/A |  |
| Sattin 2016 | USA | 3, 12 | Obese and overweight, and/or prediabetic (FPG of 100 mg/dl to 125 mg/dl). | Health Education intervention = 287  Fit body and soul intervention = 317 | Weight; FBG | Unclear | N | N/A |  |
| Schubel 2016 | Germany | 3, 5.5, 11.5 | Adults between 35-65 years, non-smokers and who are overweight or obese. | Control group = 52  Continuous Calorie Restriction = 49  Intermittent Calorie Restriction = 49 | Weight; TC; HbA1c; HOMA-IR | Low | Y | Y | Data provided. |
| Seligman 2011 | Brazil | 3, 12 | General population (patients with metabolic syndrome - no diabetics, more than half of the participants were hypertensive) | Standard-of-care strategy = 25 Healthy diet and step counter = 25 Healthy diet and fitness = 26 | Weight; TC; HDL; SBP; HOMA-IR | Low | Y | Y | Data provided; Information available from previous reviews. |
| Snel 2012 | The Netherlands | 4, 22 | Adults with insulin-dependent Type 2 diabetes mellitus and obesity | VLCD only = 14 VLCD + exercise = 13 | Weight; HbA1c; QoL | Unclear | N | N/A |  |
| Solbrig 2019 | UK | 6, 12 | General overweight and obese adult population | Motivational interviewing = 58  Functional imagery training = 63 | Weight; QoL | Unclear | N | N/A |  |
| Somers 2012 | USA | 6, 12, 18 | Patients with knee pain and osteoarthritis | Standard Care = 51 Lifestyle behavioral weight management intervention only = 59 Lifestyle behavioral weight management intervention + Pain Coping Skills Training = 62  Pain Coping Skills Training only (N/A) = 60 | Weight | Low | Y | Y | Data provided. |
| Spring 2017 | USA | 3, 6, 12 | General adult population with obesity | Control self-guided program = 32 Standard weight loss program = 32 Technology-supported = 32 | Weight | High | N | N/A |  |
| Stahre 2005 | Sweden | 2.3, 6, 12, 18 | General female population | Control = 43 Cognitive treatment = 62 | Weight | Unclear | N | N/A | Information available from previous reviews. |
| Stahre 2007 | Sweden | 2.3, 8.3, 14.3, 20.3 | Employed women who were childcare providers | Control Group (weight-reducing program) = 27 Cognitive treatment group = 27 | Weight | High | N | N/A |  |
| Stenius-Aarniala 2000 | Finland | 3.2, 6, 12 | People with asthma | Control = 19 Treatment with VLCD = 19 | Weight; QoL | Unclear | Y | N | Information available from previous reviews. |
| Stevens 1993 | USA | 3, 6, 12, 18, 276 | Men and women aged 30 to 54 years with high-normal diastolic blood pressure from 80 through 89 mm hg. | Control = 256 Intervention = 308 | Weight; SBP | Unclear | Y | Y | Data provided. |
| Stevens 2001 | USA | 6, 12, 18, 24, 30, 36 | Adults 30 to 54 years of age who had nonmedicated diastolic blood pressure of 83 to 89 mm Hg and systolic blood pressure less than 140 mm Hg and were 110% to 165% of their ideal body weight at baseline. | Control = 596 Intervention = 595 Sodium only intervention (N/A) = 594 Combined intervention (N/A) = 597 | Weight; SBP | Unclear | Y | Y | Data provided. Information available from previous reviews. |
| Strobl 2013 | Germany | 6, 12 | General population | Control, Usual care = 239 Telephone aftercare =228 | Weight | Low | N | N/A | Information available from previous reviews. |
| Tapsell 2017 | Australia | 3, 12 | Adult residents, 25-54 years, BMI 25-40kg/m2 | Usual care (Control) = 126  Intervention Group = 125 Intervention plus food supplement group (N/A) = 126 | Weight; TC/HDL ratio; TC; SBP; HbA1c; QoL; Remission HTN | High | N | N/A |  |
| TarragaMarcos 2017 | Spain | 0.5, 1, 3, 6, 12 | Adult general obese/overweight population. | G3 = 55 G2 = 61 G1 = 60 | Weight; TC; HDL | Unclear | Y | N |  |
| Teeriniemi 2018 | Finland | 12, 24 | Residents aged 20–60 years living in the city of Oulu who were overweight or obese. | Control = 89  SHG Counselling = 87 CBT Counselling = 85 Control plus HBCSS = 91 SHG Counselling plus HBCSS = 92 CBT Counselling plus HBCSS = 88 | Weight; HDL; SBP; FG | Unclear | Y |  |  |
| ter Bogt 2009 | Netherlands | 12, 36 | Patients 40–70 years of age with BMI: 25 to 40 and either hypertension or dyslipidemia or both. | GP usual care = 232 Lifestyle counselling from NP = 225 | Weight; TC; HDL; SBP; FG | Unclear | N | N/A |  |
| The Look AHEAD Research Group 2010 | USA | 12, 24, 36, 48, 60, 72, 84, 96, 108, 115, 120 | Adults with Type 2 Diabetes Mellitus | Diabetes support and education = 2575 Intensive lifestyle intervention = 2570 | Weight; TC; HDL; SBP: QoL; Incidence CV morbidity; Incidence CV mortality; Incidence T2DM; Remission T2DM | High | N | N/A | Information available from previous reviews. |
| Trepanowski 2017 | USA | 1, 2, 3, 4, 5, 6, 7, 8, 9, 10, 11, 12 | General population with a BMI ≥ 25. | No-intervention control group = 31  Daily calorie restriction group = 35  Alternate-day fasting group = 34 | Weight; HOMA-IR | High | Y | Y | Data provided. |
| Tsai 2010 | USA | 3, 6, 12 | General population | Control = 26 Brief counselling = 24 | Weight; TC; HDL; SBP; FG | Unclear | N | N/A | Information available from previous reviews. |
| Tuomilehto 2009 | Finland | 3, 12, 24, 60 | Patients with mild obstructive sleep apnoea | Control = 41 Intervention = 40 | Weight; TC; HDL; SBP; FBG; Plasma insulin; QoL | Low | N | N/A | Information available from previous reviews. |
| van de Glind 2017 | England, The Netherland, Norway, Portugal | 3, 12 | Males | Comparison group = 553 EuroFIT group = 560 | Weight; TC; SBP; HbA1c; QoL; Final timepoint only: Incidence CV morbidity | High | Y | Y | Information/data received. |
| vanWier 2011 | Netherlands | 6, 24 | General population | Control – Brochure = 460 Internet Group = 464  Phone Group = 462 | Weight; TC; SBP | Low | N | N/A |  |
| Vissers 2010 | Belgium | 3, 6, 12 | General overweight or obesity patients. | Control = 21  Diet only group (Diet) = 20  Diet + fitness training group (Fitness) = 20 Diet + WBV group (Vibration) = 18 | Weight; HDL; SBP; FBG; Final follow-up only: Incidence T2DM | Unclear | N | N/A | Information available from previous reviews. |
| von Gruenigen 2012 | USA | 3, 6, 12 | Women who are overweight with histologically confirmed Stage I or II endometrial cancer | Control = 34 Intervention = 41 | Weight | Unclear | N | N/A | Information available from previous reviews. |
| vonGruenigen 2008 | USA | 3, 6, 12 | Women with endometrial cancer | Control, Usual care = 22 Lifestyle intervention = 23 | Weight; QoL | Unclear | N | N/A |  |
| West 2007 | USA | 6, 12, 18 | Women with type 2 diabetes treated by oral diabetes medications but not insulin | Attention control = 108 Motivational interviewing = 109 | Weight; HbA1c | Unclear | N | N/A |  |
| West 2011 | USA | 4, 12 | Older-adult participants | Control = 112 Lifestyle Intervention = 116 | Weight | Unclear | Y | Y | Data provided. |
| Whelton 1998 | USA | 6, 9, 12, 15, 18, 21, 24, 27, 30 | Adults 60-80 years, with systolic blood pressure lower than 145 mm Hg and diastolic blood pressure lower than 85 mm Hg while receiving treatment with a single antihypertensive medication. | Non-weight loss (Usual lifestyle, control group plus sodium reduction) = NS Weight loss (Weight loss alone plus weight loss and sodium reduction combined intervention) = NS | Weight | High | N | N/A | Information available from previous reviews. |
| Wilson 2016 | USA | 3, 6, 12 | General population | Control - Self Study Group = 242  Phone Fuel Your Life = 182  Group Fuel Your Life = 236 | Weight | Unclear | Y | N |  |
| Wilson 2016b | USA | 6, 12 | General population | Control = 457 FUEL Your Life peer health coaches + nurse education = 459 | Weight | High | Y | Y | Data provided. |
| Wing 1998 | USA | 6, 12, 24 | Overweight participants who had one or two parents with diabetes | Control = 40 Diet = 37 Exercise = 37 Diet plus exercise = 40 | Weight; TC/HDL ratio; TC; HDL; SBP; HbA1c; FG; At final follow-up only: Incidence T2DM | Unclear | N | N/A | Information available from previous reviews. |
| Yannakoulia 2008 | Greece | 2, 12 | Type 2 diabetes mellitus patients | Usual care group = 15 Intensive care group = 15 | Weight; HbA1c | High | Y | Y | Data provided; Information available from previous reviews. |
| Yardley 2014 | UK | 6, 12 | GP patients | Usual care = 43 Web-based only = 45  Basic nurse support = 44 Regular nurse support = 47 | Weight | High | N | N/A | Information available from previous reviews. |
| Yates 2009 | UK | 3, 6, 12, 24 | Patients with impaired glucose tolerance | Control group = 34 PREPARE with pedometer = 33  PREPARE group = 31 | Weight; TC; HDL; SBP; FG; At final follow-up only: Incidence T2DM | Low | N | N/A | Information available from previous reviews. |
| Yates 2018 | USA | 4, 12 | Obese, postmenopausal women with prediabetes and normal endometrial biopsy; Participants were recruited from the community, Harris Health System, and employees at MD Anderson Cancer Center | Placebo + no lifestyle = 8 Metformin + no lifestyle (N/A) = 7 Placebo + lifestyle = 7 Metformin + lifestyle (N/A) = 7 | Weight | High | Y | N | Information available from previous reviews. |
| Yeh 2016 | USA | 6, 12 | Chinese immigrants with prediabetes living in New York City | Control group = 30 Intervention group = 30 | Weight; TC; SBP; HbA1c | Unclear | Y | N | Information available from previous reviews. |
| Yin 2018 | China | 6, 12 | Women with pre-diabetes | Comparison-Control Group = 75 Intervention Group = 109 | Weight; HbA1c; FG | Unclear | N | N/A |  |
| Zhang 2016 | China | 6, 12, 24 | Patients with Nonalcoholic Fatty Liver Disease | Control = 74 Moderate exercise = 73 Vigorous-moderate exercise = 73 | Weight; TC; HDL; SBP; FG | Low | N | N/A | Information available from previous reviews. |
| **BED:** Binge Eating Disorder; **BMI:** Body Mass Index (kg/m^2^); **CBT:** Cognitive Behaviour Therapy; **CV:** Cardiovascular; **DPP:** Diabetes Prevention Program; **FG:** Fasting glucose (including fasting plasma glucose and other glucose measures)**; HbA1c:**  Haemoglobin A1C**; HDL:** High-density lipoprotein cholesterol; **N:** No; **HOMA-IR:** Homeostatic Model Assessment of Insulin Resistance**; HTN:** Hypertension; **MI:** Motivational interviewing; **MR:** Meal replacement; **N/A:** Not applicable; **NS:** Not specified; **QoL:** Quality of Life; **SBP:** Systolic Blood Pressure; **T2DM:** Type 2 Diabetes Mellitus; **TC:** Total cholesterol; **TC/HDL**: Total cholesterol/High-density lipoprotein ratio; **VLCD:** Very low calorie diet; **Y:** Yes.  *Not all outcomes measures collected at all follow-up time points; Outcome measures collected at baseline only not listed.  ^#^ Additional information or data obtained from study authors. | | | | | | | | | |

### Table 6. Baseline demographics

| **Study ID** | **Groups:** | **Randomised** | **Number of participants reported at baseline** | **Gender**  **(%F)** | **Age** | | **BMI** | | **Comorbidities at baseline (%) *** | | |
| --- | --- | --- | --- | --- | --- | --- | --- | --- | --- | --- | --- |
|  |  |  |  |  | ***Mean*** | ***SD*** | ***Mean*** | ***SD*** | ***CV morbidity*** | ***Type II DM*** | ***Hypertension*** |
| Abed 2013 | Control | 75 | 75 | 33 | 60.3 | 10.3 | 33.8 | 4.1 | 100 | 28 | 87 |
|  | Weight Management | 75 | 75 | 32 | 59.8 | 9.5 | 32.8 | 3.5 | 100 | 24 | 83 |
| Ackermann 2011 | Standard advice alone (controls) | 46 | 46 | 61 | 60.1 | 10.5 | 30.8 | 5.1 | NR | NR | NR |
|  | YMCA DPP intervention | 46 | 46 | 50 | 56.5 | 9.7 | 32.0 | 4.8 | NR | NR | NR |
| Agras 1990 | Computer alone | 30 | 30 | 100 | 45.2 | 12.4 | 29.7 | 4.3 | NR | NR | NR |
|  | Computer + group support | 30 | 30 |  |  |  |  |  | NR | NR | NR |
|  | Behaviour therapy | 30 | 30 |  |  |  |  |  | NR | NR | NR |
| Ahern 2017 | Brief intervention | 211 | 211 | 68 | 51.9 | 14.1 | 34.4 | 4.6 | NR | 13.5 | 49.8 |
|  | 12-week behavioural weight-loss programme | 530 | 528 | 68 | 53.6 | 13.3 | 34.7 | 5.4 |  |  |  |
|  | 52-week behavioural weight-loss programme | 528 | 528 | 68 | 53.3 | 14.0 | 34.5 | 5.1 |  |  |  |
| Almanza -Aguilera 2018 | Control (general recommendations) | 48 | 27 | 100 | 44.4 | 3.3 | 36.3 | 5.7 | NR | 0 | NR |
|  | Treatment (lifestyle weight loss intervention) | 67 | 30 | 100 | 45.7 | 3.5 | 35.4 | 4.1 | NR | 0 | NR |
| Anderson 2014 | Control (weight loss booklet only) | 166 | 166 | 26 | 63.6 | 6.7 | 30.4 | 3.9 | NR | 14.3 | NR |
|  | Intervention (BeWEL) | 163 | 163 | 26 | 63.5 | 7.0 | 31.0 | 4.5 | NR |  | NR |
| Appel 2011 | Control (Self-directed) | 138 | 138 | 63.8 | 52.9 | 10.1 | 36.8 | 5.1 | NR | 23.8 | 76.8 |
|  | Remote Support Only (N/A) | 139 | N/A | N/A | N/A | N/A | N/A | N/A | N/A | N/A | N/A |
|  | In-Person Support | 138 | 138 | 63.8 | 53.3 | 10.5 | 36.8 | 5.2 | NR | 23.9 | 71.0 |
| Ard 2004 | “advice only” comparison group | 273 | 273 | 63 | 49.5 | 8.8 | 32.9 | 5.6 | NR | NR | 14.0 |
|  | “established” behavioural intervention group | 268 | 268 | 64.9 | 50.2 | 8.6 | 33.0 | 5.5 | NR | NR | 13.7 |
|  | Established + DASH Intervention Group | 269 | 269 | 57.2 | 50.2 | 9.2 | 33.3 | 6.3 | NR | NR | 13.8 |
| Ard 2018 | Exercise Only | 54 | 54 | 68.5 | 69.9 | 4.5 | 33.9 | 0.4 | NR | NR | NR |
|  | Exercise + Diet Quality + Weight Maintenance | 55 | 55 | 60 | 70.5 | 4.8 | 33.8 | 0.4 | NR | NR | NR |
|  | Exercise + Diet Quality + Weight Loss | 55 | 55 | 58.2 | 70.3 | 4.8 | 33.3 | 0.4 | NR | NR | NR |
| Ash 2006 | Control Group - Booklet only | 63 | 54 | 77.8 | 47 | 14 | 35.8 | 6.2 | NR | NR | NR |
|  | Individualised Dietetic Treatment | 66 | 65 | 75.4 | 48 | 13 | 34.2 | 5.9 | NR | NR | NR |
|  | Fat Booters Incorporated | 62 | 57 | 66.7 | 49 | 13 | 33.7 | 4.6 | NR | NR | NR |
| Aveyard 2016 | Advice only | 942 | 942 | 57 | 56.2 | 15.6 | 35.1 | 5.1 | NR | NR | NR |
|  | Advice plus weight loss programme | 940 | 940 | 57.3 | 55.8 | 16.5 | 34.8 | 4.6 | NR | NR | NR |
| Azar 2013 | Control, Usual care | NR | 81 | 45.7 | 52.5 | 10.6 | 32.4 | 6.3 | NR | NR | NR |
|  | Self-directed | NR | 81 | 45.7 | 51.8 | 9.9 | 31.7 | 4.7 | NR | NR | NR |
|  | Coach-led | NR | 79 | 48.1 | 54.6 | 11.0 | 31.8 | 5.1 | NR | NR | NR |
| Bacon 2002 | Health at Every Size - control | NR | 29 | 100 | 39.3 | 4.5 | 35.9 | 4.1 | NR | NR | NR |
|  | Diet Group - intervention | NR | 23 |  |  |  | 36.6 | 4.1 | NR | NR | NR |
| Barnes 2017 | Treatment as usual (N/A) | 30 | 30 | N/A | N/A | N/A | N/A | N/A | N/A | N/A | N/A |
|  | Nutrition - ATTENTION CONTROL | 29 | 29 | 69 | 48.9 | 11.6 | 35.1 | 7.5 | NR | NR | NR |
|  | Motivational interviewing | 30 | 30 | 80 | 47.1 | 10.0 | 34.7 | 7.1 | NR | NR | NR |
| Bartels 2015 | Control, Fitness club membership | 106 | 106 | 55 | 43.5 | 11.6 | 37.5 | 8.8 | NR | NR | NR |
|  | IN SHAPE | 104 | 104 | 47 | 44.3 | 10.9 | 36.2 | 7.5 | NR | NR | NR |
| Beavers 2017 | Weight loss | 82 | 82 | 72 | 66.3 | 4.5 | 34.7 | 4.0 | 23.2 | 15.9 | 69.5 |
|  | Weight loss + Aerobic training | 86 | 86 | 72.1 | 67.5 | 5.1 | 33.9 | 3.5 | 27.9 | 22.1 | 77.9 |
|  | Weight loss + Resistance training | 81 | 81 | 69.1 | 66.9 | 4.4 | 34.8 | 3.6 | 27.2 | 19.8 | 74.1 |
| Bennett 2012 | Control, Usual care | 185 | 185 | 65.9 | 54.7 | 11.0 | 36.99 | 5.2 | NR | NR | NR |
|  | Be Fit, Be Well | 180 | 180 | 71.1 | 54.6 | 10.8 | 37.03 | 5.0 | NR | NR | NR |
| Bennett 2013 | Control, usual care | 97 | 94 | 100 | 35.2 | 5.5 | 30.2 | 2.4 | NR | 5.3 | 36.2 |
|  | Weight gain prevention intervention | 97 | 91 | 100 | 35.6 | 5.5 | 30.1 | 2.7 | NR | 5.8 | 36.3 |
| Berry 2014 | Control | 162 | 162 | 92.6 | 36.8 | 8.1 | 39.1 | 8.3 | NR | NR | NR |
|  | Family based. Nutrition, exercise and coping skills intervention | 184 | 184 | 92.9 | 36.9 | 8.1 | 36.4 | 8.3 | NR | NR | NR |
| Bertram 1990 | Control - diet only | 15 | 8 | 100 | 37.4 | 1.6^b^ | 34.3 | 1.3^b^ | NR | NR | NR |
|  | Diet plus lectures | 15 | 15 | 100 | 38.4 | 1.8^b^ | 34.8 | 1.3^b^ | NR | NR | NR |
|  | Diet plus exercise | 15 | 13 | 100 | 37.2 | 1.8^b^ | 34.6 | 1.6^b^ | NR | NR | NR |
| Bertz 2012 | Control | 17 | 17 | 100 | 32.2 | 4.6 | 30.2 | 3.4 | NR | NR | NR |
|  | Diet Only | 17 | 17 | 100 | 33.7 | 4.2 | 33.7 | 2.6 | NR | NR | NR |
|  | Exercise only | 18 | 18 | 100 | 33.2 | 3.7 | 33.2 | 3.1 | NR | NR | NR |
|  | Intervention | 16 | 16 | 100 | 33.9 | 4.5 | 33.9 | 2.2 | NR | NR | NR |
| Bo 2007 | Control standard care | 188 | 166 | 57.8 | 55.7 | 5.6 | 29.8 | 4.6 | NR | NR | 36.1 |
|  | Intervention lifestyle by trained professional | 187 | 169 | 58.6 | 55.7 | 5.7 | 29.7 | 4.1 | NR | NR | 36.1 |
| Brown 2014 | Control | 66 | 66 | 70 | 44.9 | 10.1 |  | 3.4 | NR | NR | NR |
|  | RENEW | 70 | 70 | 64 | 44.4 | 11.7 |  | 2.6 | NR | NR | NR |
| Burke 2005 | Control usual care | 118 | 118 | 57 | 55.3 | 7.5 | 29.7 | 2.5 | NR | NR | 100 |
|  | Low sodium + fish diet | 123 | 123 | 54.5 | 57.1 | 7.2 | 30.4 | 2.9 | NR | NR | 100 |
| Burke 2015 | Standard behavioural weight loss treatment | 72 | 72 | 83.1 | 53 | 9.6 | 33.2 | 4.11 | NR | NR | NR |
|  | Self-efficacy enhancement plus standard behavioural weight loss treatment | 58 | 58 |  |  |  |  |  |  |  |  |
| Cheyette 2007 | Control | 20 | 20 | 40 | 58 | 10.7 | 31.7 | 5.4 | NR | 100 | NR |
|  | Weight No More intervention group | 29 | 29 | 51.7 | 56.7 | 9.7 | 34.1 | 4.7 | NR | 100 | NR |
| Christensen 2012 | Reference group | 44 | 44 | 100 | 46 | 8.6 | 30.4 | 4.9 | NR | NR | NR |
|  | Intervention group | 54 | 54 | 100 | 45.7 | 6.36 | 30.5 | 5.4 | NR | NR | NR |
| Cleo 2018 | Wait list control (N/A) | 25 | N/A | N/A | N/A | N/A | N/A | N/A | N/A | N/A | N/A |
|  | TTT Top Ten Tips habit formation | 25 | 25 | 80 | 48.2 | 11.3 | 34.6 | 5.2 | NR | NR | NR |
|  | DSD Do Something Different online software | 25 | 25 | 76 | 51.3 | 10.0 | 35.2 | 7.4 | NR | NR | NR |
| Conroy 2015 | Self-guided | 50 | 49 | 100 | 54 | 5.6 | 33.4 | 5.4 | NR | 23.5 | 56.1 |
|  | Interventionist led | 49 | 49 | 100 | 53.8 | 5.3 | 36.1 | 5.4 | NR |  |  |
| Cooper 2010 | Guided Self-Help Control | 51 | 51 | 100 | 41.86 | 8.67 | 35.41 | 2.71 | NR | 0 | NR |
|  | Behaviour Therapy | 50 | 50 | 100 | 41.38 | 9.90 | 34.79 | 3.06 | NR | 0 | NR |
|  | Cognitive Behaviour Therapy | 49 | 49 | 100 | 41.2 | 8.77 | 33.85 | 2.71 | NR | 0 | NR |
| Cousins 1992 | Control | 56 | 27 | 100 | 33.8 | 7.0 | 31.6 | 4.9 | NR | NR | NR |
|  | Individual | 56 | 32 | 100 | 33.6 | 6.4 | 31.7 | 5.0 | NR | NR | NR |
|  | Family | 56 | 27 | 100 | 32.8 | 6.1 | 30.3 | 4.5 | NR | NR | NR |
| Craighead 1989 | Control, minimal contact | 20 | 11 | NR | NR | NR | NR | NR | NR | NR | NR |
|  | Contracted Exercise | 20 | 14 | NR | NR | NR | NR | NR | NR | NR | NR |
|  | Supervised Exercise | 22 | 17 | NR | NR | NR | NR | NR | NR | NR | NR |
| Dale 2009 | Control | 23 | 23 | 74 | 45 | 30, 68^c^ | 36.5 | 4.3 | NR | NR | NR |
|  | Modest | 31 | 31 | 61 | 48 | 32, 62^c^ | 33.9 | 4.4 | NR | NR | NR |
|  | Intensive intervention | 25 | 25 | 68 | 46 | 31, 68^c^ | 32.5 | 5.2 | NR | NR | NR |
| Dalziel 2006 | Control | 303 | 303 | 7.9 | 53.5 | 10 | 25.8 | 3.4 | 100 | NR | NR |
|  | Experimental | 302 | 302 | 10.6 | 53.5 | 10 | 25.8 | 3.4 | 100 | NR | NR |
| Damschroder 2014 | Control, MOVE - usual care | 159 | 159 | 12.6 | 54.6 | 10.5 | 36.8 | 6.4 | NR | 37.7 | 65.4 |
|  | ASPIRE group, individual telephone counselling | 162 | 162 | 16 | 55.4 | 10.0 | 36.3 | 6.2 | NR | 32.7 | 67.9 |
|  | ASPIRE group, group counselling | 160 | 160 | 16.2 | 54.9 | 9.5 | 36.2 | 6.1 | NR | 40.0 | 65.6 |
| Daumit 2013 | Control, Usual care | 147 | 147 | 49 | 44.1 | 11.0 | 36.5 | 7.3 | NR | NR | NR |
|  | ACHIEVE | 144 | 144 | 51.4 | 46.6 | 11.5 | 36 | 7.2 | NR | NR | NR |
| deVos 2016 | Control | 204 | 204 | 100 | 55.7 | 3.2 | 32.5 | 4.5 | NR | NR | 74.5 |
|  | Tailor-made lifestyle intervention | 203 | 203 | 100 | 55.7 | 3.2 | 32.2 | 4.1 | NR | NR | 68.5 |
| Diabetes Prevention Program R G 2009 | Placebo | 1082 | 1082 | 69 | 50.3 | 10.4 | 32.2 | 6.7 | NR | All at high risk of T2DM | 30.0 |
|  | Metformin (N/A) | 1073 | 1073 | N/A | N/A | N/A | N/A | N/A | N/A |  |  |
|  | Lifestyle | 1079 | 1079 | 68 | 50.6 | 11.3 | 33.9 | 6.8 | NR |  |  |
| Djuric 2002 | Control | 13 | 48 | 100 | 51.7 | 8.4 | 34.9 | 1.2^b^ | NR | 6.3 | NR |
|  | Weight Watchers | 11 |  | 100 |  |  | 35 | 1.2^b^ | NR |  | NR |
|  | Individualized group | 13 |  | 100 |  |  | 35.5 | 1.1^b^ | NR |  | NR |
|  | Comprehensive group | 11 |  | 100 |  |  | 36.8 | 1^b^ | NR |  | NR |
| Duncan 2016 | Control | 162 | 159 | 42.8 | 54.8 | 8.48 | 31.8 | 6.91 | NR | NR | NR |
|  | Intervention | 158 | 154 | 45.1 | 53.1 | 9.83 | 33.8 | 7.14 | NR | NR | NR |
| Eakin 2014 | Usual care | 151 | 151 | 43.0 | 58.3 | 9.0 | 33.2 | 6.0 | 74.8 | 100 | NR |
|  | Telephone intervention | 151 | 151 | 44.4 | 57.7 | 8.1 | 33.1 | 6.3 | 84.1 | 100 | NR |
| Eaton 2016 | Control, Standard Intervention | 106 | 106 | 83 | 48.6 | 11.2 | 37.8 | 6.7 | NR | NR | NR |
|  | Enhanced Intervention | 105 | 105 | 75.2 | 48.5 | 11.9 | 37.7 | 6.5 | NR | NR | NR |
| Fernandez-Ruiz 2018 | Control | 37 | 37 | 51.4 | 62.8 | 8.9 | 34.3 | 4.5 | NR | 62.2 | 86.5 |
|  | Intervention (healthy eating, exercise & CBT) | 37 | 37 | 48.6 | 59.4 | 9.1 | 32.4 | 3.8 | NR | 43.2 | 78.4 |
| Foley 2016 | Usual care (Control) | 175 | 175 | 68 | 50.5 | 8.7 | 35.9 | 3.7 | NR | 3.4 | 29.1 |
|  | Weight loss intervention | 176 | 176 | 68 | 50.9 | 9.1 | 35.9 | 4.1 | NR | 3.4 | 29.5 |
| Foster-Schubert 2012 | Control- usual care | 87 | 87 | 100 | 57.4 | 4.4 | 30.7 | 3.9 | NR | NR | NR |
|  | Calorie reduced diet | 118 | 118 | 100 | 58.1 | 5.9 | 31 | 3.9 | NR | NR | NR |
|  | Aerobic exercise (N/A) | 117 | 117 | N/A | N/A | N/A | N/A | N/A | N/A | N/A | N/A |
|  | Intervention - diet and exercise | 117 | 117 | 100 | 58.0 | 4.5 | 31 | 4.3 | NR | NR | NR |
| Fuller 2012 | Western diet group | 35 | 35 | 54.3 | 47.1 | 11.1 | 31.0 | 3.8 | NR | NR | NR |
|  | Korean diet group | 35 | 35 | 71.4 | 43.7 | 11 | 31.2 | 4.0 | NR | NR | NR |
| Goodwin 2014 | Mailed-based intervention | 167 | 167 | 100 | 60.4 | 7.8 | 31.1 | 5.3 | NR | 0.0 | NR |
|  | Individual lifestyle intervention | 171 | 171 | 100 | 61.6 | 6.7 | 31.4 | 5.0 | NR | 0.0 | NR |
| Green 2015 | Usual care | 96 | 96 | 71.9 | 48.3 | 9.7 | 38.2 | 7.3 | NR | 16.7 | 30.2 |
|  | STRIDE | 104 | 104 | 72.1 | 46.2 | 11.4 | 38.3 | 9.1 | NR | 13.5 | 28.8 |
| Grilo 2011 | Cognitive Behavioral Therapy (CBT) | 45 | 45 | 64.4 | 45.2 | 8.5 | 39.3 | 6.1 | NR | NR | NR |
|  | Behavioral weight loss (BWL) | 45 | 45 | 62.2 | 44.6 | 10.5 | 38 | 5.3 | NR | NR | NR |
|  | CBT + BWL (N/A) | 35 | NR |  |  |  |  |  | N/A | N/A | N/A |
| Grilo 2014 | Placebo | 27 | 27 | 66.7 | 43.2 | 12.4 | 39.3 | 5.5 | NR | NR | NR |
|  | Placebo/CBTsh | 25 | 25 | 80 | 45.7 | 12.4 | 36.5 | 5.3 | NR | NR | NR |
|  | Sibutramine (N/A) | 26 | NR | N/A | N/A | N/A | N/A | N/A | N/A | N/A | N/A |
|  | Sibutramine/CBTsh (N/A) | 26 | NR | N/A | N/A | N/A | N/A | N/A | N/A | N/A | N/A |
| Hanson 1976 | No treatment control condition (N/A) | 10 | 66 | 87.9 | 40 | NR | NR | NR | NR | NR | NR |
|  | Attention-placebo control condition | 11 |  |  |  |  |  |  |  |  |  |
|  | Conventional self-management condition | 7 |  |  |  |  |  |  |  |  |  |
|  | Programmed text with low therapist-group contact | 12 |  |  |  |  |  |  |  |  |  |
|  | Programmed text with high therapist-group contact | 13 |  |  |  |  |  |  |  |  |  |
| Hardcastle 2013 | Control | 131 | 131 |  | 50.41 | 10.87 | 33.37 | 4.47 | NR | NR | 18.3 |
|  | MI counselling intervention | 203 | 203 |  | 50.1 | 10.54 | 33.66 | 5.12 | NR | NR | 22.7 |
| Harrigan 2016 | Usual Care Group | 33 | 33 | 100 | 58 | 7.5 | 34 | 7.5 | NR | NR | NR |
|  | Telephone Weight Loss Counseling | 34 | 34 | 100 | 60 | 7.7 | 31.8 | 5.4 | NR | NR | NR |
|  | In-Person Weight Loss Counseling | 33 | 33 | 100 | 58.9 | 7.3 | 33.5 | 6.7 | NR | NR | NR |
| Harris 2017 | Waist Winners Too | 24 | 24 | 58.3 | 43.6 | 14.0 | 41.2 | 8.1 | NR | 12.5 | 45.8 |
|  | TAKE 5 | 26 | 26 | 69.2 | 40.6 | 15.0 | 40.2 | 6.8 | NR | 3.8 | 46.2 |
| Hunt 2014 | Control, Wait-list | 373 | 373 | 0 | 47.2 | 7.89 | 35.1 | 4.8 | NR | NR | NR |
|  | FFIT | 374 | 374 | 0 | 47 | 8.07 | 35.5 | 5.1 | NR | NR | NR |
| Huseinovic 2016 | Control Group | 56 | 56 | 100 | 32.6 | 4.7 | 31^a^ | 26.3–48.7^c^ | NR | NR | NR |
|  | Diet behaviour modification Group | 54 | 54 | 100 | 31.8 | 4.5 |  |  | NR | NR | NR |
| Irwin 2003 | Control Group | 86 | 86 | 100 | 60.6 | 59.1, 62.1^d^ | 30.6 | 29.8, 31.4^d^ | NR | NR | NR |
|  | Exercise group | 87 | 87 |  | 61 | 59.6, 62.5^d^ | 30.5 | 29.6, 31.4^d^ | NR | NR | NR |
| Jackson 1982 | Control | 6 | 6 | 100 | 23.5 | 16-34^c^ | NR | NR | NR | NR | NR |
|  | Treatment | 6 | 6 | 100 | 21.8 | 16-34^c^ | NR | NR | NR | NR | NR |
| Jakicic 2011 | Self Help Group | 89 | 89 | 92 | 44.7 | 7.9 | 27.1 | 1.7 | NR | NR | NR |
|  | Moderate Physical Activity | 82 | 82 | 90 | 43.5 | 8.8 | 27.1 | 1.7 | NR | NR | NR |
|  | High Physical Activity | 98 | 98 | 92 | 45 | 8.4 | 27 | 1.6 | NR | NR | NR |
| Jebb 2011 | Standard care | 395 | 395 | 86 | 48.2 | 12.2 | 31.3 | 2.6 | NR | 6.8 | 25.1 |
|  | Commercial programme | 377 | 377 | 88 | 46.5 | 13.5 | 31.5 | 2.6 | NR | 6.4 | 25.5 |
| Jebb 2017 | Usual care | 140 | 140 | 60.0 | 47.4 | 12.8 | 36.8 | 5.1 | NR | 14.3 | 21.4 |
|  | Low energy total diet replacement programme | 138 | 138 | 60.5 | 48.2 | 11.5 | 37.6 | 5.7 | NR | 15.2 | 23.9 |
| Jeffery 1995 | Control group | 40 | 40 | 50 | 35.7 | NR | 31.1 | NR | NR | NR | NR |
|  | Standard Behavioural Therapy (SBT) | 40 | 40 |  | 37.5 | NR | 30.9 | NR | NR | NR | NR |
|  | SBT + Incentives (I) | 41 | 41 |  | 38.1 | NR | 31.1 | NR | NR | NR | NR |
|  | SBT + Food Provision (FP) | 40 | 40 |  | 38.5 | NR | 30.8 | NR | NR | NR | NR |
|  | SBT + FP + I | 41 | 41 |  | 37.6 | NR | 31.1 | NR | NR | NR | NR |
| Jenkins 2017 | Control | 486 | 486 | 79.4 | 44.9 | 43.8, 46.0 d | 32.5 | 32.0, 33.0 d | 5.8 | NR | 8.6 |
|  | Dietary advice only | 145 | 145 | 75.9 | 46.2 | 44.0, 48.4 d | 31.7 | 30.8, 32.7 d | 7.6 | NR | 9.0 |
|  | Food basket only | 148 | 148 | 72.3 | 44.9 | 43.1, 46.7 d | 32.6 | 31.6, 33.5 d | 7.4 | NR | 10.1 |
|  | Food and advice | 140 | 140 | 76.4 | 42.4 | 40.4, 44.4 d | 32.7 | 31.7, 33.7 d | 5.0 | NR | 5.7 |
| John 2011 | Control | 22 | 22 | 18.2 | NR | NR | 34.7 | 3.2 | 9.1 | 4.4^#^ | 68.2 |
|  | Deposit contracts group | 44 | 44 | 15.9 | NR | NR | 34.6 | 2.4 | 18.2 | 15.9^#^ | 59.1 |
| Jolly 2011 | Minimal intervention comparator | 100 | 100 | 75 | 49.67 | 13.83 | 33.9 | 4.4 | NR | NR | NR |
|  | Choice (N/A) | 100 | N/A | N/A | N/A | N/A | N/A | N/A | N/A | N/A | N/A |
|  | Pharmacy | 70 | 70 | 73 | 48.9 | 15.8 | 33.4 | 3.5 | NR | NR | NR |
|  | General practice | 70 | 70 | 67 | 50.5 | 13.8 | 33.1 | 3.5 | NR | NR | NR |
|  | Weight Watchers | 100 | 100 | 72 | 50.7 | 14.6 | 34.0 | 3.9 | NR | NR | NR |
|  | NHS Size Down | 100 | 100 | 64 | 48.8 | 15.6 | 33.8 | 3.9 | NR | NR | NR |
|  | Rosemary Conley | 100 | 100 | 69 | 49.8 | 49.8 | 33.4 | 3.5 | NR | NR | NR |
|  | Slimming world | 100 | 100 | 65 | 48.8 | 14.9 | 33.8 | 3.8 | NR | NR | NR |
| Jones 1986 | Individual | 21 | 160 | 100 | 50.3 | 13.5 | 35.1 | 9.2 | NR | 0.0 | NR |
|  | Group | 17 |  |  |  |  |  |  | NR |  | NR |
|  | Leaflet Individual | 22 |  |  |  |  |  |  | NR |  | NR |
|  | Leaflet Group | 20 |  |  |  |  |  |  | NR |  | NR |
|  | Diary Individual | 20 |  |  |  |  |  |  | NR |  | NR |
|  | Diary Group | 19 |  |  |  |  |  |  | NR |  | NR |
|  | Leaflet Diary Individual | 21 |  |  |  |  |  |  | NR |  | NR |
|  | Leaflet Diary Group | 20 |  |  |  |  |  |  | NR |  | NR |
| Jones 1999 | Control Group | NS | 51 | 49.0 | 59 | 7 | 34 | 6 | NR | NR | 100 |
|  | Weight Loss Group | NS | 51 | 54.9 | 57 | 6 | 34 | 6 | NR | NR | 100 |
| Katzer 2008 | Mail-delivered 'non-dieting' program (P3) | 101 | 225 | 100 | 46.1 | 8.9 | 35.4 | 5.7 | NR | NR | NR |
|  | Group 'non-dieting' program (P2) | 62 |  |  |  |  |  |  | NR | NR | NR |
|  | Group 'non-dieting' program plus Relaxation (P1) | 62 |  |  |  |  |  |  | NR | NR | NR |
| Kuller 2012 | Control - health education | 255 | 255 | 100 | 57 | NR | 30.9 | 3.8 | NR | NR | NR |
|  | Intervention - lifestyle change | 253 | 253 | 100 | 56 | NR | 30.6 | 3.8 | NR | NR | NR |
| Leahey 2014 | SURI alone | 46 | 46 | 82.6 | 46.5 | 1.7 ^b^ | 35.1 | 1.3 ^b^ | NR | NR | NR |
|  | SURI plus Internet behavioral weight loss program | 90 | 90 | 82.2 | 46.2 | 1.2^b^ | 34.7 | 0.7 ^b^ | NR | NR | NR |
|  | SURI plus Internet behavioral weight loss program plus optional group sessions | 94 | 94 | 86.2 | 47.7 | 1.1 ^b^ | 33.4 | 0.7 ^b^ | NR | NR | NR |
| Ley 2004 | Control diet | 70 | 70 | 20 | 52 | 0.8 ^b^ | 29.1 | 0.6 ^b^ | NR | NR | NR |
|  | Reduced-fat | 66 | 66 | 31.8 | 52.5 | 0.8 ^b^ | 29.3 | 0.6 ^b^ | NR | NR | NR |
| Li 2016 | Usual care group | 60 | 60 | 35 | 59 | 3.9 | 25.2 | 0.9 | NR | 100 | NR |
|  | Diet group | 79 | 79 | 46.8 | 59.7 | 6.5 | 27.2 | 2.8 | NR | 100 | NR |
|  | 50g-oats group | 80 | 80 | 48.8 | 59.7 | 6.1 | 26.9 | 2.7 | NR | 100 | NR |
|  | 100g-oats group | 79 | 79 | 58.2 | 59.4 | 6.8 | 27.4 | 2.4 | NR | 100 | NR |
| Lindstrom 2003 | Control | 257 | 257 | 68.5 | 55 | 7 | 31.4 | 4.5 | NR | NR | 31.1 |
|  | Intervention | 265 | 265 | 65.7 | 55 | 7 | 31.1 | 4.5 | NR | NR | 29.1 |
| Liss 2016 | Standard care arm | 167 | 167 | 48.5 | 56.6 | 12.2 | 34.9 | 7.3 | NR | 100 | 76.6 |
|  | Standard care plus group-based lifestyle intervention | 164 | 164 | 51.8 | 57.1 | 10.6 | 36.2 | 7.8 | NR | 100 | 80.5 |
| Little 2016 | Control, Nurse follow-up | 279 | 279 | 66 | 52.7 | 13.3 | 37.1 | 6.0 | NR | NR | NR |
|  | Web-based support with minimal support (Remote) | 270 | 270 | 60 | 54.7 | 13.0 | 36.3 | 5.7 | NR | NR | NR |
|  | Web-based + nurse support (face to face) | 269 | 269 | 65 | 53.7 | 13.2 | 36.7 | 5.4 | NR | NR | NR |
| Ma 2015 | Control, Enhanced usual care | 165 | 165 | 70.9 | 47.7 | 12.1 | 37.6 | 5.7 | NR | NR | NR |
|  | Diet and counselling | 165 | 165 | 70.3 | 47.5 | 12.6 | 37.4 | 6.0 | NR | NR | NR |
| Manzoni 2016 | Control, Standard behavioral inpatient program | 52 | 158 | 100 | 35.6 | 8.04 | 42.2 | 6.01 | NR | NR | NR |
|  | Cognitive–behavioral therapy | 54 |  |  |  |  |  |  | NR | NR | NR |
|  | CBT + Virtual reality | 57 |  |  |  |  |  |  | NR | NR | NR |
| Marniemi 1990 | Control group | 42 | 42 | 76.2 | 38.0 | NR | 33.6 | NR | NR | NR | NR |
|  | Lactovegetarian weight reduction group | 31 | 31 | 74.2 |  |  | 34.4 | NR | NR | NR | NR |
|  | Mixed diet weight reduction | 37 | 37 | 73.0 |  |  | NR | NR | NR | NR | NR |
| Martin 2008 | Control, Standard Care | 69 | 69 | 100 | 42.6 | 11.4 | 39.8 | 7.8 | NR | NR | NR |
|  | Tailored physician/lifestyle counselling | 68 | 68 | 100 | 40.8 | 12.7 | 38.3 | 7.5 | NR | NR | NR |
| Mefferd 2007 | Control | 29 | 29 | 100 | 56.4 | 7.5 | 31.3 | 4.8 | NR | NR | NR |
|  | Intervention | 56 | 56 | 100 | 55.9 | 8.7 | 31 | 3.7 | NR | NR | NR |
| Melchart 2017 | Control group | 57 | 55 | 72.7 | 52.1 | 10 | 31.5 | 2 | NR | 0.0 | NR |
|  | Intervention group | 109 | 111 | 74.8 | 49.9 | 9.7 | 31.8 | 2 | NR | 0.0 | NR |
| Melin 2003 | Control, less intensively treated | 21 | 21 | 90.7 | 39.4 | 26-57^c^ | 35.2 | 4.6 | NR | 14.3 | NR |
|  | Intensively treated | 22 | 22 |  | 40.7 | 25-60^c^ | 35.6 | 4.5 | NR | NR | NR |
| Menard 2005 | Control - usual care | 36 | 36 | 38.9 | 55.9 | 8.6 | 32.6 | 5.7 | NR | 100 | NR |
|  | Intervention - intensive multitherapy | 36 | 36 | 25 | 53.7 | 7.5 | 32.9 | 5.5 | NR | 100 | NR |
| Mensinger 2016 | Control, Weight Neutral Program | 40 | 40 | 100 | 39.8 | 4.34 | 37.4 | 0.6 | NR | NR | NR |
|  | Weight Loss Program | 40 | 40 | 100 | 39.4 | 3.91 | 38.6 | 0.7 | NR | NR | NR |
| Messier 2013 | Exercise only | 150 | 150 | 72 | 66 | 6 | 33.5 | 3.7 | 8.0 | 12.0^#^ | 59.3 |
|  | Diet-induced weight loss only | 152 | 152 | 71 | 66 | 6 | 33.7 | 3.8 | 12.5 | 11.8^#^ | 61.2 |
|  | Diet-induced weight loss plus exercise | 152 | 152 | 72 | 65 | 6 | 33.6 | 3.7 | 7.2 | 15.1^#^ | 59.9 |
| Miller 2002 | Control Group (Monitoring) | 23 | 23 | 68 | 54 | 8 | 34.2 | 6.2 | NR | NR | 23 |
|  | Lifestyle Intervention | 22 | 22 | 57 | 53 | 11 | 32.8 | 5.4 | NR | NR | 22 |
| Mitsui 2008 | Control | 22 | 21 | 54.5 | 67.4 | 10.6 | 25.6 | 2.5 | NR | NR | 18.6 |
|  | Intervention | 24 | 22 | 54.2 | 64 | 8.9 | 24.8 | 2.2 | NR | NR |  |
| Morgan 2010 | Control (Information and self-help) | 31 | 31 | 0 | 34 | 11.6 | 30.5 | 3.0 | NR | NR | NR |
|  | SHED-IT (Internet) group | 34 | 34 | 0 | 37.5 | 10.4 | 30.6 | 2.7 | NR | NR | NR |
| Muggia 2014 | Standard care group | 83 | 83 | 71.1 | 43.5 | 10.0 | 32.5 | 3.7 | NR | NR | NR |
|  | Brief CBT group | 80 | 80 | 76.3 | 46.2 | 11.7 | 31.9 | 3. | NR | NR | NR |
| Munsch 2003 | GP control | 17 | 122 | 58.8 | 45.2 | 23.9 | 32.6 | 1.8 | NR | NR | NR |
|  | Clinic BASEL | 52 |  | 76.9 |  |  | 38.5 | 7.5 | NR | NR | NR |
|  | GP BASEL | 53 |  | 79.2 |  |  | 36.2 | 6.5 | NR | NR | NR |
| Nakata 2014 | Control (N/A) | 63 | 63 | N/A | N/A | N/A | N/A | N/A | N/A | N/A | N/A |
|  | Education-only | 62 | 62 | 66 | 51.7 | 6.8 | 29.2 | 3.8 | NR | 0.0 | 29.0 |
|  | Group-based support | 63 | 63 | 81 | 50.7 | 6.7 | 29 | 3.0 | NR | 0.0 | 17.5 |
| Nanchahal 2012 | Usual care control | 190 | 190 | 72.6 | 49.4 | 14.1 | 33.0 | 5.4 | NR | 12.3 | NR |
|  | CAMWEL Intervention | 191 | 191 | 71.7 | 48.2 | 15.5 | 33.9 | 5.6 | NR |  | NR |
| Ng 2015 | Control group | 43 | 43 | 30.7 | 52 | 9.3 | 30.5 | 4.2 | NR | 25.6^#^ | 20.9 |
|  | Lifestyle modification program | 61 | 61 | 21.3 | 51.4 | 9.1 | 30.2 | 3.9 | NR | 23.0^#^ | 26.2 |
| Nicklas 2004 | Healthy lifestyle control | 78 | 78 | 68 | 69 | 0.1^b^ | 34.2 | 0.6 ^b^ | 35.9 | 11.5^#^ | 59.0 |
|  | Exercise only | 80 | 80 | 74 | 69 | 0.7 ^b^ | 34.2 | 0.6 ^b^ | 42.5 | 13.8^#^ | 67.5 |
|  | Diet only | 82 | 82 | 72 | 68 | 0.8 ^b^ | 34.5 | 0.6 ^b^ | 28.0 | 7.3^#^ | 62.2 |
|  | Diet plus exercise | 76 | 76 | 74 | 76 | 0.8 ^b^ | 34 | 0.7 ^b^ | 34.2 | 15.8^#^ | 57.9 |
| Nilsen 2011 | Control, Individual Physician Group | 104 | 104 | 47 | 45.9 | 11 | 35.9 | 6 | NR | NR | NR |
|  | Individual Plus Interdisciplinary Group | 109 | 109 | 53 | 47 | 11 | 37.6 | 6 | NR | NR | NR |
| Nordby 2012 | Control | 15 | 12 | 0 | 31 | 7 | 28 | 1.5 | NR | 0.0 | 0.0 |
|  | Training and increased diet (N/A) | 13 | N/A | N/A | N/A | N/A | N/A | N/A | N/A | N/A | N/A |
|  | Training | 17 | 12 | 0 | 28 | 5 | 28.3 | 1.1 | NR | 0.0 | 0.0 |
|  | Energy-reduced diet | 15 | 12 | 0 | 32 | 7 | 28 | 1.3 | NR | 0.0 | 0.0 |
| Nurkkala 2015 | Control | 30 | 17 | 77 | 46 | 10 | 35.8^a^ | 32.6, 39.4^a^ | NR | NR | NR |
|  | Intervention group | 90 | 59 | 71 | 46 | 10 | 34.3^a^ | 32.4, 38.9^a^ | NR | NR | NR |
| Oldroyd 2006 | Control group | 39 | 32 | 31.25 | 57.5 | 41 – 73^c^ | 29.9 | 4.9 | NR | NR | NR |
|  | Intervention group | 39 | 37 | 54.05 | 58.2 | 41 – 75^c^ | 30.4 | 5.6 | NR | NR | NR |
| Pan 1997 | Control | 138 | 138 | 43 | 46.6 | 9.3 | 26.2 | 3.8 | NR | 0.0 | NR |
|  | Intervention group (Exercise: n=155; Diet: n = 148; Diet plus exercise: n = 135) | 438 | 438 | 47 | 44.7 | 9.3 | 25.6 | 4.0 | NR | 0.0 | NR |
| Parikh 2010 | Control | 49 | 49 | 84 | 50 | 18 | 31 | 5.0 | NR | 0.0 | NR |
|  | Intervention | 50 | 50 | 86 | 46 | 15 | 32 | 4.0 | NR | 0.0 | NR |
| Patel 2016 | Control group | 50 | 50 | 82.0 | 44.9 | 10.6 | 37.3 | 6.1 | NR | NR | NR |
|  | Standard premium discount | 51 | 49 | 81.6 | 45.1 | 9.9 | 37.2 | 5.4 | NR | NR | NR |
|  | Immediate premium discount | 50 | 48 | 87.5 | 45.7 | 9.5 | 37.1 | 5.3 | NR | NR | NR |
|  | Daily lottery incentive | 50 | 49 | 76.0 | 43.9 | 9.2 | 36.1 | 4.3 | NR | NR | NR |
| Pekkarinen 2015 | Control, Follow up without intervention | 99 | 99 | 72 | 47.3 | 10.5 | 42.1 | 5.7 | NR | NR | NR |
|  | One-year maintenance program | 100 | 100 | 71 | 47.4 | 10.1 | 41.4 | 6.4 | NR | NR | NR |
| Perri 1984 | Non-behavioural therapy | 15 | 129 | 89.2 | 38.8 | NR | NR | NR | NR | NR | NR |
|  | Non-behavior therapy plus post-treatment contact | 16 |  |  |  | NR | NR | NR | NR | NR | NR |
|  | Behavior therapy | 21 |  |  |  | NR | NR | NR | NR | NR | NR |
|  | Behavior therapy plus relapse prevention training | 15 |  |  |  | NR | NR | NR | NR | NR | NR |
|  | Behavior therapy plus post-treatment contact | 15 |  |  |  | NR | NR | NR | NR | NR | NR |
|  | Behavior therapy plus relapse prevention training plus post-treatment contact | 17 |  |  |  | NR | NR | NR | NR | NR | NR |
| Perri 2001 | Control, Standard Behavioural Therapy (BT) | NR | 18 | 100 | 45.2 | 10.1 | 36.4 | 4.7 | NR | NR | NR |
|  | BT + Relapse prevention training | NR | 28 | 100 | 49.2 | 7.2 | 35 | 4.0 | NR | NR | NR |
|  | BT + problem-solving therapy | NR | 34 | 100 | 45.4 | 9.3 | 36.1 | 4.9 | NR | NR | NR |
| Perri 2014 | Control, Education group | 169 | 169 | 81.7 | 52 | 10.8 | 36.3 | 3.9 | NR | NR | NR |
|  | Low dose, (low intensity lifestyle counselling) | 148 | 148 | 75.7 | 51.5 | 12.3 | 36.1 | 4.2 | NR | NR | NR |
|  | Moderate dose, (Moderate intensity lifestyle counselling) | 134 | 134 | 81.3 | 52.8 | 10.6 | 36.2 | 3.8 | NR | NR | NR |
|  | High dose, (High intensity lifestyle counselling) | 161 | 161 | 74.5 | 53.2 | 12.0 | 36.7 | 4.0 | NR | NR | NR |
| Pettman 2009 | Control | 50 | 50 | 72 | NR | NR | 36.5 | 6.5 | NR | NR | NR |
|  | Intervention B - Passive follow-up | 54 | NR | NR | NR | NR | 37.3 | 6.2 | NR | NR | NR |
|  | Intervention A - Active follow-up | 49 | NR | NR | NR | NR | 36.1 | 6.6 | NR | NR | NR |
| Poelman 2015 | Control Condition | 139 | 139 | 84.2 | 45.4 | 9.2 | 32.9 | 5.0 | NR | NR | NR |
|  | Intervention condition | 139 | 139 | 84.9 | 45.9 | 9.2 | 32 | 4.6 | NR | NR | NR |
| Promrat 2010 | Control | 10 | 10 | 20 | 47.6 | 12.0 | 33.7 | 4.7 | NR | 40.0 | NR |
|  | Lifestyle Intervention | 21 | 21 | 33.3 | 48.9 | 10.9 | 33.9 | 5.3 | NR | 52.4 | NR |
| Provencher 2009 | Control group | 48 | 47 | 100 | 41.8 | 6.0 | 30.5 | 3.0 | NR | 0.0 | NR |
|  | Social support | 48 | 46 | 100 | 42.3 | 5.5 | 30.6 | 3.1 | NR | 0.0 | NR |
|  | Health-At-Every-Size | 48 | 48 | 100 | 42.8 | 5.5 | 30.1 | 3.0 | NR | 0.0 | NR |
| Rejeski 2011 | Successful aging control arm | 93 | 93 | 66.7 | 67.2 | 4.8 | 32.6 | 3.5 | 100 | 19.4 | 64.5 |
|  | Physical activity | 97 | 97 | 66.0 | 67.2 | 5.1 | 32.8 | 3.9 | 100 | 15.5 | 67.0 |
|  | Weight loss and physical activity | 98 | 98 | 68.4 | 66.8 | 4.6 | 33.1 | 4.1 | 100 | 16.3 | 74.5 |
| Ridgeway 1999 | Control | 28 | 20 | 75 | 65 | NR | NR | NR | NR | 100 | NR |
|  | Intervention Group | 28 | 18 | 61 | 62 | NR | NR | NR | NR | 100 | NR |
| Rolls 2005 | Comparison-control | 50 | 50 | NR | 45.2 | 1.2^b^ | 31.3 | 0.4^b^ | NR | NR | NR |
|  | Two snacks | 50 | 50 | NR | 44.5 | 1.2 ^b^ | 31.4 | 0.4^b^ | NR | NR | NR |
|  | One soup | 50 | 50 | NR | 45.1 | 1.2 ^b^ | 30.9 | 0.5^b^ | NR | NR | NR |
|  | Two soups | 50 | 50 | NR | 43.8 | 1.2 ^b^ | 30.8 | 0.5^b^ | NR | NR | NR |
| Rosas 2015 | Usual care | 41 | 41 | 78 | 47.6 | 10.5 | 34.9 | 4.4 | NR | 43.9^#^ | NR |
|  | Case-management intervention | 84 | 84 | 76.2 | 47.9 | 11.9 | 36 | 5.7 | NR | 44.0^#^ | NR |
|  | Case-management + Community health worker intervention | 82 | 82 | 76.8 | 46 | 10.7 | 35.5 | 5.1 | NR | 41.5^#^ | NR |
| Ross 2012 | Control condition | 241 | 241 | 70.1 | 52.4 | 11.8 | 32 | 4.2 | NR | NR | 33.2 |
|  | Behavioral intervention group | 249 | 249 | 70.28 | 51.3 | 11 | 32.6 | 4.1 | NR | NR | 25.7 |
| Samaras 1997 | Control | 13 | 13 | 53.8 | 60.5 | 2.1^b^ | 35.7 | 1.6^b^ | NR | NR | NR |
|  | Intervention | 13 | 13 | 69.2 | 60.5 | 7.8^b^ | 32.3 | 1.1^b^ | NR | NR | NR |
| Santanasto 2011 | Physical Activity plus Successful Ageing | 15 | 15 | 86.7 | 69.9 | 5.9 | 32 | 3.1 | NR | NR | NR |
|  | Physical Activity plus Weight Loss | 21 | 21 | 81 | 70.6 | 5.9 | 33.6 | 3.3 | NR | NR | NR |
| Sattin 2016 | Health Education intervention | 287 | 287 | 82.6 | 46.4 | 10.9 | 35.6 | 7.6 | NR | 0 | NR |
|  | Fit body and soul intervention | 317 | 317 | 84.2 | 46.6 | 10.9 | 35.8 | 7 | NR | 0 | NR |
| Schubel 2016 | Control group | 52 | 52 | 52 | 50.7 | 7.1 | 31.1 | 3.6 | NR | 0 | NR |
|  | Continuous Calorie Restriction | 49 | 49 | 49 | 50.5 | 8.0 | 31.2 | 4.0 | NR | 0 | NR |
|  | Intermittent Calorie Restriction | 49 | 49 | 49 | 49.4 | 9.0 | 32 | 3.8 | NR | 0 | NR |
| Seligman 2011 | Standard-of-care strategy | 25 | 25 | 32 | 42 | 8^b^ | 34.7 | 0.6^b^ | NR | NR | 52.0 |
|  | Healthy diet and step counter | 25 | 25 | 36 | 44 | 7^b^ | 34.4 | 0.6^b^ | NR | NR | 64.0 |
|  | Healthy diet and fitness | 26 | 25 | 36 | 43 | 8^b^ | 35.2 | 0.5^b^ | NR | NR | 64.0 |
| Snel 2012 | VLCD only | 14 | 14 | 38.5 | 56 | 2 | 37.9 | 1.4 | NR | 100 | NR |
|  | VLCD + exercise | 13 | 13 | 57.1 | 53 | 3 | 36.4 | 1.1 | NR | 100 | NR |
| Solbrig 2019 | Motivational interviewing | 58 | 55 | 72.72 | 42 | 19–70^c^ | 32.54 | 24.5–53.3^c^ | NR | NR | NR |
|  | Functional imagery training | 63 | 59 | 72.88 | 45 | 20–72^c^ | 33.21 | 26.0–48.0c | NR | NR | NR |
| Somers 2012 | Standard Care | 51 | 51 | 78 | 57.9 | 10.1 | 34.1 | 4.6 | NR | NR | NR |
|  | Lifestyle behavioral weight management intervention only | 59 | 59 | 80 | 58.3 | 11.0 | 33.5 | 4.4 | NR | NR | NR |
|  | Lifestyle behavioral weight management intervention + Pain Coping Skills Training | 62 | 62 | 92 | 57.5 | 9.43 | 34.1 | 4.3 | NR | NR | NR |
|  | Pain Coping Skills Training only (N/A) | 60 | 60 | N/A | N/A | N/A | N/A | N/A | N/A | N/A | N/A |
| Spring 2017 | Control self-guided program | 32 | 32 | 84.4 | 40.1 | 11.1 | 34.3 | 3.2 | NR | NR | NR |
|  | Standard weight loss program | 32 | 32 | 81.3 | 37.3 | 13.3 | 34.8 | 3.0 | NR | NR | NR |
|  | Technology-supported | 32 | 32 | 87.5 | 40.4 | 10.7 | 34.8 | 2.8 | NR | NR | NR |
| Stahre 2005 | Control | 43 | 43 | 100 | 45.2 | 11.3 | 39.2 | NR | NR | NR | NR |
|  | Cognitive treatment | 62 | 62 | 100 | 45.4 | 9.8 | 40.4 | NR | NR | NR | NR |
| Stahre 2007 | Control Group (weight-reducing program) | 27 | 16 | 100 | 47 | 8.2 | NR | NR | NR | NR | NR |
|  | Cognitive treatment group | 27 | 26 | 100 | 50.1 | 7.8 | NR | NR | NR | NR | NR |
| Stenius-Aarniala 2000 | Control | 19 | 19 | 68.7 | 48.3 | 23-60^c^ | 36.7 | 32.8-41.8^c^ | NR | NR | NR |
|  | Treatment with VLCD | 19 | 19 | 68.4 | 49.7 | 34-60^c^ | 35.8 | 31.3-39.4^c^ | NR | NR | NR |
| Stevens 1993 | Control | 256 | 256 | 37 | 42.4 | 6.2 | 29.5 | 2.8 | NR | NR | 0.0 |
|  | Intervention | 308 | 308 | 17 | 43.1 | 6.0 | 29.5 | 2.9 | NR | NR | 0.0 |
| Stevens 2001 | Control | 596 | 596 | 31.7 | 43.2 | 6.1 | 30.9 | 3.2 | NR | NR | NR |
|  | Intervention | 595 | 595 | 37 | 43.4 | 6.1 | 31 | 3.3 | NR | NR | NR |
|  | Sodium only intervention (N/A) | 594 | N/A | N/A | N/A | N/A | N/A | N/A | N/A | N/A | N/A |
|  | Combined intervention (N/A) | 597 | N/A | N/A | N/A | N/A | N/A | N/A | N/A | N/A | N/A |
| Strobl 2013 | Control, Usual care | 239 | 239 | 44 | 48.03 | 9.8 | 36.3 | 3.4 | NR | NR | NR |
|  | Telephone aftercare | 228 | 228 | 46 | 48.54 | 9.8 | 35.4 | 3.6 | NR | NR | NR |
| Tapsell 2017 | Usual care (Control) | 126 | 126 | 73 | 43.8 | 7.46 | 32.49 | 4.12 | NR | NR | 11.1 |
|  | Intervention Group | 125 | 124 | 73 | 43.79 | 7.97 | 32.59 | 4.25 | NR | NR | 16.1 |
|  | Intervention plus food supplement group (N/A) | 126 | N/A | N/A | N/A | N/A | N/A | N/A | N/A | N/A | N/A |
| TarragaMarcos 2017 | G3 | 55 | 55 | 32.4 | 49.8 | 6.6 | 30.7 | 3.4 | NR | NR | NR |
|  | G2 | 61 | 61 | 34.3 | 49.7 | 6.4 | 30.8 | 3.6 | NR | NR | NR |
|  | G1 | 60 | 60 | 33.3 | 50.1 | 7.2 | 30.3 | 3.2 | NR | NR | NR |
| Teeriniemi 2018 | Control | 89 | 89 | 51.7 | 46.5 | 10.2 | 30.5 | 2.3 | NR | 2.2^#^ | 25.8 |
|  | SHG Counselling | 87 | 87 | 48.3 | 44.4 | 10.2 | 30.7 | 2.2 | NR | 2.3^#^ | 26.4 |
|  | CBT Counselling | 85 | 85 | 49.4 | 46.4 | 9.7 | 30.5 | 1.9 | NR | 3.5^#^ | 20.0 |
|  | Control plus HBCSS | 91 | 91 | 47.3 | 47 | 9.4 | 30.3 | 2.0 | NR | 3.3^#^ | 22.0 |
|  | SHG Counselling plus HBCSS | 92 | 92 | 48.9 | 46.4 | 10.5 | 30.4 | 2.1 | NR | 1.1^#^ | 25.0 |
|  | CBT Counselling plus HBCSS | 88 | 88 | 50 | 44.8 | 9.6 | 30.3 | 2.1 | NR | 2.3^#^ | 15.9 |
| ter Bogt 2009 | GP usual care | 232 | 232 | 53.9 | 56.9 | 7.8 | 29.6 | 3.6 | NR | NR | 62.5 |
|  | Lifestyle counselling from NP | 225 | 225 | 49.8 | 55.3 | 7.7 | 29.5 | 3.1 | NR | NR | 60.9 |
| The Look AHEAD Research Group 2010 | Diabetes support and education | 2575 | 2575 | 59.6 | 58.9 | 6.9 | 36 | 5.8 | NR | 100.0 | 84.0 |
|  | Intensive lifestyle intervention | 2570 | 2570 | 59.3 | 58.6 | 6.8 | 35.9 | 6.0 | NR | 100.0 | 84.5 |
| Trepanowski 2017 | No-intervention control group | 31 | 31 | 87 | 44 | 11 | 34 | 4 | 0 | 0 | NR |
|  | Daily calorie restriction group | 35 | 35 | 83 | 43 | 12 | 35 | 4 | 0 | 0 | NR |
|  | Alternate-day fasting group | 34 | 34 | 88 | 44 | 10 | 34 | 4 | 0 | 0 | NR |
| Tsai 2010 | Control | 26 | 26 | 88 | 47.6 | 12.7 | 37.6 | 5.6 | NR | NR | NR |
|  | Brief counselling | 24 | 24 |  | 51.3 | 11.3 | 35.4 | 5.9 | NR | NR | NR |
| Tuomilehto 2009 | Control | 41 | 41 | 27 | 50.9 | 8.6 | 31.4 | 2.7 | NR | 7.3 | 36.6 |
|  | Intervention | 40 | 40 | 25.7 | 51.8 | 9.0 | 33.4 | 2.8 | NR | 10 | 45.0 |
| van de Glind 2017 | Comparison group | 553 | 553 | 0.0 | 45.6 | 8.7 | 33.4 | 4.7 | NR | NR | NR |
|  | EuroFIT group | 560 | 560 | 0.0 | 45.9 | 9.0 | 33.1 | 4.6 | NR | NR | NR |
| vanWier 2011 | Control – Brochure | 460 | 460 | 33.5 | 43 | 8.7 | 29.6 | 3.7 | 2.0 | 2.0^#^ | 10.0 |
|  | Internet Group | 464 | 464 | 34.9 | 43 | 8.4 | 29.6 | 3.4 |  |  |  |
|  | Phone Group | 462 | 462 | 30.5 | 43 | 8.8 | 29.5 | 3.5 |  |  |  |
| Vissers 2010 | Control | 21 | 21 | 74.7 | 44.8 | 11.4 | 30.8 | 3.4 | NR | NR | NR |
|  | Diet only group (Diet) | 20 | 20 |  | 45.5 | 13.1 | 32.9 | 3.1 | NR | NR | NR |
|  | Diet + fitness training group (Fitness) | 20 | 20 |  | 44.7 | 13 | 33.1 | 3.4 | NR | NR | NR |
|  | Diet + WBV group (Vibration) | 18 | 18 |  | 43.3 | 9.6 | 31.9 | 4.7 | NR | NR | NR |
| von Gruenigen 2012 | Control | 34 | 34 | 100 | 58.9 | 10.9 | 36.5 | 9.6 | NR | 26.5 | 35.3 |
|  | Intervention | 41 | 41 | 100 | 57.0 | 8.6 | 36.4 | 5.5 | NR | 14.1 | 31.8 |
| von Gruenigen 2008 | Control, Usual care | 22 | 22 | 100 | 55.4 | 7.5 | 41.1 | 10.3 | NR | NR | NR |
|  | Lifestyle intervention | 23 | 23 | 100 | 54.0 | 9.6 | 43.5 | 10.1 | NR | NR | NR |
| West 2007 | Attention control | 108 | 108 | NR | 52 | 10 | 36.5 | 5.4 | NR | 100.0 | NR |
|  | Motivational interviewing | 109 | 109 | NR | 54 | 10 | 36.5 | 5.5 | NR | 100.0 | NR |
| West 2011 | Control | 112 | 112 | 77 | 71.9 | 6.6 | 35.0 | 4.2 | NR | NR | NR |
|  | Lifestyle Intervention | 116 | 116 | 91 | 70.6 | 6.6 | 37.1 | 5.7 | NR | NR | NR |
| Whelton 1998 | Non-weight loss (Usual lifestyle, control group plus sodium reduction) | NR | NR | 57 | NR | NR | 31.1 | 2.4 | NR | NR | 100.0 |
|  | Weight loss (Weight loss alone plus weight loss and sodium reduction combined intervention) | NR | NR | 47 | NR | NR | 31.2 | 2.2 | NR | NR | 100.0 |
| Wilson 2016 | Control - Self Study Group | 242 | 147 | 59.9 | 46.6 | NR | 34.5 | NR | NR | NR | NR |
|  | Phone Fuel Your Life | 182 | 106 | 67.9 | 47.8 | NR | 33.6 | NR | NR | NR | NR |
|  | Group Fuel Your Life | 236 | 165 | 58.2 | 45.9 | NR | 32.7 | NR | NR | NR | NR |
| Wilson 2016b | Control | 457 | 457 | 6.3 | 47 | NR | 29.9 | 5.6 | NR | NR | NR |
|  | FUEL Your Life peer health coaches + nurse education | 459 | 459 | 5.4 | 44 | NR | 31.9 | 5.4 | NR | NR | NR |
| Wing 1998 | Control | 40 | 40 | 80 | 45.3 | 4.9 | 36.0 | 5.4 | NR | 0.0 | NR |
|  | Diet | 37 | 37 | 78 | 45.0 | 4.7 | 36.1 | 4.1 | NR | 0.0 | NR |
|  | Exercise | 37 | 37 | 81 | 46.4 | 4.5 | 36 | 3.7 | NR | 0.0 | NR |
|  | Diet plus exercise | 40 | 40 | 77 | 46.3 | 3.8 | 35.7 | 4.1 | NR | 0.0 | NR |
| Yannakoulia 2008 | Usual care group | 15 | 15 | 53.3 | 56.9 | 10 | 31.6 | 5 | NR | 100 | NR |
|  | Intensive care group | 15 | 15 | 40.0 | 56.3 | 8.8 | 32.2 | 4.1 | NR | 100 | NR |
| Yardley 2014 | Usual care | 43 | 43 | 65.1 | 49.9 | 13.8 | 36.2 | 4.9 | NR | NR | NR |
|  | Web-based only | 45 | 45 | 68.9 | 51.2 | 13.9 | 34.8 | 4.4 | NR | NR | NR |
|  | Basic nurse support | 44 | 44 | 65.9 | 51.4 | 13.0 | 36.4 | 6.5 | NR | NR | NR |
|  | Regular nurse support | 47 | 47 | 63.8 | 52.1 | 12.7 | 35.4 | 6.0 | NR | NR | NR |
| Yates 2009 | Control group | 34 | 29 | 41 | 65 | 10 | 29.8 | 4.4 | NR | NR | NR |
|  | PREPARE group | 31 | 29 | 31 | 64 | 7 | 29.5 | 4.9 | NR | NR | NR |
|  | PREPARE with pedometer | 33 | 29 | 31 | 66 | 8 | 28.7 | 4.8 | NR | NR | NR |
| Yates 2018 | Placebo + no lifestyle | 8 | 8 | 100 | 60 | 4.5 | 36.7 | 5.5 | 3.4 | NR | 50.0 |
|  | Metformin + no lifestyle (N/A) | 7 | 7 |  | N/A | N/A | N/A | N/A |  | N/A | N/A |
|  | Placebo + lifestyle | 7 | 7 |  | 57.1 | 3.3 | 39.7 | 5.1 |  | NR | 28.6 |
|  | Metformin + lifestyle (N/A) | 7 | 7 |  | N/A | N/A | N/A | N/A |  | N/A | N/A |
| Yeh 2016 | Control group | 30 | 30 | 50 | 60.9 | 12.2 | 25.8 | 2.3 | NR | NR | NR |
|  | Intervention group | 30 | 30 | 63.3 | 56.8 | 9.5 | 26.3 | 2.4 | NR | NR | NR |
| Yin 2018 | Comparison-Control Group | 75 | 75 | 100 | 53.27 | 7.17 | 27.43 | 2.75 | NR | NR | 24.0 |
|  | Intervention Group | 109 | 109 | 100 | 51.06 | 7.15 | 27.42 | 2.91 | NR | NR | 27.5 |
| Zhang 2016 | Control | 74 | 74 | 62.2 | 54 | 6.8 | 28 | 2.7 | NR | NR | NR |
|  | Moderate exercise | 73 | 73 | 69.9 | 54.4 | 7.4 | 28.1 | 3.3 | NR | NR | NR |
|  | Vigorous-moderate exercise | 73 | 73 | 71.2 | 53.2 | 7.1 | 27.9 | 2.7 | NR | NR | NR |
| **CBT:** Cognitive Behaviour Therapy; **CV** = Cardiovascular; **DM:** Diabetes Mellitus; **DPP:** Diabetes Prevention Program**; N/A** = Not applicable; **NR** = Not reported; **VLCD:** Very low calorie diet  * Comorbidity definitions varied for each study; ^#^Unclear whether DM percentage listed includes Type II and Type I  ^a^ Median (IQR); ^b^ Standard error; ^c^ Range; ^d^ 95% Confidence intervals | | | | | | | | | | | |

### Table 7. Intervention characteristics

| **Study ID** | **Groups:** | **Intervention type** | **Intervention faded in intensity** | **Features**  (meal replacements, nutrition education, financial incentives, intermittent fasting, content designed to help participants following programme end) | **Provider ^a^** | **Provider training received** | **Delivery** | | **Intervention setting** | **Intervention timing (months)** | | **Sessions** | | | **Intervention personalised, titrated or adapted** |
| --- | --- | --- | --- | --- | --- | --- | --- | --- | --- | --- | --- | --- | --- | --- | --- |
|  |  |  |  |  |  |  | **Mode** | **Format** |  | **Last contact** | **End**  **(step change in intensity)** | **N^c^** | **Frequency** | **Length per session with description for varying lengths.  (minutes** ^b^**)** |  |
| Abed 2013 | Control | Control | No | Nutrition Edu.; Help following programme end |  | No |  |  | Health Care |  |  | 0 |  |  | No |
|  | Weight Management | Diet and exercise | Yes | MR-P; Nutrition Edu.; Help following programme end | Physician | No | Individual | Face to Face; Print | Health Care | 15 | 2 | 5 | Every 3 months | 20 - 40 mins; Additional goal-directed face-to-face clinic visits were scheduled as required;  8 weeks VLCD. 3m to 15m low GI meals. Exercise plan of increasing intensity.  Exercise 3 per week for 15 months; 24-hour e-mail and telephone support provided as required. | Yes |
| Ackermann 2011 | Standard advice alone (controls) | Diet and exercise | No | Nutrition Edu. |  |  | Individual | Face to Face | Community | 0 | 0 | 1 | Once | 5 | Yes |
|  | YMCA DPP intervention | Diet and exercise | No | Nutrition Edu. |  |  | Individual and Group | Face to Face | Community | 5 | 5 | 16 | Weekly | 60 – 90 | Yes |
| Agras 1990 | Computer alone | Diet and exercise | No | Fin. Incentives |  | No | Other - remote, plus one group session | Other | Home | 3 | 3 |  | Daily | N/A as computer which they had on them at all times | Yes |
|  | Computer + group support | Diet and exercise | Yes | Fin. Incentives |  | Unclear | Other - computer and group support | Face to Face; Other | Community; Home | 3 | 3 | 4 | Every second week | 2, 4, 6 and 8 weeks | Yes |
|  | Behaviour therapy | Diet and exercise | No | Fin. Incentives |  | No | Group | Face to Face | Community | 3 | 3 | 10 | Approx. weekly | 10 sessions over 12 weeks | No |
| Ahern 2017 | Brief intervention | Control | No | Nutrition Edu. | Other | Unclear | Individual | Face to Face; Print | Community | 1 | 1 | 1 | Once |  | No |
|  | 12-week behavioural weight-loss programme | Diet and exercise | No | Nutrition Edu. | Health Trainer | Yes | Group | Face to Face; Internet | Community | 12 | 12 | 12 | Weekly | 30 | No |
|  | 52-week behavioural weight-loss programme | Diet and exercise | No | Nutrition Edu. | Health Trainer | Yes | Group | Face to Face; Internet | Community | 52 | 52 | 52 | Weekly | 30 | No |
| Almanza -Aguilera 2018 | Control (general recommendations) | Control | No | Nutrition Edu. | Nutritionist | No | Unclear | Face to Face; Other |  | 12 |  | 2 | 3m, 12m |  | No |
|  | Treatment (lifestyle weight loss intervention) | Diet and exercise | No | Nutrition Edu. | Nutritionist | No | Unclear | Face to Face; Other |  | 12 | 3 | 13 | Weekly (0-3m); Once at 12m |  | No |
| Anderson 2014 | Control (weight loss booklet only) | Control | No | Nutrition Edu.; Help following programme end |  | No | Individual | Print | Home | 12 | 12 | 0 |  |  | No |
|  | Intervention (BeWEL) | Diet and exercise | Yes | Nutrition Edu.; Help following programme end | Psychologist/ Counsellor | No | Individual | Face to Face; Telephone; Print | Health Care; Home | 3 | 12 | 12 | Monthly | The 3 counsellor sessions were each 1 hour. The 9 phone calls were each 15 minutes. | Yes |
| Appel 2011 | Control (Self-directed) | Control | No | Nutrition Edu. | Health Trainer | Unclear | Individual | Face to Face; Print | Health Care |  |  | 1 | 0, 24m |  |  |
|  | Remote Support Only (N/A) | N/A | N/A | N/A | N/A | N/A | N/A | N/A | N/A | N/A | N/A | N/A | N/A | N/A | N/A |
|  | In-Person Support | Diet and exercise | Yes | Nutrition Edu. | Other AHPs; Health Trainer | Yes | Individual and Group | Face to Face; Telephone; Internet; Other | Health Care | 24 | 6 | 57 | Weekly (0-3m); Three monthly contacts over the next 3 months; Two monthly contacts for the remainder of the study. | Individual sessions approx. 20 mins; In-person group sessions: 90 mins | Yes |
| Ard 2004 | “advice only” comparison group | Diet and exercise | No | Nutrition Edu. | Dietitian | No | Individual | Face to Face; Print; | Health Care | 6 | 6 | 2 |  | 30 | Yes |
|  | “established” behavioural intervention group | Diet and exercise | Yes | Nutrition Edu.; Help following programme end |  |  | Individual and Group | Face to Face | Health Care | 18 | 6 | 23 | Weekly (3m),  Biweekly (3m), Monthly (12m) |  | Yes |
|  | Established + DASH Intervention Group | Diet and exercise | Yes | Nutrition Edu.; Help following programme end |  |  | Individual and Group | Face to Face | Health Care | 18 | 6 | 23 | Weekly (3m), Biweekly (3m), Monthly (12m) |  | Yes |
| Ard 2018 | Exercise Only | Exercise only | No |  |  | Unclear | Group | Face to Face; Print | Community | 12 | 6 | 38 | Weekly 0-24 weeks and then biweekly until 12m | 1 hour | No |
|  | Exercise + Diet Quality + Weight Maintenance | Diet and exercise | No | Nutrition Edu. |  | Unclear | Group | Face to Face; Print; | Community | 12 | 6 | 38 | Weekly 0-24 weeks and then biweekly until 12m | 1 hour | No |
|  | Exercise + Diet Quality + Weight Loss | Diet and exercise | No | Nutrition Edu. |  | Unclear | Group | Face to Face; Print | Community | 12 | 6 | 38 | Weekly 0-24 weeks and then biweekly until 12 m | 1 hour | No |
| Ash 2006 | Control Group - Booklet only | Control | No | Nutrition Edu. |  | Yes | Other – booklet only | Print | Community |  |  | 0 |  |  | No |
|  | Individualised Dietetic Treatment | Diet and exercise | Yes | Nutrition Edu. | Dietitian | Yes | Individual | Face to Face; Telephone; Print | Health Care; Community | 6 | 2 | 12 | Weekly for 8 weeks, monthly from week 8 until 6m. | 1 hour initial individual consultation, followed by seven 20-min weekly review sessions and four monthly follow-up sessions | Yes |
|  | Fat Booters Incorporated | Diet and exercise | Yes | Nutrition Edu.; Help following programme end | Dietitian; Nutritionist | Yes | Group | Face to Face; Telephone; Print | Health Care; Community | 6 | 2 | 11 | Weekly for 6 weeks with monthly follow up until 6m. | 1.5 hrs for 6 sessions. Not stated for 5 follow up visits. | No |
| Aveyard 2016 | Advice only | Control | No |  | GP | Yes | Individual | Face to Face | Health Care | 0 | 0 | 1 |  | 30 seconds | No |
|  | Advice plus weight loss programme | Diet and exercise | No | Nutrition Edu. | GP; Health Trainer | Yes | Individual | Face to Face | Health Care; Community | 3 | 3 | 12 | Weekly | 60 | No |
| Azar 2013 | Control, Usual care | Control | No |  |  |  | Unclear | Face to Face | Community |  |  | 4 |  |  | No |
|  | Self-directed | Diet and exercise | Yes | Nutrition Edu. | Dietitian |  | Individual and Group | Face to Face; Internet; Other | Home | 15 | 3 | 40 |  |  | Yes |
|  | Coach-led | Diet and exercise | Yes | Nutrition Edu. | Psychologist/ Counsellor; Dietitian |  | Individual and Group | Face to Face | Community | 15 | 3 | 52 | Weekly | 90 – 120 | Yes |
| Bacon 2002 | Health at Every Size - control | Diet and exercise | No | Nutrition Edu. | Psychologist/ Counsellor |  | Group | Face to Face | Community | 12 | 6 | 30 | Weekly for first 6m, then monthly | 1.5 hour | No |
|  | Diet Group - intervention | Diet and exercise | No | Nutrition Edu. | Dietitian |  | Group | Face to Face | Community | 12 | 6 | 30 | Weekly for first 6m, then monthly | 1.5 hour | No |
| Barnes 2017 | Treatment as usual (N/A) | Control | N/A | N/A | N/A | N/A | N/A | N/A | N/A | N/A | N/A | N/A | N/A | N/A | N/A |
|  | Nutrition - ATTENTION CONTROL | Diet only | No | Nutrition Edu. | Other AHPs |  | Individual | Face to Face Internet; Print | Community | 3 | 3 | 5 | Every 3 weeks | 60 mins the first session and 20 mins the rest (4 sessions) |  |
|  | Motivational interviewing | Diet and exercise | No | Nutrition Edu. |  |  | Individual | Face to Face; Internet; Print | Community | 3 | 3 | 5 | Every 3 weeks | 60 mins the first session and 20 mins the rest (4 sessions) |  |
| Bartels 2015 | Control, Fitness club membership | Exercise only | No |  |  | No | Individual | Face to Face | Community |  |  | 1 |  |  | No |
|  | IN SHAPE | Diet and exercise | No | Nutrition Edu. | Health Trainer; Personal Trainer | Yes | Individual | Face to Face | Community | 12 | 12 | 52 | Weekly | 45 – 60 | Yes |
| Beavers 2017 | Weight loss | Diet only | Yes | Nutrition Edu.; Help following programme end |  | Yes | Individual and Group | Face to Face | Community | 18 | 6 | 48 | 4/m (1-6m);  3/m (7-12m);  1/m (12-18m) | 60 | Yes |
|  | Weight loss + Aerobic training | Diet and exercise | Yes | Nutrition Edu.; Help following programme end |  | Yes | Individual and Group | Face to Face | Community | 18 | 6 | 312 | 4/month (1-6m)  3/Month (7-12m); 1/Month (12-18m); 4 days/week exercises sessions (1-18m) | 60 minutes WL sessions; 45 exercise sessions | Yes |
|  | Weight loss + Resistance training | Diet and exercise | Yes | Nutrition Edu.; Help following programme end |  | Yes | Individual and Group | Face to Face | Community | 18 | 6 | 312 | 4/month (1-6m); 3/Month (7-12m); 1/Month (12-18m); 4 days/week exercises sessions (1-18m) | 60 minutes WL sessions; 45 exercise sessions | Yes |
| Bennett 2012 | Control, Usual care | Diet only | No |  |  | No | Other – Print only | Print |  |  |  |  |  |  | No |
|  | Be Fit, Be Well | Diet and exercise | Yes | Nutrition Edu. | Health care professional (not specified) | Yes | Individual and Group | Face to Face; Telephone; Internet | Community; Home | 24 | 12 | 30 | Monthly for the first year and bimonthly for the second year. Additional 12 optional monthly group sessions | 15-20 mins;  Telephone counselling sessions were held monthly for the first year and bimonthly for the second year. There were an additional 12 optional monthly group sessions | Yes |
| Bennett 2013 | Control, usual care | Control | No |  |  | No |  |  |  | 12 |  |  |  |  | No |
|  | Weight gain prevention intervention | Diet and exercise | No | Nutrition Edu. |  | Yes | Individual | Telephone; | Community; Home | 12 | 12 | 64 | Weekly (52) and monthly (12) | 10 mins (52 weekly IVR (interactive voice response calls))  12 monthly 20 min calls | Yes |
| Berry 2014 | Control | Control | No |  |  | No |  |  |  |  |  |  |  |  | No |
|  | Family based. Nutrition, exercise and coping skills intervention | Diet and exercise | No | Nutrition Edu. | Nurse (General); Dietitian; Personal Trainer | No | Group | Face to Face | Community | 12 | 3 | 21 | Weekly for 3m; Monthly until 12m | Weekly for 12 weeks. 60 mins nutrition and exercise education and coping skills followed by 45 mins of exercise. Monthly for 9m 60 mins class and 45 mins exercise. | No |
| Bertram 1990 | Control - diet only | Diet only | No | Nutrition Edu. |  | No | Individual | Print | Home | 4 | 4 | 0 |  |  | No |
|  | Diet plus lectures | Diet only | No | Nutrition Edu. | Dietitian | No | Group | Face to Face; Print | Community | 4 | 4 | 16 | Weekly | 1 hour | No |
|  | Diet plus exercise | Diet and exercise | No | Nutrition Edu. | Personal Trainer | No | Group | Face to Face; Print | Community | 4 | 4 | 64 | 3 x per week | 1 hour | No |
| Bertz 2012 | Control | Control | No |  |  | No |  |  |  |  |  |  |  |  | No |
|  | Diet Only | Diet only | No | Nutrition Edu. | Dietitian | No | Individual | Face to Face; Telephone; SMS | Health Care; Community; Home | 3 | 3 | 2 | Week 0, 6 | 1.5 hrs at start of intervention, 1hr at week 6 | Yes |
|  | Exercise only | Exercise only | No |  | Physiotherapist | No | Individual | Face to Face; Telephone; SMS | Health Care; Community; Home | 3 | 3 | 2 | Week 0, 6 | 1.5 hours at start of intervention, 1 hour at week 6 | No |
|  | Intervention | Diet and exercise | No | Nutrition Edu. | Dietitian; Physiotherapist | No | Individual | Face to Face; Telephone; SMS | Health Care; Community; Home | 3 | 3 | 4 | Week 0, 6 | 2 x 1.5 hours at start of intervention,  2 x 1 hour at week 6 | Yes |
| Bo 2007 | Control standard care | Control | No | Nutrition Edu. | GP | Yes | Individual | Face to Face | Health Care | 0 |  | 1 |  |  | No |
|  | Intervention lifestyle by trained professional | Diet and exercise | No | Nutrition Edu. | Physician; Nutritionist | Yes | Individual and Group | Face to Face; Print | Health Care; Community | 12 | 12 | 6 |  | 60 | Yes |
| Brown 2014 | Control | Control | No |  |  | No |  |  |  |  |  |  |  |  | No |
|  | RENEW | Diet and exercise | Yes | MR-P; Nutrition Edu. | Nurse (General); Other AHPs; Personal Trainer | No | Group | Face to Face; Telephone; Print | Health Care; Home | 12 | 3 | 63 | Weekly | Weeks 1-12 intensive intervention (weekly 3 hour intervention; 2 meal replacements per day, 1 hour exercise 2 x per week).  Weeks 13-24 maintenance phase (3 hour monthly session, 1 hour exercise 2 x per week, weekly phone calls, weekly newsletter). Weeks 25-52 intermittent support (weekly phone calls and monthly mailings). | No |
| Burke 2005 | Control usual care | Control | No |  |  | No |  | Face to Face; Print | Health Care |  |  |  |  |  |  |
|  | Low sodium + fish diet | Diet and exercise | No | Nutrition Edu. | Physician; Dietitian | No | Individual and Group | Face to Face; Telephone; Print; | Health Care; Home | 16 | 4 | 18 | 6 group plus individual (0-4m). Then, group: 2 x month for 1st month. 1 x month for 2 months. Then 1 x every 3m | Group = 90 mins. Length of individual sessions not stated. |  |
| Burke 2015 | Standard behavioural weight loss treatment | Diet and exercise | Yes | Nutrition Edu. | Health care professional (not specified) | No | Group | Face to Face | Community; Home | 18 | 12 | 20 | Weekly the first month, biweekly the second month, monthly for next 10 months, and every 6 weeks for 13-18m. | 1 hour group sessions |  |
|  | Self-efficacy enhancement plus standard behavioural weight loss treatment | Diet and exercise | Yes | Nutrition Edu. | Health care professional (not specified) | No | Individual and Group | Face to Face; Telephone | Community; Home | 18 | 12 | 50 | SE - 1:1 every 2 weeks for first 12m. Then at least monthly. SBT: weekly the first month, biweekly the second month, monthly for next 10 months, and every 6 weeks for months 13-18. | 1 hour group session. 1:1 sessions: 23 mins. |  |
| Cheyette 2007 | Control | Diet only | No | Nutrition Edu. | Dietitian | No | Individual | Face to Face | Health Care |  |  | 1 minimum | Annually |  | No |
|  | Weight No More intervention group | Diet and exercise | No | Nutrition Edu.; Help following programme end | Dietitian; Physiotherapist | Unclear | Group | Face to Face; Print | Community | 4 | 4 | 8 | Fortnightly | 1.5 hours | No |
| Christensen 2012 | Reference group | Control | No | Nutrition Edu. |  | No | Group | Face to Face |  | 12 |  | 12 | Monthly | 2 hours | No |
|  | Intervention group | Diet and exercise | No | Nutrition Edu.; Help following programme end |  | No | Group | Face to Face | Workplace | 12 | 3 | 48 | Weekly | 1 hour; Participants also instructed to spend additional personal time doing physical exercise (see 'Procedures') | Yes |
| Cleo 2018 | TTT Top Ten Tips habit formation | Diet and exercise | No |  |  | No | Group | Face to Face; Print | Community; Home | 3 | 3 | 13 | Weekly phone calls | 2 hrs group induction; Call length not stated. | No |
|  | DSD Do Something Different online software | Diet and exercise | No |  |  | No | Individual and Group | Face to Face; Telephone; Internet | Community; Home | 3 | 3 | 13 | Weekly tasks and phone calls | 2 hrs group induction. Call length not stated. tasks, length not stated | Yes |
|  | Wait list control (N/A) | N/A | N/A | N/A | N/A | N/A | N/A | N/A | N/A | N/A | N/A | N/A | N/A | N/A | N/A |
| Conroy 2015 | Self-guided | Diet and exercise | No | Nutrition Edu. | Other | No | Other - Self-guided manual | Print | Home | 3 | 3 |  |  | 12-week self-guided manual | No |
|  | Interventionist led | Diet and exercise | No | Nutrition Edu. | Physician; Other | No | Group | Face to Face | Health Care | 3 | 3 | 12 | Weekly | 60 mins | No |
| Cooper 2010 | Guided Self-Help Control | Control | No | Nutrition Edu. | Psychologist/ Counsellor | Yes | Individual | Face to Face; Telephone | Health Care; Home | 5.5 | 5.5 | 17 |  | 20 mins;  'lasted 24 weeks and involved two initial face-to-face sessions with a therapist followed by up to 15 20-min telephone sessions.' |  |
|  | Behaviour Therapy | Diet only | Yes | Nutrition Edu.; Help following programme end | Physician; Psychologist/ Counsellor; Dietitian | Yes | Individual | Face to Face | Health Care | 10 | 10 | 24 | Weekly for the first 7 weeks and every 2 weeks from week 8 to 44. | 50 mins; Weight loss phase lasted until week 24-30. |  |
|  | Cognitive Behaviour Therapy | Diet and exercise | Yes | Nutrition Edu; Help following programme end | Physician; Psychologist/ Counsellor; Dietitian | Yes | Individual | Face to Face | Health Care | 10 | 10 | 24 | Weekly for the first 7 weeks and every 2 weeks from 8 to week 44. | 50 mins;  Weight loss phase lasted until week 24-30. |  |
| Cousins 1992 | Control | Diet and exercise | No | Nutrition Edu.; Help following programme end |  | No | Other | Print | Home |  |  |  |  |  | No |
|  | Individual | Diet and exercise | Yes | Nutrition Edu.; Help following programme end | Dietitian | No | Group | Face to Face; Print | Community | 12 | 6 | 30 | 24 x weekly, then 6 x monthly |  | Yes |
|  | Family | Diet and exercise | Yes | Nutrition Edu. | Dietitian | No | Group | Face to Face; Print | Community | 12 | 6 | 30 | 24 x weekly, then 6 x monthly |  | Yes |
| Craighead 1989 | Control, minimal contact | Diet and exercise | No | Nutrition Edu. |  | No | Other - 12 written lessons with feedback | Print | Home | 12 | 6 |  |  |  | No |
|  | Contracted Exercise | Diet and exercise | No | Nutrition Edu. | Other | No | Group | Face to Face; Print | Community | 12 | 6 | 12 | Weekly | 60 | No |
|  | Supervised Exercise | Diet and exercise | No | Nutrition Edu. | Psychologist/ Counsellor; Other | No | Group | Face to Face; Print | Community | 12 | 6 | 33 | Weekly group meetings from week 5. 3 x per week from week 5 to 12 | 60 mins 1 x per week for 12 weeks. 40 mins 3 x per week for 8 weeks (from week 5 to week 12). | Yes |
| Dale 2009 | Control | Control | No |  |  | No |  |  |  |  |  |  |  |  | No |
|  | Modest | Diet and exercise | No | MR-F; Nutrition Edu. | Dietitian; Exercise physiologist; | Unclear | Individual and Group | Face to Face; Telephone; | Community | 4 | 4 | 36 | Twice weekly |  | Yes |
|  | Intensive intervention | Diet and exercise | No | Nutrition Edu. | Dietitian; Exercise physiologist | Unclear | Individual and Group | Face to Face; Telephone | Community | 4 | 4 | 36 | Twice weekly |  | Yes |
| Dalziel 2006 | Control | Control | No | Nutrition Edu. | Physician; Dietitian | No | Unclear | Face to Face | Health Care |  |  |  |  |  | No |
|  | Experimental | Diet only | No | Nutrition Edu. | Physician; Dietitian | No | Unclear | Face to Face | Health Care | 24 | 2 | 3 | At 8 weeks, then annually from baseline | 1 hour first session, length of follow-up sessions not reported. | No |
| Damschroder 2014 | Control, MOVE - usual care | Diet and exercise | Yes | Nutrition Edu. | Nurse (General); Psychologist/ Counsellor; Dietitian; Physiotherapist | Unclear | Group | Face to Face | Health Care | 24 | 3 | 58 | Weekly for 3m, then either quarterly or twice monthly | 11-12 weekly open-group sessions of 90 mins each over 3 months. During months 4-12, one group met quarterly for 90 minutes and the other groups met twice a month for 60 minutes. Some participants had the option of re-enrolling in the initial series of weekly sessions. Total hours over the year ranged from 22 to 35 hours. 12-24 mths as above for mths 4-12. | Yes |
|  | ASPIRE group, individual telephone counselling | Diet and exercise | Yes | Nutrition Edu. | Health Trainer | Yes | Individual | Telephone | Home | 24 | 3 | 34 | Same as ASPIRE-Group but duration of sessions varied | Up to 30 mins for the first 3 mths and 20 mins for the remaining 9 mths, totalling 11 hours across the year. 12-24 mths coaching every other mth, 6 sessions. | Yes |
|  | ASPIRE group, group counselling | Diet and exercise | Yes | Nutrition Edu. Help following programme end | Health Trainer | Yes | Group | Face to Face | Health Care | 24 | 3 | 34 | Both ASPIRE small-changes treatment arms consisted of weekly sessions for 3m, followed by 6m of sessions every other week, and then 3 monthly sessions over 12 months, for a total of 28 sessions. | Up to 90 mins for the first 3 mths and 60 mins for the remaining 9 mths, totalling 33 hours across the year. 12-24 mths coaching every other mth, 6 sessions. | Yes |
| Daumit 2013 | Control, Usual care | Control | No | Nutrition Edu. |  | No | Other |  |  |  |  | 1 |  |  |  |
|  | ACHIEVE | Diet and exercise | Yes | Nutrition Edu. | Health Trainer | Yes | Individual and Group | Face to Face | Health Care | 18 | 6 | 279 | 1-6m: Group weight management class weekly, individual visit monthly, group PA class 3x per week, weigh-in weekly; 7-18m: monthly group and individual class, group PA class 3x week, weigh in weekly | Group weight management class = 45 mins; Individual visit with interventionist 15-20 mins; group PA class 45 mins, weigh-in = 2 mins | Yes |
| deVos 2016 | Control | Control | No |  |  | No |  |  |  |  |  |  |  |  |  |
|  | Tailor-made lifestyle intervention | Diet and exercise | No | Nutrition Edu. | Dietitian; Physiotherapist | No | Individual and Group | Face to Face | Community | 30 | 6 | 23 + |  | First 3 dietician appointments were biweekly, after that the frequency of visits was determined by mutual agreement. Invited to attend 20 weekly physical activity classes.  "...definition of compliance as attendance at ≥ 6 dietitian visits and ≥7 physical activity classes." | Yes |
| Diabetes Prevention Program R G 2009 | Placebo | Diet and exercise | No | Nutrition Edu. | Other |  | Individual | Face to Face; Print |  | 36 | 36 | 4 | Annually | 20 – 30 |  |
|  | Metformin (N/A) | N/A | N/A | N/A | N/A | N/A | N/A | N/A | N/A | N/A | N/A | N/A | N/A | N/A | N/A |
|  | Lifestyle | Diet and exercise | Yes | MR-P; Nutrition Edu.; Help following programme end | Psychologist/ Counsellor; Dietitian; Exercise physiologist; Health Trainer | Yes | Individual and Group | Face to Face; Telephone; Print | Community | 36 | 6 | 358 | 16 sessions in first 24 weeks then monthly. [At least 2 exercise classes per week] | 45 mins;  Core curriculum sessions 30-60 mins;  Sessions: 16+6+12+12=46. [Physical activity 2x52 x 3 years = 312] | Yes |
| Djuric 2002 | Control | Control | No | Nutrition Edu. | Dietitian | No | Unclear | Print |  | 0 | 0 | 1 |  |  |  |
|  | Weight Watchers | Diet and exercise | No | Nutrition Edu. | Dietitian | Yes | Group | Face to Face | Community | 12 | 12 | 52 | Weekly |  |  |
|  | Individualized group | Diet and exercise | Yes | Nutrition Edu. | Dietitian |  | Individual and Group | Face to Face; Telephone; Print | Community | 12 | 3 | 21 | 0-3m: weekly;  3-6m: every other week;  6-12m: monthly |  | Yes |
|  | Comprehensive group | Diet and exercise | Yes | Nutrition Edu. | Dietitian |  | Individual and Group | Face to Face; Telephone | Community | 12 | 3 | 73 | Weekly |  | Yes |
| Duncan 2016 | Control | Control | No |  | GP | No | Individual | Face to Face | Health Care |  |  | 1 |  |  | No |
|  | Intervention | Diet and exercise | No | Nutrition Edu.; Help following programme end | Health Trainer | Yes | Individual | Face to Face | Home | 4 | 4 | 6 | Ranged from 1-4 weeks | 60 | Yes |
| Eakin 2014 | Usual care | Control | No | Nutrition Edu. |  | Unclear | Individual | Print | Health Care |  |  | 0 |  |  | No |
|  | Telephone intervention | Diet and exercise | Yes | Nutrition Edu. | Health Trainer | Yes | Individual | Telephone; Print | Health Care | 18 | 6 | 27 | 4 weekly calls; fortnightly calls for 5 months; monthly calls for 12 months. |  | Yes |
| Eaton 2016 | Control, Standard Intervention | Diet and exercise | Yes | Nutrition Edu. | Physician; Psychologist/ Counsellor | Yes | Individual | Face to Face; Print | Community; Home | 18 | 4 | 3 | In person sessions at 0, 6, 12m. Mailing at 1, 2, 4, 15 & 18m | 90 | No |
|  | Enhanced Intervention | Diet and exercise | Yes | Nutrition Edu. | Physician; Psychologist/ Counsellor; Dietitian | Yes | Individual | Face to Face; Telephone; Print; Video | Community; Home | 24 | 6 | 30 | 0-6m: weekly feedback & mailings, monthly phone, 2 DVDs.  6-12m: weekly mailings, bi-monthly phone.  12-18m: bimonthly mailings, 2 DVDs 1 phone.  18-24m: monthly mailing. | 90 | Yes |
| Fernandez-Ruiz 2018 | Control | Control | No |  |  | Unclear |  |  |  |  |  |  |  |  | No |
|  | Intervention (healthy eating, exercise & CBT) | Diet and exercise | No | Nutrition Edu. | Nurse (General); Physician; Psychologist/ Counsellor; Nutritionist; Exercise physiologist | Unclear | Individual and Group | Face to Face | Health Care | 12 | 12 | 232 | 4 x per week physical activity; monthly CBT & health ed. | 208 exercise sessions: 4 x per week, 40 mins. CBT 12 sessions, 1 per month, 60 mins. Health education (nurse) 12 sessions, 1 per month, 60 mins. | Yes |
| Foley 2016 | Usual care (Control) | Control | No |  |  | No | Other | Print | Health Care |  |  |  |  |  | No |
|  | Weight loss intervention | Diet and exercise | Yes | Nutrition Edu. | Psychologist/ Counsellor; Health care professional (not specified); Other | No | Individual | Face to Face; Telephone; Internet; App; Print; SMS | Health Care; Home | 12 | 3 | 18 | Calls 1-4: weekly;  Calls 5-10: biweekly  Calls 11-18: monthly |  | No |
| Foster-Schubert 2012 | Control- usual care | Control | No |  |  | No | Other – no contact |  |  |  |  |  |  |  |  |
|  | Calorie reduced diet | Diet only | Yes | Nutrition Edu. | Dietitian | No | Individual and Group | Face to Face; Telephone; Internet | Community; Home | 12 | 6 | 38 | 2 + 24 weekly 0-24. Then 2 per month (12) during weeks 24 – 52 |  | Yes |
|  | Aerobic exercise (N/A) | N/A | N/A | N/A | N/A | N/A | N/A | N/A | N/A | N/A | N/A | N/A | N/A | N/A | N/A |
|  | Intervention - diet and exercise | Diet and exercise | Yes | Nutrition Edu. | Dietitian; Exercise physiologist; | No | Individual and Group | Face to Face; Telephone; Internet | Community; Home | 12 | 6 | 194 | 3 per week exercise. Plus 38 diet sessions. | 45 | Yes |
| Fuller 2012 | Western diet group | Diet and exercise | No |  | Dietitian | No | Individual | Face to Face | Health Care; Community | 3 | 3 | 1 |  |  | No |
|  | Korean diet group | Diet and exercise | No | MR-F | Dietitian | No | Individual | Face to Face | Health Care; Community | 3 | 3 | 1 |  |  | No |
| Goodwin 2014 | Mailed-based intervention | Diet and exercise | No | Nutrition Edu. |  | No | Individual | Print | Community | 12 | 12 | 2 | 0, 12m |  | No |
|  | Individual lifestyle intervention | Diet and exercise | Yes | Nutrition Edu.; Help following programme end | Health Trainer | Unclear | Individual | Telephone; Print | Community | 12 | 6 | 19 | Weekly (0-1m); Biweekly (2-3m); Monthly (4-6m); every 2 months (7-12m); every 3 months (13-24m) | 30 – 60 | Yes |
| Green 2015 | Usual care | Control | No | Nutrition Edu. |  |  | Unclear |  |  |  |  |  |  |  | No |
|  | STRIDE | Diet and exercise | Yes | Nutrition Edu. | Psychologist/ Counsellor; Nutritionist |  | Individual and Group | Face to Face; Telephone; Print; | Health Care | 12 | 6 | 3 | Weekly: 0- 6m; Monthly: 6-12m | 120 | Yes |
| Grilo 2011 | Cognitive Behavioral Therapy (CBT) | Diet only | No | Nutrition Edu. | Psychologist/ Counsellor | Yes | Group | Face to Face | Health Care | 5.5 | 5.5 | 16 | 16 sessions over 24 weeks | 60 | No |
|  | Behavioral weight loss (BWL) | Diet and exercise | No | Nutrition Edu. | Psychologist/ Counsellor | Yes | Group | Face to Face | Health Care | 5.5 | 5.5 | 16 | 16 sessions over 24 weeks | 60 | No |
|  | CBT + BWL (N/A) | N/A | N/A | N/A | N/A | N/A | N/A | N/A | N/A | N/A | N/A | N/A | N/A | N/A | N/A |
| Grilo 2014 | Placebo | Control | No |  | Physician | Unclear |  |  |  | 4 | 4 | 0 |  |  | No |
|  | Placebo/CBTsh | Diet only | No | Nutrition Edu.; Help following programme end | Physician | Yes | Individual | Face to Face; Print | Health Care | 4 | 4 | 1 |  |  | No |
|  | Sibutramine (N/A) | N/A | N/A | N/A | N/A | N/A | N/A | N/A | N/A | N/A | N/A | N/A | N/A | N/A | N/A |
|  | Sibutramine/CBTsh (N/A) | N/A | N/A | N/A | N/A | N/A | N/A | N/A | N/A | N/A | N/A | N/A | N/A | N/A | N/A |
| Hanson 1976 | No treatment control condition (N/A) | N/A | N/A | N/A | N/A | N/A | N/A | N/A | N/A | N/A | N/A | N/A | N/A | N/A | N/A |
|  | Attention-placebo control condition | Control | No |  | Psychologist/ Counsellor | No |  |  |  |  |  | 0 |  |  | No |
|  | Conventional self-management condition | Diet only | No |  | Psychologist/ Counsellor | No | Group | Face to Face; Print |  | 2.5 | 2.5 | 10 | Weekly | 1 hour | No |
|  | Programmed text with low therapist-group contact | Diet only | No |  | Psychologist/ Counsellor | No | Group | Face to Face; Print |  | 2.5 | 2.5 | 3 | First meeting week 1, second meeting week 5, and 3 meeting week 10 |  | No |
|  | Programmed text with high therapist-group contact | Diet only | No |  | Psychologist/ Counsellor | No | Group | Face to Face; Print |  | 2.5 | 2.5 | 10 | Weekly | 1 hour | No |
| Hardcastle 2013 | Control | Diet and exercise | No | Nutrition Edu. | Nurse (General) | Yes | Other - Single appointment; provided leaflet | Print | Health Care | 6 | 6 | 1 | Once |  | No |
|  | MI counselling intervention | Diet and exercise | No | Nutrition Edu. | Psychologist/ Counsellor; Personal Trainer | Yes | Individual | Face to Face | Health Care | 6 | 6 | 5 | Anytime over a 6m period | 20 – 30 | Yes |
| Harrigan 2016 | Usual Care Group | Diet and exercise | No |  |  | Unclear | Unclear | Unclear; | Health Care |  |  | 2 |  |  | No |
|  | Telephone Weight Loss Counseling | Diet and exercise | Yes |  | Dietitian | No | Individual | Telephone | Home | 6 | 6 | 11 | Once per week (month 1), then every two weeks (months 2 and 3), and once per month (months 4, 5, and 6) | 30 | No |
|  | In-Person Weight Loss Counseling | Diet and exercise | Yes |  | Dietitian | No | Individual | Face to Face | Health Care | 6 | 6 | 11 | Once per week (month 1), then every two weeks (months 2 and 3), and once per month (months 4, 5, and 6) | 30 | No |
| Harris 2017 | Waist Winners Too | Diet and exercise | No | Nutrition Edu.; Help following programme end | Dietitian; Health care professional (not specified) | Yes | Individual | Face to Face | Health Care; Home | 12 | 6 | 15 | Every 2-3 weeks during weight loss phase (months 1-6); monthly during maintenance phase (months 7-12) | 40 – 60 | Yes |
|  | TAKE 5 | Diet and exercise | No | Nutrition Edu.; Help following programme end | Dietitian; Health care professional (not specified) | Yes | Individual | Face to Face | Health Care; Home | 12 | 6 | 15 | Every 2-3 weeks during weight loss phase (months 1-6); monthly during maintenance phase (months 7-12) | 40 – 60 | Yes |
| Hunt 2014 | Control, Wait-list | Control | No | Nutrition Edu. |  |  |  | Print |  |  |  |  |  |  |  |
|  | FFIT | Diet and exercise | Yes | Nutrition Edu.; Help following programme end | Personal Trainer | Yes | Group | Face to Face; Print | Community | 12 | 3 | 19 | Weekly for 12 weeks. 6 emails over 9 months. 1 reunion at 6 months. | 1.5 hours for 12 weekly sessions. Then 6 emails over 9m and 1 x reunion at 6m. | Yes |
| Huseinovic 2016 | Control Group | Diet only | No | Nutrition Edu. |  | No | Other - leaflet at baseline | Print; | Health Care | 0 | 0 |  |  |  | No |
|  | Diet behaviour modification Group | Diet and exercise | No | Nutrition Edu.; Help following programme end | Dietitian | Unclear | Individual | Face to Face; Telephone; Internet; Print; SMS | Health Care | 12 | 3 | 4 | Single face-to-face; biweekly SMS (3) followed by biweekly calls (3); monthly emails (9) | 1.5 hours single face-to-face session; Call length not stated. | Yes |
| Irwin 2003 | Control Group | Control | No |  |  |  |  |  |  | 0 | 0 | 0 | 0 | 0 | No |
|  | Exercise group | Exercise onl | Yes | Help following programme end | Exercise physiologist |  | Group | Face to Face | Community; Home | 12 | 3 | 36 | 3 times per week for the first 3 months and one per week the rest 9 months | 45 | No |
| Jackson 1982 | Control | Control | No |  | Other | No | Unclear | Face to Face | Community |  |  |  |  |  | Yes |
|  | Treatment | Diet and exercise | No | Nutrition Edu.; Fin. Incentives | Other | Unclear | Group | Face to Face; Print | Community | 4 | 4 | Mothers: 7 sessions; Participants: 6 sessions | Mothers: fortnightly; Participants: weekly | Mothers: 1 hr sessions; Participants: not reported | Yes |
|  | Cognitive-behavioral therapy | Diet and exercise | Yes | Nutrition Edu. | Psychologist/ Counsellor; Dietitian; Exercise physiologist | Unclear | Individual and Group | Face to Face; Telephone | Inpatient; Home | 7 | 7 | 44 | Inpatient phase (2 weekly nutrition education sessions, 5 weekly physical activity classes, 2 weekly psychotherapy sessions); Outpatient phase: 8 telephone psychotherapy sessions (2 sessions per month the first two mths after discharge and one session/mth for 4 months. | 45 min nutrition education; 45 min psychotherapy sessions; Telephone call length not stated. | Yes |
| Jakicic 2011 | Self Help Group | Exercise only | No |  |  | No | Other - provided manual and newsletters | Print; | Community | 18 |  | 1 |  | Monthly newsletter. | No |
|  | Moderate Physical Activity | Exercise only | Yes | Nutrition Edu. | Personal Trainer | Unclear | Individual and Group | Face to Face; Telephone; Print | Community | 18 | 6 | 156 | 4 x per week for 6 months, weekly contact between months 7 - 18 | Length of face-to-face sessions not reported; Telephone calls (< 10 mins) | No |
|  | High Physical Activity | Exercise only | Yes | Nutrition Edu. | Personal Trainer | Unclear | Individual and Group | Face to Face; Telephone; Print | Community | 18 | 6 | 156 | 4 x per week for 6 months, weekly contact between months 7 - 18 | Length of face-to-face sessions not reported; Telephone calls (< 10 mins)) | No |
|  | MAINTAIN Group (N/A) | N/A | N/A | N/A | N/A | N/A | N/A | N/A | N/A | N/A | N/A | N/A | N/A | N/A | N/A |
| Jebb 2011 | Standard care | Diet and exercise | No | Nutrition Edu. | GP | Yes | Individual | Face to Face | Health Care | 0 | 0 | 1 | Single session |  |  |
|  | Commercial programme | Diet and exercise | No | Nutrition Edu. | Health Trainer | Unclear | Group | Face to Face; Internet | Community | 12 | 12 | 52 | Weekly | 60 | Yes |
| Jebb 2017 | Usual care | Diet only |  | Nutrition Edu. | Nurse (General) | No | Individual | Face to Face; Print | Health Care | 3 | 3 | 6 to 12 | Weekly or biweekly |  | Yes |
|  | Low energy total diet replacement programme | Diet only | Yes | MR-F; Nutrition Edu.; Help following programme end | Health Trainer | Yes | Individual | Face to Face | Community | 6 | 6 | 15 | Weekly for first 12 weeks, then monthly |  | Yes |
| Jeffery 1995 | Control group | Control | No |  |  |  |  |  |  |  |  |  |  |  |  |
|  | Standard Behavioural Therapy (SBT) | Diet and exercise | Yes | Nutrition Edu. | Other | Yes | Group | Face to Face; Print |  | 18 | 4.6 | 33 | Weekly for 20 weeks; Monthly from week 20 to month 18, also encouraged to attend weekly weigh-in sessions |  | Yes |
|  | SBT + Incentives (I) | Diet and exercise | Yes | Nutrition Edu.; Fin. Incentives | Other | Yes | Group | Face to Face; Print |  | 18 | 4.6 | 33 | Weekly for 20 weeks; Monthly from week 20 to month 18, also encouraged to attend weekly weigh-in sessions |  | Yes |
|  | SBT + Food Provision (FP) | Diet and exercise | Yes | MR-P; Nutrition Edu. | Other | Yes | Group | Face to Face; Print |  | 18 | 4.6 | 33 | Weekly for 20 weeks; Monthly from week 20 to month 18, also encouraged to attend weekly weigh-in sessions; food provided for 5 days/week |  | Yes |
|  | SBT + FP + I | Diet and exercise | Yes | MR-P; Nutrition Edu.; Fin. Incentives | Other | Yes | Group | Face to Face; Print |  | 18 | 4.6 | 33 | Weekly for 20 weeks; Monthly from week 20 to month 18, also encouraged to attend weekly weigh-in sessions; food provided for 5 days/week |  | Yes |
| Jenkins 2017 | Control | Control | No | Nutrition Edu. |  | Unclear |  | Print | Health Care |  |  |  |  |  | No |
|  | Dietary advice only | Diet only | Yes | Nutrition Edu. |  | Unclear | Individual | Telephone; Print | Health Care | 6 | 6 | 9 | Weekly (Month 1); Monthly (Months 0-5) | 20 – 30 | Yes |
|  | Food basket only | Diet only | No |  |  | Unclear | Individual | Print; Other | Residential | 6 | 6 | Received 26 food baskets | Weekly |  | No |
|  | Food and advice | Diet only | Yes | Nutrition Edu. |  | Unclear | Individual | Telephone; Print; Other | Health Care | 6 | 6 | 9 plus 26 food baskets | Weekly (Month 1); Monthly (Months 0-5); weekly baskets | 20 – 30 | Yes |
| John 2011 | Control | Diet only | No | Nutrition Edu.; Help following programme end | Dietitian | Unclear | Individual | Face to Face | Community | 7.4 | 7.4 | 1 | single individual sessions; monthly weigh-ins | 1 hour | Yes |
|  | Deposit contracts group | Diet only | No | Nutrition Edu.; Fin. Incentives; Help following programme end | Dietitian | Unclear | Individual | Face to Face; Print; SMS | Community | 7.4 | 7.4 | 1 | single individual sessions; monthly weigh-ins; text messages | 1 hour | Yes |
| Jolly 2011 | Minimal intervention comparator | Exercise only | No |  |  |  | Individual | Other | Community |  |  | 12 |  |  | No |
|  | Choice (N/A) | N/A | N/A | N/A | N/A | N/A | N/A | N/A | N/A | N/A | N/A | N/A | N/A | N/A | N/A |
|  | Pharmacy | Diet and exercise | No | Nutrition Edu.; Help following programme end | Other AHPs | No | Individual | Face to Face | Health Care | 3 | 3 | 12 | Weekly (although may not have taken place weekly in all cases.) | 1st session 30 mins, follow up sessions 15-20 mins | Yes |
|  | General practice | Diet and exercise | No | Nutrition Edu.; Help following programme end | Nurse (General); GP | No | Individual | Face to Face | Health Care | 3 | 3 | 12 | Weekly (although may not have taken place weekly in all cases.) | 1st session 30 mins, follow up sessions 15-20 mins | Yes |
|  | Weight Watchers | Diet and exercise | No | Nutrition Edu. | Other | Yes | Individual and Group | Face to Face | Community | 3 | 3 | 12 | Weekly | 1 hour | No |
|  | NHS Size Down | Diet and exercise | Yes | Nutrition Edu. | Health Trainer | Yes | Group | Face to Face | Community | 3 | 3 | 8 | Weekly × 6 weeks; drop-in at 9 and 12 weeks. | 2 hours for weeks 1-6; Duration of drop in sessions is unclear. | No |
|  | Rosemary Conley | Diet and exercise | No | Nutrition Edu. | Other | Yes | Individual and Group | Face to Face; Telephone; Internet | Community | 3 | 3 | 12 | Weekly | 1.5 hours | Yes |
|  | Slimming world | Diet and exercise | No | Nutrition Edu. | Other | Yes | Individual and Group | Face to Face; Telephone; Internet; Print | Community | 3 | 3 | 12 | Weekly | 1.5 hours; Duration and frequency of telephone support unclear. | Yes |
| Jones 1986 | Individual | Diet only | No | Nutrition Edu.; Help following programme end | Dietitian | No | Individual | Face to Face | Health Care |  | 4 | 5 | Monthly | 10 | Yes |
|  | Group | Diet only | No | Nutrition Edu.; Help following programme end | Dietitian | No | Individual and Group | Face to Face | Health Care |  | 4 | 5 | Monthly | 60 | Yes |
|  | Leaflet Individual | Diet only | No | Nutrition Edu.; Help following programme end | Dietitian | No | Individual | Face to Face; Print | Health Care |  | 4 | 5 | Monthly | 10 | Yes |
|  | Leaflet Group | Diet only | No | Nutrition Edu.; Help following programme end | Dietitian | No | Individual and Group | Face to Face; Print | Health Care |  | 4 | 5 | Monthly | 60 | Yes |
|  | Diary Individual | Diet only | No | Nutrition Edu.; Help following programme end | Dietitian | No | Individual | Face to Face; Print | Health Care |  | 4 | 5 | Monthly | 10 | Yes |
|  | Diary Group | Diet only | No | Nutrition Edu.; Help following programme end | Dietitian | No | Individual and Group | Face to Face; Print | Health Care |  | 4 | 5 | Monthly | 60 | Yes |
|  | Leaflet Diary Individual | Diet only | No | Nutrition Edu.; Help following programme end | Dietitian | No | Individual | Face to Face; Print | Health Care |  | 4 | 5 | Monthly | 10 | Yes |
|  | Leaflet Diary Group | Diet only | No | Nutrition Edu.; Help following programme end | Dietitian | No | Individual and Group | Face to Face; Print | Health Care |  | 4 | 5 | Monthly | 60 | Yes |
| Jones 1999 | Control Group | Control | No |  | Nurse (General) | Unclear | Individual | Face to Face | Community | 0 | 0 | 1 | Single session |  | No |
|  | Weight Loss Group | Diet only | No | Nutrition Edu. | Dietitian | Unclear | Individual and Group | Face to Face | Community | 30 | 3 | 8 + 4 to 9 | Initial session, second session at 2-4 weeks, two monthly group sessions up to 3 months, following which group sessions are every 3-6 months |  | Yes |
| Katzer 2008 | Mail-delivered 'non-dieting' program (P3) | Diet and exercise | Yes | Nutrition Edu.; Help following programme end |  | No | Other – print only | Print | Home | 2.3 | 10.3 | 0 |  |  | No |
|  | Group 'non-dieting' program (P2) | Diet and exercise | Yes | Nutrition Edu.; Help following programme end | Psychologist/ Counsellor; Dietitian; Health Trainer | Unclear | Group | Face to Face | Community | 2.3 | 10.3 | 22 | Weekly for 10 weeks, fortnightly then monthly | 2 hours | No |
|  | Group 'non-dieting' program plus Relaxation (P1) | Diet and exercise | Yes | Nutrition Edu.; Help following programme end | Psychologist/ Counsellor; Nutritionist | Unclear | Group | Face to Face; Other | Community; Home | 2.3 | 10.3 | 22 | Weekly for 10 weeks, fortnightly then monthly | 2 hours | No |
| Kuller 2012 | Control - health education | Control | Yes |  |  |  |  |  | Community |  |  | 0 |  |  |  |
|  | Intervention - lifestyle change | Diet and exercise | Yes | Nutrition Edu. | Psychologist/ Counsellor; Nutritionist; Exercise physiologist | No | Group | Face to Face | Community | 36 | 6 | 64 | 1-6m: weekly  6-12m: every 2 weeks  12-36m: monthly |  | Yes |
| Leahey 2014 | SURI alone | Diet and exercise | No |  |  |  | Other – internet | Internet; Print |  | 3 | 3 | 0 |  |  | No |
|  | SURI plus Internet behavioral weight loss program | Diet and exercise | No | Nutrition Edu. |  |  | Other - internet | Face to Face; Internet; Print | Home | 3 | 3 | 12 | Weekly | 10 – 15 | No |
|  | SURI plus Internet behavioral weight loss program plus optional group sessions | Diet and exercise | No | Nutrition Edu. | Health Trainer | No | Group | Face to Face; Internet; Print; | Community; Home | 3 | 3 | 12 | Weekly | 10 – 15 | No |
| Ley 2004 | Control diet | Control | No |  |  |  |  |  |  |  |  |  |  |  |  |
|  | Reduced-fat | Diet only |  | Nutrition Edu. |  |  |  | Face to Face |  | 12 | 12 | 12 |  |  |  |
| Li 2016 | Usual care group | Control | Yes |  | Dietitian; Other | Yes |  | Print | Inpatient | 12 | 1 | 0 |  |  | Yes |
|  | Diet group | Diet only | Yes | Help following programme end | Dietitian; Other | Yes | Group | Face to Face; Telephone; Internet; Print | Inpatient | 12 | 1 | 36 | Six weekly sessions (Month 1); Monthly sessions (Months 1 - 12) |  | Yes |
|  | 50g-oats group | Diet only | Yes | Help following programme end | Dietitian; Other | Yes | Group | Face to Face; Telephone; Internet; Print | Inpatient | 12 | 1 | 36 | Six weekly sessions (Month 1); Monthly sessions (Months 1 - 12) |  | Yes |
|  | 100g-oats group | Diet only | Yes | Help following programme end | Dietitian; Other | Yes | Group | Face to Face; Telephone; Internet; Print | Inpatient | 12 | 1 | 36 | Six weekly sessions (Month 1); Monthly sessions (Months 1 - 12) |  | Yes |
| Lindstrom 2003 | Control | Control | No | Nutrition Edu. | Nurse (General); Physician; Nutritionist | Unclear | Other - group or individual | Face to Face; Print | Health Care |  |  | 1 |  | 30 mins to 1 hour | No |
|  | Intervention | Diet and exercise | Yes | MR-P; MR-F; Nutrition Edu.; Help following programme end | Nurse (General); Physician; Nutritionist; Physiotherapist | Unclear | Individual and Group | Face to Face; Telephone; Print | Health Care; Community | 48 | 12 | 19 | 7 sessions in first year then every 3 months | 30 mins to 1 hour | Yes |
| Liss 2016 | Standard care arm | Diet and exercise | No | Nutrition Edu. | Health care professional (not specified); Other | No | Individual | Face to Face; Print | Health Care | 12 |  | 3 | Every 6m | "brief" | Yes |
|  | Standard care plus group-based lifestyle intervention | Diet and exercise | Yes | Nutrition Edu.; Help following programme end | Health Trainer | Yes | Individual and Group | Face to Face; Print | Health Care; Community | 12 | 6 | 39 | Weekly for 6m; biweekly for next 6m | 3 brief; 60-to-90-minute intervention sessions | Yes |
| Little 2016 | Control, Nurse follow-up | Diet only | No | Nutrition Edu. | Nurse (General) | No | Other | Internet | Home |  |  |  | Data collection only at 6 and 12 months |  | No |
|  | Web-based support with minimal support (Remote) | Diet and exercise | No | Nutrition Edu.; Help following programme end | Nurse (General) | Unclear | Individual | Telephone; Internet; Other | Home | 6 | 6 | 29 | 24 web-based sessions designed to be used over 6 months. Three scheduled phone or email contacts and up to two optional phone or email contacts in the first 6 months |  | Yes |
|  | Web-based + nurse support (face to face) | Diet and exercise | No | Nutrition Edu.; Help following programme end | Nurse (General) | Unclear | Individual | Face to Face; Telephone; Internet; Other | Health Care; Home | 6 | 6 | 31 | 24 web-based sessions designed to be used over 6 months. Three scheduled face-to-face appointments in the first 3 months, and then up to four more appointments during a further 3 months if needed. |  | Yes |
| Ma 2015 | Control, Enhanced usual care | Control | No |  | Other AHPs | No | Individual | Print | Health Care |  |  | 0 |  |  | No |
|  | Diet and counselling | Diet and exercise | Yes | Nutrition Edu.; Help following programme end | Dietitian; Other AHPs; Personal Trainer | Yes | Individual and Group | Face to Face; Telephone | Health Care | 12 | 6 | 18 | Intensive stage: 13 weekly small group sessions over 4 months; Transitional stage: 1 individual counselling session in month 5 and another in month 6; Extended stage: 3 bi-monthly or more phone (participants can initiate contact with interventionists at any point and participants with weight gains of 1.4 to 2.2kg will be telephoned on a biweekly basis until they return to a stable, lower weight). | Group sessions 90-120 mins, transitional phase contacts 30-60 mins; extended sessions variable | Yes |
| Manzoni 2016 | Control, Standard behavioral inpatient program | Diet and exercise | No | Nutrition Edu. | Health care professional (not specified) | Unclear | Individual and Group | Face to Face; Telephone; Internet | Inpatient | 1.4 | 1.4 | 6 | Weekly nutritional groups held by dietitians |  | Yes |
|  | Cognitive–behavioral therapy | Diet and exercise | No | Nutrition Edu.; Help following programme end | Psychologist/ Counsellor; Dietitian; Health care professional (not specified) | Yes | Individual and Group | Face to Face; Telephone; Internet | Inpatient | 1.4 | 1.4 | 21 | Weekly and biweekly |  | Yes |
|  | CBT + Virtual reality | Diet and exercise | No | Nutrition Edu.; Help following programme end | Psychologist/ Counsellor; Dietitian; Health care professional (not specified) | Yes | Individual and Group | Face to Face; Telephone; Internet; Other | Inpatient | 1.4 | 1.4 | 36 | Weekly and biweekly | 60 | Yes |
| Marniemi 1990 | Control group | Control | No |  |  | No |  |  |  |  |  | 0 |  |  | No |
|  | Lactovegetarian weight reduction group | Diet only | No | Nutrition Edu. | Dietitian | No | Group | Face to Face |  | 12 | 2.5 | 15 | 10 weekly for 2.5 months and 5 throughout the year |  | No |
|  | Mixed diet weight reduction | Diet only | No | Nutrition Edu. | Dietitian | No | Group | Face to Face; |  | 12 | 2.5 | 15 | 10 weekly for 2.5 months and 5 throughout the year |  | No |
| Martin 2008 | Control, Standard Care | Control | No |  | Physician | Yes |  |  |  |  |  | 0 |  |  | Yes |
|  | Tailored physician/lifestyle counselling | Diet and exercise | No | Nutrition Edu.; Help following programme end | Physician | Yes | Individual | Face to Face; Print | Health Care | 6 | 6 | 6 | Monthly | 15 | Yes |
| Mefferd 2007 | Control | Control | No |  |  | Unclear | Unclear |  |  |  |  |  |  |  |  |
|  | Intervention | Diet and exercise | Yes | Nutrition Edu.; Help following programme end |  | Unclear | Individual and Group | Face to Face; Telephone | Health Care | 4 | 12 | 78 | 16 weeks of weekly closed group sessions followed by once-monthly sessions and then monthly sessions for an additional 6 months; Telephone contact: twice weekly during initial two weeks; weekly thereafter |  | Yes |
| Melchart 2017 | Control group | Diet and exercise | No | Nutrition Edu. | GP | No |  | Face to Face; Print | Health Care |  |  |  |  |  | Yes |
|  | Intervention group | Diet and exercise | Yes | Nutrition Edu.; Help following programme end | Health Trainer | Yes | Individual and Group | Face to Face; Internet | Health Care | 3 | 12 | 17 | 10 weekly; 3 full day ‘introduction days’ (reduction phase); 4 full day refresh training sessions (maintenance phase) | 7 full-day sessions; 10 x 2 hour sessions | Yes |
| Melin 2003 | Control, less intensively treated | Diet and exercise | No | Nutrition Edu.; Help following programme end | Physician; Psychologist/ Counsellor; Dietitian |  | Group | Face to Face | Health Care | 24 | 24 | 27 | 2 x per week during VLCD (2 periods 25 days) + every 3m |  | No |
|  | Intensively treated | Diet and exercise | Yes | Nutrition Edu.; Help following programme end | Physician; Psychologist/ Counsellor; Dietitian |  | Group | Face to Face | Health Care | 12 | 24 | 43 | 2 x per week during VLCD (2 periods 25 days) + every fortnight during the first year and 6 meetings during the second year. |  | No |
| Menard 2005 | Control - usual care | Control | No | Nutrition Edu. | Physician |  |  | Face to Face | Health Care |  |  | 6 | Every 6m | Health ed materials at 6, 12 and 18 mths, plus 3 phone calls |  |
|  | Intervention - intensive multitherapy | Diet and exercise | No | Nutrition Edu. |  |  | Individual | Face to Face; Telephone; Print | Health Care; Home | 12 | 12 | 36 | 3 x per month | 1 session plus at least 2 phone calls per month | Yes |
| Mensinger 2016 | Control, Weight Neutral Program | Diet and exercise | No | Nutrition Edu.; Help following programme end | Psychologist/ Counsellor; Personal Trainer |  | Group | Face to Face; Print; Other | Community | 6 | 6 | 26 | Weekly. | 90 |  |
|  | Weight Loss Program | Diet and exercise | No | Nutrition Edu.; Help following programme end |  |  | Group | Face to Face; Print; Other | Community | 6 | 6 | 26 | Weekly | 90 |  |
| Messier 2013 | Exercise only | Exercise only | Yes | Help following programme end | Personal Trainer | Yes | Unclear | Face to Face; Telephone | Community | 18 | 6 | 78 | 3 days per week | 60 | Yes |
|  | Diet-induced weight loss only | Diet only | Yes | MR-P; Nutrition Edu.; Help following programme end | Nutritionist | Yes | Individual and Group | Face to Face; Print | Community | 18 | 6 | 30 | 1-6 Months: individual session and 3 group sessions per month;  7-18 Months: biweekly group sessions and an individual session every 2 months |  | Yes |
|  | Diet-induced weight loss plus exercise | Diet and exercise | Yes | MR-P; Nutrition Edu.; Help following programme end | Nutritionist | Yes | Individual and Group | Face to Face; Telephone; Print | Community | 18 | 6 | 108 | 1-6 Months: individual session and 3 group sessions per month; 7-18 Months: biweekly group sessions and an individual session every 2 months plus 3 days/week (exercise) | 60 mins exercise; diet group sessions not reported | Yes |
| Miller 2002 | Control Group (Monitoring) | Control | No |  |  |  |  |  |  |  |  |  |  |  |  |
|  | Lifestyle Intervention | Diet and exercise | No | MR-P; Nutrition Edu.; Help following programme end | Personal Trainer |  | Group | Face to Face | Health Care; Community | 2 | 2 |  |  | 30 to 45 min for the exercise sessions | No |
| Mitsui 2008 | Control | Control | No |  |  | Unclear | Unclear |  | Health Care |  |  | 0 |  |  | No |
|  | Intervention | Diet and exercise | Yes | Nutrition Edu. | Dietitian | Unclear | Individual and Group | Face to Face | Health Care | 3 | 12 | 25 | Weekly: 0-12; Every other week: 13-26; Monthly 26-52 weeks | Exercise training: 40 minutes; Individual counselling sessions: not reported | Yes |
| Morgan 2010 | Control (Information and self-help) | Control | No | Nutrition Edu. | Other | Unclear | Group | Face to Face; Print | Community |  |  | 1 | Once | 60 | No |
|  | SHED-IT (Internet) group | Diet and exercise | Yes | Nutrition Edu. | Other | Unclear | Individual and Group | Face to Face; Internet; Print | Community; Home | 3 | 3 | 8 | Submit online daily eating and exercise diaries for the first 4 weeks, for 2 weeks in the second month and for 1 week in the third month. 7 x feedback | 1st session face to face group- 75 mins. The rest internet.  7 feedback sessions. Submit online daily eating and exercise diaries for the first 4 weeks, for 2 weeks in the second month and for 1 week in the third month. 28 + 14 + 7 = 49 | Yes |
| Muggia 2014 | Standard care group | Diet only | No | Nutrition Edu. |  | No | Individual | Print | Home | 12 | 6 | 7 | Control meetings every 3 months during the first year and every 6 months during the second year | 30 | No |
|  | Brief CBT group | Diet only | No | Nutrition Edu. |  | No | Group | Face to Face; Print | Health Care | 12 | 6 | 14 | 7 treatment sessions in a monthly basis. Then control meetings every 3 months during the first year and every 6 months during the second year | 90 | No |
| Munsch 2003 | GP control | Control | No |  | GP | Yes | Unclear | Face to Face | Health Care |  |  |  |  |  | No |
|  | Clinic BASEL | Diet and exercise | No | Nutrition Edu. | Physician; Psychologist/ Counsellor; Dietitian | Yes | Group | Face to Face; Print | Health Care | 4 | 4 | 16 | Weekly | 90 | No |
|  | GP BASEL | Diet and exercise | No | Nutrition Edu. | Physician; Other | Yes | Group | Face to Face; Print | Health Care | 4 | 4 | 16 | Weekly | 90 | No |
| Nakata 2014 | Control (N/A) | N/A | N/A | N/A | N/A | N/A | N/A | N/A | N/A | N/A | N/A | N/A | N/A | N/A | N/A |
|  | Education-only | Diet and exercise | No | Nutrition Edu. | Other | No | Group | Face to Face; Print | Health Care | 6 | 6 | 1 |  | 2 hours | No |
|  | Group-based support | Diet and exercise | Yes | Nutrition Edu. | Other | Yes | Group | Face to Face; Print | Health Care | 6 | 6 | 8 | Fortnightly (Weeks 1-6); Monthly (Weeks 6 - 22) | 2 hours | No |
| Nanchahal 2012 | Usual care control |  |  | Nutrition Edu. | GP | Yes | Individual | Face to Face; Print | Health Care |  |  |  |  |  | Yes |
|  | CAMWEL Intervention | Diet and exercise | Yes | Nutrition Edu.; Help following programme end | Health Trainer | Yes | Individual | Face to Face; Print | Health Care | 6 | 12 | 14 | Fortnightly for 12 weeks, 3-weekly to 27 weeks, 4-weekly to 35 weeks and a 12-week interval to the last session | 30 | Yes |
| Ng 2015 | Control group | Control | No | Nutrition Edu. | Physician | No | Individual | Face to Face | Health Care | 6 |  | 2 | Single sessions at baseline and at 6 months |  |  |
|  | Lifestyle modification program | Diet and exercise | Yes | Nutrition Edu.; Help following programme end | Dietitian | No | Individual | Face to Face | Health Care | 12 | 4 | 24 | Weekly (Months 1-4); Monthly (Months 5-12) | Encouraged to see an exercise instructor at least once during the program and perform 30 min of aerobic exercise two to three times a week. |  |
| Nicklas 2004 | Healthy lifestyle control | Diet and exercise | No | Nutrition Edu.; Help following programme end |  | Unclear | Group | Face to Face; Telephone | Community | 18 | 6 | 12 | Months 1-3: Monthly;  Month 4-6: Monthly phone contact; Months 7-18 bimonthly | 1 hour | No |
|  | Exercise only | Exercise only | Yes | Nutrition Edu.; Help following programme end | Exercise physiologist | Yes | Unclear | Telephone; Print | Community | 18 | 6 | 234 | 3 days per week | 1 hour | Yes |
|  | Diet only | Diet only | Yes | Nutrition Edu.; Help following programme end | Psychologist/ Counsellor; Dietitian | Yes | Individual and Group | Face to Face; Telephone; Print | Community | 18 | 6 | 59 | Months 1-4: Weekly; Months 4-6: Biweekly; Months 6-18: Monthly meetings and phone contacts every 2 weeks. |  | Yes |
|  | Diet plus exercise | Diet and exercise | Yes | Nutrition Edu.; Help following programme end | Psychologist/ Counsellor; Dietitian; Exercise physiologist | Yes | Individual and Group | Face to Face; Telephone; Print | Community | 18 | 6 | 293 | Diet component (Months 1-4: Weekly; Months 4-6: Biweekly; Months 6-18: Monthly meetings and phone contacts every 2 weeks.); Exercise component: 3 days per week | Exercise component: 1 hour | Yes |
| Nilsen 2011 | Control, Individual Physician Group | Diet and exercise | No | Nutrition Edu. | Physician |  | Individual | Face to Face | Health Care | 18 | 18 | 3 | 6 monthly |  |  |
|  | Individual Plus Interdisciplinary Group | Diet and exercise | Yes | Nutrition Edu.; Help following programme end | Nurse (General); Physician; Dietitian; Physiotherapist; Other |  | Individual and Group | Face to Face | Health Care; Community | 18 | 6 | 11 | Weekly (Weeks 5 to 10. Other sessions at week 3, 16, 20, 26 52 and 78 | 7 sessions of 5 hours; 1 x individual session 30 mins; 3 x physician consultation (30 mins? [3 x exercise test] | Yes |
| Nordby 2012 | Control | Control | No |  |  | Unclear |  |  | Community |  |  | 0 |  |  | No |
|  | Training and increased diet (N/A) | N/A | N/A | N/A | N/A | N/A | N/A | N/A | N/A | N/A | N/A | N/A | N/A | N/A | N/A |
|  | Training | Exercise only | No |  |  | Unclear | Unclear | Face to Face; Print | Community | 3 | 3 | 12 | Weekly | 3–4 sessions/week of continuous exercise at moderate intensity; d 3–4 sessions/week of continuous exercise with intermittent high intensity training intervals; weekly contact with a supervisor; extra supervision incorporated when required | Yes |
|  | Energy-reduced diet | Diet only | No | Nutrition Edu. |  | Unclear | Unclear | Face to Face | Community | 3 | 3 | 12 | Weekly | Weekly contact with a supervisor; extra supervision incorporated when required. |  |
| Nurkkala 2015 | Control | Diet and exercise | No | Nutrition Edu. | Nurse (General) | Unclear | Individual | Face to Face; Print | Health Care | 0 |  | 1 | Single session |  | Yes |
|  | Intervention group | Diet and exercise | No | Nutrition Edu.; Help following programme end | Nurse (General); Nutritionist | Unclear | Individual | Face to Face | Health Care | 36 | 9 | 20 | 14 (Year 1); 4 (Year 2); 2 (Year 3) |  | Yes |
| Oldroyd 2006 | Control group | Control | No |  |  |  |  |  |  |  |  |  |  |  |  |
|  | Intervention group | Diet and exercise | No | Nutrition Edu. | Dietitian; Physiotherapist | No | Individual | Face to Face; Print | Community | 24 | 6 | 12 | In the first 6 months there were three such appointments at two weekly intervals, followed by three at monthly intervals. There was one after 9 months and five at two monthly intervals between 12 and 24 months. | 15 – 20 | Yes |
| Pan 1997 | Control | Control | No | Nutrition Edu. | Physician | Unclear | Unclear | Print | Health Care |  |  |  |  |  | No |
|  | Intervention group (Exercise: n=155; Diet: n = 148; Diet plus exercise: n = 135) |  | Yes | Nutrition Edu. | Physician | Unclear | Individual and Group | Face to Face; Print | Health Care | 72 | 72 | 30 - 60 | Frequency of group dietary counselling and exercise sessions: weekly for 1 month, monthly for 3 months, and then once every 3 months for the remainder of the study. |  | Yes |
| Parikh 2010 | Control | Control |  |  |  |  | Unclear |  | Health Care |  |  |  |  |  |  |
|  | Intervention | Diet and exercise | No | Nutrition Edu. |  | Yes | Group | Face to Face; Print | Community | 3 | 3 | 8 | 8 sessions over 10 weeks | 1.5 hours | No |
| Patel 2016 | Control group | Control | No |  | Other | No | Unclear | Internet | Workplace |  |  |  |  |  | Yes |
|  | Standard premium discount |  | No | Fin. Incentives | Other | No | Unclear | Internet | Workplace | 6 | 6 |  |  |  | Yes |
|  | Immediate premium discount |  | No | Fin. Incentives | Other | No | Unclear | Internet | Workplace | 6 | 6 |  |  |  | Yes |
|  | Daily lottery incentive |  | No | Fin. Incentives | Other | No | Unclear | Internet | Workplace | 12 | 12 |  |  |  | Yes |
| Pekkarinen 2015 | Control, Follow up without intervention | Diet and exercise | No | MR-F; Nutrition Edu.; Help following programme end | Nurse (General); Nutritionist; Other | Yes | Group | Face to Face; Print | Health Care | 4 | 4 | 17 | Weekly | 1.5 hours |  |
|  | One-year maintenance program | Diet and exercise | Yes | MR-F; Nutrition Edu.; Help following programme end | Nurse (General); Nutritionist; Other | Yes | Group | Face to Face; Print | Health Care | 16 | 4 | 29 | Weekly and the monthly. | 1.5 hours |  |
| Perri 1984 | Non-behavioural therapy | Control | No | Nutrition Edu. | Psychologist/ Counsellor | No | Group | Face to Face | Community | 3 | 3 | 18 | 15 weekly and 3 post-treatment fu | 2 hours | No |
|  | Non-behavior therapy plus post-treatment contact | Diet only | No | Nutrition Edu.; Help following programme end | Psychologist/ Counsellor | No | Group | Face to Face; Telephone; Print | Community | 9 | 3 | 18 | 15 weekly and 3 post-treatment fu PLUS 5-10min phone calls up to 9 months | 2 hours | No |
|  | Behavior therapy | Diet only | No | Nutrition Edu. | Psychologist/ Counsellor | No | Group | Face to Face | Community | 3 | 3 | 18 | 15 weekly and 3 post-treatment fu | 2 hours | No |
|  | Behavior therapy plus relapse prevention training | Diet only | No | Nutrition Edu. | Psychologist/ Counsellor | No | Group | Face to Face; Telephone | Community | 3 | 3 | 18 | 15 weekly and 3 post-treatment fu | 2 hours | No |
|  | Behavior therapy plus post-treatment contact | Diet only | No | Nutrition Edu.; Help following programme end | Psychologist/ Counsellor | No | Group | Face to Face; Telephone; Print | Community | 9 | 3 | 18 | 15 weekly and 3 post-treatment fu PLUS 5-10min phone calls up to 9 months | 2 hours | No |
|  | Behavior therapy plus relapse prevention training plus post-treatment contact | Diet only | No | Nutrition Edu.; Help following programme end | Psychologist/ Counsellor | No | Group | Face to Face; Telephone; Print | Community | 9 | 3 | 18 | 15 weekly and 3 post-treatment fu PLUS 5-10min phone calls up to 9 months | 2 hours | No |
| Perri 2001 | Control, Standard Behavioural Therapy (BT) | Diet and exercise | No | Nutrition Edu. | Psychologist/ Counsellor |  | Group | Face to Face | Community | 5 | 5 | 20 | Weekly | 2 hours |  |
|  | BT + Relapse prevention training | Diet and exercise | Yes | Nutrition Edu.; Help following programme end | Psychologist/ Counsellor |  | Group | Face to Face; Print | Community | 17 | 5 | 46 | Weekly and biweekly | 2 hours | Yes |
|  | BT + problem-solving therapy | Diet and exercise | Yes | Help following programme end | Psychologist/ Counsellor |  | Group | Face to Face; Print | Community | 17 | 5 | 46 | Weekly and biweekly | 2 hours | Yes |
| Perri 2014 | Control, Education group | Diet and exercise |  | Nutrition Edu. | Other | Yes | Group | Face to Face; Telephone | Community; Home | 24 | 6 | 21 | Weekly |  | No |
|  | Low dose, (low intensity lifestyle counselling) | Diet and exercise | Yes | Nutrition Edu.; Help following programme end | Other | Yes | Individual and Group | Face to Face; Telephone | Community; Home | 24 | 6 | 21 | Weekly |  | Yes |
|  | Moderate dose, (Moderate intensity lifestyle counselling) | Diet and exercise | Yes | Nutrition Edu.; Help following programme end | Other | Yes | Individual and Group | Face to Face; Telephone | Community; Home | 24 | 6 | 42 | Weekly |  | Yes |
|  | High dose, (High intensity lifestyle counselling) | Diet and exercise | Yes | Nutrition Edu.; Help following programme end | Other | Yes | Individual and Group | Face to Face; Telephone | Community; Home | 24 | 6 | 63 | Weekly |  | Yes |
| Pettman 2009 | Control | Control | No | Nutrition Edu. |  | No | Individual | Print |  |  |  |  |  |  | No |
|  | Intervention B - Passive follow-up | Diet and exercise | No | Nutrition Edu. | Health Trainer | Unclear | Group | Face to Face; Telephone; Other | Community | 4 | 12 | 32 | Weekly group session and exercise session | 2 hour group sessions: 1 hour exercise session. | No |
|  | Intervention A - Active follow-up | Diet and exercise | Yes | Nutrition Edu.; Help following programme end | Health Trainer | Unclear | Group | Face to Face; Telephone; Other | Community | 4 | 12 | 40 | Weekly group session and exercise session | 2 hour group sessions;  1 hour exercise session. | No |
| Poelman 2015 | Control Condition | Control | No |  |  |  |  |  |  |  |  | 0 |  |  | No |
|  | Intervention condition | Diet only | No | Nutrition Edu. | Dietitian; Health care professional (not specified) | No | Individual and Group | Face to Face; Internet | Community | 12 | 3 | 3 | Biweekly | 3 hour cooking class, 8-minute video | No |
| Promrat 2010 | Control | Diet and exercise | No | Nutrition Edu. | Nutritionist; Health Trainer | Unclear | Group | Face to Face | Health Care | 12 | 12 | 4 | Once every 12 weeks. |  |  |
|  | Lifestyle Intervention | Diet and exercise | Yes | Nutrition Edu.; Help following programme end | Nutritionist; Health Trainer | Unclear | Individual and Group | Face to Face | Health Care | 12 | 6 | 36 | Months 1-6: weekly; Months 7-12: biweekly |  | Yes |
| Provencher 2009 | Control group | Control | No |  |  |  |  |  |  |  |  |  |  |  |  |
|  | Social support | Diet only | No | Nutrition Edu. | Psychologist/ Counsellor; Dietitian | Yes | Group | Face to Face | Community | 4 | 4 | 14 | Weekly | 2 hours | Yes |
|  | Health-At-Every-Size | Diet and exercise | No | Nutrition Edu. | Psychologist/ Counsellor; Dietitian | Yes | Group | Face to Face; Print | Community | 4 | 4 | 14 | Weekly | "13 three-hour evening sessions and 1 intensive-day session of 6 hours)." | Yes |
| Rejeski 2011 | Successful aging control arm | Control |  |  |  | No |  |  |  |  |  | 18 |  |  | No |
|  | Physical activity | Exercise only | Yes | Help following programme end | Health Trainer | Unclear | Individual and Group | Face to Face; Telephone | Community; Home | 18 | 6 | 48 |  | Group sessions lasted 90 mins, and individual sessions lasted 30 mins. Phone session and the last group session lasted 10-20 mins. | Yes |
|  | Weight loss and physical activity | Diet and exercise | Yes | Nutrition Edu.; Help following programme end | Dietitian; Health Trainer | Unclear | Individual and Group | Face to Face; Telephone | Community; Home | 18 | 6 | 48 |  |  | Yes |
| Ridgeway 1999 | Control | Control | No |  |  | No |  |  |  |  |  |  |  |  | No |
|  | Intervention Group | Diet and exercise | No | Nutrition Edu. | Nurse (General); Dietitian | Unclear | Individual and Group | Face to Face; Print | Health Care | 12 | 6 | 7 | Monthly; Plus single follow-up session at 12 months | 90 minutes group sessions | Yes |
| Rolls 2005 | Comparison-control | Control | No |  |  | No |  |  |  |  |  |  |  |  |  |
|  | Two snacks | Diet and exercise | No | MR-P; Nutrition Edu.; Help following programme end | Dietitian | No | Individual | Face to Face |  | 12 | 6 | 24 | Weekly from 1 to 3 months, fortnightly 4 to 6 months, and monthly from 7 to 12 months | 15 – 30 | No |
|  | One soup | Diet and exercise | No | MR-P; Nutrition Edu.; Help following programme end | Dietitian | No | Individual | Face to Face |  | 12 | 6 | 24 | Weekly from 1 to 3 months, fortnightly 4 to 6 months, and monthly from 7 to 12 months | 15 – 30 | No |
|  | Two soups | Diet and exercise | No | MR-P; Nutrition Edu.; Help following programme end | Dietitian | No | Individual | Face to Face |  | 12 | 6 | 24 | Weekly from 1 to 3 months, fortnightly 4 to 6 months, and monthly from 7 to 12 months | 15 - 30 | No |
|  | Case-management intervention |  | Yes | Nutrition Edu.; Help following programme end | Health Trainer | No | Individual and Group | Face to Face | Health Care | 24 | 6 | 20 | 16 sessions from 0-12 months. 4 sessions from 12-24 months. | Group sessions last 2 hours, individual sessions last 30 minutes. | Yes |
|  | Case-management + Community health worker intervention |  | Yes | Nutrition Edu.; Help following programme end | Health Trainer | No | Individual and Group | Face to Face | Health Care; Community;Home | 24 | 6 | 27 | Same as CM group, with additional 5 home visits from 0-12 months and 2 home visits from 12-24 months. | Same as CM group. The length of the additional CHW home visits is not clear. | Yes |
| Rosas 2015 | Usual care |  | No |  | GP | No | Individual | Face to Face | Health Care |  |  |  |  |  | No |
|  | Case-management intervention |  | Yes | Nutrition Edu.; Help following programme end | Health Trainer | No | Individual and Group | Face to Face | Health Care | 24 | 6 | 20 | 16 sessions from 0-12 months. 4 sessions from 12-24 months. | Group sessions last 2 hours, individual sessions last 30 minutes. | Yes |
|  | Case-management + Community health worker intervention |  | Yes | Nutrition Edu.; Help following programme end | Health Trainer | No | Individual and Group | Face to Face | Health Care; Community;Home | 24 | 6 | 27 | Same as CM group, with additional 5 home visits from 0-12 months and 2 home visits from 12-24 months. | Same as CM group. The length of the additional CHW home visits is not clear. | Yes |
| Ross 2012 | Control condition | Control | No | Nutrition Edu. | Physician | No | Unclear | Face to Face | Health Care |  |  |  | Usual schedule (typically once a year). |  | Yes |
|  | Behavioral intervention group | Diet and exercise | No | Nutrition Edu.; Help following programme end | Health Trainer | Yes | Individual and Group | Face to Face |  | 24 | 6 | 33 | First 6m: 8 sessions in first 6 weeks, then every 2 weeks. Months 7-24, monthly sessions. | (0–6 months, 15 sessions, 15 hours); Months 7–12 (6 sessions, 3–6 hours); Months 13–24 (12 sessions, 6–12 hours) | Yes |
| Samaras 1997 | Control | Control | No |  |  |  |  |  |  |  |  |  |  |  |  |
|  | Intervention | Exercise only | No | Help following programme end | Nurse (General); Physician; Dietitian; Exercise physiologist; Other | Yes | Group | Face to Face; Print; Video | Community | 6 | 6 | 6 | Monthly | 1 hour | Yes |
| Santanasto 2011 | Physical Activity plus Successful Ageing | Diet only | Yes | Nutrition Edu.; Help following programme end |  | Unclear | Individual and Group | Face to Face | Community; Home | 12 | 6 | 68. | Monthly SA session; Exercise sessions: 3 x sessions/week (Weeks 1 – 8); two sessions/week (weeks 9–24); optional exercise session at the center once per week (weeks 25–52) | 60 min exercise sessions | No |
|  | Physical Activity plus Weight Loss | Diet and exercise | Yes | Nutrition Edu.; Help following programme end | Nutritionist | Unclear | Individual and Group | Face to Face | Community; Home | 12 | 6 | 87 | Nutrition sessions: 24 weekly, 2 bimonthly, and 5 monthly; Exercise sessions: 3 x sessions/week (Weeks 1 – 8); two sessions/week (weeks 9–24); optional exercise session at the center once per week (weeks 25–52) | 60 minutes exercise sessions | No |
| Sattin 2016 | Health Education intervention | Control | No | Nutrition Edu. | Health care professional (not specified) | Yes | Group | Face to Face | Community | 9 | 3 | 18 | Weekly for the first 12 weeks and monthly for the remaining 6m |  | Yes |
|  | Fit body and soul intervention | Diet and exercise | No | Nutrition Edu.; Help following programme end | Health care professional (not specified) | Yes | Group | Face to Face | Community | 9 | 3 | 18 | Weekly for the first 12 weeks and monthly for the remaining 6m |  | Yes |
| Schubel 2016 | Control group | Control | Yes | Nutrition Edu. | Dietitian; Nutritionist | Yes | Individual | Face to Face; Telephone | Community | 11.5 | 3 | 8 | Biweekly phone calls (Week 1-12); Two single sessions at the beginning and end of the Intervention phase | "The number of personal contacts and counseling sessions was the same for all study participants overall, but individuals in the ICR and CCR arms received longer and more comprehensive counseling sessions with personalized dietary plans, specific for the ICR or CCR regimens." | No |
|  | Continuous Calorie Restriction | Diet only | Yes | Nutrition Edu.; Help following programme end | Dietitian; Nutritionist | Yes | Individual | Face to Face; Telephone; Print | Community | 11.5 | 3 | 8 | Biweekly phone calls (Week 1-12); Two single sessions at the beginning and end of the Intervention phase | "The number of personal contacts and counseling sessions was the same for all study participants overall, but individuals in the ICR and CCR arms received longer and more comprehensive counseling sessions with personalized dietary plans, specific for the ICR or CCR regimens." | Yes |
|  | Intermittent Calorie Restriction | Diet only | Yes | Nutrition Edu.; Inter. Fasting; Help following programme end | Dietitian; Nutritionist | Yes | Individual | Face to Face; Telephone; Print | Community | 11.5 | 3 | 8 | Biweekly phone calls (Week 1-12); Two single sessions at the beginning and end of the Intervention phase | "The number of personal contacts and counseling sessions was the same for all study participants overall, but individuals in the ICR and CCR arms received longer and more comprehensive counseling sessions with personalized dietary plans, specific for the ICR or CCR regimens." | Yes |
| Seligman 2011 | Standard-of-care strategy | Diet only |  | Nutrition Edu. |  | Unclear | Individual | Print |  | 3 | 3 |  |  |  | Yes |
|  | Healthy diet and step counter | Diet and exercise |  | Nutrition Edu. |  | Unclear | Individual | Face to Face; Print |  | 3 | 3 | 2 |  |  | No |
|  | Healthy diet and fitness | Diet and exercise |  | Nutrition Edu. |  | Unclear | Individual | Face to Face; Print |  | 3 | 3 | 2 |  |  | Yes |
| Snel 2012 | VLCD only | Diet only | No | MR-F; Nutrition Edu. |  | Unclear | Unclear | Face to Face | Health Care | 4 | 4 |  |  |  | No |
|  | VLCD + exercise | Diet and exercise | No | MR-F; Nutrition Edu. | Physiotherapist | Unclear | Unclear | Face to Face | Health Care; Home | 4 | 4 | 16 | Weekly at minimum | One-hour supervised exercise sessions plus at least 4 home training sessions | No |
| Solbrig 2019 | Motivational interviewing | Diet and exercise | Yes |  | Psychologist/ Counsellor | No | Individual | Face to Face; Telephone; Print | Community | 6 | 6 | 13 | 2 sessions after baseline assessment and fortnightly calls up to 6 months | Session 1: 1 hour Session 2: 35min phone calls: 5–15 min | Yes |
|  | Functional imagery training | Diet and exercise | Yes |  | Psychologist/ Counsellor | No | Individual | Face to Face; Telephone; App | Community | 6 | 6 | 13 | 2 sessions after baseline assessment and fortnightly calls up to 6 months | Session 1: 1 hour Session 2: 35min phone calls: 5–15 min | Yes |
| Somers 2012 | Standard Care | Control | No |  |  | No | Unclear | Unclear | Health Care |  |  |  |  |  |  |
|  | Lifestyle behavioral weight management intervention only | Diet and exercise | Yes | Nutrition Edu.; Help following programme end | Psychologist/ Counsellor; Exercise physiologist | Yes | Group | Face to Face; Telephone; Unclear | Community | 12 | 6 | 21 | Group sessions (12 weekly, 12 biweekly); 3 exercise sessions in 12 weeks; 6 monthly maintenance calls | Group sessions (60 minutes); Exercise sessions (90 minutes); Maintenance calls (20 minutes) | Yes |
|  | Lifestyle behavioral weight management intervention + Pain Coping Skills Training | Diet and exercise | Yes | Nutrition Edu.; Help following programme end | Psychologist/ Counsellor; Exercise physiologist | Yes | Group | Face to Face; Telephone; Unclear | Community | 12 | 6 | 21 | Group sessions (12 weekly, 12 biweekly); 3 exercise sessions in 12 weeks; 6 monthly maintenance calls | Group sessions (120 mins); Exercise sessions (90 mins); Maintenance calls (20 mins) | Yes |
|  | Pain Coping Skills Training only (N/A) | N/A | N/A | N/A | N/A | N/A | N/A | N/A | N/A | N/A | N/A | N/A | N/A | N/A | N/A |
| Spring 2017 | Control self-guided program | Diet and exercise | Yes | Nutrition Edu.; Fin. Incentives; |  | Unclear | Group | Face to Face; Print; Video | Community; Home | 6 | 6 | 1 | Single session | 60 | No |
|  | Standard weight loss program | Diet and exercise | Yes | Nutrition Edu.; Fin. Incentives | Psychologist/ Counsellor; Exercise physiologist | Unclear | Individual and Group | Face to Face; Telephone; Print | Community | 6 | 6 | 19 | Weekly group sessions and calls (Months 1-2); Monthly calls (Months 3-6) | Group session (90 mins); Guided walking exercise (30 mins); Telephone calls (10-15 mins) | Yes |
|  | Technology-supported | Diet and exercise | Yes | Nutrition Edu.; Fin. Incentives | Psychologist/ Counsellor; Exercise physiologist | Unclear | Individual and Group | Face to Face; Telephone; Internet; App; Print; SMS | Community; Home | 6 | 6 | 19 | Weekly group sessions and calls (Months 1-2); Monthly calls (Months 3-6) | Group session (90 mins); Guided walking exercise (30 mins); Telephone calls (10-15 mins) | Yes |
| Stahre 2005 | Control | Control | No |  |  |  |  |  |  |  |  |  |  |  |  |
|  | Cognitive treatment | Diet only | No | Nutrition Edu. |  |  | Group | Face to Face | Health Care | 2.3 | 6 | 10 | Weekly | 3 hours | Yes |
| Stahre 2007 | Control Group (weight-reducing program | Diet and exercise | No | Nutrition Edu. | Nurse (General); Physician; Physiotherapist; Other | No | Group | Face to Face | Community | 2.3 | 2.3 | 10 | Weekly | 2 hours | No |
|  | Cognitive treatment group | Diet only | No | Nutrition Edu. | Other AHPs | Unclear | Group | Face to Face | Community | 2.3 | 2.3 | 10 | Weekly | 2 hours | No |
| Stenius-Aarniala 2000 | Control | Diet only | No | Nutrition Edu. |  | No | Group | Face to Face | Health Care | 3.2 | 3.2 | 12 | Weekly | 30 |  |
|  | Treatment with VLCD | Diet only | No | MR-F; Nutrition Edu. |  | Yes | Group | Face to Face | Health Care; Home | 3.2 | 3.2 | 12 | Weekly sessions for 14 weeks, 8 weeks VLCD |  |  |
| Stevens 1993 | Control | Control | No |  |  | No |  |  |  | 0 | 0 | 0 |  |  | No |
|  | Intervention | Diet and exercise | Yes | Nutrition Edu.; Help following programme end | Psychologist/ Counsellor; Dietitian; Exercise physiologist | Unclear | Individual and Group | Face to Face; Telephone; Internet; Print | Community | 18 | 3 | 54 |  | 90 minutes group, individual length not reported.  'The intervention started with an individual counseling session, followed by 14 weekly group meetings led by dietitians or health educators. After this 14-week intensive phase, participants attended six biweekly group meetings and then monthly group meetings. Beginning in the 18th month, participants were offered a variety of options to keep them involved in the intervention, including individual counseling sessions and special group sessions focused on selected weight loss topics.' | Yes |
| Stevens 2001 | Control | Control | No |  |  | Unclear |  |  |  |  |  | 0 |  |  |  |
|  | Intervention | Diet and exercise | Yes | Nutrition Edu.; Help following programme end | Dietitian; Health Trainer | Unclear | Individual and Group | Face to Face; Telephone; Print; Other | Community | 36 | 6 | 51 | Intensive phase (0-6 Months): 1 individual, 14 weekly, 6 biweekly sessions. Extended phase (7-36 Months): biweekly contacts with monthly face-to-face meetings until the intensive intervention is completed for the first cohort then mini-modules to be offered with continued biweekly contact. Specifically tailored follow-up where indicated. | 90 mins in first phase | Yes |
|  | Sodium only intervention (N/A) | N/A | N/A | N/A | N/A | N/A | N/A | N/A | N/A | N/A | N/A | N/A | N/A | N/A | N/A |
|  | Combined intervention (N/A) | N/A | N/A | N/A | N/A | N/A | N/A | N/A | N/A | N/A | N/A | N/A | N/A | N/A | N/A |
| Strobl 2013 | Control, Usual care | Diet and exercise | No | Nutrition Edu. |  | Yes | Individual and Group | Face to Face | Inpatient | 0.7 | 0.7 | NR |  | 3-week treatment (nutrition therapy, physical exercise, and psychoeducation), number and length of sessions not stated. | Yes |
|  | Telephone aftercare | Diet and exercise | Yes | Nutrition Edu. | Personal Trainer | Yes | Individual and Group | Face to Face; Telephone | Inpatient; Health Care; Home | 6 | 0.7 | NR + 8 |  | 3-week treatment (nutrition therapy, physical exercise, and psychoeducation), number and length of sessions not stated. PLUS 8 sessions [1 x 50 min group session; 1 x 10 min individual; 6 x 5-10 min telephone call]. | Yes |
| Tapsell 2017 | Usual care (Control) | Diet and exercise | Yes | Nutrition Edu. | Nurse (General) | Unclear | Individual | Face to Face; Telephone; Print | Health Care | 12 | 3 | 11 | Months 1-3: Monthly;  Months 1 – 12: Quarterly;  Phone calls: Quarterly | 30 mins clinics; 15 min phone calls | Yes |
|  | Intervention Group | Diet and exercise | Yes | Nutrition Edu. | Dietitian; Health Trainer | Yes | Individual | Face to Face; Telephone; Print | Health Care | 12 | 3 | 11 | Months 1-3: Monthly;  Months 1 – 12: Quarterly;  Phone calls: Quarterly | 1 hour clinics; 15 min phone calls | Yes |
|  | Intervention plus food supplement group (N/A) | N/A | N/A | N/A | N/A | N/A | N/A | N/A | N/A | N/A | N/A | N/A | N/A | N/A | N/A |
| TarragaMarcos 2017 | G3 | Diet and exercise | No | Nutrition Edu. |  | Unclear | Group | Face to Face | Health Care |  |  | 1 |  |  | No |
|  | G2 | Diet and exercise | Yes | Nutrition Edu. |  | Unclear | Group | Face to Face; Internet; Other | Health Care; Home | 12 | 3 | 6 | After the initial visit, visits were scheduled after 15 days, 1m, 3m, 6m and one year. |  | No |
|  | G1 | Diet and exercise | Yes | Nutrition Edu.; Help following programme end | Nurse (General) | Unclear | Group | Face to Face | Health Care | 8 | 3 | 10 | Every two weeks from weeks, 1 to 12 and then monthly from weeks 13 to 32 | 1 hour | No |
| Teeriniemi 2018 | Control | Control | No | Nutrition Edu. |  | No | Other | Print | Health Care |  |  | 0 |  |  | Yes |
|  | SHG Counselling | Diet and exercise | No | Nutrition Edu. | Nurse (General) | Yes | Group | Face to Face | Community | 0.7 | 0.7 | 2 |  | 90 | No |
|  | CBT Counselling | Diet and exercise | Yes | Nutrition Edu. | Nutritionist | No | Group | Face to Face | Community | 4.1 | 4.1 | 8 | 7 sessions every second week, last session after 1 month | 90 | No |
|  | Control plus HBCSS | Diet and exercise | No | Nutrition Edu.; Help following programme end |  | No | Other | Internet; Print | Health Care; Community | 12 | 12 | 0 |  | 52-week access to Web-based HBCSS | Yes |
|  | SHG Counselling plus HBCSS | Diet and exercise | No | Nutrition Edu.; Help following programme end | Nurse (General) | Yes | Group | Face to Face; Internet | Community | 12 | 12 | 2 |  | 90 minutes 52-week access to Web-based HBCSS | Yes |
|  | CBT Counselling plus HBCSS | Diet and exercise | Yes | Nutrition Edu.; Help following programme end | Nutritionist | No | Group | Face to Face; Internet | Community | 12 | 12 | 8 | 7 sessions every second week, last session after 1 month | 90 minutes 52-week access to Web-based HBCSS | Yes |
| ter Bogt 2009 | GP usual care | Control | No |  | GP | Unclear | Individual | Face to Face | Health Care |  |  | 1 |  | 10 | No |
|  | Lifestyle counselling from NP | Diet and exercise | Yes | Nutrition Edu. | Nurse (General) | Yes | Individual | Face to Face; Telephone | Health Care | 36 | 8 | 11 | Four visits (at months 1, 2, 3, 8); 1 telephone call (5 months) in the first year; one visit and one telephone call per year (year 2, 3) | Average duration of the visits was 35 minutes for the first and second visit (range 15–60 minutes) and 25 minutes for the third visit (range 15–40 minutes). | Yes |
| The Look AHEAD Research Group 2010 | Diabetes support and education | Diet and exercise | No | Nutrition Edu.; Help following programme end | Nurse (General); Dietitian; Health Trainer; Personal Trainer | Yes | Group | Face to Face; Telephone; Print; Other | Community |  | 48 | 22 | 3 sessions annually for the first 4 years of follow-up; thereafter, one session was provided annually | 60 – 90 | No |
|  | Intensive lifestyle intervention | Diet and exercise | Yes | MR-P; Nutrition Edu.; Help following programme end | Nurse (General); Physician; Psychologist/ Counsellor; Dietitian; Personal Trainer | Yes | Individual and Group | Face to Face; Telephone; Internet; Print; Other | Community | 115 | 12 | 134 | Months: 1-6: weekly;  Months 7-12: 3/month;  Years 2-4: Minimum of 1/month;  Year 5+: Monthly recommended. | Months 1-6: Group sessions: 60 to 75 minutes; Individual sessions: 20 to 30 minutes. | Yes |
| Trepanowski 2017 | No-intervention control group | Control | No |  |  | Unclear |  |  | Community |  |  | 0 |  |  | No |
|  | Daily calorie restriction group | Diet only | No | Nutrition Edu.; Help following programme end | Dietitian; Nutritionist | Unclear | Individual | Face to Face; Other | Community | 12 | 6 | 14 | Counselling: Months 4 – 6: weekly;  Months 6 – 12: monthly |  | Yes |
|  | Alternate-day fasting group | Diet only | No | Nutrition Edu.; Inter. Fasting; Help following programme end | Dietitian; Nutritionist | Unclear | Individual | Face to Face; Other | Community | 12 | 6 | 14 | Counselling: Months 4 – 6: weekly;  Months 6 – 12: monthly |  | Yes |
| Tsai 2010 | Control | Control | No | Nutrition Edu. | GP | No | Individual | Face to Face; Print | Health Care | 12 | 12 | 4 | Quarterly | 2 – 3 | No |
|  | Brief counselling | Diet and exercise | No | Nutrition Edu. | Other AHPs; Health care professional (not specified) | Yes | Individual | Face to Face; Telephone; Print | Health Care | 12 | 6 | 12 | PCP visits: quarterly. MA visits: weeks 0, 2, 4, 8, 12, 16, 20, 24 | 15 – 20 | Yes |
| Tuomilehto 2009 | Control | Diet and exercise | No | Nutrition Edu. | Nurse (General); Physician | Yes | Individual |  |  | 12 |  | 3 | At baseline, 3ms and 12m |  | No |
|  | Intervention | Diet and exercise | Yes | MR-F; Nutrition Edu. | Nutritionist; Physiotherapist | Unclear | Individual and Group | Face to Face | Health Care; Home | 12 | 3 | 14 | Every 2 weeks until week 12 then monthly | 60 – 90 | Yes |
| van de Glind 2017 | Comparison group | Control | No | Nutrition Edu. |  | No |  | Print | Community |  |  |  |  |  | No |
|  | EuroFIT group | Diet and exercise | No | Nutrition Edu.; Help following programme end | Health Trainer | Yes | Group | Face to Face; App; Print; Other | Community | 6 - 9 | 3 | 13 | Weekly for Weeks 1 to 12; One reunion meeting held 6–9 months after the program end. | 90 | Yes |
| vanWier 2011 | Control – Brochure | Control | No | Nutrition Edu. |  |  | Other – information booklet |  |  |  |  |  |  |  | No |
|  | Internet Group | Diet and exercise | No | Nutrition Edu. | Psychologist/ Counsellor; Health Trainer | Yes | Individual | Internet | Workplace; Home | 6 | 6 | 10 | Every 2 weeks | Work on module on internet. Email contact after completion of each module. | No |
|  | Phone Group | Diet and exercise | No | Nutrition Edu. | Psychologist/ Counsellor; Health Trainer | Yes | Individual | Telephone | Workplace; Home | 6 | 6 | 10 | Every 2 weeks | Call every 2 weeks. Work on modules individually in between calls. | No |
| Vissers 2010 | Control | Control | No |  |  | No |  |  |  |  |  |  |  |  |  |
|  | Diet only group (Diet) | Diet only | Yes | Nutrition Edu. | Dietitian | Unclear | Individual | Face to Face | Community | 12 | 3 | 12 | During the first 3 months participants had a dietary counseling every fortnight. During the next 3 months there was a dietary counseling once a month. 3 more visits months 6-12 |  | Yes |
|  | Diet + fitness training group (Fitness) | Diet and exercise | Yes | Nutrition Edu. | Dietitian; Physiotherapist | Unclear | Individual and Group | Face to Face | Health Care; Community; Home | 12 | 3 | 51 | As per diet only plus 2 x week for first 3m, 1 x week for second 3m |  | Yes |
|  | Diet + WBV group (Vibration) | Diet and exercise | Yes | Nutrition Edu. | Dietitian; Physiotherapist | Unclear | Individual and Group | Face to Face | Community; Home | 12 | 3 | 51 | As per diet only plus 2 x week for first 3m, 1 x week for second 3m |  | Yes |
| von Gruenigen 2012 | Control | Control | No | Nutrition Edu.; |  | Unclear | Individual | Face to Face; Print | Health Care |  |  | 1 | Once |  | No |
|  | Intervention | Diet and exercise | Yes | Nutrition Edu. | Physician; Psychologist/ Counsellor; Dietitian; Physiotherapist | Unclear | Individual and Group | Face to Face; Print; Internet | Health Care | 12 | 6 | 16 | Group sessions (10 weekly followed by 6 bi-weekly); Physician face-to-face counseling visits occurred at 3, 6 and 12 months | Group sessions were 60 min | Yes |
| von Gruenigen 2008 | Control, Usual care | Control | No | Nutrition Edu. |  |  | Unclear | Print | Health Care |  |  | 0 |  |  | No |
|  | Lifestyle intervention | Diet and exercise | Yes | Nutrition Edu.; Help following programme end | Psychologist/ Counsellor; Dietitian; Other |  | Individual and Group | Face to Face; Telephone; Print | Health Care | 6 | 6 | 24 | Weekly for 6 weeks, bi-weekly for I month, and monthly for 3 months. |  | Yes |
| West 2007 | Attention control | Diet and exercise | Yes | Nutrition Edu.; Help following programme end | Nutritionist; Exercise physiologist; Health Trainer | No | Group | Face to Face |  | 18 | 6 | 47 | Weekly for 6m, Biweekly for 6m, and then monthly for 6m. | 45 | No |
|  | Motivational interviewing | Diet and exercise | Yes | Nutrition Edu.; Help following programme end | Psychologist/ Counsellor; Nutritionist; Exercise physiologist; Health Trainer | No | Individual and Group | Face to Face |  | 18 | 6 | 47 | Weekly for 6m, Biweekly for 6m, and then monthly for 6m. Five individual motivational interviewing sessions were offered, with the first session before starting group therapy and then at 3, 6, 9, and 12m. | 45 | Yes |
| West 2011 | Control | Control | No |  |  |  |  |  |  |  |  |  |  |  | No |
|  | Lifestyle Intervention | Diet and exercise | No | Nutrition Edu.; Help following programme end | Health Trainer | Yes | Group | Face to Face; Print | Community | 12 | 4 | 20 | Weekly for first 4m (12 weeks?), then monthly for 8m | 60 | Yes |
| Whelton 1998 | Non-weight loss (Usual lifestyle, control group plus sodium reduction) | Control | No |  |  | Unclear | Group | Face to Face | Community | 12 | 12 | 3 | Quarterly for 1 year |  | No |
|  | Weight loss (Weight loss alone plus weight loss and sodium reduction combined intervention) | Diet and exercise | Yes | Nutrition Edu.; | Nutritionist; Personal Trainer | Unclear | Individual and Group | Face to Face; Telephone; Print | Community | 30 | 8 | 46 | Weekly during the intensive phase (Months 0-4); Biweekly during the extended phase (Months 5-8); Monthly during the maintenance phase. |  | Yes |
| Wilson 2016 | Control - Self Study Group | Diet and exercise | No | Nutrition Edu. |  | No | Other – self-study | Print | Home |  |  | 1 |  |  | No |
|  | Phone Fuel Your Life | Diet and exercise | Yes | Nutrition Edu. | Health Trainer | No | Individual | Telephone; Print | Home | 12 | 6 | 11 | 0-2m: biweekly.  2-6m: monthly.  6-12m: bimonthly. | 20 mins' 8 sessions with a health coach | Yes |
|  | Group Fuel Your Life | Diet and exercise | Yes | Nutrition Edu. | Health Trainer | No | Group | Face to Face; Print | Workplace | 12 | 6 | 11 | 0-2m: biweekly.  2-6m: monthly.  6-12m: bimonthly. | 60 mins' 8 sessions with a health coach | No |
| Wilson 2016b | Control | Control | No |  |  | No |  |  |  |  |  |  |  |  | No |
|  | FUEL Your Life peer health coaches + nurse education | Diet and exercise | No | Nutrition Edu. | Nurse (General); Dietitian; Health Trainer | Yes | Individual and Group | Face to Face; Print | Workplace | 6 | 6 | 6 | Monthly | Baseline: initial 1:1 session 0-6m: 6 x 10 min group sessions and weekly announcements. | Yes |
| Wing 1998 | Control | Diet and exercise | No | Nutrition Edu. |  | No |  | Print |  |  |  |  |  |  | No |
|  | Diet | Diet only | No | Nutrition Edu.; Help following programme end | Psychologist/ Counsellor; Dietitian | No | Group | Face to Face | Community; Home | 24 | 6 | 51 | Weekly for the first 6m;  Biweekly for the next 6m |  | No |
|  | Exercise | Exercise only | No |  | Psychologist/ Counsellor; Exercise physiologist | No | Group | Face to Face | Community; Home | 24 | 6 | 51 | Weekly for the first 6m;  Biweekly for the next 6m | 50 – 60 min walk with the therapist at each of these weekly meetings. | No |
|  | Diet plus exercise | Diet and exercise | No | Nutrition Edu.; Help following programme end | Psychologist/ Counsellor; Dietitian; Exercise physiologist | No | Group | Face to Face | Community; Home | 24 | 6 | 51 | Weekly for the first 6m;  Biweekly for the next 6m |  | No |
| Yannakoulia 2008 | Usual care group | Control | No | Nutrition Edu. | Dietitian | No | Individual | Face to Face | Health Care |  |  | 1 |  |  | Yes |
|  | Intensive care group | Diet and exercise | No | Nutrition Edu. | Dietitian | No | Individual | Face to Face | Health Care | 2 | 2 | 5 | Every two weeks |  | Yes |
| Yardley 2014 | Usual care | Control | No |  | Other AHPs | No |  |  | Health Care |  |  | 0 |  |  |  |
|  | Web-based only | Diet and exercise | No | Nutrition Edu.; Help following programme end |  | No | Individual | Internet; Other | Health Care; Home | 3 | 3 | 0 | Instructed to access website weekly |  | Yes |
|  | Basic nurse support | Diet and exercise | Yes | Nutrition Edu.; Help following programme end | Nurse (General) | No | Individual | Face to Face; Telephone; Internet | Health Care; Home | 3 | 3 | 3 | 2 weeks, 1m, 3m | 15 – 20 | Yes |
|  | Regular nurse support | Diet and exercise | No | Nutrition Edu.; Help following programme end | Nurse (General) | No | Individual | Face to Face; Telephone; Internet | Health Care; Home | 6 | 6 | 7 | 2 weeks, and then monthly for the first 6m | 15 – 20 | Yes |
| Yates 2009 | Control group | Control | No |  |  | No |  | Print |  |  |  | 0 |  |  | No |
|  | PREPARE group | Diet and exercise | No | Nutrition Edu. | Health Trainer | No | Individual and Group | Face to Face | Community | 6 | 6 | 3 | 1, 3 and 6m | The first session lasted 180 min and the follow-up review progress lasted 10 mins. | Yes |
|  | PREPARE with pedometer | Diet and exercise | No | Nutrition Edu. | Health Trainer | No | Individual and Group | Face to Face | Community | 6 | 6 | 3 | 1, 3 and 6m | The first session lasted 180 min and the follow-up review progress lasted 10 mins. | Yes |
| Yates 2018 | Placebo + no lifestyle | Control | No |  |  |  |  |  |  |  |  |  |  |  |  |
|  | Metformin + no lifestyle (N/A) | N/A | N/A | N/A | N/A | N/A | N/A | N/A | N/A | N/A | N/A | N/A | N/A | N/A | N/A |
|  | Placebo + lifestyle | Diet and exercise | No | Nutrition Edu. |  | No | Individual and Group | Face to Face |  | 4 | 4 | 16 | Weekly |  | Yes |
|  | Metformin + lifestyle (N/A) | N/A | N/A | N/A | N/A | N/A | N/A | N/A | N/A | N/A | N/A | N/A | N/A | N/A | N/A |
| Yeh 2016 | Control group | Control | No |  |  | No |  |  |  |  |  |  |  |  |  |
|  | Intervention group | Diet and exercise | Yes | Nutrition Edu. | Health Trainer | No | Group | Face to Face | Community | 12 | 6 | 18 | Every second week the first six months and monthly during the second semester | 1.5-2 hours | Yes |
| Yin 2018 | Comparison-Control Group | Diet and exercise | No | Nutrition Edu. | Nurse (General) | No | Individual and Group | Face to Face | Health Care | 6 | 6 | 7 | Every three-four weeks | Participants in the comparison group received a counselling session and were invited to attend 6 general health education classes on PA, nutrition, chronic diseases (obesity, diabetes, heart diseases) and menopause at the same venue as the intervention group. |  |
|  | Intervention Group | Diet and exercise | No | Nutrition Edu. | Health Trainer | Yes | Individual and Group | Face to Face; Telephone | Health Care; Home | 6 | 6 | 22 | Weekly | 1 hour | Yes |
| Zhang 2016 | Control | Control | Yes |  | Other | No | Group | Face to Face |  | 12 | 12 | 18 | Biweekly | All participants attended group health education sessions, which were held biweekly in the first 6m and monthly in the last 6m of the intervention. | No |
|  | Moderate exercise | Exercise only | No |  | Other | No | Other - education sessions: group-based; moderate-exercise sessions unsupervised | Face to Face; Telephone | Home | 12 | 12 | 18 | Biweekly and weekly | Participants were instructed to briskly walk at approximately 120 steps per minute for 30 minutes per session and 5 sessions per week.  All participants attended group health education sessions, which were held biweekly in the first 6m and monthly in the last 6m of the intervention.  plus, Participants in the moderate exercise program were required to wear pedometers and record their daily exercise in a log, which was reviewed weekly by study staff. | No |
|  | Vigorous-moderate exercise | Exercise only | No |  | Physician; Other | No | Individual and Group | Face to Face; Telephone | Community; Home | 12 | 12 | 138 | Biweekly and weekly | Participants were required to participate in 5, 30-min., vigorous exercise sessions each week supervised by a study physician at a local community health center.  All participants attended group health education sessions, which were held biweekly in the first 6m and monthly in the last 6m of the intervention.  Participants were required to participate in 5 vigorous exercise sessions each week supervised by a study physician at a local community health center. After 6 months of vigorous exercise, participants switched to moderate exercise for another 6 months.  plus, Participants in the moderate exercise program were required to wear pedometers and record their daily exercise in a log, which was reviewed weekly by study staff. | No |
| **Approx.:** Approximately; **Appt.**: Appointment/s; **Fin. Incentives;** Financial Incentives**; GP:** General Practitioner **Inter. Fasting:** Intermittent Fasting; **Min/s:** Minute/s **M/Mths:** month/s**; MR – F =** Meal replacement (Full); **MR – P =** Meal replacement (Partial); **N:** Number; **N/A:** Not applicable; **NR:** Nor reported; **Nutrition Edu.** = Nutrition Education; **PA:** Physical Activity**; SMS**: Short Message Service; **VLCD:** Very low-calorie diet  ^a^ See table below for Provider category descriptions ^b^ Unless otherwise stated; ^c^ Exercise sessions were assumed to be unsupervised and did not contribute to the number of sessions unless otherwise stated.   \| **Provider** \| **Provider descriptions as reported in included studies** \| \| --- \| --- \| \| Nurse (Specialist) \|  \| \| Nurse (General) \| Nurse educator;  RNS; \| \| GP \| General internists \| \| Physician  (Any doctor not a GP) \| Medical doctors; Specialists in endocrinology, and internal medicine; Clinicians; Endocrinologists; Graduates in medicine; Research cardiologist; Doctoral-level clinicians (with an average of 4.8 years of experience delivering behavioral weight loss treatment); Occupational doctor. \| \| Psychologist/ Counsellor \| Therapist; Masters-level counseling psychology students; MA in behavioural psychology; Lifestyle counsellor; Graduates In psychology; Psychology graduate students; Advanced degree in behavioral psychology; Mental health counsellor; Wellness counsellors; Professional Counsellor; Psychotherapist; Psychotherapists and masters students graduate students in clinical psychology; Clinical psychology graduate students; Lifestyle counsellor; Clinical psychology graduate students; Experienced behavioural weight control counsellors; Behavior therapist; Counsellor with a degree in nutrition or physical activity \| \| Dietitian \| Dietitian; Masters of Dietetics Students \| \| Nutritionist \| Provider described by authors as nutritionist; Nutrition technician; Graduates in nutrition; Advanced degree in nutrition; Nutritional interventionist; Nutritionist (MSc in nutrition); Nutrition/Diet interventionists; Two qualified or student clinical nutritionists \| \| Physiotherapist \| Physical therapist; Physical/recreational therapists \| \| Exercise physiologist \| Exercise consultants; MA in exercise physiology; Graduates in physical activity and sport science (SPAS); Advanced degree in exercise physiology; Exercise counsellors \| \| Other Allied Health Professionals \| Occupational therapist; Pharmacist; Nurses/physician assistants; Hospital staff; Social worker with special competence in CT; Medical-assistant \| \| Health trainer \| Lifestyle coaches; Mindfulness meditation instructors; Community health educator; MA in health education; Behavioural consultant; Health educator; Telephone counsellors; Trained lifestyle coaches; Health Promotion coaches; Weight loss coaches; Wellness leader; Weight Watchers leader; Trained interventionists with expertise in both content area (i.e., physical activity and nutrition) and behavioral therapy;  Food advisors recruited from local community; Community Health Workers; Lifestyle activity consultant; Trained lifestyle coaches; Lifestyle Coach/ medical assistant; Masters-level staff with extensive training in behavioral weight loss; Nutrition health educator; IHM health staff graduates; 6 trained CAMWEL advisors recruited from various occupational backgrounds including healthcare, in line with the NHS health trainers initiative; Weight loss group leaders supervised by an exercise physiologist; Study coordinator (with health/nutrition background) together with a peer leader/study coordinator (experienced in adult training and self-management programs); Health educator; Degree in health sciences; Trainers (for meal replacement group); Health coach and health practitioner backgrounds and trained by the senior psychologists; Diabetes educators; EuroFIT coaches; Program providers who were trained in nutrition, education, and behavioral interventions; Masters degree–level health educators delivered health education sessions; Behaviorist;  Trained lay health educators (LHEs) (community volunteers or existing senior center staff); Peer health coach; Educators held an undergraduate degree in a relevant discipline (dietician, sports scientist) \| \| Personal Trainer \| Certified exercise trainer; Trained fitness instructor; Physical activity specialist; Football coaching staff; Physical Activity Counselor; Trained interventionist and exercise coaches who were skilled in exercise science; Exercise programme supervised by a professional trainer; Fitness professional; Exercise interventionists; Exercised in a supervised setting; Trained certified technicians assessed each participant;  Sports therapist; Exercise specialists; \| \| Health care professional (not specified) \| Church health advisors (CHAs) were members of their respective church’s health ministry (e.g., nurses, pharmacists, physicians) and were trained by a co-investigator certified to perform GLB training;  Standard clinical care provider; Hospital based care; Primary care providers; Master's trained health professionals; health professional \| \| Other \| Research staff; Behavioral specialist; PhD-level interventionists; Doctoral level graduate students; Research assistant; Case manager; Coaches; YMCA staff; Peer leader; Teacher; Interventionist; Successful group members selected through interview; Varied, may be successful slimmers; Well-trained investigators; Research assistant; PhD holders or PhD candidates in at least their third year of study; BE WELL intervention staff; Physical activity, psychological support male researcher; The tutors; Study investigator; Ergonomist; Study coordinator; Interventionist; Cooperative Extension Service Family and Consumer Sciences Agents or individuals with bachelors or masters degrees in nutrition, exercise science, or psychology; Study partner; Trained interventionists; Group facilitator; External people representing diverse areas of expertise;  Two experienced coleaders; Administrative study staff (not intervention staff); Trained graduate or undergraduate students; Had backgrounds in dietetics, psychology and/or exercise physiology; Primary investigator; Study staff \| \| If it was an OR between providers, both were listed \| \| | | | | | | | | | | | | | | | |

### Table 8. Sensitivity analyses

| **Outcome** | **Analysis** |
| --- | --- |
| Quality of life (standardized mean) | 35 arms from 29 studies (n = 8,708 participants) were classed as not being at high risk of bias. Removing studies at high risk of bias increased both the estimate of average trend in standardised QoL over time for the random effects and the meta-regression model. For the random intercept model, mean trend decreased from -0.004 to -0.008 (95% CI -0.02 to 0.006) per month, and for the meta-regression model, average change decreased from -0.007 to 0.009 (95% CI -0.03 to 0.08) per month. After excluding studies at high risk of bias, the association between weight difference at last follow-up and difference in standardised QoL was no longer significant with every Kg increase in weight difference leading to an average change (95% CI) in standardised quality of life of -0.06 (-0.14 to 0.02). Removing studies at high risk of bias from the time-to-event model did not alter the estimate of the median time (18 months). |

### Table 9. Cost-effectiveness sensitivity analysis using outputs from meta-regression (model 2): total per person cost of intervention at the threshold of intervention cost-savings and at the threshold range for cost-effectiveness defined by the National Institute for Health and Care Excellence (NICE) for a weight difference of 2.5kg at programme end.

| Threshold | Perspective | |
| --- | --- | --- |
|  | Health care | Health and social care |
| **Linear weight regain after weight loss** | | |
| Cost-saving (£0 per QALY) | **£95 (£0.97 to £620)** | **£200 (£1.9 to £1300)** |
| NICE lower (£20,000 per QALY) | **£890 (£8.90 to £5600)** | **£990 (£9.80 to £6300)** |
| NICE upper (£30,000 per QALY) | **£1300 (£13 to £8000)** | **£1400 (£14 to £8700)** |

# SUPPLEMENTAL FIGURES

### Figure 1. PRISMA diagram of study flow


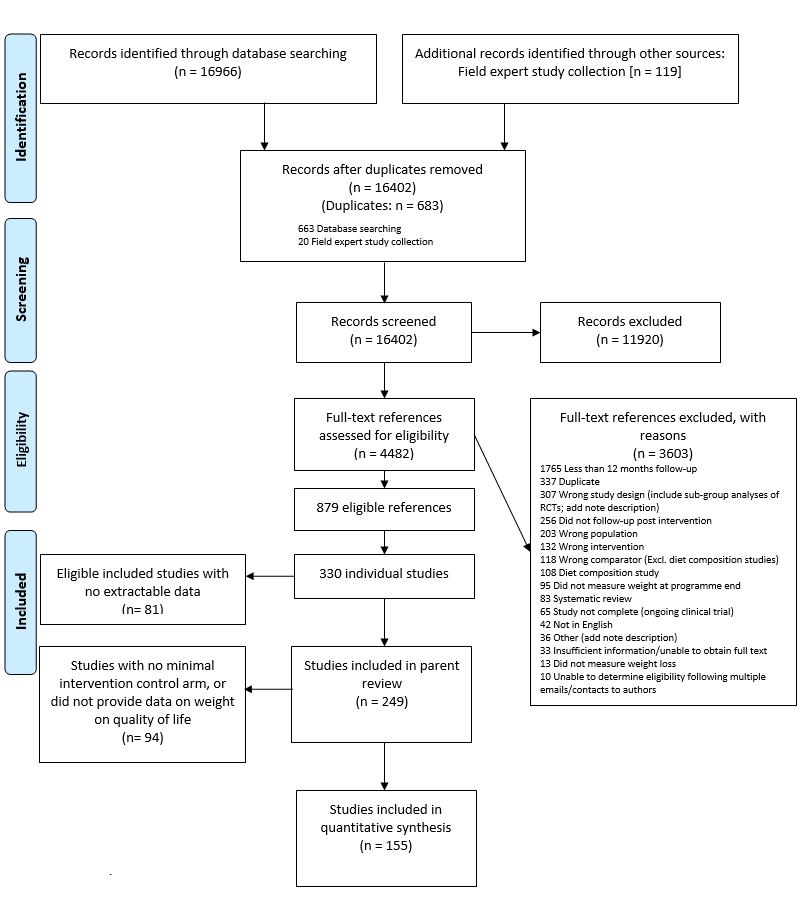


### Figure 2. Mean difference in weight change at programme end mapped against follow-up time


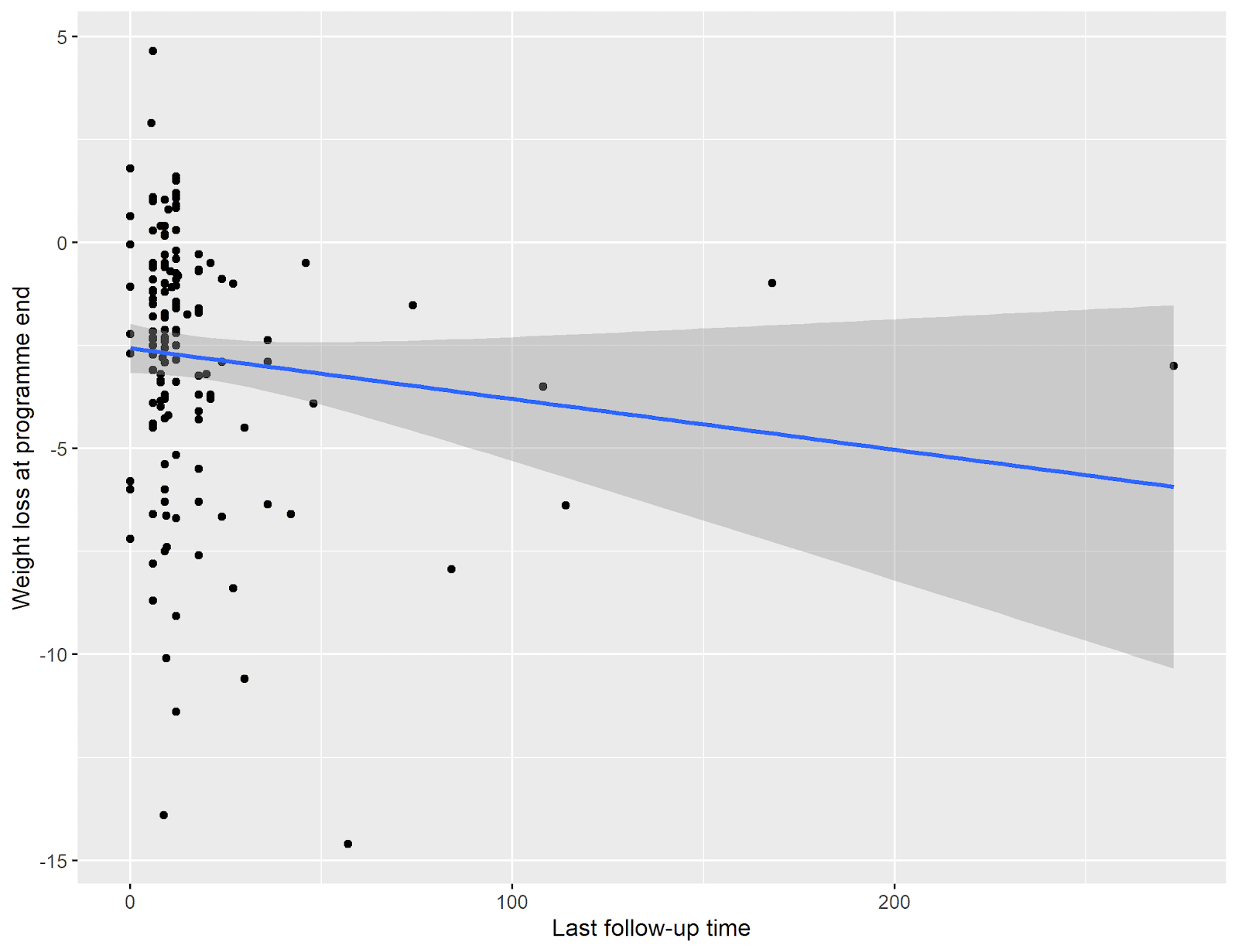


### Figure 3. Weight regain trajectory as per the linear model overlaid on the observed difference in weight change between intervention and comparator arms by time since programme end. Dot size is proportional to number of participants in each study. The red dashed line represents estimates of average trend in weight change difference from the random effects model. The green dotted line represents the meta-regression model.


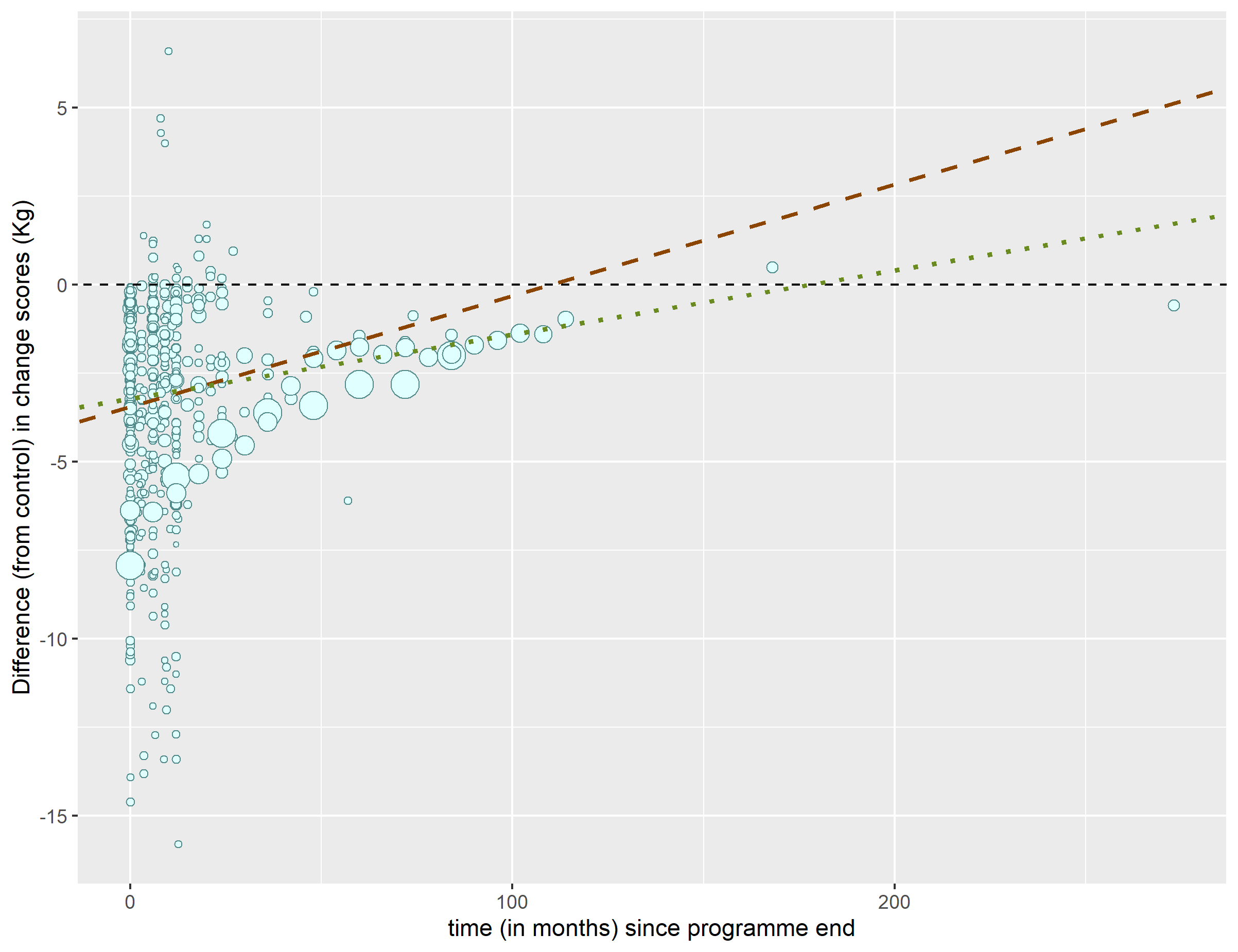


### Figure 4. Kaplan Meier plot showing time for intervention group mean weight to reach that of control group


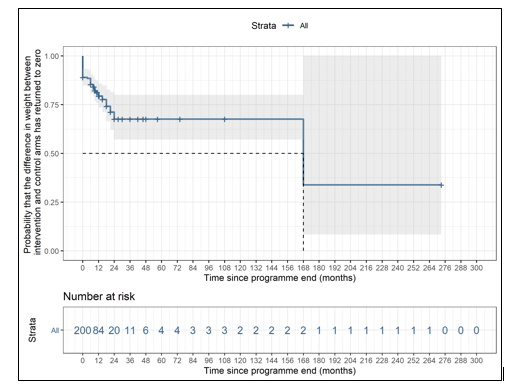


### Figure 5. Difference in standardised quality of life change between intervention and comparator arms by time since programme end (higher = better). Dots size is proportional to number of participants in the study. Dashed lines represent estimates of average trend from model 1 and model 2


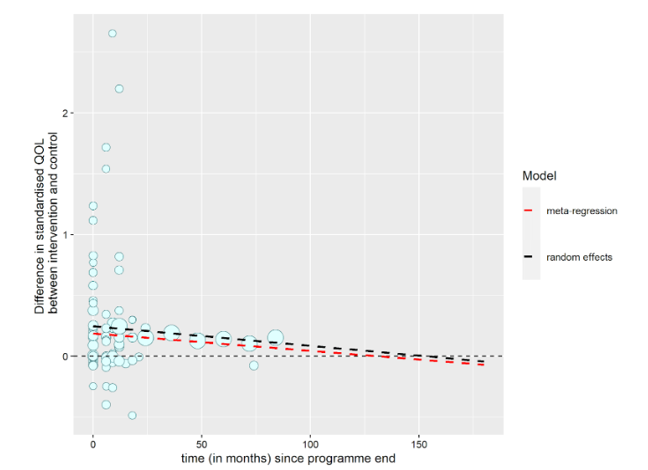


### Figure 6. Kaplan Meier plot showing probability that study arm that had quality of life difference at programme end is zero on follow-up.


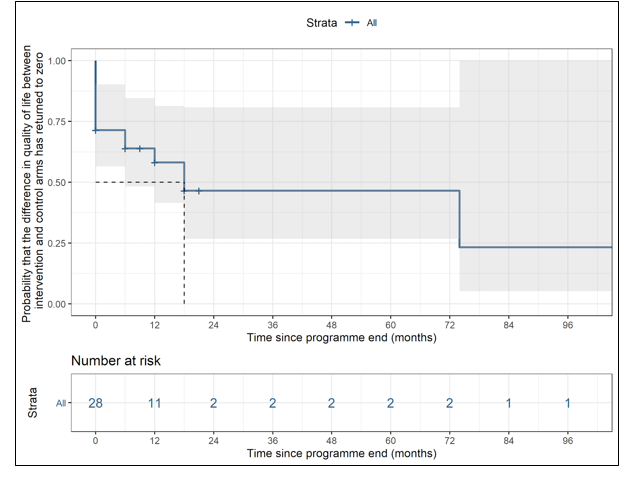


### Figure 7. Tornado plot for the primary outcome, centred on median outcome from 1500 runs of the PRIMEtime model.
